# A systematic review and meta-analysis of diagnostic test accuracy of mental health screening tools applicable to adolescents in sub-Saharan Africa

**DOI:** 10.3389/fpsyt.2026.1728252

**Published:** 2026-06-16

**Authors:** Jean Berchmans Niyibizi, Stefan Jansen, Elin Charlotte Larsson, Japhet Ntabanganyimana, Malachi Ochieng Arunda

**Affiliations:** 1 Department of Global Public Health, Karolinska Institutet, Stockholm, Sweden; 2 Directorate of Research and Innovation, College of Medicine and Health Sciences, University of Rwanda, Kigali, Rwanda

**Keywords:** adolescents, diagnostic test accuracy, mental health screening tools, meta-analysis, sub-Saharan Africa, systematic review

## Abstract

**Introduction:**

Mental health disorders (MHDs) is a global public health concern. Existing evidence highlighted the need for context-specific measurements of MHDs among adolescents in sub-Saharan Africa (SSA). This review aimed to provide an evidence-based inventory of effective mental health (MH) screening tools applicable to adolescents in SSA aged 12–18 years.

**Methods:**

We systematically searched Medline, Web of Science Core Collection, PsycINFO, and CINAHL for validation studies of MH screening tools published between January 1, 2000 and December 31, 2024. Pooled sensitivity and specificity were estimated using hierarchical summary receiver operating characteristic (HSROC) models. Study quality was assessed using the Quality of Diagnostic Accuracy Studies-2 (QUADAS-2) tool. Heterogeneity was evaluated using *I*² statistic and forest plots, while funnel plots and Deeks’ funnel plot asymmetry test were used to assess publication bias. The analyses were conducted in R 4.5.3 version.

**Results:**

Out of the 18,918 screened articles, 128 studies (covering 26 MHDs) were included in the systematic review. Of these, 60% (77/128) were rated high risk of bias on at least one domain of the QUADAS-2 tool. Only 21 of 48 SSA countries validated at least one screening tool. The meta-analysis included 36 articles evaluating four tools—Edinburg Postnatal Depression Scale (EPDS), Patient Health Questionnaire-9 (PHQ-9)/two-step PHQ-9 (PHQ-2/9), two-item PHQ (PHQ-2), or Kessler Psychological Distress Scale (K-10)—which focused on depression only. Heterogeneity was present across validation studies for all tools in the meta-analyses. Both Deeks’ test and visual assessment of funnel plots did not suggest publication bias for EPDS and PHQ-9/PHQ-2/9.

**Discussion:**

Prioritizing the validation of mental health screening tools tailored to adolescents aged 12–18 years is crucial to effectively address the MHDs in SSA. Future research in SSA should focus on adapting and validating existing tools, updating outdated versions, and developing new tools for emerging mental health challenges.

**Systematic review registration:**

https://www.crd.york.ac.uk/prospero/, identifier CRD42023454180.

## 1 Introduction

Mental health disorders (MHDs) are a global public health concern (1). MHDs clinically impair an individual’s emotion regulation, behavior, or cognition, which results in dysfunction in the psychologic, biological and/or developmental processes that are crucial to mental functioning (2). MHDs include conditions such as depression, anxiety, and schizophrenia, among others (3).

According to 2021 estimates from the United Nations International Children’s Emergency Fund (UNICEF), 13% of adolescents aged 10–19 years globally have a mental disorder, which is equivalent to 89 million boys and 77 million girls (3). In the United States, a multisite study revealed that 17.5% of human immunodeficiency virus (HIV)-positive youth are living with psychological symptoms such as anxiety, somatization, and depression (4). A recent sub-Saharan Africa (SSA) systematic review covering epidemiological studies conducted among adolescents and published during the period 2008–2020 reported a depression median prevalence of 26.9% among the general population of adolescents and 29.0% among at-risk adolescents, while the median prevalence of anxiety disorders was 29.8% and 19.3%, respectively (5). In this study, the at-risk adolescents included those affected by HIV and acquired immunodeficiency syndrome (AIDS) and exposed to violence and trauma, poverty, orphanhood, being “out of school”, socioeconomic disadvantages, and high levels of deprivation (5). For emotional and behavioral problems, the median point prevalence was 40.8% among the general population of adolescents and 45.0% among at-risk adolescents (5). For suicidal thoughts, it was 20.8% and 11.6%, respectively, while for post-traumatic stress disorder (PTSD) the median point prevalence was reported for the general population only and was 24% (5). Similarly, Cortina and colleagues reported that 14.3% of children aged ≤16 years in SSA have psychopathologies including depression, anxiety disorders, disruptive conduct, and reactive behavior disorders, and PTSD (6). Another review reported that 25% HIV-positive adolescents have psychiatric disorder, with emotional or behavioral difficulties or psychological distress varying between 30% and 50% (7). These statistics demonstrate the high prevalence of MHDs among SSA adolescents, underscoring the need for attention and care.

Mental disorders in adolescence have a direct negative effect on the adolescents’ health and subsequently on their adulthood and elderly health. The recent report of World Health Organization (WHO, 2023) on adolescents’ and young adults’ health cited self-harm, a symptom and manifestation of a mental illness, as one of the leading causes of deaths among adolescents and that half of adult mental illnesses begin by the age 14 (8). The impact of MHDs’ comorbidity is challenging in the treatment of chronic diseases such as HIV among SSA youth; for instance, in Rwanda, it was reported that youth living with HIV missed or refused antiretroviral therapy (ART) partly due to high rates of psychological symptoms of depression, anxiety, and conduct disorders (9). Similarly, in Ethiopia, skipping and forgetting ART among HIV+ patients was associated with depression (10). This strongly supports the opinion of Stein and his colleagues that non-adherence may provide insight into how mental illnesses aggravate chronic diseases (11). A recent systematic literature review ranked psychosocial needs as the top priority for adolescents living with HIV and receiving ART in SSA (12). Furthermore, the cost–benefit of investing in treatment of mental health issue has been proven; a recent cost–benefit analysis demonstrated that spending $1 on treatment for typical mental health problems returns $4 in better productivity and health (3, 13). Therefore, treating mental disorders at adolescence has lifelong health and economic benefits (14).

Despite this, World Health Organization (WHO) reports that the majority of adolescent cases of mental problems remain undiagnosed and untreated (8), and this exacerbates the issue of MHDs in adolescents in low- and middle-income countries (LMICs), particularly in SSA. This underscores the urgent need for the effective identification and management of MHDs in adolescents, including HIV-infected young people in SSA. A foundational step in solving this challenge is identifying psychometrically validated mental health screening tools that are culturally and contextually appropriate for adolescents in SSA. Reliable tools are essential for healthcare workers and researchers to accurately assess mental health in local target populations.

The existing evidence does not consider MH screening tools applicable to the general population of adolescents in SSA. A review by Ali et al. (15) focused on validated screening tools for common mental disorders (CMDs) in general LMIC populations and appeared to be old because most of the included studies were published 12 years ago, and thus the findings from this review may no longer reflect the current situation (15). Moreover, the study by Ali and colleagues solely focused on brief CMD screening tools (≤21 items), did not specify the language in which the tools were validated, and did not specify the age range, and only a small proportion of the studies, 10%, was screened by two reviewers, raising concerns about the consistency and reliability of the study selection (15). So, the study of Ali et al. (15) did not comply with the guidelines for conducting a systematic review to ensure data quality and accuracy (16). Another more recent systematic review by Orth and van Wyk (17) was limited to adolescents living with a chronic physical condition (18) and did not report the psychometric properties of the tools, thus limiting its utility in recommending validated screening tools, nor suggested further studies to develop screening tools for MHDs among adolescents.

In light of the afore-mentioned gaps of existing systematic reviews on MH screening tools in SSA (15, 16), there is a need for a comprehensive and up-to-date systematic review of validated MH screening tools applicable to adolescents aged 12–18 years in SSA. This age range corresponds with pubertal development and transition to legal and social independence (i.e., the legal definition of “adulthood” in many nations) (19). Although a time of strength and resiliency, adolescence is also when psychiatric disorders frequently start (8, 19). Thus, the aim of this systematic review is to provide researchers, policy makers, and healthcare professionals with an evidence-based inventory of the most appropriate, psychometrically validated MH screening tools applicable to SSA adolescents in the 12–18 years age range.

Importantly, multiple studies suggest that most MH screening tools can be applied to both the general population and those with chronic conditions, including HIV (20–22), Therefore, a review focusing on the general population will allow the inclusion of tools used across diverse health statuses. Since MHDs are more prevalent among HIV-positive youth than their peers, identifying effective tools applicable to all adolescents will help ensure broader reach and relevance (22).

## 2 Materials and methods

This systematic review was conducted in six main steps, namely: (i) formulating an appropriate research question, (ii) performing a comprehensive literature search, (iii) selecting studies for inclusion, (iv) extracting data and assessing the study quality, (v) conducting a narrative synthesis of findings, and (vi) performing a meta-analysis for studies that fulfilled the criteria and interpreting the results (23). The systematic review followed the Preferred Reporting Items for Systematic Review and Meta-analysis (PRISMA) statement (24). The study protocol was registered with PROSPERO (CRD42023454180) but was not published in a peer-reviewed journal.

### 2.1 Eligibility criteria

#### 2.1.1 Inclusion criteria

This review included the studies that (1) were either cross-sectional or prospective cohort designs, (2) aimed to either develop, adapt, or validate screening tool(s)/scale(s) for MH disorders, and (3) targeted adolescents aged 12–18 years or a study population with overlapping age ranges that include a subset of individuals within the age range of 12–18 years. Fourthly, this review considered studies that were conducted in any SSA country (https://twas.org/sub-saharan-african-countries). Furthermore, in case of a multi-country study that included SSA countries, only information from SSA countries were considered in this review. Finally yet importantly, it considered studies that were conducted from January 1, 2000 to December 31, 2024. No language restriction was applied.

As mentioned earlier, this review targeted to identify MH screening tools applicable to adolescents aged 12–18 years and additionally included those validated to a broader age range that either partially or completely overlap the age bracket of 12–18 years. The latter consideration intended to avoid the omission of tools that are commonly used and recommended to screen for MHDs in broader age ranges, which include adolescents, in research and clinical contexts. Considering that this review aimed to provide a comprehensive overview of screening tools that researchers, clinicians, and policy makers could realistically consider to assess MH in adolescents aged 12–18 years, limiting to tools validated exclusively in adolescents would likely have provided incomplete evidence since adolescent-specific validation studies are relatively scarce (15) (25). Therefore, this review considered tools validated in broader age ranges, and that is a pragmatic approach to map tools potentially suitable for adolescents. In this review, we considered the broader age range to sufficiently overlap with the 12–18 years age range if at least three consecutive ages (12–14 or 16–18) were included at either the lower or upper bound, respectively. We also defined an adequate age range if is either exactly 12–18 years or it extends by no more than 2 years beyond either limit of the 12–18 years age range. We set it in that way because the intent of this review was to identify any MH screening tool that was validated for adolescents aged 12–18 years, regardless of whether the tool was validated for a wider age range that includes a subset of ages within the age range of 12–18 years.

#### 2.1.2 Exclusion criteria

Studies in which diagnosis was based on observation checklist, chart review, or self-reported diagnosis were excluded. Studies that did not report sensitivity/specificity or lacked data to calculate these measures were likewise excluded. Intervention studies that focused on treatment, not validation, of MHDs were excluded as well.

### 2.2 Information sources and search strategy

A comprehensive literature search was performed in the following databases: Medline (Ovid), Web of Science Core Collection (Clarivate), PsycINFO (EBOSCO), and CINAHL (EBOSCO). The initial search was conducted on November 6, 2023, updated on October 25, 2024, and finalized on February 20, 2025 to capture peer-reviewed publications from 2000 to 2024.

The search strategy was developed in Medline (Ovid) in collaboration with the librarian at Karolinska Institutet University Library. For each search concept, Medical Subject Headings (MeSH) terms and free text terms were identified. The search was then translated, in part using Polyglot Search Translator (26), into the other databases. The full search strategies are provided in **Supplementary Appendix 1**. Duplicate records were removed using Covidence and Bramer method (27).

**Figure 1:**
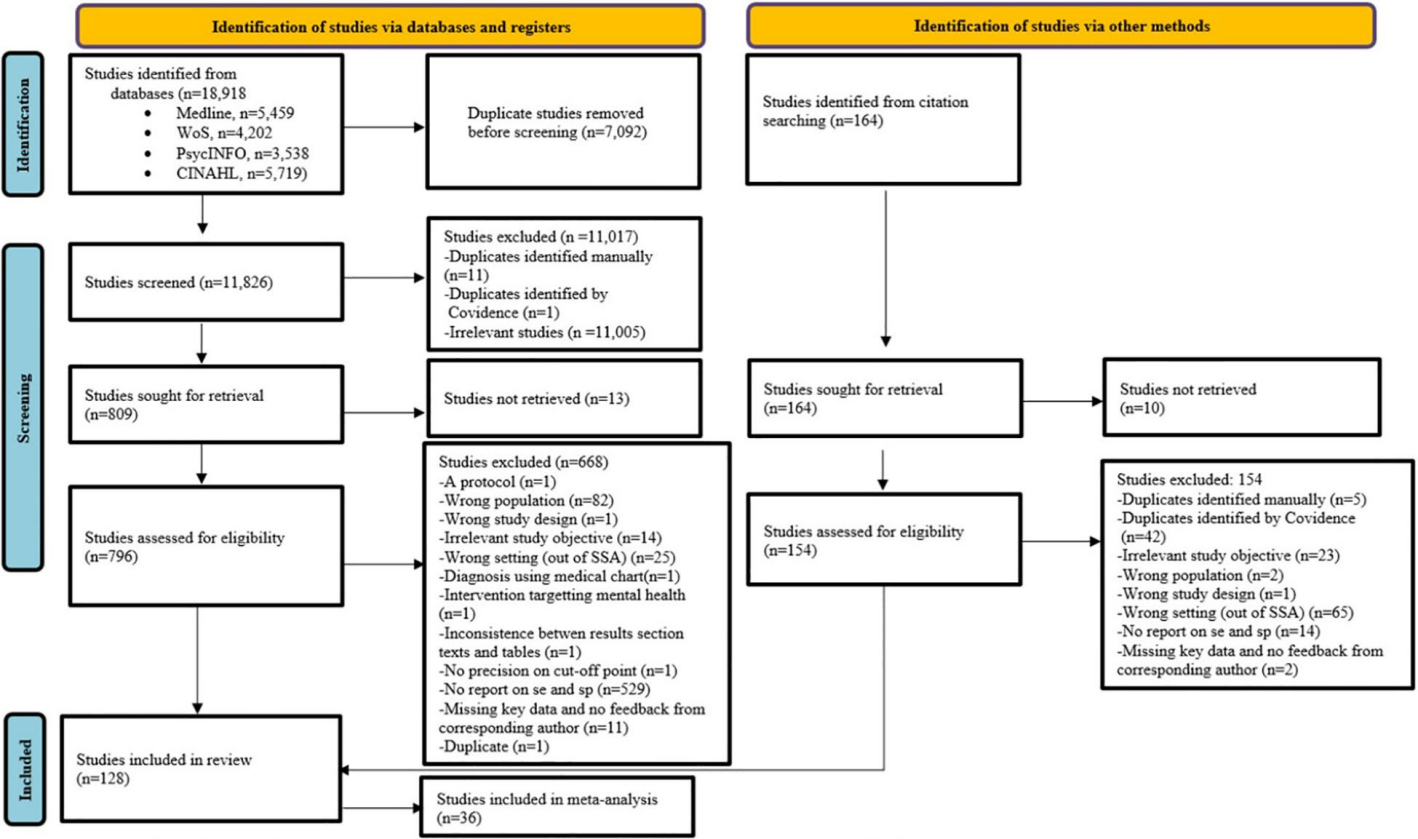
Flow diagram showing study selection process for systematic review and meta-analysis.

Additionally, we conducted a snowballing search by scanning the reference lists of articles included in this review. References that appeared eligible upon reading the title were screened by title and abstract and then uploaded to Covidence for full-text screening. Importantly, other search strategies—such as searching conference abstracts or gray literature—were not utilized in this specific study to maintain the focus on peer-reviewed and published validation data. **Figure 1** illustrates the process taken to select the studies included in the systematic review and meta-analysis.

### 2.3 Selection process

Two reviewers independently screened titles and abstracts and categorized them as “excluded”, “included”, or “potentially eligible”. The full-text screening of articles labeled as “included” or “potentially eligible” was then performed based on the inclusion criteria. As part of the study design, one reviewer (JBN, PhD candidate) screened all records, and the second reviewer was either a research assistant (JN) or research intern (EPN). Disagreement between the reviewers were resolved through a discussion. No disagreement cases required the consultation with a third reviewer. Inter-reviewer agreement was assessed using proportionate agreement, random agreement probability, and Cohen’s Kappa, as automatically computed by Covidence.

### 2.4 Data extraction process and data items

Data were extracted independently by two reviewers using a predefined, standardized, and piloted data extraction form designed by JBN and MOA. The form was reviewed and approved by JBN, MOA, SJ, and ECL. The form captured the following variables: study title, lead author, publication year, country, original tool name, validated tool name, target condition(s), Diagnostic and Statistical Manual of Mental Disorders (DMS) version, study design, target population, sample size, gender, age range, study area (rural/urban), setting (community, primary healthcare center, or hospital), tool administrator, gold standard, language, number of items, number of factors in a validated tool (and name of factors if provided), time required for completion, internal consistency, cutoff point, and diagnostic accuracy measures (sensitivities, specificities, positive predictive value, negative predictive value and area under the curve (AUC) with their respective 95% confidential intervals, if provided). When key data (e.g., age range, country, sensitivity, specificity) were missing, the corresponding authors were contacted via email; if no response was received after one reminder, the concerned article was excluded from the review. JBN extracted data from all of the included articles, and the second reviewer to extract data was either one of the research assistants/interns (JN, EK, AM, VIM, RN, SK, or AM). Discrepancies were resolved through a discussion, and no case required third-party consultation.

### 2.5 Quality assessment

Risk of bias for each included study was assessed using the Quality of Diagnostic Accuracy Studies-2 (QUADAS-2) tool (28). The tool is used to assess the risk of bias for four domains (patient selection, index test, reference standard, and flow and timing) using signaling questions per domain as stated in the paper of Whiting PF and colleagues (28), except one signaling question, under index test domain, that was changed. The revised signaling question of the index test domain was “if a threshold was used, was it prespecified?” This was changed to “was a threshold reported or determined for the validated tool?” We made this change because the initial formulation of the signaling question could not work for the tool developed from scratch; it is also important to note that validation of the tools goes with a specific cutoff score that could differ from the original tool that was validated or adapted. It is worth to note that the applicability of the first three domains of QUADAS-2 tool was assessed. The WHO protocol for the translation and adaptation of instruments into local languages was assessed using one item from a modified Greenhalgh’s 10-item checklist for diagnostic or screening validation studies (15, 25), which was used for quality assessment of the included studies, but the results are not reported here. The item about translation assessment in a modified Greenhalgh’s 10-item checklist is stated as follows: “Was the tool appropriately translated, adapted, and/or designed for the study setting and population? (If using an existing tool, did the authors employ the standardized WHO translation protocol)?” (15, 25).

The assessment of both risk of bias and applicability concern using QUADAS-2 tool was conducted by two reviewers in such a way that the first assessor was JBN for all included articles, and the second assessor was either EK, AM, VIM, RN, or SK. Discrepancies were resolved through a discussion, and no reviewer disagreements required further consultation. The QUADAS-2 tool results were not based on overall quality score; rather, they were evaluated at item level using domain-based judgment approach and were presented using tabular presentation and graphical displays (traffic light bar plots) as suggested by Whiting PF and colleagues (28). The traffic light bar plots were generated in R 4.5.3 version using ggplot2 package, and bars for risk of bias and applicability were displayed horizontally and color-coded as green, yellow, and red for low risk/concern, unclear risk/concern, and high risk/concern, respectively.

### 2.6 Description of result synthesis and data analysis

Individual screening tools or their factors were aggregated either by gender, language, type of a disorder, severity of a disorder, and instance of a disorder and were treated as separated distinct screening tools if their respective cutoff scores and sensitivity and specificity data were reported. We conducted a narrative synthesis of findings to provide a descriptive overview of the included studies, and supplementary appendices were provided for more details. Where applicable, the description of the certainty assessment was described based on the confidential intervals of the estimated diagnostic accuracy measures (sensitivities and specificities). We performed meta-analysis for studies that validated tools which were validated in at least five studies, as recommended in the study of Myung (29); the same requirement was considered for the sub-group meta-analysis. We performed a bivariate random effect of diagnostic accuracy using the hierarchical summary receiver operating characteristic (HSROC) framework using the Reitsma model. We preferred the HSROC style because it is recommended for the meta-analysis of the same screening tool validated in different studies for the same disorders, but with a different cutoff. The latter was expected in the case of the present review.

Sensitivity and false positive rate (FPR) were modeled on the logit scale together, taking into account their within-study correlation. Between-study heterogeneity was also incorporated through random effects for both logit sensitivity and logit FPR, assuming a bivariate normal distribution with an unstructured variance–covariance matrix. The parameters of the model were estimated using restricted maximum likelihood (REML). A Spearman correlation between logit sensitivity and logit FPR from the HSROC model outputs was used to assess the presence of a threshold effect. Additionally, we manually computed (observed) sensitivity and FPR in R and assessed their association using Spearman correlation in order to complement and triangulate the assessment of the presence of a threshold effect. We assessed the between-study heterogeneity using the *I*^2^ statistic from the HSROC meta-analysis, applying Holling’s unadjusted sample size approach. The *I*^2^ statistic indicates the proportion of variability in sensitivity and specificity across studies due to differences in test performance (*I*^2^) rather than chance. Forest plots visualized between-study heterogeneity. These analyses were conducted using the mada *package* from R. Since the inputs of the HSROC model are true positive (TP), false positive (FP), false negative (FN), and true negative (TN), we only performed meta-analysis for the studies that reported either directly the data on TP, FP, FN, and TN or sensitivities, specificities, sample size, and actual positives (or prevalence per reference standard) of the concerned mental disorders allowing to deduce TP, FP, FN, and TN (30). **Box 1** provides the details on how TP, FN, FP, and TP were determined.

Box 1 **Formula used to manually calculate TP, FN, FP, and TP**

A. Required data/information to calculate true positive (TP), false negative (FN), false positive (FP), and true negative (TN) which normally constitute 2 × 2 table

1. Diagnostic accuracy data: Sensitivity (se) and specificity (sp)
2. Data on study participants: Gold-standard-based prevalence or actual positives (AP)
3. Sample size (*N*)

B. Formula

Step 1: Calculation of AP (number of diseased cases according to gold standard) if not provided in the paper

AP = *N* × gold-standard-based prevalence

Step 2: Calculation of actual negatives (AN)

AN = *N* – AP

Step 3: Calculation of TP and FN

TP = se × AP

FN = AP – TP

Step 4: Calculation of TN and FP

TN = sp x AN

FP = AN – TN

The final output is the 2 × 2 table.

**Table:** 

	Gold standard + (diseased)	Gold standard – (not diseased)	Total
Index positive	**TP**	FP	TP + FP
Index negative	FN	**TN**	FN + TN
**Total**	**AP = TP + FN**	**AN = FP + TN**	***N***

Publication bias was assessed using both visual inspection of funnel plot and Deeks’ funnel plot asymmetry test and was performed on studies that validated screening tools validated in a minimum of 10 validation studies, as recommended in the study by Sterne and colleagues (31). A two-sided *p*-value <0.05 was considered statistically significant, where relevant, and all analyses were conducted using R 4.5.3 version.

## 3 Results

### 3.1 Study selection

As illustrated in **Figure 1**, we identified 18,918 articles from databases and included 128 articles in this review. During the title and abstract screening (see **Table 1**), the proportionate agreement (Pa) between JN and JBN was 95.8%, with Cohen’s kappa (*K*) of 0.63, indicating moderate agreement (32). For EVP and JBN, Pa was 80.7%, with weak agreement (*K* = 0.42). In full text screening, Pa between JN and JBN was 98.2%, with almost perfect agreement (*K* = 0.94), and the Pa between EVP and JBN was 97.1%, with strong agreement (*K* = 0.81) (32).

**Table 1:** Agreement between reviewers for title and abstract and full-text screening.

Stage	Reviewer 1	Reviewer 2	Proportionate agreement	Random agreement probability	Cohen’s kappa
Title and abstract screening	JN	JBN	95.8%	88.7%	0.63
	EVP	JBN	80.7%	66.9%	0.42
Full-text screening	JN	JBN	98.2%	70.5%	0.94
	EVP	JBN	97.1%	84.7%	0.81

### 3.2 Study characteristics

We included 128 studies from 21 SSA countries, published between January 1, 2000 and December 31, 2024. These studies validated or adapted 438 unique mental health screening tools, covering 26 broad mental disorders including depression, anxiety, common mental disorders (CMDs), substance use, post-traumatic stress disorder (PTSD), attention deficit/hyperactivity disorder (ADHD), autism spectrum disorder, sleep disorder, suicide, and others (**Supplementary Appendix 2**). Different studies validated specific screening tools for either sub-categories of the 26 broad mental disorders, categories of study population such as health status, gender, and age range, language, country, or classification (by severity or duration) of the targeted condition (**Supplementary Appendix 2**).

A total of 12 tools were validated in a two-country study, resulting in a total of 450 individual validation studies (**Supplementary Appendix 3**). The number of tools validated per country ranged from 1 to 178; the first five countries with the highest number of validated tools were South Africa with 178 tools, followed by Nigeria, which is, in turn, followed by Ethiopia and Zambia with 30 each and Zimbabwe with 28. In the contrast, the last five countries with the lowest number were Mali and Sudan with one tool each, Burkina Faso with two, and Eritrea and Ghana with three each (**Supplementary Appendix 3**).

### 3.3 Quality of studies included in the review

The results from the QUADAS-2 tool-based analysis for both risk of bias and concern for applicability of studies included in this review are presented in **Table 2**.

**Table 2:** Results of assessment of risk of bias and concern of applicability of domains using QUADAS-2-tool.

No.	Study	Risk of bias				Applicability concerns		
		Patient selection	Index test	Reference standard	Flow and timing	Patient selection	Index test	Reference standard
1	Humeniuk 2008 (33)	☹	☹	⚇	☹	☹	☹	☹
2	Seth 2015 (34)	⚇	⚇	⚇	☹	☹	☹	☹
3	Stockton 2023 (35)	☹	☹	☹	☹	☹	☹	☹
4	Miafo 2024 (36)	☹	⚇	⚇	⚇	☹	☹	☹
5	Awadu 2021 (37)	☹	☹	☹	⚇	☹	☹	☹
6	Atuhaire 2023 (38)	☹	☹	☹	☹	☹	☹	☹
7	Stockton 2023 (39)	⚇	☹	☹	☹	☹	☹	☹
8	Vissoci 2023 (40)	⚇	⚇	☹	☹	☹	☹	☹
9	Gebreegziabhere 2024 (41)	☹	⚇	⚇	⚇	☹	☹	☹
10	Kwobah 2024 (42)	☹	⚇	☹	☹	☹	☹	☹
11	Lovero 2024 (43)	☹	⚇	☹	☹	☹	☹	☹
12	Musoni-Rwililiza 2024 (44)	⚇	☹	☹	⚇	☹	☹	☹
13	Nalugya 2024 (45)	☹	⚇	⚇	☹	☹	☹	☹
14	Nwokolo 2024 (46)	☹	⚇	⚇	⚇	☹	☹	☹
15	Stockton 2024 (47)	☹	⚇	☹	☹	☹	☹	☹
16	Stockton 2024 (48)	☹	☹	☹	☹	☹	☹	☹
17	Kaaya 2002 (49)	☹	☹	☹	☹	☹	☹	☹
18	Uwakwe 2003 (50)	⚇	☹	⚇	☹	☹	☹	☹
19	Adewuya 2005 (51)	☹	☹	☹	☹	☹	⚇	⚇
20	Adewuya 2006 (52)	☹	⚇	⚇	☹	☹	☹	☹
21	Adewuya 2006 (53)	☹	☹	☹	☹	⚇	⚇	☹
22	Adewuya 2007 (54)	☹	☹	☹	☹	☹	☹	☹
23	Baggaley 2007 (55)	☹	☹	☹	☹	☹	☹	☹
24	Hanlon 2008 (56)	☹	☹	☹	☹	☹	☹	☹
25	Jordans 2008 (57)	☹	☹	⚇	☹	☹	☹	☹
26	Myer 2008 (58)	⚇	☹	☹	☹	☹	☹	☹
27	Monahan 2009 (59)	⚇	⚇	☹	☹	☹	☹	☹
28	Spies 2009 (60)	⚇	⚇	⚇	⚇	☹	☹	☹
29	Stewart 2008 (61)	☹	☹	☹	☹	☹	☹	☹
30	Weobong 2008 (62)	⚇	☹	☹	☹	☹	☹	☹
31	Chibanda 2010 (63)	☹	☹	☹	⚇	☹	☹	☹
32	Chipimo 2010 (64)	⚇	☹	☹	☹	☹	☹	☹
33	Spies 2010 (65)	⚇	⚇	⚇	⚇	☹	☹	☹
34	Tesfaye 2010 (66)	⚇	⚇	⚇	☹	☹	☹	☹
35	Andersen 2011 (67)	⚇	⚇	⚇	☹	☹	☹	☹
36	Chishinga 2011 (68)	☹	☹	☹	☹	☹	☹	☹
37	Ertl 2011 (69)	☹	☹	☹	☹	☹	☹	☹
38	Lowenthal 2011 (70)	⚇	☹	☹	☹	☹	☹	☹
39	Murray 2011 (71)	⚇	⚇	☹	☹	☹	☹	☹
40	Scholte 2011 (72)	⚇	☹	☹	☹	☹	☹	☹
41	Akena 2012 (73)	☹	☹	☹	☹	☹	☹	☹
42	Betancourt 2012 (74)	⚇	☹	☹	☹	☹	☹	☹
43	Nakimuli-Mpungu 2012 (75)	⚇	☹	☹	☹	☹	☹	☹
44	Tunde-Ayinmode 2012 (76)	⚇	☹	☹	☹	☹	☹	☹
45	Akena 2013 (77)	⚇	☹	☹	☹	☹	☹	☹
46	Gelaye 2013 (78)	⚇	☹	☹	☹	☹	☹	☹
47	Gelaye 2013 (79)	⚇	⚇	☹	☹	☹	☹	☹
48	Mbewe 2013 (80)	☹	☹	☹	☹	☹	☹	☹
49	Rochat 2013 (81)	☹	⚇	⚇	☹	☹	☹	☹
50	Vythilingum 2013 (82)	⚇	⚇	⚇	⚇	☹	☹	⚇
51	Haney 2014 (83)	⚇	⚇	☹	☹	☹	☹	☹
52	Kim 2014 (84)	☹	☹	☹	☹	☹	☹	☹
53	Makanjuola 2014 (85)	☹	⚇	⚇	☹	☹	☹	☹
54	Ng 2014 (86)	☹	☹	☹	☹	☹	☹	☹
55	Tsai, 2014 (87)	☹	⚇	☹	☹	☹	☹	☹
56	Ventevogel 2014 (88)	☹	☹	☹	☹	☹	☹	☹
57	Abdullahi 2015 (89)	☹	☹	☹	☹	☹	☹	☹
58	Bell 2015 (90)	⚇	⚇	☹	☹	☹	☹	☹
59	Bhana 2015 (91)	⚇	⚇	⚇	☹	☹	☹	☹
60	Francis 2015 (92)	☹	⚇	⚇	☹	☹	☹	☹
61	Hanlon 2015 (93)	☹	☹	☹	☹	☹	☹	☹
62	Khalifa 2015 (92)	☹	☹	☹	☹	☹	☹	☹
63	Binagwaho 2016 (94)	⚇	⚇	⚇	⚇	☹	☹	☹
64	Chibanda 2016 (95)	☹	☹	☹	☹	☹	☹	☹
65	Gelaye 2016 (96)	⚇	☹	☹	☹	☹	☹	☹
66	Kane 2016 (97)	☹	⚇	☹	⚇	☹	☹	☹
67	Nakku 2016 (98)	☹	☹	☹	☹	☹	☹	☹
68	Unterhitzenberger 2016 (99)	⚇	⚇	⚇	⚇	☹	☹	☹
69	Valjee 2016 (100)	☹	⚇	⚇	☹	☹	☹	☹
70	vanderWesthuizen 2016 (101)	☹	⚇	⚇	⚇	☹	☹	☹
71	vanderWesthuizen 2016 (102)	☹	⚇	⚇	⚇	☹	☹	☹
72	Aloba 2017 (103)	☹	⚇	⚇	☹	☹	☹	☹
73	Baron 2017 (104)	⚇	☹	☹	☹	☹	☹	☹
74	Marsay 2017 (105)	☹	☹	☹	☹	☹	☹	☹
75	Mellins 2017 (106)	☹	☹	☹	☹	☹	☹	☹
76	Morojele 2017 (107)	☹	⚇	☹	☹	☹	☹	☹
77	Ola 2017 (108)	⚇	☹	☹	☹	☹	☹	☹
78	Akena 2018 (109)	⚇	☹	☹	☹	☹	☹	☹
79	Aloba 2018 (110)	☹	⚇	☹	☹	☹	☹	☹
80	Aloba 2018 (111)	☹	⚇	⚇	⚇	☹	☹	☹
81	Chorwe-Sungani 2018 (112)	☹	☹	☹	⚇	☹	☹	☹
82	Green 2018 (113)	☹	☹	☹	☹	☹	☹	☹
83	Manzar 2018 (114)	⚇	☹	☹	⚇	⚇	⚇	☹
84	Netsereab 2018 (115)	☹	⚇	⚇	⚇	☹	☹	☹
85	Olagundoye 2018 (116)	⚇	☹	☹	⚇	☹	☹	☹
86	Saal 2018 (117)	☹	⚇	⚇	⚇	☹	☹	☹
87	Seun-Fadipe 2018 (118)	☹	⚇	☹	⚇	⚇	⚇	☹
88	vanHeyningen 2018 (119)	☹	⚇	⚇	☹	☹	☹	☹
89	Verhey 2018 (120)	☹	☹	☹	☹	☹	☹	☹
90	Woldetensay 2018 (121)	☹	☹	☹	☹	☹	☹	☹
91	Abrahams 2019 (122)	⚇	⚇	☹	☹	☹	☹	☹
92	Ashaba 2019 (123)	☹	☹	☹	⚇	☹	☹	☹
93	Bhana 2019 (124)	⚇	☹	☹	⚇	☹	☹	☹
94	Ndetei 2019 (125)	☹	☹	☹	☹	☹	☹	☹
95	Phillips 2019 (126)	☹	⚇	⚇	⚇	☹	☹	☹
96	Sangare 2019 (127)	☹	☹	☹	⚇	☹	☹	☹
97	SmithFawzi 2019 (128)	☹	☹	☹	☹	☹	☹	☹
98	vanHeyningen 2019 (129)	☹	⚇	⚇	⚇	☹	☹	☹
99	Bantjes 2020 (130)	☹	⚇	⚇	⚇	☹	☹	☹
100	Cohen 2020 (131)	☹	⚇	☹	⚇	☹	☹	☹
101	Cumbe 2020 (132)	☹	☹	☹	☹	☹	☹	☹
102	Dedeken 2020 (133)	☹	⚇	☹	⚇	☹	☹	☹
103	Degefa 2020 (134)	☹	☹	☹	☹	☹	☹	☹
104	Molebatsi 2020 (135)	☹	☹	☹	☹	☹	☹	☹
105	Saal 2020 (136)	⚇	⚇	⚇	☹	☹	☹	☹
106	Sebera 2020 (137)	☹	⚇	⚇	☹	☹	☹	☹
107	Andersen 2021 (138)	⚇	☹	☹	☹	☹	☹	☹
108	Atkins 2021 (139)	⚇	⚇	⚇	⚇	☹	☹	☹
109	Binagwaho 2021 (140)	⚇	⚇	⚇	⚇	☹	☹	⚇
110	Blanchard 2021 (141)	⚇	⚇	☹	⚇	☹	☹	☹
111	Lovero 2021 (142)	⚇	⚇	⚇	☹	☹	☹	☹
112	Manzar 2021 (143)	☹	⚇	⚇	⚇	⚇	⚇	☹
113	Pence 2012 (144)	☹	☹	☹	⚇	☹	☹	☹
114	Akanni 2022 (145)	☹	⚇	⚇	☹	☹	☹	☹
115	Belus 2022 (146)	⚇	☹	☹	☹	☹	☹	☹
116	Habtamu 2022 (147)	☹	⚇	⚇	⚇	☹	☹	☹
117	Kagee 2022 (148)	⚇	⚇	⚇	⚇	☹	☹	☹
118	Kaiser 2022 (149)	☹	☹	☹	⚇	☹	☹	☹
119	Lovero 2022 (150)	☹	☹	☹	☹	☹	☹	☹
120	Manahan 2022 (151)	☹	☹	☹	⚇	☹	☹	☹
121	Moya 2022 (152)	☹	☹	☹	⚇	⚇	⚇	☹
122	Abas 2023 (153)	☹	☹	☹	⚇	☹	☹	☹
123	Basaraba 2023 (154)	⚇	⚇	⚇	⚇	☹	☹	☹
124	Bothe 2023 (155)	☹	⚇	⚇	⚇	☹	☹	⚇
125	Marlow 2023 (156)	☹	☹	☹	☹	☹	☹	☹
126	Mutiso 2023 (157)	⚇	☹	⚇	⚇	☹	☹	☹
127	Tele 2023 (158)	☹	☹	☹	☹	☹	☹	☹
128	Yusuf 2023 (159)	☹	☹	☹	☹	☹	☹	☹

Quality of studies rated as ☹, low risk; ☹, high risk; and ⚇, unclear.

#### 3.3.1 Results on the assessment of risk of bias

**Figure 2:**
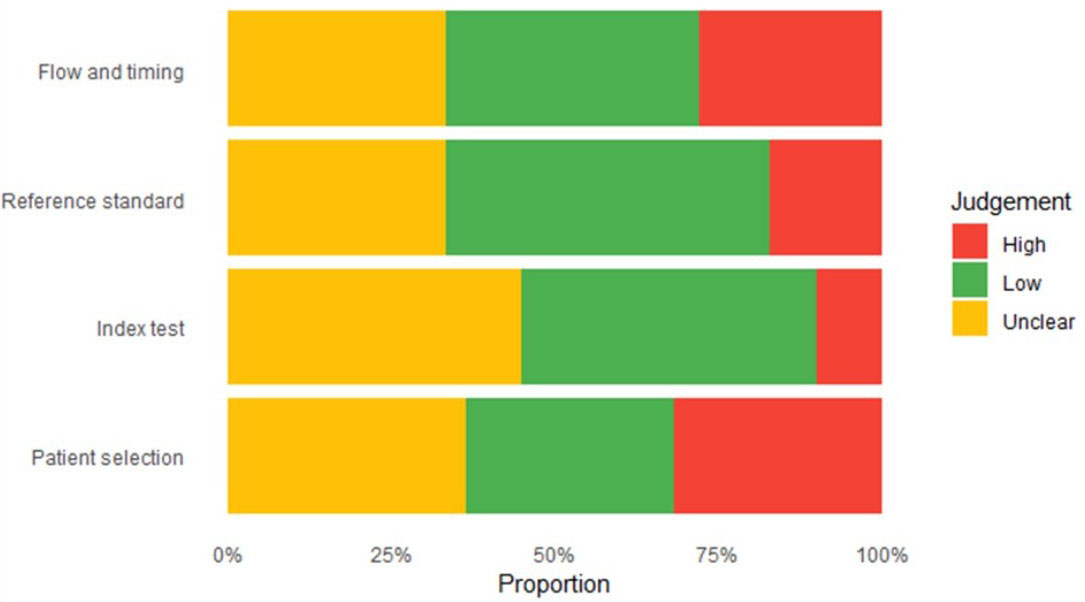
Proportions of studies with low, high, or unclear risk of bias per domain of QUADAS-2 tool.

Of the 128 studies included in the review, 40% (51/128) of them were not rated high risk on any of the domains, 37% (47/128) were rated with high risk of bias on one domain, 20% (26/128) on two domains, 3% (4/128) on three domains, and none on all four domains (**Table 2**). **Figure 2** displays the proportions of studies with low, high, or unclear risk of bias per domain.

The proportions of studies whose risk of bias for patient selection domain rated low, those rated high risk, and those rated unclear were at 32% (41/128), 31% (40/128), and 37% (47/128), respectively (**Table 2**). Among those rated high risk, 38% (15/40) were rated as such because they did not enroll the participants using consecutive or random sampling method and failed to avoid the case–control design. Furthermore, 63% (25/40) were rated high risk because they exclusively failed in only one of the following three criteria: (1) enrolling participants using consecutive or random sampling method, (2) avoiding case–control design, or (3) avoiding inappropriate exclusion of participants. Among the studies rated unclear, 24% (11/46) of them were unclear or did not report concomitantly on the above-mentioned three criteria, 38% (18/47) did not report on the first two criteria only, and 38% (18/47) were exclusively unclear in only one of the above-mentioned three criteria.

The proportion of studies whose risk of bias for index test domain was rated low was 45% (58/128), high risk was 10% (13/128), and unclear was 45% (57/128) (**Table 2**). Among those rated high risk, 61% (8/13) were rated as such because the index test results were interpreted with the knowledge of the results from the reference standard, while 31% (4/13) did not report a threshold. Only 8% (1/13) of those rated high risk concomitantly interpreted the index test results with the knowledge of the results from the reference standard and did not report a threshold. Among those rated unclear, 5% (3/57) of them were rated as such because they were concomitantly unclear on two criteria, namely: (1) whether index test results were interpreted without the knowledge of the results from the reference standard and (2) whether the threshold was reported. The proportion of studies that were exclusively unclear on the first criteria was 88% (50/57). The proportion of the studies that were rated unclear because they were exclusively unclear for the second criteria was 7% (4/57).

The proportion of studies whose risk of bias for the reference standard domain was rated low was 50% (64/128), high risk was 17% (22/128), and unclear was 33% (42/128) (**Table 2**). Among those rated high risk, 77% (17/22) were rated as such because the reference standard was judged to unlikely correctly classify the target condition, while 18% (4/22) were rated high risk because the results from the reference standard were interpreted with the knowledge of the results from the index test. Moreover, 5% (1/22) concomitantly did not meet two criteria, namely: (1) judged to unlikely correctly classify the target condition and (2) judged to have interpreted the results from standard reference with the knowledge of the results from the index test. Among those rated unclear, 86% (36/42) of them were exclusively unclear whether results from reference standard were interpreted with the knowledge of the results from the index test. In addition, 14% (6/42) were classified unclear because they were concomitantly unclear on two criteria, namely: (1) whether the tool correctly classified the target condition and (2) whether the results from standard reference were interpreted without the knowledge of the results from the index test. Moreover, 2% (1/42) were rated unclear because they were exclusively unclear whether the reference standard correctly classified the target condition.

The proportion of studies whose risk of bias for flow and timing domain was rated low was 39% (50/128), high risk was 28% (36/128), and unclear was 33% (42/128) (**Table 2**). Among those rated high risk, majority of them, 61% (22/36), concomitantly did not administer a reference standard to all study participants/patients and did not include all study participants in the analysis. Then, 3% (1/36) did not concomitantly consider an appropriate interval time between index test(s) and reference standard, did not administer a reference standard to all study participants/patients, and did not include all study participants in the analysis. Another 3% (1/36) did not exclusively meet the criteria of providing the same reference standard to all study participants/patients, and 33% (12/36) exclusively did not include all study participants in the analysis. Considering the four criteria [(1)—whether the interval between index test(s) and reference standard was appropriate, (2) whether all patients received a reference standard, (3) whether all patients received the same reference standard, and (4) whether all patients were included in the analysis] to assess flow and timing domain, above three quarters (79%, 33/42) among those rated unclear were exclusively unclear for the first criteria. Furthermore, among those rated unclear, the proportions of those rated unclear on all four criteria, on the first three criteria only, and on the second and the fourth criteria were the same at 7% (3/42) for each category, while the proportion of those rated unclear on the first and the fourth criteria was 5% (2/42).

#### 3.3.2 Applicability of patient selection, index test, and reference standard domains

**Figure 3:**
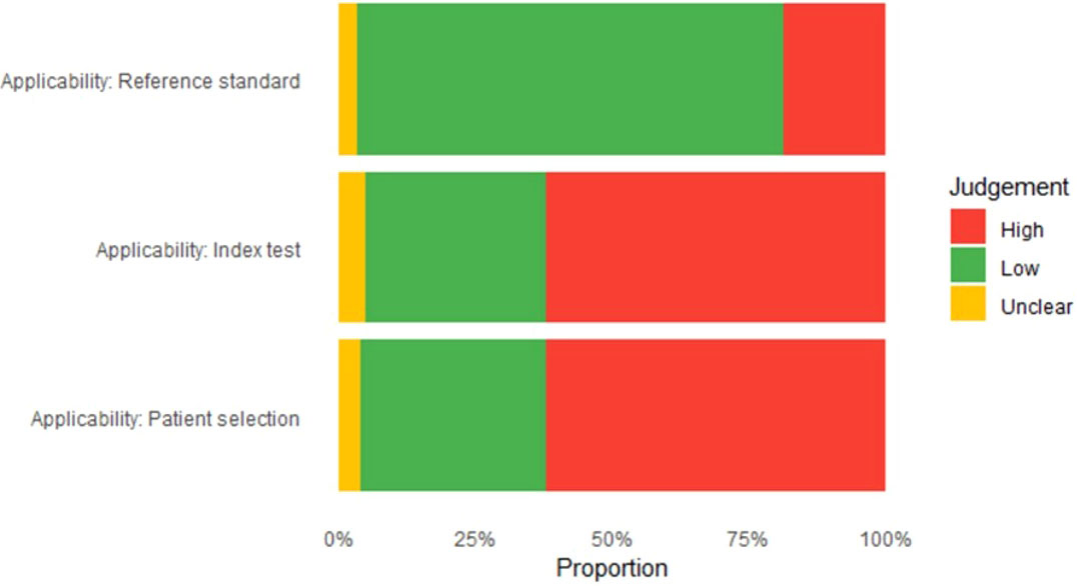
Proportion of studies with low, high, or unclear concerns regarding applicability.

Of the 128 studies included in the review, 27% (34/128) of them were not rated of high concern of applicability on any of the domains, 19% (24/128) were rated of high concern of applicability on one domain, 46% (59/128) on two domains, and 9% (11/128) on three domains (**Table 2**). **Figure 3** displays the proportions of studies with low, high, or unclear concern regarding applicability for the three domains: patient selection, index test, and reference standard.

The proportions of studies whose concern about applicability to the patient selection and index test domains were rated the same across the three categories were as follows: 34% (44/128), 61% (79/128), and 4% (5/128) for row, high, and unclear, respectively (**Table 2**). Moreover, 100% of all studies whose concern about applicability to the patient selection and index test domains rated high were rated as such because the overlap between age range of the study participants of the concerned studies and the age range mainly considered in this review is very minimal, 18 years (or 17 years) and above versus 12–18 years. In addition, 100% of studies whose concern about applicability to patient selection and index test domains rated unclear were rated as such because they did not report the age range of the study participants; rather, they reported the mean age range (standard deviation). The latter served to decide whether the age of some participants of a concerned study is within the age bracket 12–18 years, which is mainly targeted by the present review.

The proportion of studies whose concern about applicability to reference standard was rated low was 78% (100/128), high was 19% (24/128), and unclear was 3% (4/128) (**Table 2**). Among those rated high concern of applicability, 67% (16/24) used reference standards that are not clinically and research-wise recognized as reference standard, 13% (3/24) used tools that relied on expert opinion, which is subjected to subjectivity as experts may judge differently. Furthermore, 8% (2/24) used tools or questionnaires that required the interviewees (study participants/patients) to classify themselves and their status, and this is subjected to social desirability. Finally, the proportions of studies that did not used a gold/reference standard at all, used a gold standard but did not clarify/provide its name, and used a full or long version to validate a short form were the same at 4% (1/24) per case.

### 3.4 Results of individual studies

Comprehensive information for all the 128 studies included in this review is available in**Supplementary Appendix 2**. This includes author, title, country, name of the validated or adapted tool(s), targeted condition, characteristics of the study population, participant age range, sample size, tool language, internal consistency, cutoff points, sensitivity, specificity, PPV, NPV, and AUC. However, some studies did not report all psychometric properties of the validated or adapted tools. As can be deduced from **Supplementary Appendix 2**, most of the studies did not comply with the reporting standards for scale validation (15, 160). In this review, cutoff point was not reported for 11% (50/438) of the tools. Although all validated tools reported sensitivity (se) and specificity (sp), 71% (311/438) did not report their 95% CI. Half, 50% (221/438), did not report PPV, and 55% (240/438) did not report NPV. While 82% (357/438) reported AUC, 34% (123/357) of those with AUC did not report their 95% CI.

Of the 128 studies, 37% (47/128) were considered of adequate age group, whereas 63% (81/128) were not, considering the operationalization of adequate age group in the methods section (**Supplementary Appendix 2**). Among studies considered adequate concerning age range, 87% (41/47) reported the exact age range, with lower bounds ranging from 4 to 16 years and upper bounds ranging from 13 to 87 years. Then, 13% (6/47) of the studies considered adequate in regard to age range did not report the exact age ranges; they provided only a lower bound (15 or 16 years) with an open-ended upper bound. It is important to note that only a single study (84) considered the study population aged 12–18 years, which is the exact age range mainly targeted by this review. We also found only 12 studies whose study participants were within the age range of 10–20 years and included those in age range of 10–17 years (74, 86), 10–19 years (156, 158), 12–17 years (149), 12–19 years (150), 12–20 years (36, 100), 13–17 years (97, 108, 123), and 13–18 years (54), and they were considered comparable to the exact age range mainly targeted in this review because the differences in their lower and upper bounds were minimal.

Among the studies considered inadequate concerning age range, 36% (29/81) reported the exact age range, with a lower bound of 18 for 28 studies and 17 for one study (55) and upper bounds ranging from 24 to 99 years. Then, 64% (52/81) of the studies considered inadequate about age range did not report the exact age ranges; they provided either a lower bound (19) with an open-ended upper bound or mean age (± SD).

We found that nearly one-third of the articles included in the review, 30% (38/128), did not appropriately translate, adapt, and/or design for the study setting and population, which also indicates that, for the studies which adapted existing tools, the authors did not employ standardized WHO translation protocol.

### 3.5 Results of synthesis

#### 3.5.1 Description of mental health screening tools and validation studies

We found that, out of 128 studies, only 36 (27%) met the requirement to be included in the meta-analysis because they validated tools that were validated in at least five studies, as recommended in the study of Myung (29) (see **Supplementary Appendix 4**).

The included studies validated one or more of four mental health screening tools: either Edinburg Postnatal Depression Scale (EPDS), Patient Health Questionnaire-9 (PHQ-9)/two-step Patient Health Questionnaire-9 (PHQ-2/9),/two-item Patient Health Questionnaire (PHQ-2), or Kessler Psychological Distress Scale (K-10). These tools together targeted one broad mental health condition—depression.

#### 3.5.2 Results from meta-analysis per tool

##### 3.5.2.1 Edinburgh Postnatal Depression Scale

Edinburg Postnatal Depression Scale (EPDS) was validated 15 times in 13 studies across nine SSA countries—Uganda (38), Zimbabwe (63), Sudan (161), Malawi (112, 152), Kenya (113, 157), South Africa (81, 129), Nigeria (50, 53), Ghana (62), and Cameroon (36)—and involved a total of 4,262 participants (sample sizes varying from 86 to 1,633) aged 12 years and above. These studies focused on prenatal and postnatal women, with one study (63) in Zimbabwe (63) specifically examining HIV-infected and uninfected postpartum women (63). The study of Green et al. (113) in Kenya (113) assessed EPDS using both 1- and 2-week recall periods (113), which were treated as separate tools. The study of Adewuya et al. (53) in Nigeria (53) validated EPDS for both major depressive disorder and minor depressive disorder. Sensitivity across studies ranged from 0.60 to 0.89 and specificity from 0.36 to 0.92, with cutoff scores between 9 and 16 (**Table 3**).

**Table 3:** Outputs from the HSROC meta-analysis model for EPDS considering for both prenatal and postpartum depression.

Study author, country	Sample size (*N*)	Cutoff	Se (95% CI)	Sp (95% CI)	TP	FN	FP	TN	Key outputs of HSROC model
Miofa 2024, Cameroon	1,633	11	0.92 (0.91–0.94)	0.53 (0.51–0.56)	920	74	299	340	1. Pooled se (95% CI): 0.81 (0.75–0.86) 2. Pooled FPR (95% CI): 0.17 (0.12–0.24) 3. Pooled sp (95% CI)^a^: 0.83 (0.76–0.88) 4. AUC: 0.88 5. Partial AUC (restricted to FPR): 0.82 6. Between-study std. dev. for the logit-transformed se: 0.52 7. Between-study std. dev. for the logit-transformed FPR: 0.82 8. rho between the logit-transformed se and the logit-transformed FPR: 0.20 9. *I*^2^ estimate by Holling sample size unadjusted approaches: 34.8%–66%
Atuhaire 2023, Uganda	278	10	0.87 (0.77–0.94)	0.92 (0.88–0.95)	66	10	16	186	
Uwakwe 2003, Nigeria	225	9	0.75	0.97	18	6	6	195	
Adewuya 2006a, Nigeria	86	10	0.867	0.867	13	2	6	65	
Adewuya 2006b, Nigeria	86	12	1	0.961	9	0	3	74	
Weobong 2008, Ghana	160	10/11	0.78	0.73	14	4	38	104	
Chibanda 2010, Zimbabwe	210	12	0.88	0.87	56	8	19	127	
Rochat 2013, South Africa	109	≥ 13	0.69	0.78	35	16	13	45	
Khalifa 2015, Sudan	238	10	0.89	0.82	19	2	39	177	
Chorwe-Sungani 2018, Malawi	97	10	0.68 (0.47–0.85)	0.88 (0.78–0.94)	17	8	9	63	
Green 2018a, Kenya	193	16	0.70	0.72	7	3	51	132	
Green 2018b, Kenya	193	13	0.60	0.73	6	4	49	134	
vanHeyningen 2019, South Africa	376	13	0.75	0.78	112	38	50	176	
Moya 2022, Malawi	115	10	0.77	0.67	10	3	34	68	
Mutiso 2022, Kenya	544	11	0.81 (0.71–0.89)	0.83 (0.79–0.86)	64	15	81	384	

**Figure 4:**
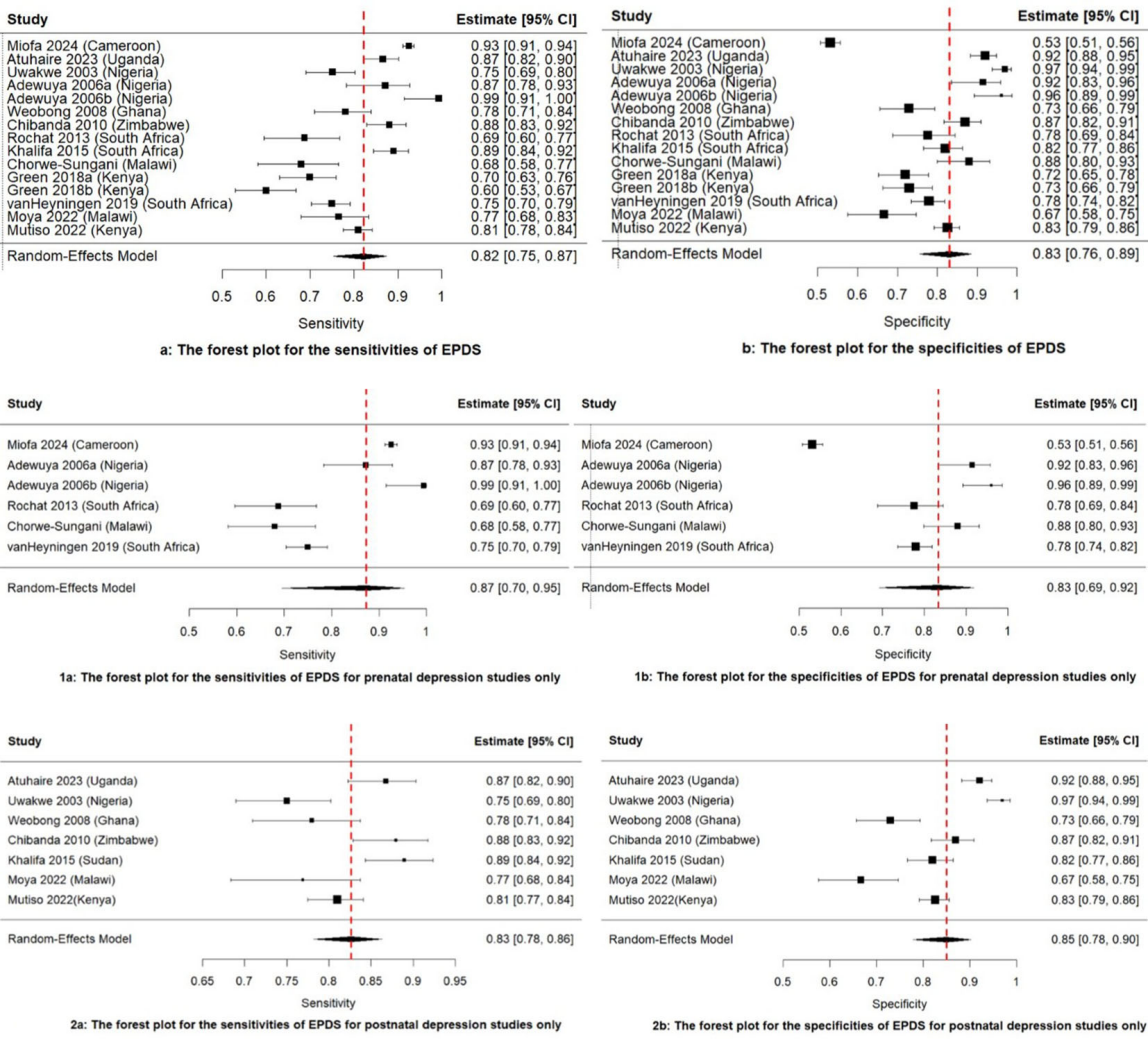
**(a)** The forest plot for the sensitivities of EPDS. **(b)** The forest plot for the specificities of EPDS.

The HSROC meta-analysis (**Table 3**) indicated pooled sensitivity and specificity of 0.81 (0.75–0.86) and 0.83 (0.76–0.88), respectively. The 95% CI of both estimates (sensitivity and specificity) are relatively moderate, reflecting a moderate uncertainty concerning the pooled estimates across 15 validations. Between-study standard deviation (SD) for the logit-transformed sensitivity and between-study SD for the logit-transformed FPR are 0.52 and 0.82, respectively, suggesting that specificity varies more substantially than specificity across studies. Heterogeneity (*I*^2^) was between 34.8% and 66%, suggesting that much of the observed variability in sensitivity and specificity was due to heterogeneity between studies rather than chance or differences in sample sizes. The forest plot (**Figures 4a, b**) visualized this notable heterogeneity. This is especially noted in both sensitivity and specificity estimates, with compressed intervals (dotted lines) caused by extreme values in some studies.

We also found that there is a weak positive correlation (0.02) between logit-transformed sensitivity and logit-transformed false positive rate (FPR) (FPR = 1 - specificity), which indicates minimal threshold effect in heterogeneity across studies. Along the same lines, Spearman’s rank correlation between sensitivity and FPR was -0.24 (*p* = 0.389), indicating no statistically significant association between sensitivity and FPR, which suggests that a threshold effect is unlikely to be a main contributor to heterogeneity across studies.

We further performed a sub-group meta-analysis for EPDS measuring prenatal and postnatal depression separately.

**Table 1.1 in Supplementary Appendix 5** presents the outputs of the subgroup meta-analysis for prenatal depression based on six validations from five studies across four SSA countries—Malawi (112), South Africa (81, 129), Nigeria (53), and Cameroon (36)—which collectively involved 2,301 pregnant women. The cutoff scores varied between 10 and 13. Compared to the overall pooled sensitivity and pooled specificity from the HSROC meta-analysis, the corresponding measures were lower in the studies of the prenatal group with pooled sensitivity and specificity of 0.81 (0.75–0.86) vs. 0.74 (0.059–0.86) and 0.83 (0.76–0.88) vs. 0.82 (0.69–0.90), respectively. Moreover, the substantial overlap in 95% CI for both estimates is obvious and indicates that the prenatal group did not meaningfully influence the EPDS performance, but uncertainty is high in this group.

We also found that the between-study SD for the logit-transformed sensitivity substantially increased from 0.52 to 0.82, while the logit-transformed FPR slightly decreased from 0.82 to 0.80. The *I*^2^ values slightly widened from 34.8%–66% to 27.2%–76.6%, suggesting more heterogeneity within the prenatal subgroup, an unexpected output of the subgroup analysis. The forest plots (**Figures 4.1a, b**) visualized the expression of more heterogeneity within the prenatal subgroup. As found in the overall HSROC meta-analysis model, Spearman’s rank correlation between sensitivity and FPR in the prenatal group was not statistically significant (rho = -0.257, *p*-value = 0.658), suggesting that a threshold effect is unlikely to be a main contributor to heterogeneity across prenatal depression studies.

**Table 1.2 in Supplementary Appendix 6** summarizes the results of the sub-group meta-analysis of seven validations of EPDS for postpartum depression in seven different studies across seven SSA countries—Uganda (38), Nigeria (50), Ghana (62), Zimbabwe (63), Sudan (161), Malawi (152), and Kenya (157)—comprising 1,770 postpartum women. The cutoff scores varied between 9 and 12. We found that the pooled sensitivity and specificity were slightly higher compared to the overall HSROC meta-analysis model and were 0.83 vs. 0.81 and 0.85 vs. 0.83, respectively. We also noticed a substantial decrease in the between-study SD for logit-transformed sensitivity, from 0.52 to 0.03, suggesting greater consistency vis-à-vis sensitivity within this sub-group. Regarding the between-study SD for the logit-transformed FPR, we noticed a marginal increase, from 0.79 to 0.82, indicating inconstancy in specificity within the same sub-group. In other words, sensitivity becomes almost homogeneous, while specificity keeps heterogeneity across postnatal depression studies. The *I*^2^ statistic values also narrowed from 34.8%–66% to 28.3%–36.9%, which also suggests a reduction of overall heterogeneity in the postnatal sub-group. The forest plots (**Figures 4.2a, b**) visualized the expression of more homogeneity within the postnatal subgroup. Spearman’s rank correlation between sensitivity and FPR was zero (*p*-value = 1), indicating no evidence of a threshold effect across the postnatal depression studies.

It is notable that the study by Green et al. (2018) (113), which was included twice in the overall HSROC meta-analysis due to its assessment of two EDPS versions (EDPS-1 week and EPDS-2 weeks) was excluded from the sub-group meta-analysis. This is because the study sample included both pregnant and postpartum women, and no comparable studies targeting the same mixed population was available to enable a valid subgroup analysis. It is important to note that EPDS has also been validated for common mental disorders (CDM), but meta-analyses were not conducted because of the insufficient number of studies.

##### 3.5.2.2 Patient Health Questionnaire

This review included two formats of the Patient Health Questionnaire (PHQ-9): a one-step version (PHQ-9) and a two-step version (PHQ-2/9), where only those scoring above zero on the first two items (PHQ-2) proceed to complete the remaining seven items (48).

###### 3.5.2.2.1 Considering any format of PHQ-9 and all population categories

A total of 27 validations across 20 studies (*N* = 8,626; sample size range: 153–1885; study participants overall age range: 10 years and above) validated the PHQ-9 or PHQ-2/9 for depression screening across 12 SSA countries—South Africa (48, 91, 156), Ethiopia (78, 93, 121, 134), Nigeria (52), Ghana (62), Uganda (77, 98), Zimbabwe (95), Kenya (113, 158), Tanzania (128), Mozambique (132) (150), Botswana (135), Rwanda (137), and Cameroon (144)—and the study participants included patients (and their companions), prenatal/postnatal women, adolescents, students, and individuals with chronic illnesses such as HIV, hypertension, diabetes and epilepsy. The reported sensitivity ranged from 0.27 to 0.95 and specificity from 0.59 to 0.99, with cutoffs varying from 4 to 15 (**Table 4**).

**Table 4:** Outputs from the HSROC meta-analysis model for PHQ-9/PHQ-2/9 irrespective of administration steps for all categories of populations (patients and pregnant women and their respective companions and healthy population).

Study author, country	Sample size (*N*)	Cutoff	Se (95% CI)	Sp (95% CI)	TP	FN	FP	TN	Key outputs of the HSROC model
Gelaye 2013, Ethiopia	926	10	0.86 (0.78–0.92)	0.67 (0.61–0.73)	40	6	290	590	1. Pooled se (95% CI): 0.79 (0.73–0.84) 2. Pooled FPR (95% CI): 0.18 (0.13–0.24) 3. Pooled sp (95% CI)^a^: 0.82 (0.76– 0.87) 4. AUC: 0.87 5. Partial AUC (restricted to FPR): 0.76 6. Between study std. dev. for the logit-transformed se: 0.77 7. Between study std. dev. for the logit-transformed FPR: 0.97 8. rho between the logit-transformed se and the logit-transformed sp: 0.55 9. *I*^2^ estimates by Holling sample size unadjusted approaches: 53.5%–85.4%
Stockton 2024a, South Africa	1,885	5	0.84 (0.80–0.88)	0.67 (0.65–0.70)	354	66	480	985	
Stockton 2024b, South Africa	1,885	10	0.49 (0.44–0.54)	0.90 (0.89–0.92)	198	207	144	1,336	
Adewuya 2006a, Nigeria	512	5	0.90	0.99	23	3	5	481	
Adewuya 2006b, Nigeria	512	10	0.85	0.99	11	2	3	496	
Weobong 2008, Ghana	160	4/5	0.94	0.75	17	1	36	107	
Akena 2013, Uganda	368	10	0.92	0.81	59	5	58	246	
Bhana 2015, South Africa	676	9	0.49	0.94	38	39	38	561	
Hanlon 2015, Ethiopia	306	5	0.83	0.75	15	3	73	215	
Chibanda 2016, Zimbabwe	264	11	0.85 (0.78–0.90)	0.69 (0.59–0.77)	44	8	66	146	
Nakku 2016, Uganda	153	5	0.674	0.781	57	27	15	54	
Green 2018, Kenya	193	15	0.70	0.74	7	3	48	135	
Woldetensay 2018, Ethiopia	216	8	0.81	0.79	23	5	39	149	
Smith-Fawzi 2019, Tanzania	174	9	0.78 (0.52–0.94)	0.87 (0.80–0.92)	14	4	20	136	
Cumbe 2020, Mozambique	502	9	0.47	0.94	20	23	30	429	
Degefa 2020, 2020	163	4	0.88	0.78	22	3	30	108	
Molebatsi 2020, Botswana	257	9	0.72 (0.63–0.81)	0.76 (0.69–0.83)	76	29	36	116	
Sebera 2020a, Rwanda	434	5	0.72	0.70	89	34	95	216	
Sebera 2020b, Rwanda	434	5	0.89	0.59	63	8	148	215	
Sebera 2020c, Rwanda	434	7	0.94	0.65	59	4	129	242	
Stockton 2024c, South Africa	1,885	5	0.84 (0.80–0.87)	0.70 (0.67–0.72)	351	69	443	1,022	
Stockton 2024d, South Africa	1,885	10	0.49 (0.44–0.54)	0.91 (0.89–0.92)	351	69	134	1,331	
Pence 2012, Cameroon	400	10	0.27 (0.06–0.61)	0.94 (0.91–0.96)	3	8	23	366	
Lovero 2022, Mozambique	485	8	0.78	0.80	32	9	89	355	
Marlow 2022, South Africa	302	10	0.91	0.76	21	2	67	212	
Tele 2023a, Kenya	250	9	0.95	0.73	18	1	62	169	
Tele 2023b, Kenya	250	9	0.89	0.70	17	2	69	162	

^a^ Deduced from FPR: 1-FPR.

**Figure 5:**
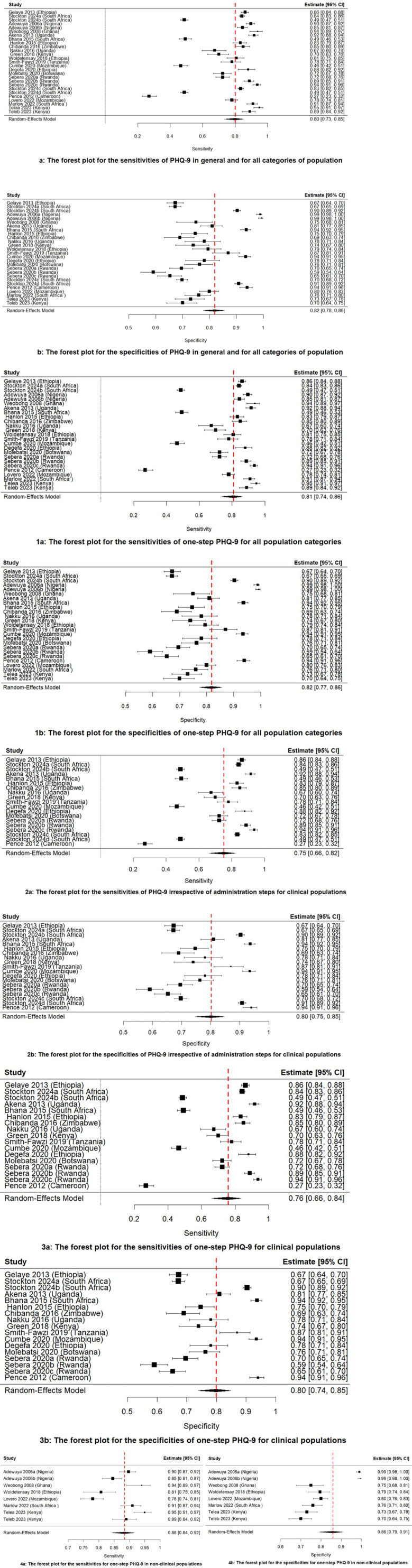
**(a)** The forest plot for the sensitivities of PHQ-9 in general and for all categories of population. **(b)** The forest plot for the specificities of PHQ-9 in general and for all categories of population.

The HSROC meta-analysis model (**Table 4**) produced a pooled sensitivity of 0.79 (0.73–0.84) and a pooled specificity of 0.82 (0.76–0.87). The outputs of the HSROC model for 27 validation studies of the PHQ-9/PHQ-2/9 (**Table 4**) revealed variability across studies. The between-study SD for the logit-transformed sensitivity and between-study SD for the logit-transformed FPR were 0.77 and 0.97, respectively, indicating heterogeneity in test accuracy. The correlation between the logit-transformed sensitivity and the logit-transformed FPR was 0.55, which is positively moderate (162). This indicates that sensitivity and specificity have a tendency to increase or decrease together. We found a statistically significant moderate correlation between sensitivity and FPR (Spearman’s correlation = 0.419, *p*-value = 0.029), indicating a threshold effect across the validation studies of PHQ-9 and/or PHQ-2/9. The *I*^2^ values ranged from 53.5% to 85.4% (**Table 4**), indicating the proportion of total variability due to between-study heterogeneity that was high. The forest plots visually demonstrated this, showing a wide variation in both sensitivity and specificity across studies (**Figures 5a, b**). Notably, the pooled sensitivity computed from the HSROC meta-analysis model slightly differed from those generated from the forest plot, 0.79 vs. 0.80, while the pooled specificity (0.82) did not change. This variation is likely due to differences in computing the pooled estimates. In the HSROC model, the inputs are raw counts of TP, FN, FP, and TN, whereas in the forest plot the inputs are decimal numbers (sensitivities of individual studies) and whole numbers (sample size).

In the sub-group meta-analyses, we considered analysis per type of PHQ (PHQ-9 or PHQ-2/9) and category of populations to which validation studies were conducted. Each sub-group meta-analysis is described below.

###### 3.5.2.2.2 One-step PHQ-9 across all population categories

First, we conducted a sub-group meta-analysis on one-step PHQ-9 validated to all population categories (patients/their respective companions, prenatal/postnatal women, adolescents, and students) in 25 validations from the same number of studies across the same countries as the overall HSROC meta-analysis model involving 8,626 participants (**Table 2.1 in Supplementary Appendix 5** presents the details). The cutoff scores varied between 4 and 15. Compared to the overall HSROC model that included all types of PHQ-9 and all population categories, both pooled estimates (sensitivity and specificity) remained unchanged. This suggests that the two-step PHQ-9 (PHQ-2/9) studies did not influence the pooled estimates in the overall HSROC meta-analysis model.

The between-study SD for the logit transformed sensitivity and for the logit-transformed FPR marginally increased, from 0.77 to 0.81 and 0.97 to 0.99, respectively. Compared to the overall HSROC meta-analysis model, *I*^2^ was slightly changed from 53.4%–85.4% to 43.2%–77.6%, suggesting that there is no notable difference between the overall HSROC model and the model considering one-step PHQ-9 validation for all population categories. The forest plots of both sensitivity and specificity across the validation studies of one-step PHQ-9 for all population categories do not differ from those of the overall model (**Figures 5.1a, b**). As the overall model, we found a statistically significant moderate correlation between sensitivity and FPR (Spearman’s correlation = 0.440, *p*-value = 0.028), suggesting a threshold effect across the validation studies of one-step PHQ-9. It should be noted that we did not perform a sub-group meta-analysis for validations of the two-step PHQ-9 because of the insufficient number of studies.

###### 3.5.2.2.3 PHQ-9 in general in clinical populations (and their companions)

The second sub-group analysis focused on 19 validations in 14 studies (*N* = 6,701) that validated both PHQ-9 and PHQ-2/9 among study participants in clinics (patients and their companions, and prenatal/postnatal women), with cutoff scores varying between 4 and 15 as presented in the **Table 2.2 in Supplementary Appendix 5**. Six studies (with eight validations) conducted in community settings— Adewuya 2006a/b study (52), which involved university students; Weobong 2008 (62) study, which involved postnatal women; Woldetensay 2018 (121) study, which involved pregnant women from the community; the Levero 2022 (150), Tele 2023a/b (158), and Marlow 2022 (156) studies, which involved adolescents—were excluded.

In this sub-group meta-analysis, both pooled sensitivity and specificity slightly decreased compared to the overall HSROC meta-analysis model, 0.79 vs. 0.72 and 0.82 vs. 0.80, respectively. This minimal change suggests an insignificant effect of the variability in diagnostic test performance among validation studies using PHQ-9/PHQ-2/9 in non-clinical populations on the overall model.

The between-study SD for the logit-transformed sensitivity slightly increased from 0.76 to 0.81, while the between-study deviation for the logit-transformed FPR substantially decreased from 0.96 to 0.74. Compared to the overall HSROC meta-analysis model, the *I*^2^ values decreased in both lower and upper bounds from 53.4%–85.4% to 43.3%–79.2%. Despite the decrease in both lower and upper bounds of *I*^2^, the increase in the range of *I*^2^ indicates a more uncertain estimation of heterogeneity in this validation studies of PHQ-9 irrespective of administration steps among the clinical population. This variability was also expressed in the forest plots (**Figures 5.2a, b**). We also found a statistically significant strong correlation between sensitivity and FPR (Spearman’s correlation = 0.78, *p*-value <0.002) in the validation studies of PHQ-9 in clinical populations, suggesting a threshold effect across these studies.

###### 3.5.2.2.4 One-step PHQ-9 in clinical populations (and their companions)

The third sub-group meta-analysis considered 17 validations from 14 studies (*N* = 6,701), validating the one-step PHQ-9 among clinical populations only (patients and pregnant women and their companions), with cutoff scores varying between 4 and 15 as indicated in **Table 2.3 in Supplementary Appendix 5**. For this model, the excluded studies are the same as those excluded for the model of PHQ-9, in general, for clinical populations (and their companions) plus two validations—Stockton 2024c/d studies (48) that validated the two-step PHQ-9 (PHQ-2/9).

Compared to the overall HSROC, both the pooled sensitivity and specificity marginally decreased from 0.79 to 0.72 and from 0.82 to 0.80, respectively. This suggests that the excluded studies do not quietly differ from studies in the overall model to detect both depression cases and non-cases.

The between-study SD for logit-transformed sensitivity slightly increased from 0.77 to 0.81, while the between-study SD for the logit-transformed FPR decreased from 0.97 to 0.74. These suggest that the between-study variability in sensitivity for one-step PHQ-9 among patients and their companions is higher than the between-study variability in sensitivity for PHQ-9, irrespective of its administration steps, considering all population categories. This variability was also expressed in the forest plots (**Figures 5.3a,b**). This was unexpected because the sub-group analysis should reduce heterogeneity.

The *I*^2^ values marginally changed from 53.4%–85.4% to 47.1%–80.1%; this indicates a more precise but still considerable heterogeneity, and the decrease in both lower and upper limits of *I*² suggests less heterogeneity among validation studies of one-step PHQ-9 validated to clinical populations (and their companions). We also found a statistically significant strong correlation between sensitivity and FPR (Spearman’s correlation = 0.74, *p*-value <0.001) in the validation studies of PHQ-9 in clinical populations, suggesting a threshold effect across these studies.

###### 3.5.2.2.5 One-step PHQ-9 in non-clinical populations (adolescents and students)

The final sub-group meta-analysis was conducted on one-step PHQ-9 that included eight validations from six studies (*N* = 1,925) across six SSA countries—Nigeria (53), Ethiopia (121), Kenya (158), Ghana (62), Mozambique (150), and South Africa (156)—which were conducted among adolescents or students (**Table 2.4 in Supplementary Appendix 5**). The cutoff scores varied between 4/5 and 10. Compared to the overall HSROC meta-analysis model, both pooled sensitivity and specificity increased significantly, from 0.78 to 0.85 and from 0.83 to 0.88, respectively. This suggests that PHQ-9 performs exceptionally well in detecting and ruling out depression among non-clinical populations (adolescents and university students). The between-study SD for logit-transformed sensitivity substantially decreased from 0.76 to 0.03, while the between-study SD for the logit-transformed FPR increased from 0.96 to 1.62. The change of the two between-study SD in opposite directions indicate that the variability in sensitivity was largely reduced, while the variability in specificity remained high in the HSROC model of non-clinical populations. This variability was also expressed in the forest plots (**Figures 5.4a, b**), where we found a moderate positive correlation between sensitivity and FPR (Spearman’s correlation = 0.67), although this association was not statistically significant (*p*-value = 0.083). This suggests a lack of evidence of a threshold effect in this model. The *I*^2^ values extremely decreased from 53.4%–85.4% to the range of 26.5%–36.6%, reflecting a much lower heterogeneity as expected in the subgroup analysis. Due to the insufficient number of studies, we did not perform publication bias assessment for the validation studies of PHQ-9 in non-clinical populations. It is also important to note that no separate sub-group analysis was conducted for PHQ-2/9, as it was validated in only one study conducted in South Africa (Stockton 2024) among patients or companions in clinics.

##### 3.5.2.3 PHQ-2 for depression

PHQ-2 was validated for depression screening in eight validations from eight different studies across five SSA countries—South Africa (48, 91, 124), Ethiopia (93, 96), Zimbabwe (95), Mozambique (132), and Uganda (98) (*N* = 5,363; sample sizes ranged from 153 to 1,885). Sensitivity and specificity varied widely (0.50–0.94 and 0.40–0.84, respectively), with cutoff scores ranging from >0 to 3 (**Table 5**). The study participants were patients (and their companions) at healthcare facilities (**Supplementary Appendix 2**).

**Table 5:** Outputs from the HSROC meta-analysis model for PHQ-2 for depression.

Study author, country	Sample size (*N*)	Cut-off	Se (95% CI)	Sp (95% CI)	TP	FN	FP	TN	Key outputs of HSROC model 1
Stockton 2024, South Africa	1,885	>0	0.94 (0.92–0.96)	0.43(0.41–0.46)	396	24	830	635	1. Pooled se (95% CI): 0.78 (0.65–0.87) 2. Pooled FPR (95% CI): 0.36 (0.26–0.48) 3. Pooled sp (95% CI)^a^: 0.64 (0.52–0.74) 4. AUC: 0.76 5. Partial AUC (restricted to FPR): 0.76 6. Between study std. dev. for the logit-transformed se: 0.89 7. Between study std. dev. for the logit-transformed FPR: 0.69 8. rho between the logit-transformed se and the logit-transformed sp: 0.92 9. *I*^2^ estimate: 65.3%–76.4%
Bhana 2015, South Africa	676	2	0.60	0.84	47	31	96	503	
Hanlon 2015, Ethiopia	306	1	0.83	0.61	15	3	113	175	
Chibanda 2016, Zimbabwe	264	2	0.91 (0.86–0.95)	0.40 (0.31–0.50)	47	5	127	85	
Gelaye 2016, Ethiopia	363	3	0.74 (0.59–0.86)	0.60 (0.54–0.65)	34	12	128	189	
Nakku 2016, Uganda	153	1	0.66	0.59	56	28	28	41	
Bhana 2019, South Africa	1,214	3	0.58	0.77	33	24	262	895	
Cumbe 2020, Mozambique	502	2	0.74	0.72	32	11	130	329	

^a^ Deduced from FPR: 1-FPR.

**Figure 6:**
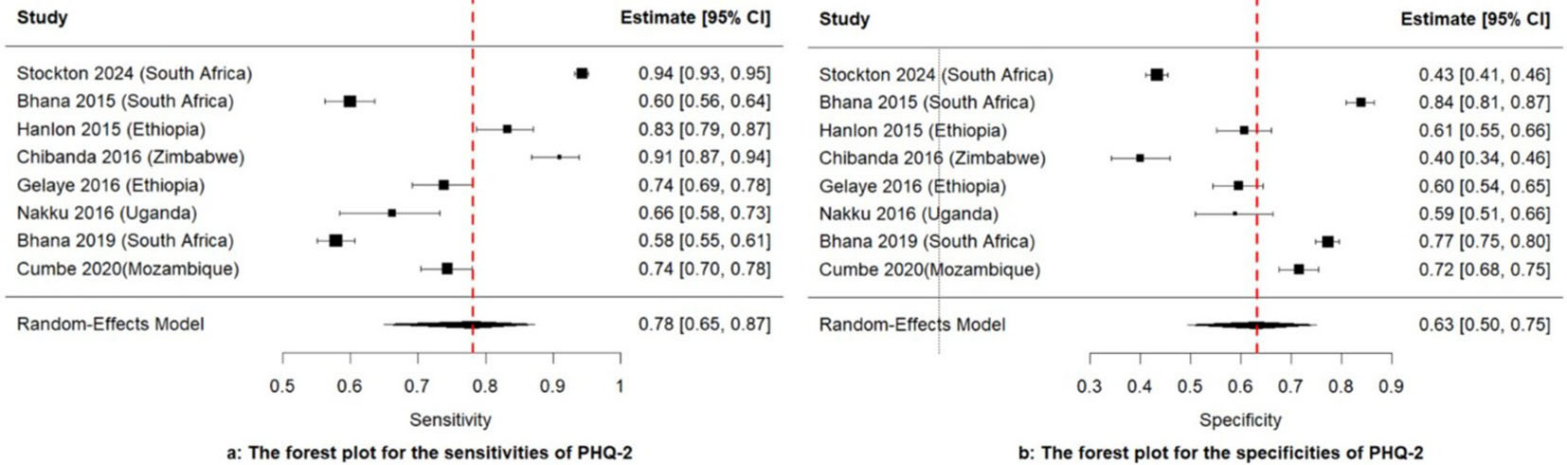
**(a)** The forest plot for the sensitivities of PHQ-2. **(b)** The forest plot for the specificities of PHQ-2.

The HSROC meta-analysis model outputs—as reported in **Table 4**—indicated that the pooled sensitivity and pooled specificity were 0.78 (0.65–0.87) and 0.64 (0.52–0.74), respectively. The 95% CI of the two pooled estimates suggested variability of sensitivity and specificity across eight validation studies for the PHQ-2. We found that the between-study SD for logit-transformed sensitivity and between-study SD for the logit-transformed FPR are 0.89 and 0.69, respectively; and they are high, which also suggests variability in sensitivity and in specificity across validation studies for PHQ-2. The correlation between the logit-transformed sensitivity and the logit-transformed FPR was 0.92, which is strong, meaning that sensitivity and specificity change in the same direction on the logit scale. This suggests that there is no trade-off between sensitivity and specificity for screening depression using PHQ-2. We found a strong correlation between sensitivity and FPR (Spearman’s correlation = 0.74), with marginal statistical significance (*p*-value = 0.046 ≈ 0.05) among the PHQ-2 validation studies, indicating a weak evidence that between-study heterogeneity is mainly attributable to differences in thresholds. We further found that *I*^2^ varied from 65.3% to 76.4% (**Table 5**), and our further investigation on heterogeneity using the forest plot indicated a substantial heterogeneity for both sensitivity and specificity (**Figures 6a, b**). The observed heterogeneity could be attributed to the differences in study settings (different countries) which go with different languages, type of depression diagnosed (depression in general or major depressive disorder), and thresholds (**Table 5**). However, we were not able to perform a sub-group meta-analysis because of the insufficient number of validation studies for PHQ-2.

Our further investigation indicated that one of the studies, the study of Bhana et al. in 2019 (124), involved in the HSROC meta-analysis used sub-optimal reference standard (adult primary care guidelines) against which PHQ-2 was validated. In our sensitivity analysis, by removing the study of Bhana et al. in 2019 (124), we found a slight increase in sensitivity (0.78 vs. 0.81) but with a slight decrease in specificity (0.64 vs. 0.62) compared with the overall HSROC model, and there are no significant changes on the other remaining parameters that need attention (**Table 3.1 in Supplementary Appendix 5**).

##### 3.5.2.4 Kessler Psychological Distress Scale (K-10) for depressive disorders

K-10 was validated for screening depressive disorders in eight validations from five studies conducted in four SSA countries—one in Bukina Faso (55), two in South Africa (60, 65), one in Uganda (77), and one in Ethiopia (93)—with 1,293 participants in total (**Table 6**), aged 15.5 years and above, in general. The cutoff score was the same (10) across all eight validations, while the sensitivity and specificity ranged from 0.53 to 1 and from 0.54 to 0.93 (**Table 6**). The depressive disorders included postnatal depression, current major depressive disorder (MDD), past MDD, past dysthymic disorder, current major depressive episode (MDE), past MDE, and depression, in general.

**Table 6:** Outputs from the HSROC meta-analysis model for Kessler Psychological Distress Scale (K10).

Study author, country	Sample size	Cutoff	Se (95% CI)	Sp (95% CI)	TP	FN	FP	TN	Key outputs of the HSROC model
Baggaley 2007, Burkina Faso	61	10	0.59	0.91	16	11	3	31	1. Pooled se (95% CI): 0.72(0.64-0.78) 2. Pooled FPR (95% CI): 0.23(0.15-0.34) 3. Pooled sp (95% CI)^a^: 0.77 (0.66–0.85) 4. AUC: 0.78 5. Partial AUC (restricted to FPR): 0.71 6. Between study std. dev. for the logit-transformed SE: 0.33 7. Between study std. dev. for the logit-transformed FPR: 0.70 8. rho between the logit-transformed se and the logit-transformed FPR: 0.49 9. *I*^2^ estimate: 14.9%–46.1%
Spies 2009a, South Africa	129	10	0.73	0.54	12	4	52	61	
Spies 2009b, South Africa	129	10	0.53	0.63	10	8	41	70	
Spies 2009c, South Africa	129	10	1	0.93	1	0	9	119	
Spies 2010a, South Africa	429	10	0.67	0.77	153	75	46	155	
Spies 2010b, South Africa	429	10	0.77	0.75	95	28	76	229	
Akena 2013, Uganda	368	10	0.83	0.72	53	11	84	220	
Hanlon 2015, Ethiopia	306	10	0.78	0.767	14	4	67	221	

^a^ Deduced from FPR: 1-FPR.

The HSROC meta-analysis model output (**Table 6**) indicated that the pooled sensitivity and specificity were 0.72 (0.64–0.78) and 0.77 (0.66–0.85), respectively. The 95% CIs of both estimates were wide, suggesting variability of both estimates in eight validations. The between-study SD for the logit-transformed sensitivity and between-study SD for the logit-transformed FPR were 0.33 and 0.70, respectively; and they are high, suggesting variability in sensitivity and specificity between validation studies of K-10 for depressive disorders. The correlation between the logit-transformed sensitivity and the logit-transformed FPR was 0.49, which was positively moderate (162), indicating that heterogeneity is likely influenced by other study-level factors rather than threshold variation. We further found a very weak positive correlation between sensitivity and FPR that was not statistically significant (Spearman’s correlation = 0.108, *p*-value = 0.799), expressing lack of evidence of a threshold effect. The latter is 100% right since the cutoff score was the same across the eight validations.

**Figure 7:**
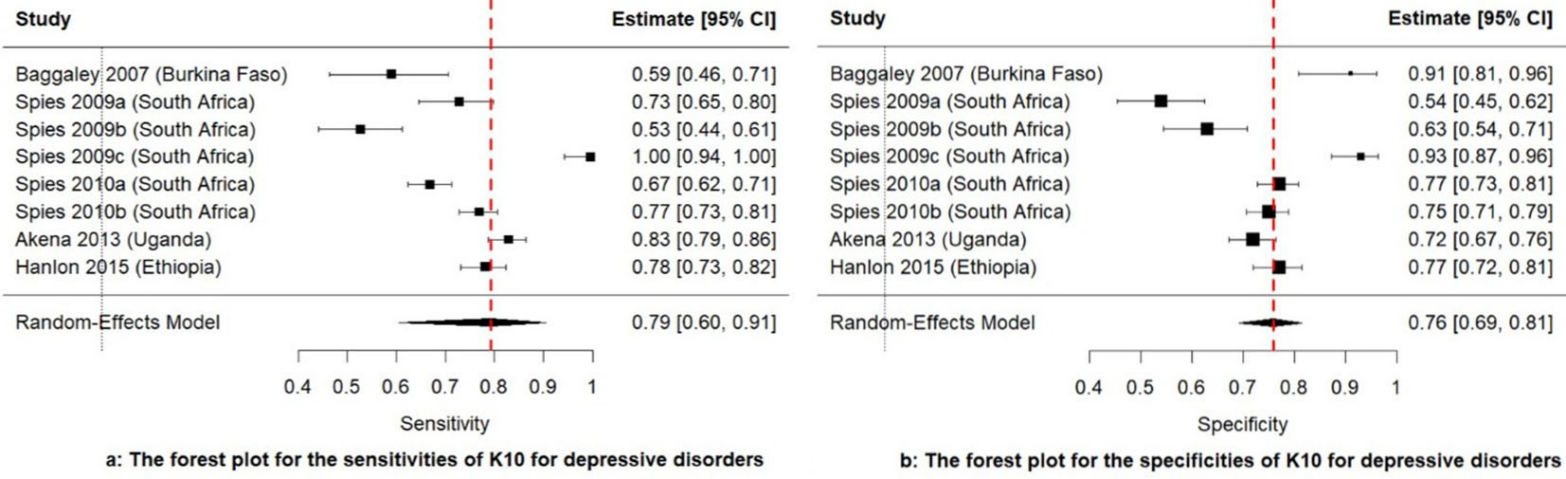
**(a)** The forest plot for the sensitivities of K10 for depressive disorders. **(b)** The forest plot for the specificities of K10 for depressive disorders.

The I^2^ value was 14.9%–46.1%, suggesting that the big proportion of the observed variability is due to chance (**Table 6**). We further investigated heterogeneity via forest plot and did not find contradictions (**Figures 7a, b**). It is important to note that K10 has also been validated for anxiety disorders, common mental disorders, and PTSD; however, meta-analyses were not conducted because of the insufficient number of studies.

#### 3.5.3 Reporting bias

**Figure 8:**
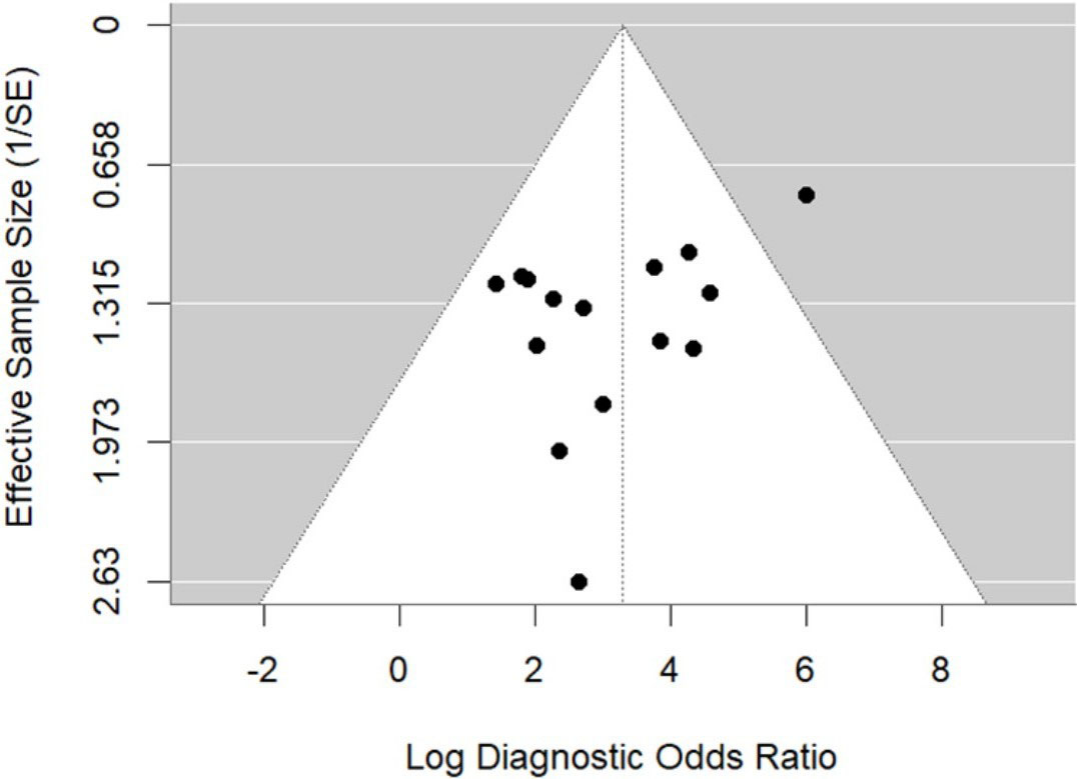
The funnel plot for the EPDS.

**Figure 9:**
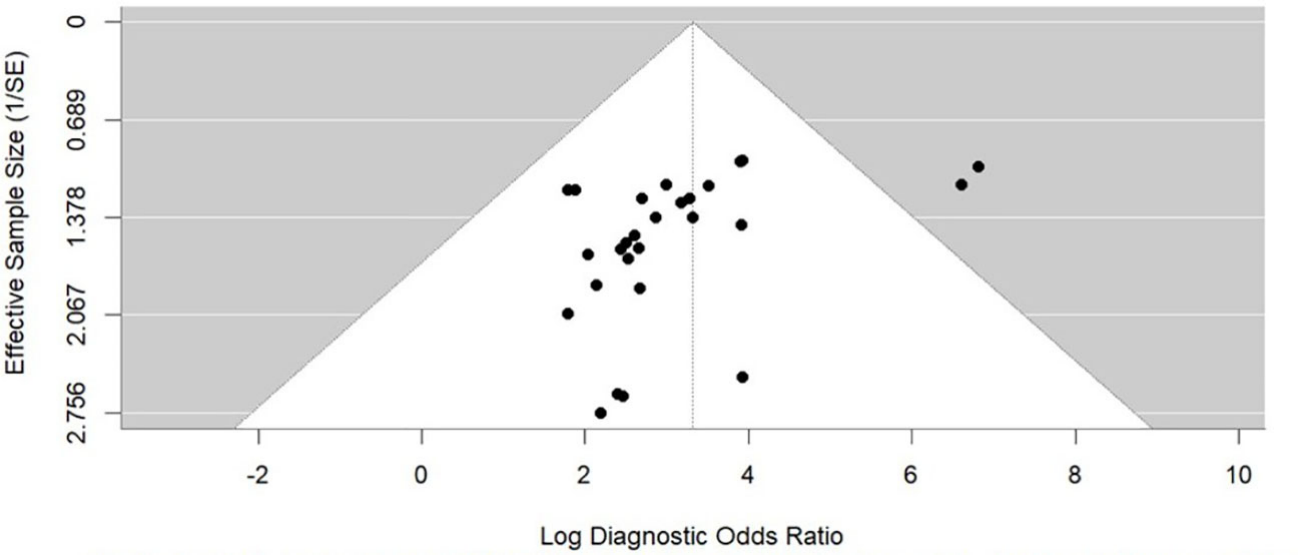
The funnel plot of the PHQ-9 irrespective of administration steps for population categories.

Publication bias assessment was conducted only for the validation studies of EPDS and PHQ-9/PHQ-2/9, as these were the only screening tools that satisfied the key requirement for such assessment, which is a minimum of 10 validation studies (31). For both tools, our visual inspection of Deeks’ funnel plot did not indicate substantial asymmetry (**Figure 8** for EPDS and **Figure 9** for PHQ-9/PHQ-2/9) and agreed with the outputs from the Deeks’ test that showed a non-statistically significant positive regression slope of 2.0 (*P*-value = 0.279) and 2.5 (*P*-value = 0.135) for EPDS and PHQ-9/PHQ-2/9, respectively. This finding suggests no evidence of publication bias for both EPDS and PHQ-9/PHQ-2/9.

## 4 Discussion

To our knowledge, this is the first systematic review and meta-analysis for diagnostic accuracy test for MH screening tools applicable to adolescents in SSA whose age range partially or fully falls within the 12–18 years range, with potential applicability to individuals outside of this age range (**Supplementary Appendix 2**). This review comprehensively identified and synthesized validation studies on MH screening tools over the past quarter century, published between January 1, 2000 and December 31, 2024, and conducted meta-analyses for tools validated in at least five studies (29). This resulted in conducting meta-analyses on four tools only, namely, EPDS, PHQ-9, PHQ-2, and K-10. Furthermore, the publication bias assessment was limited for tools validated in at least 10 studies (31), which permitted us to conduct publication bias on two tools only: EPDS and PHQ-9. The review revealed that healthcare systems in SSA have largely overlooked MH care, as fewer than half of SSA countries (42%, 21 out of 50) have validated and published at least one MH screening tool. These findings are in agreement with prior literature reporting the paucity of MH screening tools in SSA, with tools used in some SSA originally validated in developed countries (18, 48).

The quality of existing validation studies is a concern, as 60% of studies included in the review were rated high risk at least on one domain and 74% of them were rated high concern of applicability on at least one domain. The present review revealed that some validations of MH screening tools do not consider an appropriate reference standard, and this potentially results in validated tools with poor performance in screening MHDs. Therefore, rigor in developing and validating MH screening needs to be a focus in the future. Although the meta-analysis aimed to assess the diagnostic test accuracy across a range of MH screening tools and mental disorders, all validation studies and screening tools forwarded to meta-analysis focused only on one MHD, which is depression, out of 26 broad MHDs for which different screening tools were validated in SSA. This suggests that the validations of MH screening tools applicable to adolescents aged 12–18 years remains limited in SSA, with most available validation studies focusing on depression screening instruments and relatively fewer studies addressing other mental disorders. This is in agreement with the recent systematic review reported that the most frequently screened MH conditions among pregnant and postpartum women was depression in SSA (163). Heterogeneity in sensitivity and specificity were observed across all validation studies to varying extents, and the evidence of publication bias was found for some validation studies and was not for others. Two out of four tools (EPDS and PHQ-9) included in the meta-analyses and considered to be most validated were previously identified as the first two most frequently used out of 47 tools available to screen MHDs in SSA (163). This highlights the disproportionate attention to depression, and it might be due to the paucity of validation studies of MH screening tools and lack of context-specific tools in SSA. This constitutes one of the major barriers to integrating evidence-based MH screening into healthcare systems across SSA (164).

For the EPDS, the pooled sensitivity suggests that the threshold ≥13 may be inadequate in SSA context, as the sensitivities reported in four individual validations—Rochat 2013 (81), Green 2018a/b (113), and van Heyningen 2019 (129)—each using the threshold ≥13, were below the pooled estimate (0.81). The pooled sensitivity also suggests that the threshold—varying from 10 to 12—could be adequate because sensitivities reported in seven out of 10 individual validations—Miofa 2024 (36), Atuhaire 2023, Adewuya 2006a/b (53), Chibanda 2010 (63), Khalifa 2015 (161), and Mutiso 2022 (157)—whose threshold falls in the 10–12 threshold range, were above the pooled sensitivity (0.81). The three studies—Weobong 2008 (62), Chorwe-Sungani (112), and Moya 2022 (152)—that reported the threshold within the 10–12 threshold range do not show any common specific characteristic that could explain this observation. Conversely, the study of Weobong 2008 (62), Chorwe-Sungani 2018 (112), and Moya 2022 (152) had sensitivity below the pooled value despite using threshold in the range of 10–12. We attributed this exception to their relatively small sample size, which may have limited statistical power. For EPDS, the same studies that used the threshold of ≥13 also had individual specificities lower than that of the pooled estimate. In addition to this, two out of three validation studies—Weobong 2008 (62) and Moya 2022 (152), with threshold in the range of 10–12—were found to have individual sensitivities lower than the pooled sensitivity; they also had individual specificities lower than the pooled specificity. What is common to those two studies is that they were conducted among postnatal women, suggesting that the variability in EPDS validations could be linked with the variability in study populations and settings. Since many studies reported sensitivity and specificity close to the pooled estimates, we suggested that EPDS performs consistently across SSA. However, these present findings indicated that the global cutoff of ≥13 (165), originally set in the EPDS validation study (166), should not be generalized to all contexts and study populations.

The AUC of 0.88 from the HSROC meta-analysis model for EPDS indicated moderate diagnostic accuracy (167). The pooled sensitivity (0.81) and specificity (0.83) are higher than those in a recent systematic review in SSA by Gyimah et al. (163), which reported 75.5% and 76.5%, respectively (163). Both estimates from our review (cutoff ranging from 9 to ≥16) and from Gyimah et al. (163) (at a cutoff ≥13) are substantially lower than those in studies outside SSA, such as that by Fellmeth et al. (168) in India (168). The pooled sensitivity from our study (0.82) is higher than the pooled sensitivity from a study conducted among Arabic speaking populations (0.77) at a cutoff ≥11. However, the pooled specificity (0.83) is marginally lower than that among Arabic-speaking populations (0.85) (169). While the cutoff range in our review (9 to 16) differs from those of other reviews, e.g., Shrestha et al. (170), 2–12 (170), it supports a well-established view that optimal cutoffs vary across settings (168). This justifies the need to conduct a specific validation study across local SSA contexts, considering that variability in accuracy for both sensitivity and specificity is likely influenced by sample characteristics, threshold, and methodologies.

The subgroup analysis among prenatal and postpartum women showed no notable changes in estimates. However, in the prenatal group, *I*^2^ expectedly increased, and this may be attributed to the heterogeneity in sample size—the smallest and the highest sample size found in the overall EPDS validation studies were from the prenatal group; this justifies the minimal threshold effect that we found. In contrast to the prenatal group, the subgroup analysis for postpartum women showed a decrease in *I*^2^ and improvements across all parameters, reflecting a better performance of EPDS among postpartum women. This is not surprising because the EPDS was originally developed for the postpartum group, as reflected in its name.

For PHQ-9/PHQ-2/9, the pooled sensitivity (0.79) in this review is slightly higher than the pooled sensitivity reported in previous systematic reviews in general population settings globally (171), though it is slightly lower than that of from the meta-analysis using machine learning at 0.80 (172). The pooled specificity (0.82) is lower than those in the studies of both Manea et al. (2015) (171) and Kim et al. (2021) (172), each of which reported 0.85. These findings highlight variations in performance test accuracy across settings and the need for context-specific validation of PHQ-9/PHQ-2/9 for depression screening in SSA. The observed heterogeneity suggested that subgroup analysis could help explain performance differences because we were not able to deduce any clear patterns from the validation studies’ characteristics (cutoff points, study population, settings, etc.).

Compared to the overall model, the results from three out of four sub-group meta-analyses (one-step PHQ-9 considering all population categories, PHQ-9 irrespective of administration steps considering clinical populations, and one-step PHQ-9 populations) did not show a considerable difference, except a slight increase or decreases in pooled estimates (sensitivity and specificity) and *I*^2^. In the overall model and the three abovementioned sub-group meta-analyses, the presence of a threshold effect was presented and could be attributed to the observed heterogeneity. This implies that the substantial heterogeneity observed across the PHQ-9/PHQ-2/9 validation studies should partly be attributed to the threshold effects, as different validation studies used varying cutoff scores, which are known to influence sensitivity and specificity in diagnostic accuracy meta-analyses, in general, and for PHQ-9 in particular (173). Compared to the overall model, the results from the fourth sub-group analysis—which was about one-step PHQ-9 among non-clinical populations—improved in such a way that the pooled sensitivity and specificity increased and *I*^2^ decreased. Importantly, in this model, there was no evidence of a threshold effect. The findings, under the fourth model, suggest that PHQ-9 is a reliable and comparable measure of depressive disorders across both clinical and non-clinical or general populations in SSA context. These results are in agreement with the study of Doi and colleagues who found PHQ-9 validity and invariance across these groups (174).

For PHQ-2, we found that it performs better in identifying individuals who truly have depression than identifying individuals who truly do not have depression (78% vs. 64%). In the sensitivity meta-analysis, by removing the study of Bhane et al., 2019 (124), we observed a slight increase in sensitivity and a slight decrease in specificity compared with the overall model (0.81 vs. 0.78 and 64 vs. 62, respectively). Changes in other parameters do not need attention as well because they are marginal. Due to the insufficient number of validation studies, we were not able to conduct sub-group meta-analyses in order to draw a robust conclusion. However, PHQ-2 showed a slightly higher pooled sensitivity (0.78) than that reported in Manea et al. (171) for the general population (0.76) (171), but with a lower specificity (0.64 vs. 0.87) (171). The findings suggest that PHQ-2 performs well to correctly identify individuals with depression than exclude those without it. We therefore recommend using PHQ-2 in screening depression in resource- and time-constrained settings when the interest is on identifying the cases.

For K-10 for depressive disorders, we found high heterogeneity and variability of sensitivity and specificity, which we could attribute to the difference in study populations/settings, sample size, and different forms of depression measured across the validation studies. However, we were not able to compare the results from this review with other (previous) studies because of lack of meta-analyses specifically evaluating the diagnostic accuracy of K-10 for depressive disorders.

Regarding overall diagnostic accuracy, EPDS and PHQ-9/PHQ-2/9 showed high diagnostic accuracy (167), with AUC of 0.88 and 0.87, respectively, whereas PHQ-2 and K-10 showed moderate diagnostic accuracy (167), with AUC of 0.76 and 0.78, respectively. This finding suggests that all of these four MH screening tools may be suitable for adaptation and use in other SSA countries or settings under resource and time constraints. Notably, this review also confirms that MH screening tools used in the general population can also be used in HIV-infected populations, particularly those identifying depression. This highlights the need to further investigate the link between MHDs and HIV and to develop MH screening tools tailored for HIV-infected youth in SSA. However, we strongly recommend using PHQ-9/PHQ-2/9 to screen depressive disorders for both clinical populations (including HIV-infected population) and non-clinical populations. We also recommend using EPDS for postpartum women and developing or adopting other tools to screen depression among prenatal women.

Our review and meta-analyses highlight a significant lack of validation studies of MH screening tools applicable to adolescents 12–18 years in SSA, with existing evidence largely limited to depression. This reflects the neglect of mental healthcare in the region (175). The scarcity of validation studies for many screening tools limited our ability to perform meta-analyses. Even for a few screening tools in the meta-analyses (in the case of PHQ-2 and K-10), the small number of validation studies precluded strong conclusions.

In the absence of other validation studies targeting the same study population as our study (adolescents aged 12–18 years), we compared the findings to meta-analyses conducted on different populations on condition that their age range partially or fully overlap the 12–18 years age range mainly targeted in our review. Thus, all conclusions made regarding tool effectiveness, applicability, and adaptability are not robust for adolescents aged 12–18 years across SSA. No evidence of publication bias was identified for EPDS and PHD-9/PHQ-2/9; however, this finding should be interpreted cautiously because the absence of previous systematic reviews or meta-analyses precludes external comparison. The investigation on publication bias was not conducted for PHQ-2 and K-10 due to the small number of studies (fewer than 10) (31, 176).

For tools validated in more than one study, we found that performance accuracy differed across studies (**Supplementary Appendix 2**), and these findings supported the previous findings that difference in study population, settings, and tool administrators can influence prevalence and diagnostic performance (30, 177, 178). This indicates that the reliability of screening tools validated in one setting is not guaranteed when applied in another context without formal adaptation and validation (168)—for instance, using inappropriate threshold can lead to underdiagnoses or overdiagnoses, straining the already overburdened healthcare systems in SSA (170).

We therefore strongly recommend conducting more high-quality validation studies for MH screening tools for adolescents in SSA context. Where local validation is not possible due to resource constraints, our findings suggest using tools validated in another SSA country, at a standardized threshold previously shown to be effective in adolescents, with a slight compromise in test accuracy.

### 4.1 Research, clinical, and policy implications of the review

This review reveals the gaps on validation studies for MH screening tools in SSA, offering foundation for future research. Since majority of the studies in this review considered participants in broader age ranges, not exclusive to 12–18 years, the diagnostic accuracy metrics reported in the present review should not be considered true performance in adolescents aged 12–18 years. Rather, they should be regarded as indicative of potential utility, indicating tools that warrant further age-specific validation. It provides clinicians with a comprehensive list of MH screening tools that could guide clinical decision making. For policymakers, the findings support evidence-based planning and future budget allocations for development and validation studies of MH screening tools across the region.

### 4.2 Strengths and limitations

This systematic review and meta-analysis has four main strengths. First, we conducted a comprehensive and reproducible search, enabling the identification of all MH screening tools applicable to adolescents aged 12–18 years in SSA. Second, the pooled accuracy estimates, at different thresholds, help guide tool selection for research and clinical settings. Third, dual independent screening, data extraction, and quality assessment minimized bias in the process and enhanced credibility. Fourth, our search strategy prioritized elite international databases—MEDLINE, Web of Science, PsycINFO, and CINAHL—that maintain rigorous indexing standards. Fifth, this review consider peer-reviewed and published validation data only, and thus it did not consider conference abstracts or gray literature.

This study has five main limitations. First, majority of the included studies involved participants outside the 12–18 years age range, and this is pragmatically logical, as described in the methodology section under the eligibility criteria. However, the findings from this review should be interpreted with caution because adolescence is a special development stage characterized by rapid emotional, cognitive, and social changes that may influence the expression and interpretation of mental health symptoms (179). Consequently, item understanding and interpretation, response pattern, and symptom thresholds established in adult populations may not be always extrapolated to adolescents, as highlighted in existing literature (180). Second, variability in sample characteristics, thresholds, and reference standards (detailed in **Supplementary Appendix 2** of this paper), along with limited validation studies, restricts comparability and generalizability. Third, due to few validation studies per screening tool, some meta-analyses had limited power, preventing sub-group analyses and weakening conclusions. Fourth, poor reporting was a major issue. Many studies failed to provide key psychometric data (15, 25, 160), such as confidence intervals of sensitivity, specificity, PPV, NPV, and AUC, hindering interpretation, comparability, and use. The same issue was reported in a recent systematic review on tools for screening MH conditions in primary care setting in SSA (163). Fifth, this review did not consider regional African databases, such as African Journals Online (AJOL), and this may limit comprehensiveness. We opted to omit African databases because we wanted to consider elite international databases only, and we did not consider this a concern since high-quality peer-reviewed journals published within Africa and/or hosted on AJOL are also indexed in MEDLINE, Web of Science, PsycINFO, and CINAHL and were considered in this review.

Additionally, some studies reported confusing study sites and participant residence (33, 35, 42, 47, 48, 50, 51, 53, 54, 59, 64, 65, 67, 68, 70, 76, 77, 82, 85, 91, 97, 102, 103, 107, 119, 122, 124, 126, 130, 135, 141, 149), lacked psychometric breakdown by language when tools were administered in multiple languages (33, 34, 55, 90), had missing AUCs despite the fact that ROC analyses were reported in the methods section (37, 42, 116), and inconsistently reported details of administration modes—for example, self-administered vs. clinician-administered (50). Some studies reported only partial data (e.g., scale vs. subscale inconsistency) (43) and omitted gold standard details (71, 80, 82).

Due to underreporting, some studies may have been misclassified as of lower quality when, in fact, data were simply not disclosed. We recommend methodological review and guidelines for future reporting of validation diagnostic accuracy studies. Importantly, we initially targeted the 12–18 years age bracket, and our study selection process did not consider any validation study conducted on populations whose lower age limit exceeded 18 years. We therefore recommend a study that will take this gap into account.

## 5 Conclusion

The review highlights important gaps in the validation of MH screening tools for adolescents in SSA aged 12–18 years, especially for conditions such as psychotic disorders. Over the past 25 years (2000–2024), very few tools have been validated for this age group, and most of these focus only on depression. This points to a broader neglect of MH care among adolescents in SSA. Expanding validation studies tailored to adolescents is the first step to tackling MH disorders in SSA, which has yet to be prioritized because most of the MH conditions among adults start in the early adolescence stage. However, it would be strategic to first understand the extent to which MH care is currently integrated in healthcare systems in SSA and how diagnosis is performed. Reliable and culturally appropriate tools are key to the early diagnosis of MH conditions among adolescents and providing timely and appropriate support.

We recommend future research focusing on adapting existing tools across languages and countries, updating older versions, and, where needed, developing and validating new tools for emerging mental health disorders. We recommend future validation studies of MH screening tools to consider digital screening tools and ensure that the tools are effective for vulnerable groups like HIV-infected adolescents.

## Data Availability

The original contributions presented in the study are included in the article/**Supplementary Material**. Further inquiries can be directed to the corresponding author.
