# Supplementary material for: A systematic review and meta-analysis of diagnostic test accuracy of mental health screening tools applicable to adolescents in sub-Saharan Africa
Source: Front Psychiatry. 2026 Jun 16;17:1728252. doi: 10.3389/fpsyt.2026.1728252 (PMC13317013; doi:10.3389/fpsyt.2026.1728252)
Supplement: Supplementary file 1 [file Table1.docx]

Supplementary appendix 1: Search strategy

1. Search strategy for the Medline

| Interface: Ovid MEDLINE(R) ALL  Date of Search: 20 February 2025  Number of hits: 5,459  Comment: In Ovid, two or more words are automatically searched as phrases; i.e. no quotation marks are needed | Field labels   - exp/ = exploded MeSH term - / = non exploded MeSH term - .ti,ab,kf. = title, abstract and author keywords - adjx = within x words, regardless of order - * = truncation of word for alternate endings |
| --- | --- |
| Database(s): **Ovid MEDLINE(R) ALL**1946 to February 19, 2025 Search Strategy:   \| **#** \| **Searches** \| **Results** \| \| --- \| --- \| --- \| \| 1 \| Psychometrics/ \| 94676 \| \| 2 \| Validation Studies as Topic/ \| 2447 \| \| 3 \| exp Reproducibility of Results/ \| 492208 \| \| 4 \| exp Factor Analysis, Statistical/ \| 35142 \| \| 5 \| validation study.pt. \| 115402 \| \| 6 \| (psychometric* or reliability or validated or validity or validation).ti,ab,kf. \| 1078691 \| \| 7 \| (correlated measure* or Cronbach* alpha or eigenvalue or factor analy* or factor correlation* or factor loading* or internal consistenc* or item-total correlation or Kaiser Criterion or Kaiser-Guttman rule or oblique rotation or orthogonal rotation or scree plot*).ti,ab,kf. \| 115827 \| \| 8 \| or/1-7 \| 1463339 \| \| 9 \| exp Mental Disorders/ \| 1532071 \| \| 10 \| Mental health/ \| 71450 \| \| 11 \| exp Self concept/ \| 127977 \| \| 12 \| exp Social support/ \| 84386 \| \| 13 \| exp Quality of life/ \| 301296 \| \| 14 \| ((affective or alcohol or adjustment or amphetamine or antisocial or attachment or attention deficit or behavio?r or bipolar or body dysmorphic or body integrity or borderline or capgras or cocaine or cognition or combat or communication or compulsive or conduct or conversion or consciousness or development* or dissociative or dysmorphic or eating or elimination or factitious or feeding or gender or intellectual or learning or mental or mood or motor or munchausen or narcotic? or neurodevelopmental or neurotic or opioid or panic or paranoid or passive-aggressive or personality or phobic or psychiatric or psychotic or rumination or schizoid or sleep or somatoform or speech-sound or stress* or substance or tic? or tobacco or trauma) adj3 (disorder? or disabilit* or disease? or diagnos#s or illness* or syndrome?)).ti,ab,kf. \| 813380 \| \| 15 \| (anorexia or anorectic or bulimia or bulimic or diabulimia or orthorexia or pica).ti,ab,kf. \| 48155 \| \| 16 \| (aperger* or autism or autistic or adhd).ti,ab,kf. \| 108372 \| \| 17 \| (encopresis or enuresis or gambl* or gender dysphoria or hypochondria* or neurasthen* or parasomnia* or psychos#s or pyromani* or schizophreni* or tourette* or trichotillomania).ti,ab,kf. \| 226561 \| \| 18 \| (post-traumatic or ((psychological or sexual or military) adj3 trauma*)).ti,ab,kf. \| 51515 \| \| 19 \| ((mental* or psychiatric or psychologic*) adj3 (well* or health*)).ti,ab,kf. \| 327074 \| \| 20 \| (anxiet* or phobia? or agrophobia? or kinesiophobia? or panic attack?).ti,ab,kf. \| 313058 \| \| 21 \| (addict* or alcoholism).ti,ab,kf. \| 118283 \| \| 22 \| ((alcohol or amphetamine? or angel dust or cannabis or cocaine or drug? or glue or hashish or heroin or inhalant or marihuana or marijuana or narcotic* or nicotine or opiate? or opioid? or opium or pcp or phencyclidine? or substance?) adj3 (abuse or abusing or dependen* or misus* or problem? or use*)).ti,ab,kf. \| 482018 \| \| 23 \| (depress* or involutional paraphrenia? or involutional psychos#s or dysthymic disorder? or dysthymia or premenstrual dysphoric or seasonal mood disorder? or seasonal affective disorder?).ti,ab,kf. \| 625175 \| \| 24 \| (self adj (concept* or confiden* or efficac* or esteem* or perception*)).ti,ab,kf. \| 92720 \| \| 25 \| ((social or psychosocial or psychological or family or community) adj3 support).ti,ab,kf. \| 105372 \| \| 26 \| quality of life.ti,ab,kf. \| 441649 \| \| 27 \| or/9-26 \| 3449599 \| \| 28 \| exp Africa South of the Sahara/ \| 279873 \| \| 29 \| Developing Countries/ \| 83550 \| \| 30 \| ((deprived or developing or emerging or less* developed or low* income or middle income or poor* or third-world or under developed or under served or underdeveloped or underserved) adj3 (countr* or economies or economy or nation? or population? or world)).ti,ab,kf. \| 206664 \| \| 31 \| (low* adj2 (countr* or gdp or gnp or gross domestic or gross national)).ti,ab,kf. \| 19842 \| \| 32 \| ((low* or middle*) adj4 (countr* or nation*)).ti,ab,kf. \| 76649 \| \| 33 \| (Global South or LIC or LMIC* or MIC or South-South).ti,ab,kf. \| 74430 \| \| 34 \| (africa* or angola* or benin* or botswan* or burkina faso* or cabo verde* or cameroon* or central african republic or chad or congo* or cote d'ivoire or "democratic republic of the congo" or djibouti* or equatorial guinea* or eritrea* or eswatini* or ethiopia* or gabon* or gambia* or ghan* or guinea* or guinea-bissau or kenya* or lesotho* or liberia* or malawi* or mali or mauritania* or mozambique* or namibia* or niger* or nigeria or rwanda* or "sao tome and principe" or senegal* or sierra leone or somali* or south africa or south sudan or sudan* or tanzania* or togo or uganda* or zambia* or zimbabwe*).ti,ab,kf. \| 657583 \| \| 35 \| or/28-34 \| 951155 \| \| 36 \| exp Psychological tests/ \| 358748 \| \| 37 \| exp "Surveys and Questionnaires"/ \| 1293935 \| \| 38 \| Mass Screening/ \| 120639 \| \| 39 \| Diagnostic Tests, Routine/ \| 15467 \| \| 40 \| (construct? or dimension? or item? or screen* or scale* or test* or questionnaire* or survey* or tool?).ti,ab,kf. \| 8096614 \| \| 41 \| or/36-40 \| 8645652 \| \| 42 \| 8 and 27 and 35 and 41 \| 5909 \| \| 43 \| limit 42 to yr="2000 - 2024" \| 5459 \|  \|  \| \| --- \| | |

2. Search strategy for the Web of Science Core Collection

| Interface: Clarivate Analytics  Editions = A&HCI , ESCI , SCI-EXPANDED , SSCI  Date of Search: 20 February 2025  Number of hits: 4,202 | Field labels   - TI= title - AB=abstract - AK=author keywords - NEAR/x = within x words, regardless of order - * = truncation of word for alternate endings   Note: the *Exact search*-function was used for all the searches |
| --- | --- |
| \| # \| Search Query \| Results \| \| --- \| --- \| --- \| \| 1 \| TI=(psychometric* OR reliability OR validated OR validity OR validation ) OR AB=(psychometric* OR reliability OR validated OR validity OR validation ) OR AK=(psychometric* OR reliability OR validated OR validity OR validation ) \| 2173290 \| \| 2 \| TI=("correlated measure*" OR "Cronbach* alpha" OR eigenvalue OR "factor analy*" OR "factor correlation*" OR "factor loading*" OR "internal consistenc*" OR "item-total correlation" OR "Kaiser Criterion" OR "Kaiser-Guttman rule" OR "oblique rotation" OR "orthogonal rotation" OR "scree plot*" ) OR AB=("correlated measure*" OR "Cronbach* alpha" OR eigenvalue OR "factor analy*" OR "factor correlation*" OR "factor loading*" OR "internal consistenc*" OR "item-total correlation" OR "Kaiser Criterion" OR "Kaiser-Guttman rule" OR "oblique rotation" OR "orthogonal rotation" OR "scree plot*" ) OR AK=("correlated measure*" OR "Cronbach* alpha" OR eigenvalue OR "factor analy*" OR "factor correlation*" OR "factor loading*" OR "internal consistenc*" OR "item-total correlation" OR "Kaiser Criterion" OR "Kaiser-Guttman rule" OR "oblique rotation" OR "orthogonal rotation" OR "scree plot*" ) \| 238659 \| \| 3 \| #2 OR #1 \| 2313371 \| \| 4 \| TI=((affective OR alcohol OR adjustment OR amphetamine OR antisocial OR attachment OR "attention deficit" OR behavio$r OR bipolar OR "body dysmorphic" OR "body integrity" OR borderline OR capgras OR cocaine OR cognition OR combat OR communication OR compulsive OR conduct OR conversion OR consciousness OR development* OR dissociative OR dysmorphic OR eating OR elimination OR factitious OR feeding OR gender OR intellectual OR learning OR mental OR mood OR motor OR munchausen OR narcotic$ OR neurodevelopmental OR neurotic OR opioid OR panic OR paranoid OR passive-aggressive OR personality OR phobic OR psychiatric OR psychotic OR rumination OR schizoid OR sleep OR somatoform OR speech-sound OR stress* OR substance OR tic$ OR tobacco OR trauma ) NEAR/2 (disorder$ OR disabilit* OR disease$ OR diagnos?s OR illness* OR syndrome$ )) OR AB=((affective OR alcohol OR adjustment OR amphetamine OR antisocial OR attachment OR "attention deficit" OR behavio$r OR bipolar OR "body dysmorphic" OR "body integrity" OR borderline OR capgras OR cocaine OR cognition OR combat OR communication OR compulsive OR conduct OR conversion OR consciousness OR development* OR dissociative OR dysmorphic OR eating OR elimination OR factitious OR feeding OR gender OR intellectual OR learning OR mental OR mood OR motor OR munchausen OR narcotic$ OR neurodevelopmental OR neurotic OR opioid OR panic OR paranoid OR passive-aggressive OR personality OR phobic OR psychiatric OR psychotic OR rumination OR schizoid OR sleep OR somatoform OR speech-sound OR stress* OR substance OR tic$ OR tobacco OR trauma ) NEAR/2 (disorder$ OR disabilit* OR disease$ OR diagnos?s OR illness* OR syndrome$ )) OR AK=((affective OR alcohol OR adjustment OR amphetamine OR antisocial OR attachment OR "attention deficit" OR behavio$r OR bipolar OR "body dysmorphic" OR "body integrity" OR borderline OR capgras OR cocaine OR cognition OR combat OR communication OR compulsive OR conduct OR conversion OR consciousness OR development* OR dissociative OR dysmorphic OR eating OR elimination OR factitious OR feeding OR gender OR intellectual OR learning OR mental OR mood OR motor OR munchausen OR narcotic$ OR neurodevelopmental OR neurotic OR opioid OR panic OR paranoid OR passive-aggressive OR personality OR phobic OR psychiatric OR psychotic OR rumination OR schizoid OR sleep OR somatoform OR speech-sound OR stress* OR substance OR tic$ OR tobacco OR trauma ) NEAR/2 (disorder$ OR disabilit* OR disease$ OR diagnos?s OR illness* OR syndrome$ )) \| 927963 \| \| 5 \| TI=(aperger* OR autism OR autistic OR adhd ) OR AB=(aperger* OR autism OR autistic OR adhd ) OR AK=(aperger* OR autism OR autistic OR adhd ) \| 143325 \| \| 6 \| TI=(encopresis OR enuresis OR gambl* OR "gender dysphoria" OR hypochondria* OR neurasthen* OR parasomnia* OR psychos?s OR pyromani* OR schizophreni* OR tourette* OR trichotillomania ) OR AB=(encopresis OR enuresis OR gambl* OR "gender dysphoria" OR hypochondria* OR neurasthen* OR parasomnia* OR psychos?s OR pyromani* OR schizophreni* OR tourette* OR trichotillomania ) OR AK=(encopresis OR enuresis OR gambl* OR "gender dysphoria" OR hypochondria* OR neurasthen* OR parasomnia* OR psychos?s OR pyromani* OR schizophreni* OR tourette* OR trichotillomania ) \| 288721 \| \| 7 \| TI=(post-traumatic OR ((psychological OR sexual OR military ) NEAR/2 trauma* )) OR AB=(post-traumatic OR ((psychological OR sexual OR military ) NEAR/2 trauma* )) OR AK=(post-traumatic OR ((psychological OR sexual OR military ) NEAR/2 trauma* )) \| 54463 \| \| 8 \| TI=(anxiet* OR phobia$ OR agrophobia$ OR kinesiophobia$ OR "panic attack$" ) OR AB=(anxiet* OR phobia$ OR agrophobia$ OR kinesiophobia$ OR "panic attack$" ) OR AK=(anxiet* OR phobia$ OR agrophobia$ OR kinesiophobia$ OR "panic attack$" ) \| 388122 \| \| 9 \| TI=(addict* OR alcoholism ) OR AB=(addict* OR alcoholism ) OR AK=(addict* OR alcoholism ) \| 139358 \| \| 10 \| TI=(anorexia OR anorectic OR bulimia OR bulimic OR diabulimia OR orthorexia OR pica ) OR AB=(anorexia OR anorectic OR bulimia OR bulimic OR diabulimia OR orthorexia OR pica ) OR  AK=(anorexia OR anorectic OR bulimia OR bulimic OR diabulimia OR orthorexia OR pica ) \| 54824 \| \| 11 \| TI=((alcohol OR amphetamine$ OR "angel dust" OR cannabis OR cocaine OR drug$ OR glue OR hashish OR heroin OR inhalant OR marihuana OR marijuana OR narcotic* OR nicotine OR opiate$ OR opioid$ OR opium OR pcp OR phencyclidine$ OR substance$ ) NEAR/2 (abuse OR abusing OR dependen* OR misus* OR problem$ OR use* )) OR AB=((alcohol OR amphetamine$ OR "angel dust" OR cannabis OR cocaine OR drug$ OR glue OR hashish OR heroin OR inhalant OR marihuana OR marijuana OR narcotic* OR nicotine OR opiate$ OR opioid$ OR opium OR pcp OR phencyclidine$ OR substance$ ) NEAR/2 (abuse OR abusing OR dependen* OR misus* OR problem$ OR use* )) OR AK=((alcohol OR amphetamine$ OR "angel dust" OR cannabis OR cocaine OR drug$ OR glue OR hashish OR heroin OR inhalant OR marihuana OR marijuana OR narcotic* OR nicotine OR opiate$ OR opioid$ OR opium OR pcp OR phencyclidine$ OR substance$ ) NEAR/2 (abuse OR abusing OR dependen* OR misus* OR problem$ OR use* )) \| 529936 \| \| 12 \| TI=(depress* OR "involutional paraphrenia$" OR "involutional psychos*" OR "dysthymic disorder*" or dysthymia or "premenstrual dysphoric" or "seasonal mood disorder*" or "seasonal affective disorder*") OR AB=(depress* OR "involutional paraphrenia$" OR "involutional psychos*" OR "dysthymic disorder*" or dysthymia or "premenstrual dysphoric" or "seasonal mood disorder*" or "seasonal affective disorder*") OR AK=(depress* OR "involutional paraphrenia$" OR "involutional psychos*" OR "dysthymic disorder*" or dysthymia or "premenstrual dysphoric" or "seasonal mood disorder*" or "seasonal affective disorder*") \| 782364 \| \| 13 \| TI=(self NEAR/0 (concept* OR confiden* OR efficac* OR esteem* OR perception* )) OR AB=(self NEAR/0 (concept* OR confiden* OR efficac* OR esteem* OR perception* )) OR AK=(self NEAR/0 (concept* OR confiden* OR efficac* OR esteem* OR perception* )) \| 160158 \| \| 14 \| TI=((social OR psychosocial OR psychological OR family OR community ) NEAR/2 support ) OR AB=((social OR psychosocial OR psychological OR family OR community ) NEAR/2 support ) OR AK=((social OR psychosocial OR psychological OR family OR community ) NEAR/2 support ) \| 144852 \| \| 15 \| TI="quality of life" OR AB="quality of life" OR AK="quality of life" \| 485830 \| \| 16 \| #4 OR #5 OR #6 OR #7 OR #8 OR #9 OR #10 OR #11 OR #12 OR #13 OR #14 OR #15 \| 3131125 \| \| 17 \| TI=((deprived OR developing OR emerging OR "less* developed" OR "low* income" OR "middle income" OR poor* OR third-world OR "under developed" OR "under served" OR underdeveloped OR underserved ) NEAR/2 (countr* OR economies OR economy OR nation$ OR population$ OR world )) OR  AB=((deprived OR developing OR emerging OR "less* developed" OR "low* income" OR "middle income" OR poor* OR third-world OR "under developed" OR "under served" OR underdeveloped OR underserved ) NEAR/2 (countr* OR economies OR economy OR nation$ OR population$ OR world )) OR  AK=((deprived OR developing OR emerging OR "less* developed" OR "low* income" OR "middle income" OR poor* OR third-world OR "under developed" OR "under served" OR underdeveloped OR underserved ) NEAR/2 (countr* OR economies OR economy OR nation$ OR population$ OR world )) \| 330072 \| \| 18 \| TI=(low* NEAR/2 (countr* OR gdp OR gnp OR "gross domestic" OR "gross national" )) OR AB=(low* NEAR/2 (countr* OR gdp OR gnp OR "gross domestic" OR "gross national" )) OR AK=(low* NEAR/2 (countr* OR gdp OR gnp OR "gross domestic" OR "gross national" )) \| 42918 \| \| 19 \| TI=((low* OR middle* ) NEAR/3 (countr* OR nation* )) OR AB=((low* OR middle* ) NEAR/3 (countr* OR nation* )) OR AK=((low* OR middle* ) NEAR/3 (countr* OR nation* )) \| 101269 \| \| 20 \| TI=("Global South" OR LIC OR LMIC* OR MIC OR South-South ) OR AB=("Global South" OR LIC OR LMIC* OR MIC OR South-South ) OR AK=("Global South" OR LIC OR LMIC* OR MIC OR South-South ) \| 106986 \| \| 21 \| TI=(africa* OR angola* OR benin* OR botswan* OR "burkina faso*" OR "cabo verde*" OR cameroon* OR "central african republic" OR chad OR congo* OR "cote d'ivoire" OR "democratic republic of the congo" OR djibouti* OR "equatorial guinea*" OR eritrea* OR eswatini* OR ethiopia* OR gabon* OR gambia* OR ghan* OR guinea* OR guinea-bissau OR kenya* OR lesotho* OR liberia* OR malawi* OR mali OR mauritania* OR mozambique* OR namibia* OR niger* OR nigeria OR rwanda* OR "sao tome and principe" OR senegal* OR "sierra leone" OR somali* OR "south africa" OR "south sudan" OR sudan* OR tanzania* OR togo OR uganda* OR zambia* OR zimbabwe* ) OR AB=(africa* OR angola* OR benin* OR botswan* OR "burkina faso*" OR "cabo verde*" OR cameroon* OR "central african republic" OR chad OR congo* OR "cote d'ivoire" OR "democratic republic of the congo" OR djibouti* OR "equatorial guinea*" OR eritrea* OR eswatini* OR ethiopia* OR gabon* OR gambia* OR ghan* OR guinea* OR guinea-bissau OR kenya* OR lesotho* OR liberia* OR malawi* OR mali OR mauritania* OR mozambique* OR namibia* OR niger* OR nigeria OR rwanda* OR "sao tome and principe" OR senegal* OR "sierra leone" OR somali* OR "south africa" OR "south sudan" OR sudan* OR tanzania* OR togo OR uganda* OR zambia* OR zimbabwe* ) OR AK=(africa* OR angola* OR benin* OR botswan* OR "burkina faso*" OR "cabo verde*" OR cameroon* OR "central african republic" OR chad OR congo* OR "cote d'ivoire" OR "democratic republic of the congo" OR djibouti* OR "equatorial guinea*" OR eritrea* OR eswatini* OR ethiopia* OR gabon* OR gambia* OR ghan* OR guinea* OR guinea-bissau OR kenya* OR lesotho* OR liberia* OR malawi* OR mali OR mauritania* OR mozambique* OR namibia* OR niger* OR nigeria OR rwanda* OR "sao tome and principe" OR senegal* OR "sierra leone" OR somali* OR "south africa" OR "south sudan" OR sudan* OR tanzania* OR togo OR uganda* OR zambia* OR zimbabwe* ) \| 1284975 \| \| 22 \| #17 OR #18 OR #19 OR #20 OR #21 \| 1668442 \| \| 23 \| TI=(construct$ OR dimension$ OR item$ OR screen* OR scale* OR test* OR questionnaire* OR survey* OR tool$ ) OR AB=(construct$ OR dimension$ OR item$ OR screen* OR scale* OR test* OR questionnaire* OR survey* OR tool$ ) OR AK=(construct$ OR dimension$ OR item$ OR screen* OR scale* OR test* OR questionnaire* OR survey* OR tool$ ) \| 14677063 \| \| 24 \| #23 AND #22 AND #16 AND #3 \| 4400 \| \| 25 \| #23 AND #22 AND #16 AND #3 Timespan: 2000-01-01 to 2024-12-31 \| 4202 \| | |

3. Search strategy for the PsycINFO

| Interface: EBSCO  Date of Search: 20 February 2025  Number of hits: 3,538 | Field labels   - DE = subject heading - TI = title - AB = abstract - KW = author keywords - Nx = within x words, regardless of order - * = truncation of word for alternate endings |
| --- | --- |
| \| **#** \| **Query** \| **Results** \| \| --- \| --- \| --- \| \| S38 \| S4 AND S23 AND S30 AND S36  Limiters - Publication Year: 2000-2024 \| 3,538 \| \| S37 \| S4 AND S23 AND S30 AND S36 \| 3,912 \| \| S36 \| S31 OR S32 OR S33 OR S34 OR S35 \| 2,159,973 \| \| S35 \| TI ( (construct# OR dimension# OR item# OR screen* OR scale* OR test* OR questionnaire* OR survey* OR tool# ) ) OR AB ( (construct# OR dimension# OR item# OR screen* OR scale* OR test* OR questionnaire* OR survey* OR tool# ) ) OR KW ( (construct# OR dimension# OR item# OR screen* OR scale* OR test* OR questionnaire* OR survey* OR tool# ) ) \| 2,114,085 \| \| S34 \| DE "Checklist (Testing)" OR DE "Child Behavior Checklist" OR DE "Questionnaires" OR DE "General Health Questionnaire" OR DE "Surveys" OR DE "Consumer Surveys" OR DE "Mail Surveys" OR DE "Online Surveys" OR DE "Telephone Surveys" OR DE "Rating Scales" OR DE "Likert Scales" OR DE "Test Types" \| 73,217 \| \| S33 \| DE "Scaling (Testing)" \| 2,247 \| \| S32 \| DE "Screening Tests" OR DE "Psychological Screening Inventory" OR DE "Screening" OR DE "Screening Tests" \| 20,333 \| \| S31 \| DE "Psychometrics" OR DE "Classical Test Theory" OR DE "Consistency (Measurement)" OR DE "Error of Measurement" OR DE "External Validity" OR DE "Factor Analysis" OR DE "Internal Validity" OR DE "Item Analysis (Test)" OR DE "Item Response Theory" OR DE "Measurement Invariance" OR DE "Measurement Models" OR DE "Multivariate Analysis" OR DE "Test Construction" OR DE "Test Reliability" OR DE "Test Sensitivity" OR DE "Test Specificity" OR DE "Test Validity" OR DE "Variability Measurement" \| 247,465 \| \| S30 \| S24 OR S25 OR S26 OR S27 OR S28 OR S29 \| 165,124 \| \| S29 \| TI ( (africa* OR angola* OR benin* OR botswan* OR "burkina faso*" OR "cabo verde*" OR cameroon* OR "central african republic" OR chad OR congo* OR "cote d'ivoire" OR "democratic republic of the congo" OR djibouti* OR "equatorial guinea*" OR eritrea* OR eswatini* OR ethiopia* OR gabon* OR gambia* OR ghan* OR guinea* OR guinea-bissau OR kenya* OR lesotho* OR liberia* OR malawi* OR mali OR mauritania* OR mozambique* OR namibia* OR niger* OR nigeria OR rwanda* OR "sao tome and principe" OR senegal* OR "sierra leone" OR somali* OR "south africa" OR "south sudan" OR sudan* OR tanzania* OR togo OR uganda* OR zambia* OR zimbabwe* ) ) OR AB ( (africa* OR angola* OR benin* OR botswan* OR "burkina faso*" OR "cabo verde*" OR cameroon* OR "central african republic" OR chad OR congo* OR "cote d'ivoire" OR "democratic republic of the congo" OR djibouti* OR "equatorial guinea*" OR eritrea* OR eswatini* OR ethiopia* OR gabon* OR gambia* OR ghan* OR guinea* OR guinea-bissau OR kenya* OR lesotho* OR liberia* OR malawi* OR mali OR mauritania* OR mozambique* OR namibia* OR niger* OR nigeria OR rwanda* OR "sao tome and principe" OR senegal* OR "sierra leone" OR somali* OR "south africa" OR "south sudan" OR sudan* OR tanzania* OR togo OR uganda* OR zambia* OR zimbabwe* ) ) OR KW ( (africa* OR angola* OR benin* OR botswan* OR "burkina faso*" OR "cabo verde*" OR cameroon* OR "central african republic" OR chad OR congo* OR "cote d'ivoire" OR "democratic republic of the congo" OR djibouti* OR "equatorial guinea*" OR eritrea* OR eswatini* OR ethiopia* OR gabon* OR gambia* OR ghan* OR guinea* OR guinea-bissau OR kenya* OR lesotho* OR liberia* OR malawi* OR mali OR mauritania* OR mozambique* OR namibia* OR niger* OR nigeria OR rwanda* OR "sao tome and principe" OR senegal* OR "sierra leone" OR somali* OR "south africa" OR "south sudan" OR sudan* OR tanzania* OR togo OR uganda* OR zambia* OR zimbabwe* ) ) \| 131,824 \| \| S28 \| TI ( ("Global South" OR LIC OR LMIC* OR MIC OR South-South ) ) OR KW ( ("Global South" OR LIC OR LMIC* OR MIC OR South-South ) ) OR AB ( ("Global South" OR LIC OR LMIC* OR MIC OR South-South ) ) \| 3,974 \| \| S27 \| TI ( ((low* OR middle* ) N3 (countr* OR nation* )) ) OR AB ( ((low* OR middle* ) N3 (countr* OR nation* )) ) OR KW ( ((low* OR middle* ) N3 (countr* OR nation* )) ) \| 12,745 \| \| S26 \| TI ( ( (low* N2 (countr* OR gdp OR gnp OR "gross domestic" OR "gross national" )) ) OR AB ( ( (low* N2 (countr* OR gdp OR gnp OR "gross domestic" OR "gross national" )) ) OR KW ( ( (low* N2 (countr* OR gdp OR gnp OR "gross domestic" OR "gross national" )) ) \| 4,732 \| \| S25 \| TI ( ((deprived OR developing OR emerging OR "less* developed" OR "low* income" OR "middle income" OR poor* OR third-world OR "under developed" OR "under served" OR underdeveloped OR underserved ) N2 (countr* OR economies OR economy OR nation# OR population# OR world )) ) OR AB ( ((deprived OR developing OR emerging OR "less* developed" OR "low* income" OR "middle income" OR poor* OR third-world OR "under developed" OR "under served" OR underdeveloped OR underserved ) N2 (countr* OR economies OR economy OR nation# OR population# OR world )) ) OR KW ( ((deprived OR developing OR emerging OR "less* developed" OR "low* income" OR "middle income" OR poor* OR third-world OR "under developed" OR "under served" OR underdeveloped OR underserved ) N2 (countr* OR economies OR economy OR nation# OR population# OR world )) ) \| 33,297 \| \| S24 \| DE "Developing Countries" \| 9,999 \| \| S23 \| S5 OR S6 OR S7 OR S8 OR S9 OR S10 OR S11 OR S12 OR S13 OR S14 OR S15 OR S16 OR S17 OR S18 OR S19 OR S20 OR S21 OR S22 \| 1,867,606 \| \| S22 \| TI "quality of life" OR AB "quality of life" OR KW "quality of life" \| 94,701 \| \| S21 \| TI ( ((social OR psychosocial OR psychological OR family OR community ) N2 support ) ) ) OR KW ( ((social OR psychosocial OR psychological OR family OR community ) N2 support ) ) ) OR AB ( ((social OR psychosocial OR psychological OR family OR community ) N2 support ) ) ) \| 106,919 \| \| S20 \| TI ( ( (self W1 (concept* OR confiden* OR efficac* OR esteem* OR perception* )) ) OR AB ( ( (self W1 (concept* OR confiden* OR efficac* OR esteem* OR perception* )) ) OR KW ( ( (self W1 (concept* OR confiden* OR efficac* OR esteem* OR perception* )) ) \| 148,826 \| \| S19 \| TI ( (depress* OR "involutional paraphrenia#" OR "involutional psychos*" OR "dysthymic disorder#" OR dysthymia OR "premenstrual dysphoric" OR "seasonal mood disorder#" OR "seasonal affective disorder#" ) ) OR AB ( (depress* OR "involutional paraphrenia#" OR "involutional psychos*" OR "dysthymic disorder#" OR dysthymia OR "premenstrual dysphoric" OR "seasonal mood disorder#" OR "seasonal affective disorder#" ) ) OR KW ( (depress* OR "involutional paraphrenia#" OR "involutional psychos*" OR "dysthymic disorder#" OR dysthymia OR "premenstrual dysphoric" OR "seasonal mood disorder#" OR "seasonal affective disorder#" ) ) \| 383,001 \| \| S18 \| TI ( ((alcohol OR amphetamine# OR "angel dust" OR cannabis OR cocaine OR drug# OR glue OR hashish OR heroin OR inhalant OR marihuana OR marijuana OR narcotic* OR nicotine OR opiate# OR opioid# OR opium OR pcp OR phencyclidine# OR substance# ) N2 (abuse OR abusing OR dependen* OR misus* OR problem# OR use* )) ) OR AB ( ((alcohol OR amphetamine# OR "angel dust" OR cannabis OR cocaine OR drug# OR glue OR hashish OR heroin OR inhalant OR marihuana OR marijuana OR narcotic* OR nicotine OR opiate# OR opioid# OR opium OR pcp OR phencyclidine# OR substance# ) N2 (abuse OR abusing OR dependen* OR misus* OR problem# OR use* )) ) OR KW ( ((alcohol OR amphetamine# OR "angel dust" OR cannabis OR cocaine OR drug# OR glue OR hashish OR heroin OR inhalant OR marihuana OR marijuana OR narcotic* OR nicotine OR opiate# OR opioid# OR opium OR pcp OR phencyclidine# OR substance# ) N2 (abuse OR abusing OR dependen* OR misus* OR problem# OR use* )) ) \| 231,290 \| \| S17 \| TI ( (addict* OR alcoholism ) ) OR AB ( (addict* OR alcoholism ) ) OR KW ( (addict* OR alcoholism ) ) \| 87,348 \| \| S16 \| TI ( (anxiet* OR phobia# OR agrophobia# OR kinesiophobia# OR "panic attack#" ) ) OR AB ( (anxiet* OR phobia# OR agrophobia# OR kinesiophobia# OR "panic attack#" ) ) OR KW ( (anxiet* OR phobia# OR agrophobia# OR kinesiophobia# OR "panic attack#" ) ) \| 264,838 \| \| S15 \| TI ( ((mental* OR psychiatric OR psychologic* ) N2 (well* OR health* )) ) OR AB ( ((mental* OR psychiatric OR psychologic* ) N2 (well* OR health* )) ) OR KW ( ((mental* OR psychiatric OR psychologic* ) N2 (well* OR health* )) ) \| 314,897 \| \| S14 \| TI ( ( (post-traumatic OR ((psychological OR sexual OR military ) N2 trauma* )) ) OR AB ( ( (post-traumatic OR ((psychological OR sexual OR military ) N2 trauma* )) ) OR KW ( ( (post-traumatic OR ((psychological OR sexual OR military ) N2 trauma* )) ) \| 29,681 \| \| S13 \| TI ( encopresis OR enuresis OR gambl* OR "gender dysphoria" OR hypochondria* OR neurasthen* OR parasomnia* OR psychos?s OR pyromani* OR schizophreni* OR tourette* OR trichotillomania ) ) OR AB ( encopresis OR enuresis OR gambl* OR "gender dysphoria" OR hypochondria* OR neurasthen* OR parasomnia* OR psychos?s OR pyromani* OR schizophreni* OR tourette* OR trichotillomania ) ) OR KW ( encopresis OR enuresis OR gambl* OR "gender dysphoria" OR hypochondria* OR neurasthen* OR parasomnia* OR psychos?s OR pyromani* OR schizophreni* OR tourette* OR trichotillomania ) ) \| 203,111 \| \| S12 \| TI ( (aperger* OR autism OR autistic OR adhd ) ) OR AB ( (aperger* OR autism OR autistic OR adhd ) ) OR KW ( (aperger* OR autism OR autistic OR adhd ) ) \| 102,459 \| \| S11 \| TI ( (anorexia OR anorectic OR bulimia OR bulimic OR diabulimia OR orthorexia OR pica ) ) OR AB ( (anorexia OR anorectic OR bulimia OR bulimic OR diabulimia OR orthorexia OR pica ) ) OR KW ( (anorexia OR anorectic OR bulimia OR bulimic OR diabulimia OR orthorexia OR pica ) ) \| 26,363 \| \| S10 \| TI ( ((affective OR alcohol OR adjustment OR amphetamine OR antisocial OR attachment OR "attention deficit" OR behavio#r OR bipolar OR "body dysmorphic" OR "body integrity" OR borderline OR capgras OR cocaine OR cognition OR combat OR communication OR compulsive OR conduct OR conversion OR consciousness OR development* OR dissociative OR dysmorphic OR eating OR elimination OR factitious OR feeding OR gender OR intellectual OR learning OR mental OR mood OR motor OR munchausen OR narcotic# OR neurodevelopmental OR neurotic OR opioid OR panic OR paranoid OR passive-aggressive OR personality OR phobic OR psychiatric OR psychotic OR rumination OR schizoid OR sleep OR somatoform OR speech-sound OR stress* OR substance OR tic# OR tobacco OR trauma ) N2 (disorder# OR disabilit* OR disease# OR diagnos?s OR illness* OR syndrome# ) ) OR AB ( ((affective OR alcohol OR adjustment OR amphetamine OR antisocial OR attachment OR "attention deficit" OR behavio#r OR bipolar OR "body dysmorphic" OR "body integrity" OR borderline OR capgras OR cocaine OR cognition OR combat OR communication OR compulsive OR conduct OR conversion OR consciousness OR development* OR dissociative OR dysmorphic OR eating OR elimination OR factitious OR feeding OR gender OR intellectual OR learning OR mental OR mood OR motor OR munchausen OR narcotic# OR neurodevelopmental OR neurotic OR opioid OR panic OR paranoid OR passive-aggressive OR personality OR phobic OR psychiatric OR psychotic OR rumination OR schizoid OR sleep OR somatoform OR speech-sound OR stress* OR substance OR tic# OR tobacco OR trauma ) N2 (disorder# OR disabilit* OR disease# OR diagnos?s OR illness* OR syndrome# ) ) OR KW ( ((affective OR alcohol OR adjustment OR amphetamine OR antisocial OR attachment OR "attention deficit" OR behavio#r OR bipolar OR "body dysmorphic" OR "body integrity" OR borderline OR capgras OR cocaine OR cognition OR combat OR communication OR compulsive OR conduct OR conversion OR consciousness OR development* OR dissociative OR dysmorphic OR eating OR elimination OR factitious OR feeding OR gender OR intellectual OR learning OR mental OR mood OR motor OR munchausen OR narcotic# OR neurodevelopmental OR neurotic OR opioid OR panic OR paranoid OR passive-aggressive OR personality OR phobic OR psychiatric OR psychotic OR rumination OR schizoid OR sleep OR somatoform OR speech-sound OR stress* OR substance OR tic# OR tobacco OR trauma ) N2 (disorder# OR disabilit* OR disease# OR diagnos?s OR illness* OR syndrome# ) ) \| 564,244 \| \| S9 \| DE "Quality of Life" OR DE "Health Related Quality of Life" \| 73,402 \| \| S8 \| DE "Social Support" OR DE "Perceived Social Support" \| 70,091 \| \| S7 \| DE "Self-Concept" OR DE "Academic Self Concept" OR DE "Athletic Identity" OR DE "Entitlement (Psychological)" OR DE "Impostor Phenomenon" OR DE "Professional Identity" OR DE "Self-Affirmation" OR DE "Self-Compassion" OR DE "Self-Confidence" OR DE "Self-Congruence" OR DE "Self-Esteem" OR DE "Self-Forgiveness" OR DE "Self-Regard" OR DE "Self-Worth" OR DE "Sense of Coherence" \| 91,577 \| \| S6 \| DE "Mental Health" OR DE "Youth Mental Health" \| 110,831 \| \| S5 \| DE "Mental Disorders" OR DE "Affective Disorders" OR DE "Anxiety Disorders" OR DE "Behavior Disorders" OR DE "Bipolar Disorder" OR DE "Borderline States" OR DE "Chronic Mental Illness" OR DE "Dissociative Disorders" OR DE "Eating Disorders" OR DE "Gender Dysphoria" OR DE "Mental Disorders due to General Medical Conditions" OR DE "Neurocognitive Disorders" OR DE "Neurodevelopmental Disorders" OR DE "Neurosis" OR DE "Obsessive Compulsive Disorder" OR DE "Paraphilias" OR DE "Personality Disorders" OR DE "Psychosis" OR DE "Serious Mental Illness" OR DE "Sleep Wake Disorders" OR DE "Somatoform Disorders" OR DE "Stress and Trauma Related Disorders" OR DE "Substance Related and Addictive Disorders" OR DE "Thought Disorders" OR DE "Affective Disorders" OR DE "Disruptive Mood Dysregulation Disorder" OR DE "Major Depression" OR DE "Persistent Depressive Disorder" OR DE "Premenstrual Dysphoric Disorder" OR DE "Seasonal Affective Disorder" OR DE "Anxiety Disorders" OR DE "Castration Anxiety" OR DE "Generalized Anxiety Disorder" OR DE "Panic Attack" OR DE "Panic Disorder" OR DE "Phobias" OR DE "Selective Mutism" OR DE "Separation Anxiety Disorder" OR DE "Behavior Disorders" OR DE "Conduct Disorder" OR DE "Disruptive Behavior Disorders" OR DE "Impulse Control Disorders" OR DE "Kleptomania" OR DE "Oppositional Defiant Disorder" OR DE "Pyromania" OR DE "Self-Destructive Behavior" OR DE "Bipolar Disorder" OR DE "Bipolar I Disorder" OR DE "Bipolar II Disorder" OR DE "Cyclothymic Disorder" OR DE "Mania" OR DE "Chronic Mental Illness" OR DE "Chronic Psychosis" OR DE "Dissociative Disorders" OR DE "Depersonalization" OR DE "Depersonalization/Derealization Disorder" OR DE "Dissociative Amnesia" OR DE "Dissociative Fugue" OR DE "Dissociative Identity Disorder" OR DE "Eating Disorders" OR DE "Anorexia Nervosa" OR DE "Avoidant/Restrictive Food Intake Disorder" OR DE "Binge Eating Disorder" OR DE "Bulimia" OR DE "Feeding Disorders" OR DE "Hyperphagia" OR DE "Kleine Levin Syndrome" OR DE "Orthorexia" OR DE "Pica" OR DE "Purging (Eating Disorders)" OR DE "Rumination (Eating)" OR DE "Neurocognitive Disorders" OR DE "Auditory Processing Disorder" OR DE "Consciousness Disorders" OR DE "Delirium" OR DE "Dementia" OR DE "Memory Disorders" OR DE "Mild Cognitive Impairment" OR DE "Neurodevelopmental Disorders" OR DE "Attention Deficit Disorder" OR DE "Autism Spectrum Disorders" OR DE "Communication Disorders" OR DE "Developmental Disabilities" OR DE "Disruptive Behavior Disorders" OR DE "Dyspraxia" OR DE "Emotional and Behavioral Disorders" OR DE "Intellectual Development Disorder" OR DE "Learning Disorders" OR DE "Stereotypic Movement Disorder" OR DE "Tic Disorders" OR DE "Neurosis" OR DE "Childhood Neurosis" OR DE "Experimental Neurosis" OR DE "Traumatic Neurosis" OR DE "Obsessive Compulsive Disorder" OR DE "Body Dysmorphic Disorder" OR DE "Excoriation Disorder" OR DE "Hoarding Disorder" OR DE "Koro" OR DE "Trichotillomania" OR DE "Paraphilias" OR DE "Exhibitionism" OR DE "Fetishism" OR DE "Pedophilia" OR DE "Sexual Masochism" OR DE "Sexual Sadism" OR DE "Voyeurism" OR DE "Personality Disorders" OR DE "Antisocial Personality Disorder" OR DE "Avoidant Personality Disorder" OR DE "Borderline Personality Disorder" OR DE "Dependent Personality Disorder" OR DE "Histrionic Personality Disorder" OR DE "Narcissistic Personality Disorder" OR DE "Obsessive Compulsive Personality Disorder" OR DE "Paranoid Personality Disorder" OR DE "Passive Aggressive Personality Disorder" OR DE "Sadomasochistic Personality" OR DE "Schizoid Personality Disorder" OR DE "Schizotypal Personality Disorder" OR DE "Psychosis" OR DE "Affective Psychosis" OR DE "Alcohol Induced Psychotic Disorders" OR DE "Brief Psychotic Disorder" OR DE "Capgras Syndrome" OR DE "Childhood Onset Psychosis" OR DE "Chronic Psychosis" OR DE "Delusional Disorder" OR DE "Experimental Psychosis" OR DE "Hallucinosis" OR DE "Paranoid Psychosis" OR DE "Postpartum Psychosis" OR DE "Reactive Psychosis" OR DE "Schizophrenia" OR DE "Substance Induced Psychotic Disorders" OR DE "Sleep Wake Disorders" OR DE "Hypersomnia" OR DE "Insomnia" OR DE "Narcolepsy" OR DE "Parasomnias" OR DE "Sleep Apnea" OR DE "Somatoform Disorders" OR DE "Body Dysmorphic Disorder" OR DE "Conversion Disorder" OR DE "Factitious Disorders" OR DE "Illness Anxiety Disorder" OR DE "Neurasthenia" OR DE "Somatization Disorder" OR DE "Somatoform Pain Disorder" OR DE "Stress and Trauma Related Disorders" OR DE "Acute Stress Disorder" OR DE "Adjustment Disorders" OR DE "Attachment Disorders" OR DE "Disinhibited Social Engagement Disorder" OR DE "Posttraumatic Stress Disorder" OR DE "Prolonged Grief Disorder" OR DE "Substance Related and Addictive Disorders" OR DE "Addiction" OR DE "Nonsubstance Related Addictions" OR DE "Substance Use Disorder" OR DE "Thought Disorders" OR DE "Confabulation" OR DE "Delusions" OR DE "Fantasies (Thought Disturbances)" OR DE "Fragmentation (Schizophrenia)" OR DE "Judgment Disturbances" OR DE "Magical Thinking" OR DE "Memory Disorders" OR DE "Obsessions" OR DE "Perseveration" \| 892,702 \| \| S4 \| S1 OR S2 OR S3 \| 317,598 \| \| S3 \| TI ( ( ("correlated measure*" OR "Cronbach* alpha" OR eigenvalue OR "factor analy*" OR "factor correlation*" OR "factor loading*" OR "internal consistenc*" OR "item-total correlation" OR "Kaiser Criterion" OR "Kaiser-Guttman rule" OR "oblique rotation" OR "orthogonal rotation" OR "scree plot*" ) ) OR AB ( ( ("correlated measure*" OR "Cronbach* alpha" OR eigenvalue OR "factor analy*" OR "factor correlation*" OR "factor loading*" OR "internal consistenc*" OR "item-total correlation" OR "Kaiser Criterion" OR "Kaiser-Guttman rule" OR "oblique rotation" OR "orthogonal rotation" OR "scree plot*" ) ) OR KW ( ( ("correlated measure*" OR "Cronbach* alpha" OR eigenvalue OR "factor analy*" OR "factor correlation*" OR "factor loading*" OR "internal consistenc*" OR "item-total correlation" OR "Kaiser Criterion" OR "Kaiser-Guttman rule" OR "oblique rotation" OR "orthogonal rotation" OR "scree plot*" ) ) \| 107,214 \| \| S2 \| DE "Statistical Reliability" OR DE "Statistical Validity" OR DE "Statistical Correlation" \| 23,500 \| \| S1 \| DE "Psychometrics" OR DE "Classical Test Theory" OR DE "Consistency (Measurement)" OR DE "Error of Measurement" OR DE "External Validity" OR DE "Factor Analysis" OR DE "Internal Validity" OR DE "Item Analysis (Test)" OR DE "Item Response Theory" OR DE "Measurement Invariance" OR DE "Measurement Models" OR DE "Multivariate Analysis" OR DE "Test Construction" OR DE "Test Reliability" OR DE "Test Sensitivity" OR DE "Test Specificity" OR DE "Test Validity" OR DE "Variability Measurement" OR DE "Factor Analysis" OR DE "Confirmatory Factor Analysis" OR DE "Exploratory Factor Analysis" OR DE "Factor Structure" OR DE "Item Analysis (Statistical)" OR DE "Statistical Rotation" OR DE "Item Analysis (Test)" OR DE "Differential Item Functioning" OR DE "Measurement Models" OR DE "Mixture Modeling" OR DE "Structural Equation Modeling" OR DE "Multivariate Analysis" OR DE "Factor Analysis" OR DE "Mixture Modeling" OR DE "Multiple Regression" OR DE "Path Analysis" OR DE "Principal Component Analysis" OR DE "Test Reliability" OR DE "Internal Consistency" OR DE "Interrater Reliability" OR DE "Split-Half Reliability" OR DE "Test-Retest Reliability" OR DE "Test Validity" OR DE "Clinical Validity" OR DE "Construct Validity" OR DE "Content Validity" OR DE "Criterion Validity" OR DE "Face Validity" OR DE "Factorial Validity" \| 269,645 \| | |

4.Search strategy for the CINAHL

| Interface: Ebsco  Date of Search: 20 February 2025  Number of hits: 5,719 | Field labels   - MH+ = exploded Cinahl Heading - MH = non exploded Cinahl Heading - TI = title - AB = abstract - Nx = within x words, regardless of order - * = truncation of word for alternate endings |
| --- | --- |
| \| **#** \| **Query** \| **Results** \| \| --- \| --- \| --- \| \| S31 \| S4 AND S19 AND S26 AND S29  Limiters - Publication Date: 20000101-20241231 \| 5,719 \| \| S30 \| S4 AND S19 AND S26 AND S29 \| 5,973 \| \| S29 \| S27 OR S28 \| 2,178,558 \| \| S28 \| TI ( (construct# OR dimension# OR item# OR screen* OR scale* OR test* OR questionnaire* OR survey* OR tool# ) ) OR AB ( (construct# OR dimension# OR item# OR screen* OR scale* OR test* OR questionnaire* OR survey* OR tool# ) ) \| 1,707,600 \| \| S27 \| (MH "Psychological Tests+") OR (MH "Surveys+") OR (MH "Questionnaires+") OR (MH "Scales") OR (MH "Health Screening") OR (MH "Diagnostic Tests, Routine") \| 1,141,231 \| \| S26 \| S20 OR S21 OR S22 OR S23 OR S24 OR S25 \| 221,080 \| \| S25 \| TI ( (africa* OR angola* OR benin* OR botswan* OR "burkina faso*" OR "cabo verde*" OR cameroon* OR "central african republic" OR chad OR congo* OR "cote d'ivoire" OR "democratic republic of the congo" OR djibouti* OR "equatorial guinea*" OR eritrea* OR eswatini* OR ethiopia* OR gabon* OR gambia* OR ghan* OR guinea* OR guinea-bissau OR kenya* OR lesotho* OR liberia* OR malawi* OR mali OR mauritania* OR mozambique* OR namibia* OR niger* OR nigeria OR rwanda* OR "sao tome and principe" OR senegal* OR "sierra leone" OR somali* OR "south africa" OR "south sudan" OR sudan* OR tanzania* OR togo OR uganda* OR zambia* OR zimbabwe* ) ) OR AB ( (africa* OR angola* OR benin* OR botswan* OR "burkina faso*" OR "cabo verde*" OR cameroon* OR "central african republic" OR chad OR congo* OR "cote d'ivoire" OR "democratic republic of the congo" OR djibouti* OR "equatorial guinea*" OR eritrea* OR eswatini* OR ethiopia* OR gabon* OR gambia* OR ghan* OR guinea* OR guinea-bissau OR kenya* OR lesotho* OR liberia* OR malawi* OR mali OR mauritania* OR mozambique* OR namibia* OR niger* OR nigeria OR rwanda* OR "sao tome and principe" OR senegal* OR "sierra leone" OR somali* OR "south africa" OR "south sudan" OR sudan* OR tanzania* OR togo OR uganda* OR zambia* OR zimbabwe* ) ) \| 147,039 \| \| S24 \| TI ( ("Global South" OR LIC OR LMIC* OR MIC OR South-South ) ) OR AB ( ("Global South" OR LIC OR LMIC* OR MIC OR South-South ) ) \| 10,735 \| \| S23 \| TI ( ((low* OR middle* ) N3 (countr* OR nation* )) ) OR AB ( ((low* OR middle* ) N3 (countr* OR nation* )) ) \| 24,798 \| \| S22 \| TI ( (low* N2 (countr* OR gdp OR gnp OR "gross domestic" OR "gross national" )) ) OR AB ( (low* N2 (countr* OR gdp OR gnp OR "gross domestic" OR "gross national" )) ) \| 8,441 \| \| S21 \| TI ( ((deprived OR developing OR emerging OR "less* developed" OR "low* income" OR "middle income" OR poor* OR third-world OR "under developed" OR "under served" OR underdeveloped OR underserved ) N2 (countr* OR economies OR economy OR nation# OR population# OR world )) ) OR AB ( ((deprived OR developing OR emerging OR "less* developed" OR "low* income" OR "middle income" OR poor* OR third-world OR "under developed" OR "under served" OR underdeveloped OR underserved ) N2 (countr* OR economies OR economy OR nation# OR population# OR world )) ) \| 49,225 \| \| S20 \| (MH "Africa South of the Sahara+") OR (MH "Developing Countries") OR (MH "Low and Middle Income Countries") \| 107,722 \| \| S19 \| S5 OR S6 OR S7 OR S8 OR S9 OR S10 OR S11 OR S12 OR S13 OR S14 OR S15 OR S16 OR S17 OR S18 \| 1,348,361 \| \| S18 \| TI "quality of life" OR AB "quality of life" \| 162,597 \| \| S17 \| TI ( ((social OR psychosocial OR psychological OR family OR community ) N2 support ) ) OR AB ( ((social OR psychosocial OR psychological OR family OR community ) N2 support ) ) \| 65,196 \| \| S16 \| TI ( (self W1 (concept* OR confiden* OR efficac* OR esteem* OR perception* )) ) OR AB ( (self W1 (concept* OR confiden* OR efficac* OR esteem* OR perception* )) ) \| 54,793 \| \| S15 \| TI ( (depress* OR "involutional paraphrenia#" OR "involutional psychos*" OR "dysthymic disorder#" OR dysthymia OR "premenstrual dysphoric" OR "seasonal mood disorder#" OR "seasonal affective disorder#" ) ) OR AB ( (depress* OR "involutional paraphrenia#" OR "involutional psychos*" OR "dysthymic disorder#" OR dysthymia OR "premenstrual dysphoric" OR "seasonal mood disorder#" OR "seasonal affective disorder#" ) ) \| 189,834 \| \| S14 \| TI ( ((alcohol OR amphetamine# OR "angel dust" OR cannabis OR cocaine OR drug# OR glue OR hashish OR heroin OR inhalant OR marihuana OR marijuana OR narcotic* OR nicotine OR opiate# OR opioid# OR opium OR pcp OR phencyclidine# OR substance# ) N2 (abuse OR abusing OR dependen* OR misus* OR problem# OR use* )) ) OR AB ( ((alcohol OR amphetamine# OR "angel dust" OR cannabis OR cocaine OR drug# OR glue OR hashish OR heroin OR inhalant OR marihuana OR marijuana OR narcotic* OR nicotine OR opiate# OR opioid# OR opium OR pcp OR phencyclidine# OR substance# ) N2 (abuse OR abusing OR dependen* OR misus* OR problem# OR use* )) ) \| 160,946 \| \| S13 \| TI ( (addict* OR alcoholism ) ) OR AB ( (addict* OR alcoholism ) ) \| 35,461 \| \| S12 \| TI ( (anxiet* OR phobia# OR agrophobia# OR kinesiophobia# OR "panic attack#" ) ) OR AB ( (anxiet* OR phobia# OR agrophobia# OR kinesiophobia# OR "panic attack#" ) ) \| 113,346 \| \| S11 \| TI ( ((mental* OR psychiatric OR psychologic* ) N2 (well* OR health* )) ) OR AB ( ((mental* OR psychiatric OR psychologic* ) N2 (well* OR health* )) ) \| 170,381 \| \| S10 \| TI ( (post-traumatic OR ((psychological OR sexual OR military ) N2 trauma* )) ) OR AB ( (post-traumatic OR ((psychological OR sexual OR military ) N2 trauma* )) ) \| 15,856 \| \| S9 \| TI ( (encopresis OR enuresis OR gambl* OR "gender dysphoria" OR hypochondria* OR neurasthen* OR parasomnia* OR psychos?s OR pyromani* OR schizophreni* OR tourette* OR trichotillomania ) ) OR AB ( (encopresis OR enuresis OR gambl* OR "gender dysphoria" OR hypochondria* OR neurasthen* OR parasomnia* OR psychos?s OR pyromani* OR schizophreni* OR tourette* OR trichotillomania ) ) \| 52,330 \| \| S8 \| TI ( (aperger* OR autism OR autistic OR adhd ) ) OR AB ( (aperger* OR autism OR autistic OR adhd ) ) \| 47,832 \| \| S7 \| TI ( (anorexia OR anorectic OR bulimia OR bulimic OR diabulimia OR orthorexia OR pica ) OR AB ( (anorexia OR anorectic OR bulimia OR bulimic OR diabulimia OR orthorexia OR pica ) \| 12,399 \| \| S6 \| TI ( ((affective OR alcohol OR adjustment OR amphetamine OR antisocial OR attachment OR "attention deficit" OR behavio#r OR bipolar OR "body dysmorphic" OR "body integrity" OR borderline OR capgras OR cocaine OR cognition OR combat OR communication OR compulsive OR conduct OR conversion OR consciousness OR development* OR dissociative OR dysmorphic OR eating OR elimination OR factitious OR feeding OR gender OR intellectual OR learning OR mental OR mood OR motor OR munchausen OR narcotic# OR neurodevelopmental OR neurotic OR opioid OR panic OR paranoid OR passive-aggressive OR personality OR phobic OR psychiatric OR psychotic OR rumination OR schizoid OR sleep OR somatoform OR speech-sound OR stress* OR substance OR tic# OR tobacco OR trauma ) N2 (disorder# OR disabilit* OR disease# OR diagnos?s OR illness* OR syndrome# )) ) OR AB ( ((affective OR alcohol OR adjustment OR amphetamine OR antisocial OR attachment OR "attention deficit" OR behavio#r OR bipolar OR "body dysmorphic" OR "body integrity" OR borderline OR capgras OR cocaine OR cognition OR combat OR communication OR compulsive OR conduct OR conversion OR consciousness OR development* OR dissociative OR dysmorphic OR eating OR elimination OR factitious OR feeding OR gender OR intellectual OR learning OR mental OR mood OR motor OR munchausen OR narcotic# OR neurodevelopmental OR neurotic OR opioid OR panic OR paranoid OR passive-aggressive OR personality OR phobic OR psychiatric OR psychotic OR rumination OR schizoid OR sleep OR somatoform OR speech-sound OR stress* OR substance OR tic# OR tobacco OR trauma ) N2 (disorder# OR disabilit* OR disease# OR diagnos?s OR illness* OR syndrome# )) ) \| 246,859 \| \| S5 \| (MH "Mental Disorders+") OR (MH "Mental Health") OR (MH "Self Concept+") OR (MH "Support, Social+") OR (MH "Quality of Life") \| 960,216 \| \| S4 \| S1 OR S2 OR S3 \| 806,164 \| \| S3 \| TI ( ("correlated measure*" OR "Cronbach* alpha" OR eigenvalue OR "factor analy*" OR "factor correlation*" OR "factor loading*" OR "internal consistenc*" OR "item-total correlation" OR "Kaiser Criterion" OR "Kaiser-Guttman rule" OR "oblique rotation" OR "orthogonal rotation" OR "scree plot*" ) ) OR AB ( ("correlated measure*" OR "Cronbach* alpha" OR eigenvalue OR "factor analy*" OR "factor correlation*" OR "factor loading*" OR "internal consistenc*" OR "item-total correlation" OR "Kaiser Criterion" OR "Kaiser-Guttman rule" OR "oblique rotation" OR "orthogonal rotation" OR "scree plot*" ) ) \| 46,207 \| \| S2 \| TI ( (psychometric* OR reliability OR validated OR validity OR validation ) ) OR AB ( (psychometric* OR reliability OR validated OR validity OR validation ) ) \| 251,618 \| \| S1 \| (MH "Psychometrics") OR (MH "Reliability and Validity+") OR (MH "Factor Analysis+") OR (MH "Validation Studies") OR (MH "Reproducibility of Results") \| 673,305 \| | |
