# Supplementary material for: A systematic review and meta-analysis of diagnostic test accuracy of mental health screening tools applicable to adolescents in sub-Saharan Africa
Source: Front Psychiatry. 2026 Jun 16;17:1728252. doi: 10.3389/fpsyt.2026.1728252 (PMC13317013; doi:10.3389/fpsyt.2026.1728252)
Supplement: Supplementary file 2 [file Table2.docx]

**Supplementary appendix 2:** All included articles and mental health screening tools validated

| **Article #** | **Author, years (Reference #)** | **Title** | **Country** | **Name of the tool(s)**  **(abbreviation(s)** | **Condition(s)** | **Target population** | **Age range (years)** | **Sample size** | **Language of the tool** | **Internal consistency** | **Cut-off point** | **Prevalence per reference/gold standard** | **Actual positive** | **Sensitivity** | **Specificity** | **Positive predictive value** | **Negative predictive value** | **AUC** |
| --- | --- | --- | --- | --- | --- | --- | --- | --- | --- | --- | --- | --- | --- | --- | --- | --- | --- | --- |
| 1 | Humeniuk, 2008(98)* | Validation of the alcohol, smoking and substance involvement screening test (ASSIST) | Zimbabwe | ASSIST-TSI - use / abuse | 1. Substance abuse / dependence | In-patients and out- patients from:1) primary health care centers and 2) drug treatment centers | 18 - 45  years | 150 | NR | 1. The ASSIST-TSI: 0.89 | 14.5 | NR | NR | 0.80 | 0.71 | NR | NR | 0.84 |
|  |  |  |  | ASSIST-TSI - Abuse / dependence |  |  |  |  |  |  | 28.5 | NR | NR | 0.73 | 0.66 | NR | NR | 0.73 |
|  |  |  |  | ASSIST-SSI for Alcohol - use / dependence | 2. Alcohol abuse/dependence |  |  |  |  | 2. The ASSIST-TSI- alcohol: 0.84 | 5.5 | NR | NR | 0.83 | 0.79 | NR | NR | 0.87 |
|  |  |  |  | ASSIST-SSI for Alcohol - abuse / dependence |  |  |  |  |  |  | 10.5 | NR | NR | 0.67 | 0.60 | NR | NR | 0.70 |
|  |  |  |  | ASSIST-SSI for cannabis - use / abuse | 3. cannabis abuse/dependence |  |  |  |  | 3. The ASSIST-TSI-cannabis:0.86 | 1.5 | NR | NR | 0.91 | 0.90 | NR | NR | 0.96 |
|  |  |  |  | ASSIST-SSI for cannabis - abuse / dependence |  |  |  |  |  |  | 10.5 | NR | NR | 0.57 | 0.61 | NR | NR | 0.62 |
|  |  |  |  | ASSIST-SSI for cocaine - use / abuse | 4. cocaine abuse/dependence |  |  |  |  | 4. The ASSIST-TSI-cocaine:0.93 | 0.5 | NR | NR | 0.92 | 0.94 | NR | NR | 0.95 |
|  |  |  |  | ASSIST-SSI for cocaine - abuse / dependence |  |  |  |  |  |  | 8.5 | NR | NR | 0.70 | 0.77 | NR | NR | 0.84 |
|  |  |  |  | ASSIST-SSI for amphetamine - use / abuse | 5. amphetamine-type stimulants abuse/dependence |  |  |  |  | 5. The ASSIST-TSI-ATS:0.94 | 0.5 | NR | NR | 0.97 | 0.87 | NR | NR | 0.96 |
|  |  |  |  | ASSIST-SSI for amphetamine - abuse / dependence |  |  |  |  |  |  | 11.5 | NR | NR | 0.72 | 0.68 | NR | NR | 0.77 |
|  |  |  |  | ASSIST-SSI for sedatives - use / abuse | 6. sedatives abuse/dependence |  |  |  |  | 2.7. The ASSIST-TSI-sedative: 0.89 | 0.5 | NR | NR | 0.94 | 0.91 | NR | NR | 0.96 |
|  |  |  |  | ASSIST-SSI for sedatives - abuse / dependence |  |  |  |  |  |  | 10.5 | NR | NR | 0.54 | 0.50 | NR | NR | 0.45 |
|  |  |  |  | ASSIST-SSI for opioids - use / abuse | 7. opioids abuse/dependence |  |  |  |  | 2.7. The ASSIST-TSI-opioids: 0.94 | 0.5 | NR | NR | 0.94 | 0.96 | NR | NR | 0.97 |
|  |  |  |  | ASSIST-SSI for opioids - abuse / dependence |  |  |  |  |  |  | 14.5 | NR | NR | 0.76 | 0.65 | NR | NR | 0.74 |
|  |  |  |  | ASSIST-SSI for Cocaine compared with presence in hair | Cocaine use |  |  |  |  | NR | NR | NR | NR | 0.82 | 0.91 | NR | NR | NR |
|  |  |  |  | ASSIST-SSI (SSI) for amphetamine -type stimulants (ATS) compared with presence in hair | Amphetamine -type stimulants (ATS) use |  |  |  |  |  |  | NR | NR | 0.66 | 0.73 | NR | NR | NR |
|  |  |  |  | ASSIST-SSI for Benzodiazepines compared with presence in hair |  |  |  |  |  |  |  | NR | NR | 0.73 | 0.75 | NR | NR | NR |
|  |  |  |  | ASSIST-SSI for Opioids compared with presence in hair | Opoids use |  |  |  |  |  |  | NR | NR | 0.91 | 0.8 | NR | NR | NR |
| 2 | Seth, 2015(118) | AUDIT, AUDIT-C, and AUDIT-3: Drinking patterns and screening for harmful, hazardous and dependent drinking in Katutura, Namibia | Namibia | AUDIT-C (Overall) | Harmful drinking (Harmful alcohol use, hazardous alcohol use and alcohol dependence) | General population | 18-80 years | 639 | English, Oshiwambo, or Afrikaans | NR | 3 | NR | NR | 0.95 (0.92- 0.99) | 0.78 (0.73-0.83) | NR | NR | 0.96 ( 0.94- 0.97) |
|  |  |  |  | The AUDIT-C (Men) |  |  |  |  |  |  |  | NR | NR | 0.99(0.98-1.00) | 0.78 (0.68-0.87) | NR | NR | 0.97 (0.95-0.99) |
|  |  |  |  | The AUDIT-C (Women) |  |  |  |  |  |  |  | NR | NR | 0.92 (0.86-0.98) | 0.77 (0.72- 0.83) | NR | NR | 0.95 ( 0.93-0.97) |
|  |  |  |  | The AUDIT-3 (Overall) |  |  |  |  |  | NR | 1 | NR | NR | 0.85(0.80-0.91) | 0.34 (0.28- 0.40) | NR | NR | 0.87 (0.84- 0.90) |
|  |  |  |  | The AUDIT-3 (Men) |  |  |  |  |  |  |  | NR | NR | 0.86 (0.78-0.94) | 0.29 ( 0.18- 0.41) | NR | NR | 0.85(0.79-0.90) |
|  |  |  |  | The AUDIT-3 (Women) |  |  |  |  |  |  |  | NR | NR | 0.85 (0.77-0.93) | 0.35 (0.29-0.42) | NR | NR | 0.87 (0.83-0.91) |
| 3 | Stockton, 2023(99) | Psychometric evaluation of the psychosis screening questionnaire in South Africa with attention to overlap between symptoms and normative cultural beliefs | South Africa | PSQ-Current Severe Mental Disorder | Current Severe Mental Disorder | Patients and accompaniers at primary and tertiary health facilities | 18 - 88 years | 1885 | isiXhosa or English | 0.755 | NR | 8.1% | 152 | 0.60 (0.52-0.68) | 0.68 (0.66-0.70) | NR | NR | 0.64 ( 0.60- 0.68) |
|  |  |  |  | PSQ-Current Psychotic Disorder | Current Psychotic Disorder |  |  |  |  |  |  | 5% | 95 | 0.54(0.43-0.64) | 0.67 (0.64-0.69) | NR | NR | 0.60 (0.55-0.66) |
|  |  |  |  | PSQ-Current Hypomanic or Manic Episode | Current Hypomanic or Manic Episode |  |  |  |  |  |  | 3.6% | 67 | 0.69(0.56-0.79) | 0.67 (0.65-0. 69) | NR | NR | 0.68 ( 0.62- 0.73) |
|  |  |  |  | PSQ-Lifetime Severe Mental Disorder | Lifetime Severe Mental Disorder |  |  |  |  |  |  | 11.6% | 218 | 0.65 ( 0.58-0.71) | 0.70 (0.67- 0.72) | NR | NR | 0.67 (0.64-0.70) |
|  |  |  |  | PSQ-Lifetime Psychotic Disorder | Lifetime Psychotic Disorder |  |  |  |  |  |  | 7% | 131 | 0.55 ( 0.46-0.64) | 0.67( 0.65-0.69) | NR | NR | 0.61 ( 0.57-0.65) |
|  |  |  |  | PSQ-Lifetime Hypomanic or Manic Episode | Lifetime Hypomanic or Manic Episode |  |  |  |  |  |  | 6.2% | 117 | 0.74 (0.65-0.82) | 0.68 (0.66-0.70) | NR | NR | 0.71 (0.67-0.75) |
| 4 | Miafo, 2024(40) | Validation of the Edinburgh postnatal depression scale and prevalence of depression among adolescent mothers in a Cameroonian context | Cameroon | EPDS | Perinatal depression | Adolescent mothers | 12-20 years | 1633 | French | NR | 11 | 60.9% | 994 | 0.93 (0.91-0.94) | 0.53 (0.51-0.56) | 0.76 | 0.80 | 0.85 |
| 5 | Awadu, 2021(120) | Validation of Autism Screening Assessments: Comparison of the Social Communication Questionnaire, Social Responsiveness Scale and 23Q with DSM-5 in assessing for Autism Spectrum Disorder (ASD) in Uganda | Uganda | SCQ | Autism spectrum disorder | Children aged 4-18 years | 4 - 17 years | 105  105  107 | English | 1. ASD sample: 0.61, 2. Non-ASD sample: 0.72 1.3. Combined sample: 0.93 | 15 | NR | 49 | 0.96 | 0.95 | NR | NR | NR |
|  |  |  |  | SRS-2 |  |  |  |  |  | 1. ASD sample: 0.84, 2. Non-ASD sample: 0.89 and 3. Combined sample: 0.97 | 62 | NR | 50 | 1 | 0.96 | NR | NR | NR |
|  |  |  |  | 23Q |  |  |  |  |  | 1. ASD sample: 0.60, 2. Non-ASD sample: 0.83 and 3 Combined sample: 0.65 | 3 | NR | 51 | 0.77 | 0.68 | NR | NR | NR |
| 6 | Atuhaire, 2023(47) | Validating the Edinburgh Postnatal Depression Scale Against the Diagnostic and Statistical Manual of Mental Disorders, 5th Edition for Use in Uganda | Uganda | EPDS | Postpartum depression (PPD) | Postpartum women | 18-49 years | 278 | Runyankore-Rukiga | NR | 10 | 26.5% | 76 | 0.87(0.77-0.94) | 0.92 (0.88-0.95) | 0.805 | 0.949 | 1. 0.89 for Bwizibwera HCIV 2. 0.97 for Kinoni HCIV  3. 0.84 for MRRH |
| 7 | Stockton, 2023(125) | An Ultra-Brief Proxy Measure for Early Mental and Substance Use Disorders and Suicide Risk Case Detection at the Community and Household Level: An Efficient and Feasible Clinical and Population-level Service Needs Screening Tool | Mozambique South Africa | Proxy mwTool-3 in Portuguese, isiXhosa or English version | 1. Severe mental disorder (SMD - manic episode, hypomanic episode, psychotic disorder) 2. Common mental disorder (CMD - major depressive episode, posttraumatic stress disorder, general anxiety disorder) 3. Alcohol use disorder (AUD - alcohol abuse or dependence) 4. Substance use disorder (SUD - substance abuse or dependence 5. Suicide risk (SR - moderate to high suicide risk) | Adults (patients and accompaniers) | 18-78 years | 458 | Portuguese, isiXhosa or English | NR | Several days or more to any of the 3 questions | 35.6% | NR | 0.73 (0.66-0.80) | 0.46 ( 0.40- 0.52) | NR | NR | NR |
|  |  |  | Mozambique | Proxy mwTool-3 in Portuguese version |  |  |  | 218 | Portuguese |  | Several days or more to any of the 3 questions | 38.5% | NR | 0.70(0.59- 0.80) | 0.43 (0.35- 0.52) |  |  |  |
|  |  |  | South Africa | Proxy mwTool-3 in isiXhosa or English version |  |  |  | 240 | isiXhosa or English |  | Several days or more to any of the 3 questions | 32.9% | NR | 0.80 ( 0.69-0.88) | 0.48 ( 0.41- 0.56) |  |  |  |
|  |  |  |  | Proxy mwTool-3 in isiXhosa version |  |  |  |  | isiXhosa |  | Several days or more to any of the 3 questions | 32.9% | NR | 0.75 (0.63-0.84) | 0.49 (0.41-0.58) |  |  |  |
| 8 | Vissoci, 2023(126) | Clinical evaluation of the alcohol use disorders identification test (AUDIT) in Moshi, Tanzania | Tanzania | AUDIT | Alcohol use disorders | Adults | 18 years and above | 259 | English or Swahili | 0.84 | 8 | 51.3% | NR | 0.77 | 0.78 | 0.66 | 0.86 | 0.86 ( 0.813-0.907) |
|  |  |  |  | AUDIT-3 |  |  |  |  |  | NA | 1 | 51.3% | NR | 0.64 | 0.83 | 0.67 | 0.81 | 0.76 (0.70–0.81) |
|  |  |  |  | AUDIT-4 |  |  |  |  |  | 0.68 | 5 | 51.3% | NR | 0.81 | 0.67 | 0.58 | 0.87 | 0.82(0.773–0.87) |
|  |  |  |  | AUDIT-5 |  |  |  |  |  | 0.7 | 5 | 51.3% | NR | 0.66 | 0.85 | 0.71 | 0.82 | 0.828 (0.78–0.88) |
|  |  |  |  | AUDIT-C |  |  |  |  |  | 0.71 | 5 | 51.3% | NR | 0.57 | 0.85 | 0.59 | 0.84 | 0.82(0.77–0.8) |
|  |  |  |  | AUDIT-PC |  |  |  |  |  | 0.7 | 5 | 51.3% | NR | 0.82 | 0.67 | 0.58 | 0.87 | 0.83(0.78–0.88) |
| 9 | Gebreegziabhere, 2024(127)* | Development and Evaluation of a Cognitive Battery for People With Schizophrenia in Ethiopia | Ethiopia | ECAS-WLLT | Cognitive impairment | Schizophrenia outpatients | 18-65 years | 416 | Amharic | 0.81 | ≤ 16 | NR | NR | 0.58 (0.51-0.64) | 0.82 (0.76-0.87) | NR | NR | 0.76 ( 0.72-0.80) |
|  |  |  |  | ECAS-DST | Cognitive impairment -Digit Sequencing Task - verbal working memory |  |  |  |  |  | ≤ 7 | NR | NR | 0.75 (0.68-0.81) | 0.57 ( 0.50-0.64) |  |  | 0.69 ( 0.64-0.73) |
|  |  |  |  | ECAS-CBTT | Cognitive impairment - visual working momery |  |  |  |  |  | ≤ 30 | NR | NR | 0.42 (0.36-0.49) | 0.77 ( 0.71- 0.82) |  |  | 0.61 (0.56-0.66) |
|  |  |  |  | ECAS-ANT | Cognitive impairment - Animal Naming Test-Verbal learning |  |  |  |  |  | ≤ 16 | NR | NR | 0.66 (0.60- 0.73) | 0.60 ( 0.53-0.66) |  |  | 0.68 (0.63- 0.72) |
|  |  |  |  | ECAS-DSST | Cognitive impairment -Digit Symbol Substitution Test |  |  |  |  |  | >539 | NR | NR | 0.51(0.44-0.58) | 0.90 ( 0.85-0.94) |  |  | 0.78 (0.74-0.82) |
|  |  |  |  | ECAS-TMT A & SP | Cognitive impairment -[attention] and SP [Speed of Processing] (Trail Making Test) |  |  |  |  |  | >65 | NR | NR | 0.76 (0.70- 0.82) | 0.55 ( 0.48-0.62) |  |  | 0.70 (0.65-0.74) |
|  |  |  |  | ECAS-TMT B (EF) | Cognitive impairment-Executive Function |  |  |  |  |  | >127 | NR | NR | 0.88 (0.82-0.92) | 0.48 ( 0.41-0.55) |  |  | 0.73 (0.69-0.76) |
|  |  |  |  | Overall ECAS | Cognitive impairment - Overall |  |  |  |  |  | ≤ 0.12 | NR | NR | 0.77 (0.92-0.82) | 0.62 (0.55-0.69) |  |  | 0.78 (0.74-0.82) |
| 10 | Kwobah, 2024(100) | Adaptation of the Client Diagnostic Questionnaire for East Africa | 1. Kenya 2. Uganda | CDQ | 1. Major depressive disorder 2. Alcohol abuse 3. Panic disorder 4. Anxiety 5. Drug abuse 6. PTSD 7.Psychosis | People living with HIV (PLHIV) | 18 years and above | 90 | 1. Swahili 2. Dholuo 3. Runyankole-Rukiga | NR | NR | NR | 45 | 0.87 | 0.64 | 0.71 | 0.83 | NR |
| 11 | Lovero, 2024(122) | Psychometric Evaluation of Screens for Common Mental Disorders, Severe Mental Disorders, Substance Use Disorders, and Suicide Risk in Mozambican Healthcare | Mozambique | PSQ-Psychosis | Psychosis | Mozambican adults  in general healthcare settings | 18 years and above | 911 | Portuguese | Psychosis: 0.78 | NR | 26% | 235 | 0.58 (0.51-0.64) | 0.79 (0.76-0.82) | NR | NR | NR |
|  |  |  |  | PSQ-Mania | Mania |  |  |  |  | Mania: 0.78 |  | 8% | 70 | 0.73(0.63-0.83) | 0.73 (0.70-0.76) |  |  |  |
| 12 | Musoni-Rwililiza, 2024(128) | Adaption and validation of the Rwandese version of the Mood Disorder Questionnaire for the screening of bipolar disorder | Rwanda | R-MDQ | Bipolar disorder | Outpatients diagnosed with either bipolar disorder or unipolar major depression | 18 years and above | 331 | Kinyarwanda | 0.91 | 6 | NR | 135 | 0.95 | 0.97 | NR | NR | 0.99 |
| 13 | Nalugya, 2024(129) | Improving alcohol and substance use screening in school-age children: translation, adaptation and psychometric evaluation of the CRAFFT tool for Lumasaaba, Uganda | Uganda | CRAFFT - Overall (Boys and Girls) | Substance use disorders (SUD) | Primary school aged children | 6-13 years | 460 | Lumasaaba | 0.86 | 1 | 7.2% | 32 | 0.91 | 0.92 | 0.48 | 0.9 | 0.91(0.86-0.97) |
|  |  |  |  | CRAFFT - Boys |  |  |  |  |  |  |  | NR | NR | 0.87 | 0.95 | 0.59 | 0.99 | 0.91 |
|  |  |  |  | CRAFFT - Girls |  |  |  |  |  |  |  | NR | NR | 0.94 | 0.90 | 0.41 | 0.95 | 0.92 |
| 14 | Nwokolo, 2024(130) | Validation of the social communication questionnaire amongst Nigerian adolescents | Nigeria | The Social Communication Questionnaire (SCQ) | Autism spectrum disorder | Adolescents suspected of having ASD | 11-26 years | 204 | English | 0.88 | 10 | NR | 69 | 0.81 | 0.88 | 0.75 | 0.91 | 0.83 (0.77-0.90) |
| 15 | Stockton, 2024(101) | Validation of a brief screener for broad-spectrum mental and substance-use disorders in South Africa | South Africa | mwTool-13 for any disorder sub-scale | Any disorder (CDM, AUD, SUD, SMD or SR) | Patients and their accompaniers at health facilities | 18-88 years | 1885 | isiXhosa or English | NR | NR | 36% |  | 0.83 ( 0.80-0.86) | 0.46 ( 0.43-0.49 ) | NR | NR | NR |
|  |  |  |  | mwTool-13 for CDM sub-scale 1 (with question of unspecified disorder) | CDM |  |  |  |  |  |  | 24% |  | 0.91 (0.89-0.94) | 0.45 (0.42- 0.47) |  |  |  |
|  |  |  |  | mwTool-13 for CDM sub-scale2 (without question of unspecified disorder) | CDM |  |  |  |  |  |  | 24% |  | 0.89 (0.86-0.92) | 0.48( 0.45-0.50) |  |  |  |
|  |  |  |  | mwTool-13 for MDE sub-scale | Major Depressive Disorder |  |  |  |  |  |  | 21% |  | 0.91(0.88-0.94) | 0.47 ( 0.44-0.50) |  |  |  |
|  |  |  |  | mwTool-13 for GAD sub-scale | General Anxiety Disorder |  |  |  |  |  |  | 6% |  | 0.90 (0.83-0.95) | 0.41 ( 0.39-0.43) |  |  |  |
|  |  |  |  | mwTool-13 for PTSD sub-scale | Post-traumatic stress disorder |  |  |  |  |  |  | 9% |  | 0.90(0.84-0.94) | 0.42 (0.39-0.44) |  |  |  |
|  |  |  |  | mwTool-13 for AUD sub-scale | Alcohol Used Disorder) |  |  |  |  |  |  | 10% |  | 0.57(0.49-0.64) | 0.85 (0.83-0.86) |  |  |  |
|  |  |  |  | mwTool-13 for SUD sub-scale | Substance User Disorder) |  |  |  |  |  |  | 2% |  | 0.65 (0.45-0.81) | 0.93 ( 0.92-0.95) |  |  |  |
|  |  |  |  | mwTool-13 for SMD sub-scale | Sever Mental Disorder |  |  |  |  |  |  | 8% |  | 0.72 (0.64- 0.79) | 0. 62(0.60- 0.64) |  |  |  |
|  |  |  |  | mwTool-13 for PD sub-scale | Psychiatric Disorder |  |  |  |  |  |  | 5% |  | 0.67 ( 0.57-0.77) | 0.61 ( 0.58-0.63) |  |  |  |
|  |  |  |  | mwTool-13 for M/HE sub-scale | Manic or Hypomanic Episode |  |  |  |  |  |  | 4% |  | 0.78 ( 0.66-0.87) | 0.61(0.58-0.63) |  |  |  |
|  |  |  |  | mwTool-13 for SR sub-scale | Suicide Risk |  |  |  |  |  |  | 6% |  | 0.85 (0.79-0.92) | 0.80 (0.78-0.82) |  |  |  |
| 16 | Stockton, 2024(59) | Validation of screening instruments for common mental disorders and suicide risk in south African primary care settings | South Africa | PHQ-2 | Depression | Patients or accompanies at health facilities | 18-88 years | 1885 | isiXhosa or English | NR | >0 | 22.2% | 420 | 0.94 (0.92-0.96) | 0.43 (0.41 -0.46) | NR | NR | 0.80 ( 0.78- 0.82) |
|  |  |  |  | PHQ-9 (cut off of 5) |  |  |  |  |  | 0.89 | 5 | 22.2% | 420 | 0.84 (0.80- 0.88) | 0.67 (0.65-0.70) | NR | NR | NR |
|  |  |  |  | PHQ-9 (cut off of 10) |  |  |  |  |  |  | 10 |  |  | 0.49 (0.44-0.54) | 0.90(0.89-0.92) | NR | NR | 0.83 (0.81-0.86) |
|  |  |  |  | PHQ-2/9 (cut off of 5) |  |  |  |  |  |  | 5 | 22.2% | 420 | 0.84(0.80- 0.87) | 0.70 (0.67-0.72) | NR | NR | NR |
|  |  |  |  | PHQ-2/9 (cut off of 10) |  |  |  |  |  |  | 10 |  |  | 0.49 (0.44-0.54) | 0.91 (0.89-0.92) | NR | NR | 0.84 (0.82-0.86) |
|  |  |  |  | PHQ-4 for depression |  |  |  |  |  | 0.81 | NR | 22.2% | 420 | 0.96 (0.94-0.98) | 0.37 (0.35-0.40) | NR | NR | 0.82 (0.80-0.84) |
|  |  |  |  | PHQ-4 for Anxiety | Anxiety |  |  |  |  |  | NR | 6.4% | 121 | 0.95 (0.90-0.98) | 0.32 (0.30-0.34) | NR | NR | 0.76 (0.73-0.80) |
|  |  |  |  | PHQ-4 for anxiety or panic | Anxiety or panic |  |  |  |  |  | NR | 7.1% | 135 | 0.95 ( 0.90-0.98) | 0.32 (0.30-0.34) | NR | NR | 0.77 (0.73-0.81) |
|  |  |  |  | PHQ-4 for anxiety or depression | Anxiety or depression |  |  |  |  |  | NR | 28.6% | 541 | 0.96 (0.93-0.97) | 0.38 (0.35-0.40) | NR | NR | 0.81 (0.79-0.83) |
|  |  |  |  | PHQ-4 for PTSD | PTSD |  |  |  |  |  | NR | 8.6% | 163 | 0.95 (0.93- 0.97) | 0.38(0.36-0.41) | NR | NR | 0.77 (0.74-0.80) |
|  |  |  |  | PHQ-4 for any CMD | Any Common mental disorder |  |  |  |  |  | > 0 | 24.4% | 459 | 0.96 ( 0.94-0.98) | 0.37(0.35-0.40) | NR | NR | 0.80 (0.78-0.83) |
|  |  |  |  | PHQ-Q9 Low risk of SRi | Low risk for suicide risk |  |  |  |  | 0.892 | NR | 8.8% | 165 | 0.63 (0.57-0.69) | 0.87 (0.85-0.89) | NR | NR | 0.76 (0.73-0.79) |
|  |  |  |  | PHQ-Q9 High risk of SRi | High risk for suicide risk |  |  |  |  |  | >0 | 4.3% | 81 | 0.81 (0.72-0.87) | 0.83 (0.82-0.85) | NR | NR | 0.83 (0.79-0.87) |
|  |  |  |  | GAD-2 for Anxiety | Anxiety |  |  |  |  | NR | >0 | 6.4% | 121 | 0.87 ( 0.79-0.92) | 0.45 (0.43-0.48) | NR | NR | 0.77 (0.74-0.79) |
|  |  |  |  | GAD-7 for Anxiety for cut off of 5 |  |  |  |  |  | 0.865 | 5 | 6.4% | 121 | 0.72( 0.63-0.80) | 0.71 (0.69-0.73) | NR | NR | NR |
|  |  |  |  | GAD-7 for Anxiety for cut off of 10 |  |  |  |  |  |  | 10 | 6.4% | 121 | 0.25 ( 0.17- 0.33) | 0.93(0.92-0.94) | NR | NR | 0.78 (0.74-0.81) |
|  |  |  |  | GAD-2/7 for Anxiety (cut off of 5) |  |  |  |  |  |  | 5 | 6.4% | 121 | 0.68 (0.59-0.76) | 0.72 (0.70-0.74) | NR | NR | NR |
|  |  |  |  | GAD-2/7 for Anxiety (cut off of 10) |  |  |  |  |  |  | 10 |  | 121 | 0.24 (0.17-0.33) | 0.93 (0.92-0.95) | NR | NR | 0.74 |
|  |  |  |  | GAD-2 for Anxiety or panic | Anxiety or panic |  |  |  |  |  | > 0 | 7.1% | 135 | 0.88 (0.81-0.93) | 0.45 (0.43-0.48) | NR | NR | 0.72 (0.68-0.76) |
|  |  |  |  | GAD-7 for Anxiety or panic (cut off of 5) |  |  |  |  |  |  | 5 | 7.1% | 135 | 0.74 (0.65-0.81) | 0.71 (0.69-0.73) | NR | NR | NR |
|  |  |  |  | GAD-7 for Anxiety or panic (cut off of 10) |  |  |  |  |  |  | 10 | 7.1% | 135 | 0.26 (0.18-0.34) | 0.93 (0.92-0.94) | NR | NR | 0.79 (0.75-0.82) |
|  |  |  |  | GAD-2/7 for Anxiety or panic (Cut off of 5) |  |  |  |  |  |  | 5 | 7.1% | 135 | 0.70 (0.61-0.78) | 0.7215 (0.6999-0.7424) | NR | NR | NR |
|  |  |  |  | GAD-2/7 for Anxiety or panic (Cut off of 10) |  |  |  |  |  |  | 10 | 7.1% | 135 | 0.25(0.18-0.33) | 0.94 (0.92-0.95) | NR | NR | 0.76 (0.71-0.80) |
|  |  |  |  | PC-PTSD-5 | PTSD |  |  |  |  | 0.827 | 4 | 8.6% | 163 | 0.62 (0.54-0.69) | 0.82(0.80–0.84) | NR | NR | 0.76 (0.72-0.80) |
|  |  |  |  | C-SSRS for Low Risk SRi | Low risk for suicide risk |  |  |  |  | NR | NA | 8.8% | 165 | 0.56 (0.50-0.61) | 0.91 (0.89-0.92) | NR | NR | NR |
|  |  |  |  | C-SSRS for High Risk Sri | High risk for suicide risk |  |  |  |  |  |  | 4.3% | 81 | 0.54 (0.45-0.64) | 0.96 (0.95-0.97) | NR | NR | NR |
| 17 | Kaaya, 2002(131) | Validity of the Hopkins Symptom Checklist-25 amongst HIV-positive pregnant women in Tanzania | Tanzania | HSCL-25 | 1.Anxiety 2.Depression | HIV-positive pregnant women attending antenatal clinics | 15 years and above | 97 | Kiswahili | 1.HSCL-25: 0.93 | 1.06 | 3.3% | NR | 0.89 | 0.80 | NR | NR | 0.86 (0.72- 0.99) |
|  |  |  | Tanzania | HSCL-15 |  |  |  |  |  | 2.HSCL-15: 0.90 | 1.03 | 7.7% | NR | 0.89 | 0.79 | NR | NR | 0.86 (0.73-0.99) |
|  |  |  | Tanzania | HSCL-8 |  |  |  |  |  | 3.HSCL-8: 0.85 | 1.06 | 1.1% | NR | 0.89 | 0.85 | NR | NR | 0.88 (0.75-1.00) |
| 18 | Uwakwe, 2003(57) | Affective (depressive) morbidity in puerperal Nigerian women: validation of the Edinburgh Postnatal Depression Scale | Nigeria | EPDS | Depression | Postpartum women | 18-39 years | 225 | 1. English 2. Igbo (local language) | 0.83 | 9 | 10.7% | 24 | 0.75 | 0.97 | 0.75 | 0.97 | NR |
| 19 | Adewuya, 2006(102) | Validation of the alcohol use disorders identification test (audit) as a screening tool for alcohol-related problems among Nigerian university students | Nigeria | AUDIT - Alcohol hazardous use | Alcohol hazardous use | University students | Mean (SD): 22.5 (5.2) | 248 | NR | NR | 5 | NR | 76 | 0.94 | 0.92 | 1.AUDIT- Hazardous use: 0.893 | 0.948 | 0.93( 0.90-0.97) |
|  |  |  |  | AUDIT - Alcohol harmful use | Alcohol harmful use |  |  |  |  |  | 7 | NR | 25 | 0.9 | 0.86 | 0.47 | 0.98 | 0.95 (0.90-0.99) |
|  |  |  |  | AUDIT - Alcohol dependence | Alcohol dependence |  |  |  |  |  | 9 | NR | 6 | 1 | 0.94 | 0.2 | 1 | 0.99(0.96-1.00) |
| 20 | Adewuya, 2006(65) | Validity of the patient health questionnaire (PHQ-9) as a screening tool for depression amongst Nigerian university students | Nigeria | PHQ-9 (Minor DD) | Minor depressive disorder | University students | 15 to 40 years | 512 | English | 0.85 | 5 | NR | 26 | 0.90 | 0.99 | 0.88 | 0.98 | 0.99(0.98-1.00) |
|  |  |  | Nigeria | PHQ-9 MDD | Major depressive disorder |  |  |  |  |  | 10 | NR | 13 | 0.84 | 0.99 | 0.75 | 0.10 | 1.00(0.99-1.00) |
| 21 | Adewuya, 2006(56) | Validation of the Edinburgh Postnatal Depression Scale as a screening tool for depression in late pregnancy among Nigerian women | Nigeria | EPDS - Major and minor depression | Major and minor depression | Women in late pregnancy (32 weeks and above) | 24.89 (SD= 6.41) | 86 | 1. English 2. Yoruba language | 0.85 | 10 | NR | 15 | 0.87 | 0.92 | 0.68 | 0.97 | 0.97 (0.93-1.00) |
|  |  |  |  | EPDS - Major depression only | Major depression only |  |  |  |  |  | 12 | NR | 9 | 1 | 0.96 | 0.75 | 1 | 1.00(0.98- 1.00) |
| 22 | Adewuya, 200(45) | Prevalence of major depressive disorders and a validation of the Beck Depression Inventory among Nigerian adolescents | Nigeria | BDI | Major depressive disorder | Secondary school students | 13-18 years | 1095 | NR | 0.82 | 18 | 6.9% | 76 | 0.91 | 0.97 | 0.88 | 0.98 | 0.99(0.98-1.00) |
| 23 | Baggaley, 2007(46) | Detecting depression after pregnancy: the validity of the K10 and K6 in Burkina Faso | Burkina Faso | K10 | Postnatal depression | Postpartum women | 17-46 years | 61 | 1. West African French  2. Moore 3. Dioula | 1. K10: 0.87 | 14 | 44% | 27 | 0.59 | 0.91 | NR | NR | 0.77 |
|  |  |  | Burkina Faso | K6 |  |  |  |  |  | 2. K6: 0.78 | 9 | 44% | 27 | 0.59 | 0.85 |  |  | 0.75 |
| 24 | Hanlon, 2008(132) | Detecting perinatal common mental disorders in Ethiopia: validation of the self-reporting questionnaire and Edinburgh Postnatal Depression Scale | Ethiopia | EPDS | CMD | 1. postnatal women | 15 years and above | Study 1: 52 | Amharic | 1. EPDS=0.47 | 5/6 | 40% | NR | 0.77 | 0.36 | NR | NR | 0.62 (0.49-0.76) |
|  |  |  |  | SRQ-20 - Study 1 |  | 2. Perinatal women |  |  |  | 2.1. Study 1=0.84 | 2/3 | 40% | NR | 0.86 | 0.76 |  |  | 0.82 (0.68-0.96) |
|  |  |  |  | SRQ-20 - Study 2 |  |  |  | Study 2: 119 |  | 2.2. study 2=0.88 | 6/7 | 27.7% | NR | 0.68 | 0.62 |  |  | 0.70 (0.57-0.83) |
| 25 | Jordans, 2008(133) | Development and validation of the child psychosocial distress screener in Burundi | Burundi | CPDS | Psychosocial distress | Children | 7 - 17 years | 65 | Kirundi | 0.53 | 8 | 55% | 36 | 0.84 | 0.6 | 0.68 | 0.8 | 0.81 |
| 26 | Myer, 2008(134) | Common mental disorders among HIV-infected individuals in South Africa: prevalence, predictors, and validation of brief psychiatric rating scales | South Africa | CES-D - Afrikaans, Xhosa and English | Depression | HIV-infected individuals | 18 - 65 years | 465 | 1.Afrikaans, 2.Xhosa, 3. English | NR | NR | 14% | 63 | 0.79 (0.76-0.83) | 0.61 (0.56-0.85) | 0.61 | 0.4 | 0.7553 |
|  |  |  |  | HTQ - Afrikaans, Xhosa and English | Post-traumatic stress disorder (PTSD) |  |  |  |  |  | 75 | 5% | 24 | 0.38 (0.33-0.42) | 0.8 (0.76-0.83) | 0.44 | 0.57 | 0.7367 |
|  |  |  |  | AUDIT - Afrikaans, Xhosa and English | Alcohol dependence/abuse |  |  |  |  |  | NR | 7% | 35 | 1.00 (0.75-0.83) | 0.79 (0.75-0.83) | 0.62 | 0.36 | 0.9563 |
|  |  |  |  | CES-D - Afrikaans | Depression |  |  |  |  |  |  | 14% | 63 | 0.86 | 0.76 | 0.6 | 0.93 | NR |
|  |  |  |  | HTQ - Afrikaans | Post-traumatic stress disorder (PTSD) |  |  |  |  |  |  | 5% | 24 | 0.47 | 0.87 | 0.38 | 0.91 |  |
|  |  |  |  | AUDIT - Afrikaans | Alcohol dependence/abuse |  |  |  |  |  |  | 7% | 35 | 1 | 0.80 | 0.55 | NR |  |
|  |  |  |  | CES-D - Xhosa | Depression |  |  |  |  |  |  | 14% | 63 | 0.73 | 0.56 | 0.13 | 0.96 |  |
|  |  |  |  | HTQ - Xhosa | Post-traumatic stress disorder (PTSD) |  |  |  |  |  |  | 5% | 24 | 0.00 | 0.77 | 0.00 | 0.98 |  |
|  |  |  |  | AUDIT - Xhosa | Alcohol dependence/abuse |  |  |  |  |  |  | 7% | 35 | 1 | 0.78 | 0.13 | NR |  |
| 27 | Monahan, 2009(103) | Validity/reliability of PHQ-9 and PHQ-2 depression scales among adults living with HIV/AIDS in western Kenya | Kenya | PHQ-2 - MDD | Major depressive disorder (MDD) | Adults living with HIV/AIDS | 18-61 years | 347 | English or Swahili | 1. PHQ-9: 0.78 | 3 | 13% | 45 | 0.91 | 0.77 | 0.37 | NR | 0.91 ( 0.88-0.95) |
|  |  |  |  | PHQ-2 - ADD | 2. Any depressive disorder (ADD) |  |  |  |  |  |  | 34% | 118 | 0.85 | 0.95 | 0.90 |  | 0.97 (0.95-0.98) |
| 28 | Spies, 2009(77) | Validity of the Kessler 10 (K-10) in detecting DSM-IV defined mood and anxiety disorders among pregnant women | South Africa | K-10 - Current MDE | Current major depressive disorder | pregnant women | 15.5 - 43.0 years | 129 | English or Afrikaans |  | 21.5 | 12.2% | 16 | 0.73 | 0.54 | 0.18 | 0.94 | 0.66 |
|  |  |  |  | K-10 - Past MDE | Past major depressive disorder |  |  |  |  |  | 23.5 | 13.7% | 18 | 0.53 | 0.63 | 0.19 | 0.9 | 0.58 |
|  |  |  |  | K-10 - Past bipolar disorder | Past bipolar disorder |  |  |  |  |  | 21.5 | 0.8% | 1 | 1 | 0.52 | 0.016 | 1 | 0.55 |
|  |  |  |  | K-10 -Past dysthymic disorder | Past dysthymic disorder |  |  |  |  |  | 33.5 | 0.8% | 1 | 1 | 0.93 | 0.103 | 1 | 0.93 |
|  |  |  |  | K-10 - Current panic disorder | Current panic disorder |  |  |  |  |  | 38.5 | 1.5% | 2 | 0.5 | 0.98 | 0.28 | 0.99 | 0.71 |
|  |  |  |  | K-10 - Past panic disorder | Past panic disorder |  |  |  |  |  | 21.5 | 4.6% | 6 | 0.83 | 0.53 | 0.08 | 0.98 | 0.68 |
|  |  |  |  | K-10 - Social anxiety disorder | Social anxiety disorder |  |  |  |  |  | 26.5 | 0.8% | 1 | 1 | 0.75 | 0.26 | 1 | 0.76 |
|  |  |  |  | K-10 - Current PTSD | Current PTSD (Post Traumatic Stress Disorder) |  |  |  |  |  | 28.5 | 3.1% | 4 | 0.5 | 0.8 | 0.07 | 0.98 | 0.69 |
|  |  |  |  | K-10 - Past PTSD | Past PTSD (Post Traumatic Stress Disorder) |  |  |  |  |  | 21.5 | 4.6% | 6 | 0.67 | 0.52 | 0.06 | 0.97 | 0.57 |
| 29 | Stewart, 2009(135)* | Validation of a Chichewa version of the self-reporting questionnaire (SRQ) as a brief screening measure for maternal depressive disorder in Malawi, Africa | Malawi | SRQ-cMajor DD | Current Major Depressive Disorder | Mothers/infants attending a child health clinic | 16-18 years | 114 | Chichewa | 0.85 | 7/8 | NR | NR | 0.66 | 0.78 | 0.33 | 0.93 | 0.86(0.81-0.90) |
|  |  |  |  | SRQ-cMinor and cMajor DD | Current Minor and Major Depressive Disorder |  |  |  |  |  | 7/8 | NR | NR | 0.59 | 0.85 | 0.64 | 0.83 | 0.83(0.78-0.87) |
| 30 | Weobong, 2008(58) | The comparative validity of screening scales for postnatal common mental disorder in Kintampo, Ghana | Ghana | SRQ-20 | Psychological distress | Women within postnatal period 5 and 11 weeks | 15-46 years | 160 | Twi | 0.78 | 6/7 | 11.3% | 18 | 0.77 | 0.63 | 0.19 | 0.96 | 0.74 ( 0.62-0.86) |
|  |  |  |  | EPDS | depression, anxiety and panic and thoughts of self-harm |  |  |  |  | 0.79 | 10/11 | 11.3% | 18 | 0.78 | 0.73 | 0.22 | 0.97 | 0.84 (0.76-0.92) |
|  |  |  |  | PHQ-9 | Depression |  |  |  |  | 0.79 | 4/5 | 11.3% | 18 | 0.94 | 0.75 | 0.26 | 0.99 | 0.90 (0.81-0.98) |
| 31 | Chibanda, 2010(48) | Validation of the Edinburgh Postnatal Depression Scale among women in a high HIV prevalence area in urban Zimbabwe | Zimbabwe | EPDS | Major depression | Postpartum HIV-infected and uninfected women | 18 years and older | 210 | Shona | 0.87 | 12 | 33% | 64 | 0.88 | 0.87 | 0.74 | 0.94 | 0.82 |
| 32 | Chipimo, 2010(97) | Comparative validity of screening instruments for mental distress in Zambia | Zambia | SRQ-20 | CMD:Depression and anxiety disorders | Primary health care clinic attendees | 16 - 67 years | 400 | English or Nyanja or Bemba | NR | 7 | 12.6% | NR | 0.85 | 0.94 | 0.68 | 0.97 | 0.96 |
|  |  |  |  | SRQ-10 | Mental distress |  |  |  |  |  |  | 13.6% | NR | 0.81 | 0.95 | 0.71 | 0.97 | 0.95 |
|  |  |  |  | GHQ-12 | Mental distress |  |  |  |  |  | 2 | 13.6% | NR | 0.66 | 0.86 | 0.43 | 0.94 | 0.81 |
| 33 | Spies, 2010(78) | Validity of the K-10 in detecting DSM-IV-defined depression and anxiety disorders among HIV-infected individuals: Corrigendum | South Africa | K-10 (Current MDE) | Current MDE | HIV-infected adults | 16-65 years | 429 | 1. Xhosa 2.Africaans 3. English | 0.87 | ≤ 28 | 53.12% | NR | 0.67 | 0.77 | 0.29 | 0.94 | 0.77 |
|  |  |  |  | K-10 (Past MDE) | Past MDE |  |  |  |  |  | ≤28 | 28.7% | NR | 0.77 | 0.75 | 0.17 | 0.97 | 0.75 |
|  |  |  |  | K-10 (Mood disorders) | Mood disorders |  |  |  |  |  | ≤ 30 | 11.3% | NR | 0.9 | 0.79 | 0.1 | 0.99 | 0.84 |
|  |  |  |  | K-10 (Agoraphobia) | Agoraphobia |  |  |  |  |  | ≤ 26 | 18.4% | NR | 0.65 | 0.67 | 0.08 | 0.97 | 0.69 |
|  |  |  |  | K-10 (GAD) | GAD |  |  |  |  |  | ≤ 30 | 18.4% | NR | 0.72 | 0.8 | 0.14 | 0.99 | 0.78 |
|  |  |  |  | K-10 (Social Phobia) | Social Phobia |  |  |  |  |  | ≤ 30 | 12.3% | NR | 0.92 | 0.8 | 0.12 | 0.99 | 0.9 |
|  |  |  |  | K-10 (Panic disorder) | Panic disorder |  |  |  |  |  | ≤ 28 | 15.3% | NR | 0.76 | 0.74 | 0.1 | 0.99 | 0.77 |
|  |  |  |  | K-10 (PTSD) | PTSD |  |  |  |  |  | ≤ 29 | 21.5% | NR | 0.75 | 0.78 | 0.15 | 0.99 | 0.77 |
| 34 | Tesfaye, 2010(136) | Detecting postnatal common mental disorders in Addis Ababa, Ethiopia: validation of the Edinburgh Postnatal Depression Scale and Kessler Scales | Ethiopia | EPDS | Postnatal common mental disorders | Postnatal women attending vaccination clinics | 18-38 years | 100 | Amharic | 1. EPDS: 0.71 | 6/7 | 19% | 19 | 0.79 | 0.75 | 0.43 | 0.94 | 0.85 (0.77-0.92) |
|  |  |  | Ethiopia | K6 |  |  |  |  |  | 2. K6: 0.86 | 4/5 | 19% | 19 | 0.84 | 0.83 | 0.53 | 0.96 | 0.86 (0.76-0.97) |
|  |  |  | Ethiopia | K10 |  |  |  |  |  | 3. K10: 0.90 | 6/7 | 19% | 19 | 0.84 | 0.78 | 0.47 | 0.96 | 0.87 (0.78-0.97) |
| 35 | Andersen, 2011(136) | The psychometric properties of the K10 and K6 scales in screening for mood and anxiety disorders in the South African Stress and Health study | South Africa | K10 | 1. Depression disorder  2. Anxiety disorder | General population | 18 - 92 years | 4077 | 1. Afrikaans, 2. Zulu, 3. Xhosa,  4. Northern Sotho 5. Tswana | 1. For K10: 0.84 | 16 | 11.9% | NR | 0.7 | 0.67 | 0.23 | NR | 0.73 |
|  |  |  |  | K6 |  |  |  |  |  | 2. For K6: 0.48 | 10 | 11.9% | NR | 0.7 | 0.62 | 0.21 |  | 0.72 |
| 36 | Chishinga, 2011(104)* | Validation of brief screening tools for depressive and alcohol use disorders among TB and HIV patients in primary care in Zambia | Zambia | CES-D | 1. Current Major depressive disorder (MDD) | TB and anti-retroviral therapy (ART) patients | 16 years and older | 649 | Bemba, Nyanja, Tonga or Lozi | 1. The CES-D: 0.84 | 22 | 62 | NR | 0.73 | NR | 0.76 | NR | 0.78 (0.72-0.84) |
|  |  |  |  | AUDIT - Women | 2. Alcohol use disorders (AUDs) |  |  |  |  | 2. The AUDIT: 0.98 | 24 | NR | NR | 0.6 |  | 0.60 |  | 0.98 (0.94-0.98) |
|  |  |  |  | AUDIT - men |  |  |  |  |  |  | 24 | NR | NR | 0.55 |  | 0.5 |  | 0.75 (0.66-0.84) |
| 37 | Ertl, 2011(137) | Validation of a mental health assessment in an African conflict population | Uganda | PDS | 1. Posttraumatic stress disorder (PTSD) | war-affected adolescents and young adults | 12-25 years | 68 | Luo | 1. For PDS: 0.89 | 16 | 42.6% | NR | 0.82 | 0.76 | NR | NR | 0.79 |
|  |  |  | Uganda | DHSCL | 2. Depression |  |  |  |  | 2. For DHSCL: 0.89 | 2.65 | 23.5% | NR | 0.5 | 0.83 | NR | NR | 0.76 |
| 38 | Lowenthal, 2011(105) | Validation of the Pediatric Symptom Checklist in HIV-infected Batswana | Botswana | PSC | Psychosocial health | HIV positive children | 8-16 years | 509 | 1. English or 2. Setswana | 1. PSC: 0.87 | 20 | 3.5% | NR | 0.62 (0.58-0.66) | 0.86 (0.83-0.89) | 0.36 | 0.95 | 0.85 |
|  |  |  |  | PSC-17 |  |  |  |  |  | 2. PSC-17: 0.80 | 10 | 3.5% | NR | 0.6 (0.56-0.64) | 0.88 (0.85-0.91) | 0.92 | 0.95 | 0.84 |
|  |  |  |  | PSC-Y |  |  |  |  |  | 3.PSC-Y: 0.86 | 20 | NR | NR | 0.64 (0.6-0.68) | 0.88 (0.85-0.91) | 0.31 | 0.97 | 0.81 |
|  |  |  |  | PSC-17-Y |  |  |  |  |  | 4.PSC-17-Y: 0.78 | 9 | NR | NR | 0.67(0.63-0.71) | 0.90 (0.87-0.93) | 0.89 | 0.96 | 0.82 |
| 39 | Murray, 2011(123)* | Validation of the UCLA Child Post traumatic stress disorder-reaction index in Zambia | Zambia | PTSD-RI SS | Traumatic stress | Youth experienced sexual abuse | 6-15 years | 352 | Nyanja | 1. PTSD-RI symptom scale: 0.93 | 17 | NR | NR | 0.66 | 0.79 | NR | NR | 0.74 ( 0.62-0.86 ) |
|  |  |  |  | PTSD-RI LSSS |  |  |  |  |  | 2. Locally-specific symptom scale: 0.94 | 10 | NR | NR | 0.68 | 0.69 |  |  | 0.70 (0.57-0.82) |
|  |  |  |  | PTSD-RI – TSS |  |  |  |  |  | 3. Total symptom scale: NR | 31 | NR | NR | 0.66 | 0.76 |  |  | 0.73 (0.60-0.85) |
| 40 | Scholte, 2011(138)* | Psychometric properties and longitudinal validation of the self-reporting questionnaire (SRQ-20) in a Rwandan community setting: a validation study | Rwanda | SRQ-20 - Overall | Mental disorder | Community members | 16-74 years | 99 | Kinyarwanda | 1. SRQ-2 (Overall participants): | 10 | NR | NR | 0.69 | 0.79 | 0.86 | 0.57 | 0.76 (0.66-0.86) |
|  |  |  |  | SRQ-20 - Men |  |  |  |  |  | 2. SRQ-2 (Men): 0.81 | 8 | NR | NR | 0.69 | 0.65 | 0.83 | 0.47 | 0.74 (0.59-0.90) |
|  |  |  |  | SRQ-20 - Women |  |  |  |  |  | 3. SRQ-2 (Women):0.85 | 10 | NR | NR | 0.81 | 0.8 | 0.92 | 0.62 | 0.76 (0.62-0.91) |
| 41 | Akena, 2012(139) | Sensitivity and specificity of a visual depression screening instrument among HIV-positive individuals in Uganda, an area with low literacy | Uganda | AVIDI | Depression | People living with HIV | 18-71 years | 92 | Not applicable (it is a visual tool) | NR | 10 | 18% | 17 | 0.75 | 0.71 | 0.5 | 0.83 | 0.82 |
|  |  |  |  | AVIDI-4 |  |  |  |  |  |  | 6 | 18% | 17 | 0.86 | 0.55 | 0.5 | 0.87 | 0.8 |
| 42 | Betancourt, 2012(34)* | Validating the Center for Epidemiological Studies Depression Scale for Children in Rwanda | Rwanda | CES-DC | Depression | Children and adolescents | 10-17 years | 367 | Kinyarwanda | 0.86 | 30 | NR | NR | 0.82 | 0.72 | NR | NR | 0.83 |
| 43 | Nakimuli-Mpungu, 2012(140) | Cross-cultural adaptation and validation of the self-reporting questionnaire among HIV+ individuals in a rural ART program in southern Uganda | Uganda | SRQ-20 Current depression | 1. Current Depression 2. Any depression | HIV+ individuals | 18 years and over | 200 | Luganda | 1. SRQ- Depression: 0.78 | 6 | 12% | 24 | 0.84 | 0.93 | 0.59 | 0.96 | 0.95 |
|  |  |  |  | SRQ-20 Any depression |  |  |  |  |  | 2. SRQ- Anxiety: 0.72 |  | 24% | 48 | 0.75 | 0.90 | 0.71 | 0.92 | 0.75 |
| 44 | Tunde-Ayinmode, 2012(106) | A comparison of two screening instruments in detecting psychiatric morbidity in a Nigerian pediatric primary care service: assessing clinical suitability and applicability | Nigeria | CBQ | Psychiatric disorders | Children aged 7-14 years and their accompanying mothers attending our hospital. | 7-14 years | 350 | 1. The CBQ: English or Yoruba | NR | 7 | 11.4% | 40 | 0.8 | 0.96 | 0.87 | 0.93 | 0.93 |
|  |  |  |  | RQC |  |  |  |  | 2. The RQC: English or Yoruba |  | 1 | 11.4% | 40 | 0.9 | 0.78 | 0.58 | 0.96 | 0.88 |
| 45 | Akena, 2013(66) | Sensitivity and specificity of clinician administered screening instruments in detecting depression among HIV-positive individuals in Uganda | Uganda | K-10 | 1. Depression  2. Anxiety | HIV patients | 18 years and older | 368 | NR | NR | 23 | 17.4% | NR | 0.83 | 0.72 | NR | NR | 0.82 (0.72-0.93) |
|  |  |  |  | K-2 |  |  |  |  |  |  | 13 | 17.4% | NR | 0.78 | 0.67 |  |  | 0.81 (0.71-0.93) |
|  |  |  |  | PHQ-9 |  |  |  |  |  |  | 10 | 17.4% | NR | 0.92 | 0.81 |  |  | 0.964 (0.92-0.99) |
|  |  |  |  | PHQ-2 |  |  |  |  |  |  | 3 | 17.4% | NR | 0.83 | 0.71 |  |  | 0.82 (0.71-0.93) |
|  |  |  |  | CES-D |  |  |  |  |  |  | 18 | 17.4% | NR | 0.89 | 0.81 |  |  | 0.94 (0.89-0.99) |
| 46 | Gelaye, 2013(61) | Validity of the Patient Health Questionnaire-9 for depression screening and diagnosis in East Africa | Ethiopia | PHQ-9 | Major Depressive Disorder (MDD) | Adult outpatients | 18-69 years | 926 | Amharic | 0.85 | 10 | NR | 46 | 0.86(0.78-0.92) | 0.67 (0.61-0.73) | 0.48 | 0.93 | 0.77 (0.68-0.85) |
| 47 | Gelaye, 2013(141) | Diagnostic validity of the composite international diagnostic interview (CIDI) depression module in an East African population | Ethiopia | CIDI | Major Depressive Disorder (MDD) | Adult outpatients | 18 to 69 years | 926 | Amharic | 0.97 | NR | NR | 46 | 0.44 (0.29-0.59) | 0.78 (0.73-0.82) | 0.22 | 0.91 | NR |
| 48 | Mbewe, 2013(124)* | A primary healthcare screening tool to identify depression and anxiety disorders among people with epilepsy in Zambia | Zambia | PHST – Depression and Anxiety | 1. Depression 2. Anxiety | People living with epilepsy (PWE) | 18 - 50 years | 575 | 1. Nyanja 2. Bemba | 1. For Depression (10 items): 0.77  2. Anxiety (5 items):0.57 | 18 | NR | NR | 0.57 | 0.68 | 0.67 | 0.58 | NR |
| 49 | Rochat, 2013(55) | Detection of antenatal depression in rural HIV-affected populations with short and ultrashort versions of the Edinburgh Postnatal Depression Scale (EPDS) | South Africa | EPDS-10 | Antenatal depression | Pregnant women in their second trimester who live in the catchment area | 16-40 years | 109 | Zulu | 1. EPDS-10: 0.6130 | ≥ 13 | 47% | 51 | 0.69 | 0.78 | 0.73 | 0.74 | 0.82(0.73-0.89) |
|  |  |  | South Africa | EPDS-7 |  |  |  |  |  | 2. The EPDS-7: 0.70 | ≥ 13 | 47% | 51 | 0.61 | 0.90 | 0.84 | 0.72 | 0.83 (0.75-0.90) |
|  |  |  | South Africa | EPDS-5R |  |  |  |  |  | 3. The EPDS-5R: 0.75 | ≥ 13 | 47% | 51 | 0.651 | 0.86 | 0.80 | 0.73 | 0.84 (0.77-0.91) |
|  |  |  | South Africa | EPDS-3R |  |  |  |  |  | 4. The EPDS-3R: 0.61 | ≥ 13 | 47% | 51 | 0.61 | 0.90 | 0.84 | 0.72 | 0.84(0.76 - 0.91) |
| 50 | Vythilingum, 2013(107) | Screening and pathways to maternal mental health care in a South African antenatal setting | South Africa | EPDS Item 8 | Experiencing psychological distress (Depression or anxiety disorders) | Pregnant women during their first or second antenatal visit | 18 years and above | 308 | 1. English 2. Afrikaans 3. Xhosa 4. French | NR | NR | NR | 58 | 0.78 | 0.50 | NR | NR | 0.64 |
|  |  |  |  | RFA Item |  |  |  |  |  |  |  | NR | 58 | 0.35 | 0.84 |  |  | 0.59 |
|  |  |  |  | RFA Item 11 |  |  |  |  |  |  |  | NR | 58 | 0.71 | 0.68 |  |  | 0.69 |
|  |  |  |  | PRI-3items (EPDS8,RFA1&11) |  |  |  |  |  |  | 3 | NR | 58 | 0.66 | 0.75 |  |  | 0.75 |
| 51 | Haney, 2014(142) | One size does not fit all: psychometric properties of the Shona Symptom Questionnaire (SSQ) among adolescents and young adults in Zimbabwe | Zimbabwe | SSQ - adolescents | Depression | Adolescents | 15-19 years | 2768 | Shona | 0.79 | 5 | 8.1% | NR | 0.89 | 0.94 | 0.45 | 0.99 | 0.83 |
| 52 | Kim, 2014(33) | Prevalence of depression and validation of the Beck Depression Inventory-II and the Children's Depression Inventory-Short amongst HIV-positive adolescents in Malawi | Malawi | BDI-II | Depression | HIV-positive adolescents | 12-18 years | 562 | Chichewa | 1. BDI-II: 0.80 | 15 | 18.9% | 106 | 0.78 | 0.77 | 0.43 | 0.93 | 0.82 ( 0.78-0.89 ) |
|  |  |  |  | CDI-II-S |  |  |  |  |  | 2. CDI-II-S: 0.66 | 53 | 18.9% | 106 | 0.69 | 0.67 | 0.32 | 0.90 | 0.75 (0.70-0.80) |
| 53 | Makanjuola, 2014(108)* | Validation of short screening tools for common mental disorders in Nigerian general practices | Nigeria | K6 - Depression | Depression | Hospital outpatients | 18 years and above | 1590 | Yoruba, Igbo and Hausa languages | 1.The K6: 0.929 | 4 | NR | NR | 0.696 | 0.55 | 0.10 | 0.963 | 0.62 |
|  |  |  |  | K6 - Anxiety | Anxiety |  |  |  |  |  | 4 | NR | NR | 0.65 | 0.55 | 0.12 | 0.94 | 0.58 |
|  |  |  |  | GHQ12- Depresion | Depression |  |  |  |  | 2.The GHQ12: 0.70 | 4 | NR | NR | 0.72 | 0.75 | 0.16 | 0.98 | 0.74 |
|  |  |  |  | GHQ12 - Anxiety | Anxiety |  |  |  |  |  | 4 | NR | NR | 0.59 | 0.75 | NR | NR | 0.6 |
| 54 | Ng, 2014(35) | Developing and validating the Youth Conduct Problems Scale-Rwanda: a mixed methods approach | Rwanda | YCPS-R (Youth) | Conduct disorders (CD) | Youth and caregivers | 10-17 years | 389 | Kinyarwanda | 1. YCPS-R (Youth):0.90 | 13 | 19.95% | NR | 0.57 | 0.85 | NR | NR | 0.75 (0.68-0.82) |
|  |  |  |  | YCPS-R (Female caregivers) |  |  |  |  |  | 2. YCPS-R (Caregivers): 0.94 | 9 | 10.39% | NR | 0.75 | 0.73 |  |  | 0.78 (0.65-0.90) |
|  |  |  |  | YCPS-R (Male caregivers) |  |  |  |  |  |  | 16 | 27.54% | NR | 0.54 | 0.66 |  |  | 0.62 (0.54-0.71) |
|  |  |  |  | YCPS-R SF (Youth) |  |  |  |  |  | 3. YCPS-R SF (Youth): 0.89 | 5 | 19.95% | NR | 0.77 | 0.63 |  |  | 0.75 (0.69-0.82) |
|  |  |  |  | YCPS-R SF (Female caregivers) |  |  |  |  |  | 4. YCPS-R SF (Caregivers): 0.93 | 9 | 10.39% | NR | 0.69 | 0.80 |  |  | 0.79 (0.68-0.91) |
|  |  |  |  | YCPS-R SF (Male caregivers) |  |  |  |  |  |  | 14 | 27.54% | NR | 0.49 | 0.72 |  |  | 0.63 (0.54-0.71) |
| 55 | Tsai, 2014(143) | Antenatal depression case finding by community health workers in South Africa: feasibility of a mobile phone application | South Africa  South Africa  South Africa  South Africa  South Africa | EPDS-2 (study round 1) | Antenatal depression | Study1: Pregnant women | Study 1: 18-42 years | 1144 | Xhosa | Study1.1. The EPDS-2: 0.55 | 3 | 42% | 475 | 0.79 | 0.89 | NR | NR | 0.92 (0.90-0.93) |
|  |  |  |  | EPDS-3 (study round 1) |  |  |  |  |  | Study1.2. The EPDS-3: 0.72 | 10 | 42% | 475 | 0.97 | 0.64 | NR | NR | 0.93 (0.92-0.95) |
|  |  |  |  | EPDS-5 (study round 1) |  |  |  |  |  | Study1.3. The EPDS-5: 0.80 | 10 | 42% | 475 | 0.97 | 0.82 | NR | NR | 0.97 (0.97-0.98) |
|  |  |  |  | EPDS-7 (study 1round 1) |  |  |  |  |  | Study1.4. The EPDS-7: 0.83 | 10 | 42% | 475 | 0.99 | 0.85 | NR | NR | 0.98 (0.98-0.99) |
|  |  |  |  | EPDS-2 (study round 2) |  | Study2: Pregnant women | Study 2: 18- 43 years | 361 |  | Study2.1. The EPDS-2: 0.58 | 3 | 46% | 165 | 0.81 | 0.91 | NR | NR | 0.91 (0.88-0.94) |
|  |  |  |  | EPDS-3 (study round 2) |  |  |  |  |  | Study2.2. The EPDS-3: 0.77 | 10 | 46% | 165 | 0.96 | 0.68 | NR | NR | 0.92 (0.89-0.95) |
|  |  |  |  | EPDS-5 (study round 2) |  |  |  |  |  | Study2.3. The EPDS-5: 0.79 | 10 | 46% | 165 | 0.93 | 0.87 | NR | NR | 0.96 (0.94-0.98) |
|  |  |  |  | EPDS-7 (study 1round 2) |  |  |  |  |  | Study2.4. The EPDS-7: 0.83 | 10 | 46% | 165 | 0.97 | 0.76 | NR | NR | 0.98 (0.96-0.99) |
| 56 | Ventevogel, 2014(144) | Validation of the Kirundi versions of brief self-rating scales for common mental disorders among children in Burundi | Burundi | DSRS | 1. The DSRS: Symptoms of depression | Primary school children | 10 - 15 years | 65 | Kirundi | 1. The DSRS: 0.85 | 19 | 18% | 12 | 0.64 | 0.88 | 0.54 | 0.92 | 0.85 (0.73-0.97) |
|  |  |  |  | CPSS | 2. The CPSS: PTSD |  |  |  |  | 2. The CPSS: 0.90 | 26 | 23% | 15 | 0.71 | 0.83 | 0.36 | 0.96 | 0.78 (0.62-0.95) |
|  |  |  |  | SCARED-41 | 3. The SCARED-41: Anxiety disorders |  |  |  |  | 3. The SCARED-41: 0.92 | 44 | 23% | 15 | 0.55 | 0.9 | 0.8 | 0.74 | 0.69 (0.54-0.84) |
| 57 | Abdullahi, 2015(145) | 'A pilot psychometric investigation of the Sexual Addiction Screening Test (SAST) and Women's Sexual Addiction Screening Test (W-SAST) in a Nigerian hospital setting': Erratum | Nigeria | SAST | Sexual addiction | Stable psychiatric inpatients and outpatients  Stable psychiatric inpatients and outpatients (Females only) | 18 years and above | 91  47 | English | 1. SAST: 0.70 | 14 | 100% | 91 | 0.83 | 0.93 | 0.71 | 0.97 | 0.97 |
|  |  |  |  | W-SAST |  |  |  |  |  | 2. W-SAST: 0.77 | 9 | 100% | 47 | 0.8 | 0.83 | 0.36 | 0.97 | 0.9 |
| 58 | Bell, 2015(119)* | Development of a brief screening tool for women's mental health assessment in refugee settings: A psychometric evaluation | Rwanda | SRQ-SIB | Common mental health disorders | Congolese refugee women living in long-term temporary camps in Rwanda. | 15-49 years | 810 | Kinyarwanda | 0.79 | 7 | NR | NR | 0.88 | 0.86 | NR | NR | 0.94 |
| 59 | Bhana, 2015(60) | The validity of the Patient Health Questionnaire for screening depression in chronic care patients in primary health care in South Africa | South Africa | PHQ-9 | Major depressive disorder | Chronic care patients (e.g., HIV, hypertension, diabetes) | 18-88 years | 676 | seTswana or English. | 1.The PHQ-9: 0.76 | 9 | 11.47% | NR | 0.49 | 0.94 | NR | NR | 0.85 (0.82-0.88) |
|  |  |  |  | PHQ-2 |  |  |  |  |  | 2.The PHQ-2: NA | 2 | 11.47% | NR | 0.6 | 0.84 |  |  | 0.76 (0.73-0.79) |
| 60 | Francis, 2015(146) | Validation of the MINI (DSM IV) Tool for the Assessment of Alcohol Dependence among Young People in Northern Tanzania Using the Alcohol Biomarker Phosphatidylethanol (PEth) | Tanzania | MINI | Alcohol use disorder | Alcohol users | 18-24 years | 202 | NR | NR | 4 | 12.4% | 25 | 0.4 | 0.84 | 0.26 | 0.91 | 0.62 (0.52-0.72) |
| 61 | Hanlon, 2015(62) | Validity of brief screening questionnaires to detect depression in primary care in Ethiopia | Ethiopia | PHQ-9 | Major Depressive Disorder (MDD) | Patients attending the PHC facilities | 18 years and above | 306 | Amharic | 1.PHQ-9: 0.84 | 5 | 5.9% | 18 | 0.83 | 0.75 | 0.17 | 0.99 | 0.85 (0.77-0.95) |
|  |  |  |  | PHQ-2 |  |  |  |  |  | 2.PHQ-2: NR | 1 | 5.9% | 18 | 0.83 | 0.61 | 0.12 | 0.98 | 0.78 (0.66-0.90) |
|  |  |  |  | SRQ-20 |  |  |  |  |  | 3.SRQ-20: 0.90 | 8 | 5.9% | 18 | 0.83 | 0.74 | 0.17 | 0.99 | 0.84 (0.76-0.91) |
|  |  |  |  | K10 |  |  |  |  |  | 4.K6: 0.88 | 18 | 5.9% | 18 | 0.78 | 0.77 | 0.13 | 0.98 | 0.83 (0.74-0.92) |
|  |  |  |  | K6 |  |  |  |  |  | 5.K10: 0.85 | 9 | 5.9% | 18 | 0.78 | 0.73 | 0.15 | 0.981 | 0.84 (0.75-0.92) |
| 62 | Khalifa, 2015(146) | Postnatal depression among Sudanese women: prevalence and validation of the Edinburgh Postnatal Depression Scale at 3 months postpartum | Sudan | EPDS | Postnatal depression (PND) | Women at 3 months postpartum | 15-25 years | 238 | Arabic | 0.83 | 12 | 9.2% | NR | 0.89 | 0.82 | 0.33 | 0.99 | 0.89 (0.779-0.999) |
| 63 | Binagwaho, 2016(147) | Validating the Children's Depression Inventory in the context of Rwanda | Rwanda | CDI | Depression | Children living with HIV | 7-14 years | 10 | Kinyarwanda | NR | >5 | 25% | NR | 0.76 (0.57-0.89) | 0.92 (0.84-0.96) | 0.76 | 0.92 | 0.87 (0.77-0.97) |
| 64 | Chibanda, 2016(68) | Validation of screening tools for depression and anxiety disorders in a primary care population with high HIV prevalence in Zimbabwe | Zimbabwe | SSQ-14 (any CMD) | Any common mental disorder (CMD) | Adults attending the clinic | 18 years or older | 264 | Shona | 0.74 | 9 | 37% | 97 | 0.84 (0.78-0.89) | 0.73 (0.63-0.81) | 0.82 | 0.75 | 0.86 (0.81-0.90) |
|  |  |  |  | SSQ-14 (Depression) | Depression |  |  |  |  | NR | 9 | 20% | 52 | 0.86 (0.79-0.91) | 0.7 (0.61-0.79) | 0.79 | 0.7 | NR |
|  |  |  |  | PHQ-9 (Depression) |  |  |  |  |  | 0.86 | 11 | 20% | 52 | 0.85 (0.78-0.90) | 0.69 (0.59-0.77) | 0.78 | 0.77 | 0.84 (0.79-0.88) |
|  |  |  |  | PHQ-2(Depression) |  |  |  |  |  | NR | 2 | 20% | 52 | 0.91 (0.86-0.95) | 0.4 (0.31-0.5) | NR | NR | NR |
|  |  |  |  | GAD-7 (Anxiety) | Anxiety |  |  |  |  | 0.87 | 10 | 3% | 9 | 0.89 (0.81-0.94) | 0.73 (0.65-0.8) | 0.69 | 0.91 | 0.90 (0.87- 0.94) |
| 65 | Gelaye, 2016(76) | Diagnostic validity of the Patient Health Questionnaire-2 (PHQ-2) among Ethiopian adults | Ethiopia | PHQ-2 | Major Depressive Disorder (MDD) | Adult outpatients | 18-69 years | 363 | Amharic | NR | 3 | NR | 46 | 0.74 (0.59-0.86) | 0.60 (0.54-0.65) | 0.21 | 0.94 | 0.72 (0.64-0.79) |
| 66 | Kane, 2016(42)* | Validation of a substance and alcohol use assessment instrument among orphans and vulnerable children in Zambia using Audio Computer Assisted Self-Interviewing (ACASI) | Zambia | ASSIST - TABACCO (Low vs moderate) | Tabacco use | Orphans and Vulnerable Children (OVC) | 13-17 years | 502 | 1. English 2. Bemba 3. Nyanja | 1.ASSIT TABACO: 0.8 | 24 | NR | NR | 0.57 | 0.57 | NR | NR | 0.56 (0.43-0.70) |
|  |  |  |  | ASSIST - TABACCO (Moderate vs high) |  |  |  |  |  |  | 26 | NR | NR | 0.56 | 0.50 |  |  | 0.57 (0.43-0.51) |
|  |  |  |  | ASSIST - ALCOHOL (Low vs moderate) | Alcohol use |  |  |  |  | 2.ASSIST ALCOHOL:0.85 | 11 | NR | NR | 0.69 | 0.69 |  |  | 0.71 (0.61-0.80) |
|  |  |  |  | ASSIST - ALCOHOL (Moderate vs high) |  |  |  |  |  |  | 19 | NR | NR | 0.61 | 0.5 |  |  | 0.67 (0.56-0.77) |
|  |  |  |  | ASSIST- INHALANTS (Low vs moderate) | Inhalants use |  |  |  |  | 3. ASSIST INHAHANTS: 0.84 | 19 | NR | NR | 0.75 | 0.75 |  |  | 0.80 (0.67-0.92) |
|  |  |  |  | ASSIST - INHALANTS (Moderate vs high) |  |  |  |  |  |  | 22 | NR | NR | 0.44 | 0.44 |  |  | 0.40 (0.22-0.58) |
|  |  |  |  | ASSIST - Cannabis (Low vs moderate) | Cannabis use |  |  |  |  | 4. ASSIST Cannabis: 0.89 | 21 | NR | NR | 0.65 | 0.75 |  |  | 0.76 (0.61-0.90) |
|  |  |  |  | ASSIST -Cannabis (Moderate vs high) |  |  |  |  |  |  | 25 | NR | NR | 0.48 | 0.41 |  |  | 0.46 (0.28-0.65) |
|  |  |  |  | ASSIST - COCAINE (Low vs moderate) | Cocaine use |  |  |  |  | 5 ASSIST COCAINE: 0.85 | 18 | NR | NR | 0.75 | 0.72 |  |  | 0.81 (0.69-0.93) |
|  |  |  |  | ASSIST - COCAINE (Moderate vs high) |  |  |  |  |  |  | 21 | NR | NR | 0.43 | 0.44 |  |  | 0.44 (0.25-0.64) |
|  |  |  |  | ASSIST - AMPHETAMINES (Low vs moderate) | Amphetamines |  |  |  |  | 6. ASSIST AMPHETAMINES: 0.86 | 15 | NR | NR | 0.67 | 0.71 |  |  | 0.72 (0.56-0.89) |
|  |  |  |  | ASSIST - AMPHETAMINES (Moderate vs high) |  |  |  |  |  |  | 20 | NR | NR | 0.45 | 0.47 |  |  | 0.49 (0.29-0.69) |
|  |  |  |  | ASSIST - SEDATIVES (Low vs moderate) | Sedatives use |  |  |  |  | 7 ASSIST SEDATIVES: 0.87 | 16 | NR | NR | 0.69 | 0.70 |  |  | 0.68 (0.49-0.86) |
|  |  |  |  | ASSIST - SEDATIVES (Moderate vs high) |  |  |  |  |  |  | 21 | NR | NR | 0.47 | 0.50 |  |  | 0.50 (0.29-0.71) |
|  |  |  |  | ASSIST - HALLUCINOGENS (Low vs moderate) | Hallucinogens |  |  |  |  | 8 ASSIST HALLUCINOGENS: 0.84 | 20 | NR | NR | 0.73 | 0.80 |  |  | 0.77 (0.62-0.92) |
|  |  |  |  | ASSIST - HALLUCINOGENS (Moderate vs high) |  |  |  |  |  |  | 21 | NR | NR | 0.39 | 0.40 |  |  | 0.45 (0.24-0.65) |
|  |  |  |  | ASSIST - OTHER SUBSTANCES (Low vs moderate) | Other substances use |  |  |  |  | 9. ASSIST OTHER SUBSTANCE:0.83 | 15 | NR | NR | 0.65 | 0.72 |  |  | 0.78 (0.65-0.91) |
|  |  |  |  | ASSIST - OTHER SUBSTANCE (Moderate vs high) |  |  |  |  |  |  | 21 | NR | NR | 0.39 | 0.41 |  |  | 0.42 (0.24-0.61) |
|  |  |  |  | ASSIST - TOTAL SUBSTANCE INVOLVEMENT (Low vs moderate) | Total substance involvement |  |  |  |  | 10 ASSIST TOTAL SUBSTANCE INVOLVEMENT : 0.98 | 1 | NR | NR | 0.68 | 0.63 |  |  | 0.69 (0.63-0.75) |
|  |  |  |  | ASSIST- TOTAL SUBSTANCE INVOLVEMENT (Moderate vs high) |  |  |  |  |  |  | 24 | NR | NR | 0.59 | 0.59 |  |  | 0.62 (0.55-0.69) |
| 67 | Nakku, 2016(67) | Validity and diagnostic accuracy of the Luganda version of the 9-item and 2-item Patient Health Questionnaire for detecting major depressive disorder in rural Uganda | Uganda | PHQ-9 | Depression | Primary care and hospital patients | 18-82 years | 1407 | Luganda | 1. PHQ-9=0.68 | 5 | 57% | 84 | 0.67 | 0.78 | 0.523 | NR | 0.74 (0.60-0.89) |
|  |  |  |  | PHQ-2 |  |  |  |  |  | 2. PHQ-2=NR | 1 | 57% | 84 | 0.66 | 0.59 | NR | NR | 0.68 (0.54-0.82) |
| 68 | Unterhitzenberger, 2016(148) | Preliminary evaluation of a prolonged grief questionnaire for adolescents | Rwanda | PGQ | Prolonged Grief | Parentally bereaved adolescents | 14-18 years | 69 | Kinyarwanda | 0.94 | 82.5 | NR | 34 | 0.85 | 0.86 | NR | NR | 0.90(0.83-0.98) |
| 69 | Valjee, 2016(41) | The meaning of developmental trauma: Validation of a brief screen for developmental trauma appraisals | South Africa | DTI-PTSD | Posttraumatic stress disorder (PTSD) | Adolescents | 12-20 years | 475 | NR | 0.91 | 12 | 45% | 216 | 0.68 | 0.67 | 0.67 | NR | NR |
|  |  |  |  | DTI-Complex PTSD | Complex PTSD (DTI-Complex PTSD) |  |  |  |  |  | 12 | 9% | 41 | 0.85 | 0.59 | 0.61 |  |  |
| 70 | vanderWesthuizen, 2016(149) | Validation of the Alcohol, Smoking and Substance Involvement Screening Test in a low- and middle-income country cross-sectional emergency centre study |  | ASSIST-TSI - use /abuse | Total substance use / abuse | Patients visiting emergency centers for injuries related to alcohol or drug use | 18 years or older | 200 | 1. English 2. Afrikaans | Total Substance Involvement Score (TSI): 0.87 | 22 | NR | NR | 0.79 | 0.72 | NR | NR | 0.87 |
|  |  |  |  | ASSIST-TSI - Abuse /dependence | Total substance abuse / dependence |  |  |  |  |  | 42 | NR | NR | 0.64 | 0.61 | NR | NR | 0.67 |
|  |  |  |  | The ASSIST - SSI- Alcohol - use /abuse | Alcohol use disorder use / abuse |  |  |  |  | Alcohol: 0.81 | 6.5 | 10% | NR | 0.85 | 0.89 | NR | NR | 0.94 (0.89-0.99) |
|  |  |  |  | The ASSIST -SSI- Alcohol - abuse /dependance | Alcohol use disorder use / abuse |  |  |  |  |  | 14.5 | 24% | NR | 0.6 | 0.6 | NR | NR | 0.68 (0.55-0.80) |
|  |  |  |  | ASSIST - SSI- Illicit drugs - use / abuse | Substance use disorder (illicit drug use) use / abuse |  |  |  |  | Cannabis: 0.81 Methamphetamine: 0.95 Mandrax: 0.90 | 1 | 7.5% | NR | 0.93 | 0.93 | NR | NR | 0.95 (0.87-1.00) |
|  |  |  |  | ASSIST - Illicit drugs - abuse / dependance | Substance use disorder (illicit drug use) abuse / dependence |  |  |  |  |  | 18 | 10% | NR | 0.96 | 0.87 | NR | NR | 0.96 (0.91-1.00) |
| 71 | vanderWesthuizen, 2016(109)^*^ | Validation of the Self Reporting Questionnaire 20-Item (SRQ-20) for Use in a Low- and Middle-Income Country Emergency Centre Setting | South Africa | SRQ-20 | Non-specific psychological distress, including suicidality | Emergency centre (EC) patients | 18 years or older | 200 | NR | 0.84 | 5/6 | NR | NR | 0.83 | 0.76 | NR | NR | 0.87 (0.82-0.92) |
|  |  |  |  | SRQ-20 (Men) |  |  |  |  |  | 0.84 | 4/5 | NR | NR | 0.74 | 0.75 |  |  | 0.86 (0.78-0.93) |
|  |  |  |  | SRQ-20 (Women) |  |  |  |  |  | 0.84 | 6/7 | NR | NR | 0.74 | 0.77 |  |  | 0.87 (0.79-0.96) |
| 72 | Aloba, 2017(110) | The psychometric characteristics of the 4-item Suicidal Behaviors Questionnaire-Revised (SBQ-R) as a screening tool in a non-clinical sample of Nigerian university students | Nigeria | SBQ-R-4 | Suicide risk | undergraduate students | 18-31 | 536 | NR | 0.8 | 8 | 3.2% | 17 | 0.88 | 0.88 | 0.88 | 0.88 | 0.93(0.89-0.97) |
| 73 | Baron 2017 (150) | Validation of the 10-item Centre for Epidemiological Studies Depression Scale (CES-D-10) in Zulu, Xhosa and Afrikaans populations in South Africa | South Africa | CES-D-10 - Zulu | Major depressive disorders | Community dwellers or members | 1.Zulu: 15-86 | 306 | 1. Zulu | 0.69-0.89 | 12 | 6.9% | 21 | 0.71 | 0.73 | 0.16 | 0.97 | 0.81 (0.71-0.90) |
|  |  |  |  | CES-D-10 - Xhosa |  |  | 2.Xhosa: 15-77 | 289 | 2. Xhosa |  | 13 | 6.9% | 21 | 0.81 | 0.95 | 0.55 | 0.99 | 0.94 (0.89-0.99) |
|  |  |  |  | CES-D-10 - Afrikaans |  |  | 3.Afrikaans: 15-84 | 303 | 3. Afrikaans |  | 11 | 18% | 52 | 0.85 | 0.84 | 0.54 | 0.96 | 0.93 (0.90-0.96) |
| 74 | Marsay, 2017(151) | Validation of the Whooley questions for antenatal depression and anxiety among low-income women in urban South Africa | South Africa | Whooley – no help Q-Overall | 1.Whooley-Without-Help Question -Overall: Depression, trauma / anxiety | 22 and 28 weeks pregnant women | 18-42 years | 145 | English | NR | 2 | 38.6% | 56 | 0.64(0.50-0.77) | 0.80 (0.70-0.89) | NR | NR | 0.756 (0.679-0.832) |
|  |  |  |  | Whooley – no help Q - Depression | 1.1. Whooley-Without-Help Question -Depression: Depression |  |  |  |  |  | 2 | 19.3% | 28 | 0.79 (0.59-0.92) | 0.73(0.64-0.81) |  |  | 0.79(0.72-0.86) |
|  |  |  |  | Whooley – no help Q- Trauma /Anxiety | 1.2. Whooley-Without-Help Question -Trauma /Anxiety: Trauma / anxiety |  |  |  |  |  | 2 | 22.1% | 32 | 0.81(0.63-0.93) | 0.42 (0.33-0.52) |  |  | 0.64 (0.54-0.74) |
|  |  |  |  | Whooley – with help Q - Overall | 2. Whooley-With-Help Question -Overall: Depression, trauma / anxiety |  |  |  |  |  | 2 | 38.6% | 56 | 0.73 (0.60-0.84) | 0.76 (0.66-0.85) |  |  | 0.80 (0.72-0.87) |
|  |  |  |  | Whooley – with help Q - Overall - Depression | 2.1. Whooley-With-Help Question -Depression: Depression |  |  |  |  |  | 3 | 19.3% | 28 | 0.89(0.72-0.98) | 0.68 (0.59-0.77) |  |  | 0.83(0.76-0.91) |
|  |  |  |  | Whooley – with help Q - Trauma /Anxiety | 2.2. Whooley-With-Help Question -Trauma /Anxiety:Trauma /Anxiety |  |  |  |  |  | 4 | 22.1% | 32 | 0.65 (0.45-0.81) | 0.63 (0.54-0.72) |  |  | 0.66(0.56-0.77) |
|  |  |  |  | EPDS - Overall | 3.EPDS - Overall: depression, Trauma / Anxiety: |  |  |  |  |  | 12 | 38.6% | 56 | 0.79 (0.66-0.88) | 0.84 (0.75-0.91) |  |  | NR |
|  |  |  |  | EPDS - Depression | 3.1. EPDS - Depression: Depression |  |  |  |  |  | 14 | 19.3% | 28 | 0.89(0.72-0.98) | 0.88 (0.81-0.93) |  |  |  |
|  |  |  |  | EPDS - Trauma / Anxiety | 3.2.EPDS - Trauma / Anxiety: Trauma / Anxiety |  |  |  |  |  | 11 | 22.1% | 32 | 0.81(0.63-0.93) | 0.61(0.51-0.70) |  |  |  |
|  |  |  |  | EPDS - Anxiety subscale Trauma / Anxiety | 4.EPDS -Anxiety sub-scale for Trauma / Anxiety: Trauma / Anxiety |  |  |  |  |  | 7 | 22.1% | 32 | 0.55(0.36-0.73) | 0.82 (0.73-0.88) |  |  |  |
|  |  |  |  | EPDS -Anxiety sub-scale for Trauma | 4.1. EPDS - Anxiety sub-scale for Trauma: Trauma |  |  |  |  |  | 7 | 7.6% | 11 | 0.64 (0.31-0.89) | 0.77(0.69-0.84) |  |  |  |
|  |  |  |  | EPDS - Anxiety sub-scale for Anxiety | 4.2. EPDS - Anxiety sub-scale for Anxiety: Anxiety |  |  |  |  |  | 7 | 14.5% | 21 | 0.71(0.48-0.89) | 0.62 (0.53-0.72) |  |  |  |
| 75 | Mellins, 2017(152) | Validation of the Client Diagnostic Questionnaire to Assess Mental Health in South African Caregivers of Children | South Africa | CDQ - Substance use | 1. Substance use | Female caregivers | 18-74 years | 322 | isiZulu | NR | NR | 19% | NR | 0.73 | 0.81 | 0.47 | 0.93 | NR |
|  |  |  |  | CDQ - Psychiatric disorders | 2. Psychiatric disorders |  |  |  |  |  |  | 15% | NR | 0.74 | 0.79 | 0.39 | 0.95 |  |
| 76 | Morojele, 2017(111) | Utility of brief versions of the Alcohol Use Disorders Identification Test (AUDIT) to identify excessive drinking among patients in HIV care in South Africa | South Africa | AUDIT-C (Men) | Excessive alcohol use | Patients receiving antiretroviral therapy (ART) in HIV clinics | 18 years and above | 188 | NR | 1. AUDIT Overall: 0.82 2.AUDIT men: 0.76 3.AUDIT women: 0.85 | 2 | 68.8% | NR | 0.98 | 0.14 | 0.72 | 0.8 | 0.85 (0.77-0.92) |
|  |  |  |  | AUDIT-C (Women) |  |  |  |  |  |  |  | 50.05% | RN | 0.98 | 0.23 | 0.57 | 0.92 | 0.92 (0.86-0.98) |
|  |  |  |  | AUDIT-3 (Men) |  |  |  |  |  |  | 1 | 68.8% | 64 | 0.91 | 0.31 | 0.74 | 0.6 | 0.80 (0.72-0.89) |
|  |  |  |  | AUDIT-3 (Women) |  |  |  |  |  |  |  | 50.05% | 48 | 0.81 | 0.66 | 0.71 | 0.78 | 0.85 (0.77-0.93) |
|  |  |  |  | AUDIT-QF (Men) |  |  |  |  |  |  | 2 | 68.8% | 64 | 0.97 | 0.17 | 0.72 | 0.71 | 0.82 (0.74-0.90) |
|  |  |  |  | AUDIT-QF (Women) |  |  |  |  |  |  |  | 50.05% | 48 | 0.98 | 0.30 | 0.59 | 0.933 | 0.92 (0.87-0.98) |
|  |  |  |  | AUDIT-PC-5 (Men) |  |  |  |  |  |  | 4 | 68.8% | 64 | 0.95 | 0.83 | 0.92 | 0.89 | 0.96 (0.91-1.00) |
|  |  |  |  | AUDIT-PC-5 (Women) |  |  |  |  |  |  | 3 | 50.05% | 48 | 0.98 | 0.77 | 0.81 | 0.97 | 0.97 (0.93-1.00) |
|  |  |  |  | AUDIT-4 (Men) |  |  |  |  |  |  | 4 | 68.8% | 64 | 0.95 | 0.55 | 0.82 | 0.84 | 0.92 (0.87-0.98) |
|  |  |  |  | AUDIT-4 (Women) |  |  |  |  |  |  |  | 50.05% | 48 | 0.92 | 0.85 | 0.86 | 0.91 | 0.95 (0.90-1.00) |
|  |  |  |  | m-FAST- AUDIT-4 (Men) |  |  |  |  |  |  | 2 | 68.8% | 64 | 0.95 | 0.59 | 0.84 | 0.85 | 0.93 (0.88-0.98) |
|  |  |  |  | m-FAST- AUDIT-4 (Women) |  |  |  |  |  |  | 1 | 50.05% | 48 | 0.96 | 0.55 | 0.69 | 0.93 | 0.94 (0.89-0.99) |
| 77 | Ola, 2017(43)* | Validation of CRAFFT for Use in Youth Correctional Institutions in Lagos, Nigeria | Nigeria | CRAFFT | Substance-related disorders (SRD) | Adolescents in correctional centers | 13-17 years | 178 | NR | 0.85 | > 1 | NR | NR | 0.91 | 0.81 | 0.88 | 0.87 | 0.89(0.77-1.00) |
| 78 | Akena, 2018(153) | Sensitivity and specificity of the Akena Visual Depression Inventory (AViDI-18) in Kampala (Uganda) and Cape Town (South Africa) | 1. Uganda 2. South Africa | AVIDI-18 | Major Depression Disorder (MDD) | Patients with low literacy | 18 years and above | 343 | NA as it is a visual tool | 0.87 | 16 | 22.7% | 78 | 0.87 | 0.85 | 1 | 0.78 | 0.92 (0.88-0.95) |
| 79 | Aloba, 2018(154) | Positive and Negative Suicide Ideation (PANSI) Inventory: Initial Psychometric Properties as a Suicide Risk Screening Tool Among Nigerian University Students | Nigeria | PANSI-NSI | Suicide Ideation | Under-graduate university students | 16-35 years | 514 | English | 1. Factor 1 (PANSI-NSI): 0.76 | 17 | 2.9% | 15 | 0.80 | 0.93 | 0. 91 | 0.82 | 0.82 ( 0.58-1.00) |
| 80 | Aloba, 2018(155) | Psychometric Adaptation of the Beck Hopelessness Scale as a Self-Rated Suicide Risk Screening Instrument Among Nigerian University Students | Nigeria | BHS | High suicide risk | Undergraduate students | 18 to 29 years | 554 | NR | 0.87 | 7 | 3.6% | 20 | 0.7 | 0.91 | 0.88 | 0.75 | 0.90 (0.84-0.95) |
| 81 | Chorwe-Sungani, 2018(50) | Validity and utility of instruments for screening of depression in women attending antenatal clinics in Blantyre district in Malawi | Malawi | EPDS | Depression | Pregnant women | 18 years and above | 97 | Chichewa | 1. EPDS: 0.80 | 10 | 25.8% | 25 | 0.68 (0.47–0.85) | 0.88 (0.78-0.94) | 0.65 | 0.89 | 0.85 (0.763-0.915) |
|  |  |  |  | HSCL-15 |  |  |  |  |  | 2. HSCL-15: 0.85 | 1.75 | 25.8% | 25 | 0.72 (0.51-0.88) | 0.93 (0.85-0.98) | 0.78 | 0.91 | 0.91 (0.835-0.959) |
|  |  |  |  | SRQ |  |  |  |  |  | 3.SRQ: 0.86 | 10 | 25.8% | 25 | 0.60 (0.39-0.88) | 0.97 (0.90-0.99) | 0.88 | 0.88 | 0.91 (0.84-0.96) |
|  |  |  |  | 3-item screener |  |  |  |  |  | 4. 3-item screener: 0.70 | 1 | 25.8% | 25 | 0.80 (0.59-0.79) | 0.81 (0.70-0.89) | 0.59 | 0.92 | 0.85 (0.77-0.92) |
| 82 | Green, 2018(52) | Developing and validating a perinatal depression screening tool in Kenya blending Western criteria with local idioms: A mixed methods study | Kenya | EPDS - Orginal (two weeks) | Major Depressive Episode (MDE) | Pregnant women and new mothers | 18 years old and above | 193 | Kiswahili | 1. EPDS (Original-Two weeks): 0.78 | 16 | 5.2% | NR | 0.7 | 0.72 | NR | NR | 0.8 |
|  |  |  |  | EPDS: Revised (one week) |  |  |  |  |  | 2.EPDS (Revised-One weeks):0.83 | 13 | 5.2% | NR | 0.6 | 0.73 |  |  | 0.8 |
|  |  |  |  | PHQ-9 |  |  |  |  |  | 3.PHQ-9: 0.81 | 15 | 5.2% | NR | 0.7 | 0.74 |  |  | O.79 |
|  |  |  |  | PDEPS |  |  |  |  |  | 4.PDEPS: 0.77 | 13 | 5.2% | NR | 0.9 | 0.9 |  |  | 0.89 |
| 83 | Manzar, 2018(156) | Validation of the adapted Leeds sleep evaluation questionnaire in Ethiopian university students | Ethiopia | The Adapted Leeds Sleep Evaluation Questionnaire Mizan (LSEQ-M) | Insomnia | University students | 21.87 ±4.13 years | 424 | English | 0.84 | 52.6 | 31.4% | 133 | 0.94 | 0.8 | NR | NR | 0.95 |
| 84 | Netsereab, 2018(157) | Validation of the WHO self-reporting questionnaire-20 (SRQ-20) item in primary health care settings in Eritrea | Eritrea | SRQ-20 (Overall) | Common mental disorder (CMD) | Outpatients at health facilities | 18-65 years | 266 | Tigrigna | 0.784 | 5/6 | 23% | 60 | 0.88 | 0.82 | 0.55 | 0.93 | 0.88(0.83-0.93) |
|  |  |  |  | SRQ-20 (Men) |  |  |  |  |  | NR | 4/5 | 17% | NR | 0.86 | 0.74 | NR | NR | 0.88(0.79-0.96) |
|  |  |  |  | SRQ-20(Women) |  |  |  |  |  |  | 5/6 | 27% | NR | 0.82 | 0.74 |  |  | 0.87(0.81-0.92) |
| 85 | Olagundoye, 2018(121) | Adaptation and validation of the disruptive behaviour disorders teacher rating scale as a screening tool for early detection of disruptive behaviour disorders in schools in a lower-middle income setting | Nigeria | DBD-ADHD | 1. Attention-deficit/hyperactivity disorder (ADHD) | Children/Adolescents | 4 - 16 years | 1508 | Yorubas | 0.965 | 2.14 | 43.3% | NR | 0.66 | 0.94 | 0.39 | NR | NR |
|  |  |  |  | DBD-CD | 2. Conduct disorder (CD) |  |  |  |  | NR | 1.26 | 28.9% | NR | 0.12 | 1.00 | 0.95 |  |  |
|  |  |  |  | DBD-ODD | 3. Oppositional defiant disorder (ODD) |  |  |  |  |  | 1.83 | 27.9% | NR | 0.50 | 0.97 | 0.75 |  |  |
| 86 | Saal, 2018(158) | Utility of the Beck Depression Inventory in measuring major depression among individuals seeking HIV testing in the Western Cape, South Africa | South Africa | BDI-I | Major depressive disorder (MDD) | Individuals seeking HIV testing | 18 - 71 years | 500 | English | 0.92 | 19.52 | 14.4% | NR | 0.67 | 0.67 | 0.25 | 0.92 | 0.77 (0.71-0.82) |
| 87 | Seun-Fadipe, 2018(159) | Sleep Hygiene Index: Psychometric Characteristics and Usefulness as a Screening Tool in a Sample of Nigerian Undergraduate Students | Nigeria | SHI | Poor sleep quality | Undergraduate Students | 21.60 (2.87) | 348 | English | 0.64 | 16 | 42.5% | 148 | 0.77 | 0.48 | 0.60 | 0.67 | 0.65 (0.59-0.71) |
| 88 | vanHeyningen, 2018(112)* | Comparison of mental health screening tools for detecting antenatal depression and anxiety disorders in South African women | South Africa | EPDS (MDE) | Major Depressive Episode (MDE) | Women | 18-48 years | 1. English (n = 312)  2. isiXhosa (n = 37)  3.Afrikaans(n= 27) | 1.English 2. isiXhosa  3.Afrikaans | 1. The EPDS: <0.8 and >0.7 2.The PHQ-9: >0.8  3.The K10: >0.8 4. The K6: >0.8 5. The Whooley questions: >0.8 6. The GAD-2: <0.8 and >0.7 7. The Whooly without help questions: <0.8 >0.7 8. EPDS - 3: <0.6 and >0.5 9. PHQ-2: <0.6 and >0.5 10. EPDS-Anxiety sub-scale: <0.6 and >0.5 | 14 | NR | NR | 0.86 | 0.81 | 0.56 | 0.96 | 0.91 |
|  |  |  |  | 3-item EPDS (MDE) | Major Depressive Episode (MDE) |  |  |  |  |  | 3 | NR | NR | 0.83 | 0.74 | 0.46 | 0.94 | 0.85 |
|  |  |  |  | K10 (MDE) | Major Depressive Episode (MDE) |  |  |  |  |  | 15 | NR | NR | 0.81 | 0.85 | 0.6 | 0.94 | 0.89 |
|  |  |  |  | K6 (MDE) | Major Depressive Episode (MDE) |  |  |  |  |  | 10 | NR | NR | 0.78 | 0.87 | 0.62 | 0.93 | 0.88 |
|  |  |  |  | PHQ9 (MDE) | Major Depressive Episode (MDE) |  |  |  |  |  | 10 | NR | NR | 0.79 | 0.82 | 0.55 | 0.93 | 0.89 |
|  |  |  |  | PHQ2 (MDE) | Major Depressive Episode (MDE) |  |  |  |  |  | 3 | NR | NR | 0.65 | 0.84 | 0.51 | 0.9 | 0.84 |
|  |  |  |  | Whooley (MDE) | Major Depressive Episode (MDE) |  |  |  |  |  | 2 | NR | NR | 0.83 | 0.85 | 0.6 | 0.95 | 0.86 |
|  |  |  |  | Whooley + help (MDE) | Major Depressive Episode (MDE) |  |  |  |  |  | 2 | NR | NR | 0.89 | 0.79 | 0.53 | 0.96 | 0.88 |
|  |  |  |  | EPDS - 3A (Anxiety) | Anxiety disorders |  |  |  |  |  | 5 | NR | NR | 0.67 | 0.59 | 0.33 | 0.86 | 0.69 |
|  |  |  |  | K10 (Anxiety) | Anxiety disorders |  |  |  |  |  | 11 | NR | NR | 0.76 | 0.7 | 0.43 | 0.91 | 0.77 |
|  |  |  |  | K6 (Anxiety) | Anxiety disorders |  |  |  |  |  | 8 | NR | NR | 0.69 | 0.76 | 0.46 | 0.89 | 0.77 |
|  |  |  |  | GAD-2 (Anxiety) | Anxiety disorders |  |  |  |  |  | 2 | 32% | NR | 0.64 | 0.74 | 0.43 | 0.87 | 0.73 |
|  |  |  |  | EPDS (MDE & / Anxiety) | Major Depressive Episode (MDE) and/or anxiety disorders |  |  |  |  |  | 13 | 32% | NR | 0.75 | 0.78 | 0.62 | 0.87 | 0.83 |
|  |  |  |  | 3-item EPDS (MDE & / Anxiety) | Major Depressive Episode (MDE) and/or anxiety disorders |  |  |  |  |  | 11 | 32% | NR | 0.7 | 0.77 | 0.59 | 0.84 | 0.79 |
|  |  |  |  | K10 (MDE & / Anxiety) | Major Depressive Episode (MDE) and/or anxiety disorders |  |  |  |  |  | 8 | 32% | NR | 0.8 | 0.79 | 0.65 | 0.89 | 0.85 |
|  |  |  |  | K6 (MDE & / Anxiety) | Major Depressive Episode (MDE) and/or anxiety disorders |  |  |  |  |  | 10 | 32% | NR | 0.74 | 0.85 | 0.7 | 0.87 | 0.85 |
|  |  |  |  | PHQ9 (MDE & / Anxiety) | Major Depressive Episode (MDE) and/or anxiety disorders |  |  |  |  |  | 2 | 32% | NR | 0.66 | 0.76 | 0.7 | 0.84 | 0.84 |
|  |  |  |  | PHQ2 (MDE & / Anxiety) | Major Depressive Episode (MDE) and/or anxiety disorders |  |  |  |  |  | 2 | 32% | NR | 0.75 | 0.69 | 0.54 | 0.85 | 0.78 |
|  |  |  |  | Whooley (MDE & / Anxiety) | Major Depressive Episode (MDE) and/or anxiety disorders |  |  |  |  |  | 2 | 32% | NR | 0.66 | 0.87 | 0.71 | 0.84 | 0.79 |
|  |  |  |  | Whooley + help (MDE & / Anxiety) | Major Depressive Episode (MDE) and/or anxiety disorders |  |  |  |  |  | 2 | 32% | NR | 0.73 | 0.82 | 0.66 | 0.86 | 0.81 |
| 89 | Verhey, 2018(160) | Validation of the posttraumatic stress disorder checklist - 5 (PCL-5) in a primary care population with high HIV prevalence in Zimbabwe | Zimbabwe | PCL-5 - General population | Posttraumatic Stress Disorder (PTSD) - general population | Clinic attendants | 18 years and above | 204 | Shona | 1. Whole sample:0.92 | 33 | 19.6% | 40 | 0.75 (0.60-0.86) | 0.71 (0.63-0.78) | 0.46 | 0.89 | 0.78 (0.72-0.83) |
|  |  |  |  | PCL-5 - PLWHIV | Posttraumatic Stress Disorder (PTSD) - PLWHIV |  |  | 91 |  | 2. PLWH only: NR |  | NR | NR | 0.79 (0.59-0.92) | 0.68 (0.55-0.79) | 0.52 | 0.88 | NR |
| 90 | Woldetensay, 2018(63) | Validation of the Patient Health Questionnaire (PHQ-9) as a screening tool for depression in pregnant women: Afaan Oromo version | Ethiopia | PHQ-9 | Depression | Pregnant women | 18 to 40 years | 216 | Afaan Oromo | 0.84 | 8 | 12.6% | 28 | 0.81 | 0.79 | NR | NR | 0.88 (0.81-0.95) |
| 91 | Abrahams, 2019(113) | Validation of a brief mental health screening tool for pregnant women in a low socio-economic setting | South Africa | The 3-item screening tool (based on a 4-week recall) | CMD (Common mental disorders) | Pregnant women | 15-38 years | 66: 1) 30 for English, 2) 19 for Afrikaans and 3) 17 for ixiXhosa | 1.English, 2.Afrikaans and 3. isiXhosa | NR | 2 | 24.3% | 16 | 0.78 | 0.90 | NR | NR | 0.87 |
|  |  |  |  | The 3-item screening tool (based on a 2-week recall) |  |  |  |  |  |  | 2 | 24.3% | 16 | 0.86 | 0.93 |  |  | 0.93 |
| 92 | Ashaba, 2019(44) | Development and validation of a 20-item screening scale to detect major depressive disorder among adolescents with HIV in rural Uganda: A mixed-methods study | Uganda | 20-item depression scale | Major depressive disorder | Adolescents living with HIV (ALWH) | 13-17 years | 224 | Runyankore | 0.91 | 10 | 17% | 37 | 0.78 (0.65-0.92) | 0.81 (0.76-0.87) | 0.45 | 0.94 | 0.84 (0.77-0.90) |
| 93 | Bhana, 2019(75) | Validation of a brief mental health screening tool for common mental disorders in primary healthcare | South Africa | AUD-C (Alcohol abuse) | 1. Alcohol use disorder | Primary care patients | 18 years and above | 1214 | isiZulu or English | 0.87 | 4 | 2% | 17 | 0.82 | 0.83 | 0.42 | 0.97 | 0.91 (0.88-0.95) |
|  |  |  |  | PHQ-2 | 2. Depression |  |  |  |  | 0.71 | 3 | 6% | 57 | 0.58 | 0.77 | 0.74 | 0.93 | 0.72 (0.65-0.78) |
|  |  |  |  | GAD-2 | 3. Anxiety |  |  |  |  | 0.62 | 3 | 2% | 17 | 0.59 | 0.76 | 0.90 | 0.94 | 0.69 (0.58-0.80) |
| 94 | Ndetei, 2019(161) | The psychometric properties of the Washington Early Recognition Center Affectivity and Psychosis (WERCAP) screen in adults in the Kenyan context: Towards combined large scale community screening for affectivity and psychosis | Kenya | aWERCAP Screen | Affectivity | Kenyan adults with positive screens on the WHO mental health treatment GAP- Intervention Guidelines (mhGAP-IG) | 18-90 years | 674 | Kamba | 1. aWERCAP: 0.89 | 22 | 68.8% | 464 | 0.59 | 0.64 | 0.79 | 0.42 | 0.65( 0.61-0.68) |
|  |  |  |  | pWERCAP Screen | 2. Psychosis |  |  |  |  | 2. pWERCAP: 0.89 | 20 | 68.8% | 464 | 0.82 | 0.43 | 0.76 | 0.53 | 0.66(0.62-0.70) |
| 95 | Phillips, 2019(114) | Screening for HIV-associated neurocognitive disorders in perinatally infected adolescents: youth-International HIV Dementia Scale validation | South Africa | y-IHDS - major ND | HIV-associated neurocognitive disorders | Perinatally HIV-infected adolescents and HIV-negative adolescents | 9-12 years | 247: 203 (Perinatally HIV-infected children and adolescents) and 44 (HIV-negative children and adolescents) | English and isiXhosa | NR | ≤ 10 | 3% | 8 | 1 | 0.24 | 0.60 | 1 | 0.86(0.73-0.99) |
|  |  |  |  | y-IHDS - minor ND |  |  |  |  |  |  | ≤ 11 | 39% | 96 | 0.94 | 0.24 | 0.45 | 0.85 | 0.68 (0.62-0.75) |
|  |  |  |  | y-IHDS - CI |  |  |  |  |  |  | ≤ 12 | NR | NR | 0.94 | 0.24 | 0.47 | 0.85 | 0.70(0.63-0.76) |
| 96 | Sangare, 2019(162) | Validation of two parent-reported autism spectrum disorders screening tools M-CHAT-R and SCQ in Bamako, Mali | Mali | SCQ | Autism spectrum disorders | Patients diagnosed with ASD | 4-20 years | 120 | French | NR | 15 | 50% | 60 | 0.71 (0.61-0.82) | 0.72 (0.57-0.80) | 0.7 | 0.72 | 0.7 |
| 97 | SmithFawzi, 2019(69) | Validating the Patient Health Questionnaire-9 (PHQ-9) for screening of depression in Tanzania | Tanzania | PHQ-9 | Current major depressive episode (MDE) | Public clinics patients | 18-63 years | 174 | Swahili | 0.83 | 9 | 10.3% | 18 | 0.78 (0.52-0.94) | 0.87 (0.80-0.92) | 0.42 | 0.97 | 0.87 (0.77-0.96) |
| 98 | vanHeyningen, 2019(54) | The development of an ultra-short, maternal mental health screening tool in South Africa | South Africa | Whooley01-GAD02(binary) | 1. Antenatal depression 2. Anxiety disorders  3. Maternal suicidal ideation | Pregnant women | 18 years or older | 376 | 1. Afrikaans 2. isiXhosa | NR | 1 | 55% | 167 | 0.8 | 0.74 | NR | NR | 0.81 |
|  |  |  |  | Whooley01-Whooley02 - GAD02 (binary) |  |  |  |  |  |  | 2 | 55% | 167 | 0.74 | 0.85 |  |  | 0.81 |
|  |  |  |  | Whooley01-Whooley02- EPDS10 (binary) |  |  |  |  |  |  | 2 | 40% | 150 | 0.72 | 0.83 |  |  | 0.82 |
|  |  |  |  | Whooley01- GAD02 (binary) -EPDS10 (binary) |  |  |  |  |  |  | 2 | 73% | 236 | 0.57 | 0.91 |  |  | 0.82 |
|  |  |  |  | Whooley01 - Whooley02 - GAD02 (binary) - EPDS10 (binary) |  |  |  |  |  |  | 2 | 73% | 236 | 0.78 | 0.82 |  |  | 0.83 |
|  |  |  |  | 6. EPDS items 1-10 |  |  |  |  |  |  | 13 | 40% | 150 | 0.75 | 0.78 |  |  | 0.83 (0.78-0.88) |
| 99 | Bantjes, 2020(115) | The Utility of the Hopkins Symptom Checklist as a Trans-Diagnostic Screening Instrument for Common Mental Disorders Among Persons Seeking HIV Testing | South Africa | HSCL-25 | Common mental disorders (CMD) | Individuals seeking HIV testing | 18-71 years | 500 | 1. English  2. Afrikaans. | NR | 43 | 37% | NR | 0.69 (0.62-0.76) | 0.70(0.65-0.75) | 0.57 | 0.8 | 0.80 (0.75-0.83) |
| 100 | Cohen, 2020(163)* | Development and validation of the body shape scale (BOSHAS) for assessing body shape perception in African populations | 1. Cameroon 2. Senegal | BOSHAS - Body weight Self-satisfaction | Body image disorders | NR | 18-75 years | 161 | French | NR | NR | NR | NR | 0.70 (0.52-0.72) | 0.71 (0.52-0.72) | NR | NR | NR |
|  |  |  |  | BOSHAS - Normal body- CBS |  |  |  |  |  |  |  | NR | NR | 0.70 | 0.52 |  |  |  |
|  |  |  |  | BOSHAS - Normal body- self-satisfaction |  |  |  |  |  |  |  | NR | NR | 0.72 | 0.53 |  |  |  |
|  |  |  |  | BOSHAS - Importance of appearance-Modern DBS |  |  |  |  |  |  |  | NR | NR | 0.59 | 0.55 |  |  |  |
|  |  |  |  | BOSHAS - Importance of appear-Modern IBS |  |  |  |  |  |  |  | NR | NR | 0.54 | 0.54 |  |  |  |
|  |  |  |  | BOSHAS - Importance of appearance-Modern Self-self-satisfaction |  |  |  |  |  |  |  | NR | NR | 0.52 | 0.54 |  |  |  |
|  |  |  |  | BOSHAS - Modern Aesthetic criteria-Modern DBS |  |  |  |  |  |  |  | NR | NR | 0.63 | 0.53 |  |  |  |
|  |  |  |  | BOSHAS - Modern Aesthetic criteria-Modern IBS |  |  |  |  |  |  |  | NR | NR | 0.63 | 0.54 |  |  |  |
|  |  |  |  | BOSHAS - Modern Aesthetic criteria-Modern self-satisfaction |  |  |  |  |  |  |  | NR | NR | 0.58 | 0.54 |  |  |  |
| 101 | Cumbe, 2020(70) | Validity and item response theory properties of the Patient Health Questionnaire-9 for primary care depression screening in Mozambique (PHQ-9-MZ) | Mozambique | PHQ-9-MZ | Major depressive disorder | Primary care patients | 18 years old or over | 502 | Mozambican Portuguese | 1.PHQ-9-MZ: 0.84 | 9 | 9% | 43 | 0.47 | 0.94 | 0.40 | 0.95 | 0.81 (0.73-0.89) |
|  |  |  | Mozambique | PHQ-2-MZ |  |  |  |  |  | 2.PHQ-2-MZ: 0.61 | 2 | 9% | 43 | 0.74 | 0.72 | 0.20 | 0.97 | 0.78 (0.70-0.85) |
| 102 | Dedeken, 2020(164) | Validity, Reliability, and Diagnostic Cut-off of the Kinyarwandan Version of the Hamilton Depression Rating Scale in Rwanda | Rwanda | HDRS | Depression | Patients with depression and healthy volunteers | 18 years and above | 210 | Kinyarwanda | 0.92 | 17 | 50% | 105 | 0.95 | 0.94 | 0.97 | 0.93 | 0.98 |
| 103 | Degefa, 2020(64) | Validation of the PHQ-9 depression scale in Ethiopian cancer patients attending the oncology clinic at Tikur Anbessa specialized hospital | Ethiopia | PHQ9 | Major Depressive Episode (MDE) | Cancer outpatients | 18 years and above | 163 | Amharic | 0.78 | 4 | 15.3% | 25 | 0.88 | 0.78 | NR | NR | 0.93 (0.88-0.97) |
| 104 | Molebatsi, 202(71) | The validity and reliability of the Patient Health Questionnaire-9 for screening depression in primary health care patients in Botswana | Botswana | PHQ-9 | Depression | Adult primary care attendants | 18 - 79 years | 257 | Setswana or English | 0.80 | 9 | 40.9% | 105 | 0.72(0.63-0.81) | 0.76(0.69–0.83) | 0.68 | 0.80 | 0.81 (0.76-0.85) |
| 105 | Saal, 2020(165)^*^ | Gender Differences in the Utility of the Alcohol Use Disorder Identification Test in Screening for Alcohol Use Disorder Among HIV Test Seekers in South Africa | South Africa | AUDIT (Men) | Alcohol use disorder (AUD) | HIV test-seekers | 18 - 71 years | 242  258 | English | 0.89 | 10 | NR | NR | 0.81 (0.68-0.91) | 0.77 (0.70-0.83) | 0.49 | 0.94 | 0.88 (0.83- 0.92) |
|  |  |  |  | AUDIT (Women) |  |  |  |  |  |  | 7 | NR | NR | 0.82(0.68-0.92) | 0.82 (0.76-0.87) | 0.49 | 0.96 | 0.93 (0.90-0.96) |
| 106 | Sebera, 2020(72) | Validity, reliability and cut-offs of the Patient Health Questionnaire-9 as a screening tool for depression among patients living with epilepsy in Rwanda | Rwanda | PHQ-9 - Mild depression | Mild depression | Patients with epilepsy (PwE) | 15 years and older | 424 | Kinyarwanda | 0.87 | 5 | 37.9% | 123 | 0.72 | 0.70 | 0.79 | 0.61 | 0.80 (0.74- 0.86) |
|  |  |  |  | PHQ-9 - Moderate depression | Moderate depression |  |  |  |  |  | 5 | 13.4% | 71 | 0.89 | 0.59 | 0.51 | 0.92 | 0.84 (0.80-0.90) |
|  |  |  |  | PHQ-9 - Severe depression | Severe depression |  |  |  |  |  | 7 | 15.1% | 63 | 0.94 | 0.65 | 0.32 | 0.98 | 0.87 (0.83-0.93) |
| 107 | Andersen, 2021(166) | Improving Detection of Depression in People Living with HIV: Psychometric Properties of the South African Depression Scale (SADS) | South Africa | SADS | Major Depressive Disorder (MDD) | HIV patients | 18 to 74 years | 236 | 1. English 2. isiXhosa | 0.96 | 27 | 23% | NR | 0.78 | 0.80 | 0.54 | 0.92 | 0.85 |
| 108 | Atkins, 2021(167) | Validity and item response theory properties of the Alcohol Use Disorders Identification Test for primary care alcohol use screening in Mozambique (AUDIT-MZ) | Mozambique | AUDIT-10-MZ | 1. Alcohol use 2. Alcohol dependence 3. Drug dependence 4. drug use | Antenatal, postpartum and outpatient clinics. | 18- 29 years | 502 | Mozambican Portuguese | 1. For the AUDIT-10-MZ: 0.74 | 6 | 3.2% | 16 | 0.69 | 0.92 | NR | NR | 0.94 (0.91-0.96) |
|  |  |  |  | AUDIT-C-MZ |  |  |  |  |  | 2. For the shorter AUDIT-C-MZ: 0.79 | 3 | 3.2% | 16 | 0.56 | 0.91 |  |  | 0.88 (0.80-0.96) |
| 109 | Binagwaho, 2021(168) | Addressing the mental health needs of children affected by HIV in Rwanda: validation of a rapid depression screening tool for children 7-14 years old | Rwanda | CDST | Major Depressive Disorders | Children living with HIV | 7-14 years | 296 | Kinyarwanda | NR | 6 | 14.2% | NR | 0.88 | 0.97 | 0.80 | 0.98 | 0.92 |
| 110 | Blanchard, 2021(117)* | Validating an Instrument for Direct Patient Reporting of Distress and Chemotherapy-Related Toxicity among South African Cancer Patients | South Africa | PRS-SA Instrument | Distress | Patients with solid tumors | 18 years and above | 196 | English or isiZulu | NR | 4 | NR | NR | 0.82 | 0.55 | NR | NR | 0.76 |
| 111 | Lovero, 2021(169) | Brief Screening Tool for Stepped-Care Management of Mental and Substance Use Disorders | Mozambique | mwTool - Any disorder | 1) Any disorder 2) Severe mental disorder for diagnoses of mania, psychosis, or the presence of psychotic symptoms associated with another disorder (e.g., depression) 3) Common mental disorder for major depressive episode, panic disorder, PTSD, anxiety, and/or somatization 4) Substance use disorder for alcohol abuse or dependence and/or substance abuse or dependence  5) Suicide risk | Adults (patients and accompaniers) | 18 years and above | 458 | Portuguese | NR | NR | 52% | 470 | 0.94 (0.89-0.97) | 0.34 (0.28-0.40) | NR | NR | NR |
|  |  |  |  | mwTool - SMD |  |  |  |  |  |  |  | 29% | 260 | 0.89 (0.80-0.95) | 0.47 (0.42-0.53) |  |  |  |
|  |  |  |  | mwTool - CMD |  |  |  |  |  |  |  | 36% | 330 | 0.96 (0.91-0.98) | 0.83 (0.78-0.87) |  |  |  |
|  |  |  |  | mwTool - SUD |  |  |  |  |  |  |  | 14% | 124 | 0.86 (0.68-0.96) | 0.82 (0.78-0.86) |  |  |  |
|  |  |  |  | mwTool - SRi |  |  |  |  |  |  |  | 9% | 86 | 0.77 (0.60-0.90) | 0.93 (0.90-0.96) |  |  |  |
| 112 | Manzar, 2021(170)* | Mizan sleep quality and Sleep Hygiene Index MiSQuaSHI: a psychometric investigation | Ethiopia | MiSQuaSHI | Sleep quality and sleep hygiene practices | University Students | 21.9Â±4.2 years | 395 | English | 0.7 | 7.5 | NR | NR | 0.62 | 0.88 | NR | NR | 0.81 (0.76-0.85) |
| 113 | Pence, 2012(73) | Validity of an Interviewer-Administered Patient Health Questionnaire-9 to Screen for Depression in HIV-Infected Patients in Cameroon | Cameroon | PHQ-9 | Major depressive disorder (MDD) | HIV-positive patients on anti-retroviral therapy (ART) | 18-55 Years | 400 | English | NR | 10 | 3% | 11 | 0.27 (0.06-0.61) | 0.94 (0.91-0.96) | 0.12 | 0.98 | NR |
| 114 | Akanni, 2022(171)* | Validation of the Sleep Quality Questionnaire among senior students in Benin City, Nigeria | Nigeria | SQQ | insomnia | Junior and senior secondary school students | 10-21 years | 377 | English | 0.7 | 18 | NR | NR | 0.89 | 0.98 | 0.85 | 0.99 | NR |
| 115 | Belus, 2022(172) | Psychometric Validation of a Combined Assessment for Anxiety and Depression in Primary Care in Mozambique (CAD-MZ) | Mozambique | CAAD-PC-MZ - for MDD | Major depressive disorder (MDD) | Patients attending for antenatal, postpartum, or general outpatient consultations | 18 years and older | 502 | Mozambican Portuguese | 0.84 | 7 | 8.6% | 43 | 0.54 | 0.96 | NR | NR | 0.82(0.74-0.91) |
|  |  |  |  | CAAD-PC-MZ for GAD | Generalized anxiety disorder (GAD), and any psychopathology (PTSD, alcohol abuse, alcohol dependence, substance abuse, and substance dependence) |  | 19 years and older |  |  |  |  | 13.3% | 67 | 0.21 | 0.94 |  |  | 0.72 (0.65-0.78) |
|  |  |  |  | CAAD-PC-MZ for MDD or GAD | Major depressive disorder (MDD), generalized  anxiety disorder (GAD), and any psychopathology (PTSD, alcohol abuse, alcohol dependence, substance abuse, and substance dependence) |  | 20 years and older |  |  |  |  | 21.9% | 110 | 0.29 | 0.97 |  |  | 0.76 (0.70-0.81) |
| 116 | Habtamu, 2022(173)* | Psychometric properties of Alcohol Use Disorder Identification Test screening tool among medical outpatients in Dilla University Referral Hospital, southern Ethiopia, 2020 | Ethiopia | AUDIT (Women) | Alcohol use disorder | Medical outpatients | 18-63 years | 325 | Gedofa | NR | 8 | NR | NR | 0.92 | 0.87 | 0.70 | 0.97 | 0.96 (0.92-0.99) |
|  |  |  |  | AUDIT (Men) |  |  | 18-63 years |  |  |  | 10 | NR | NR | 0.91 | 0.84 | 0.73 | 0.95 | 0.93 (0.90-0.97) |
|  |  |  |  | AUDIT (Overall) |  |  | 18-63 years |  |  | 0.9 | 8 | NR | NR | 0.94 | 0.84 | 0.70 | 0.97 | 0.96 (0.92-0.99) |
| 117 | Kagee, 2022(174) | Predicting posttraumatic stress disorder caseness using the PTSD Checklist for DSM-5 among patients receiving care for HIV | South Africa | PCL-5 | Posttraumatic Stress Disorder (PTSD) | Patients receiving antiretroviral therapy (ART) | 18 years or older | 688 | English | 0.97 | 32 | 14.74% | 101 | 0.88 (0.80-0.94) | 0.88 (0.85-0.91) | 0.56 | 0.98 | 0.94(0.93-0.96) |
| 118 | Kaiser, 2022(38) | Challenges in simultaneous validation of mental health screening tools in multiple languages: Adolescent assessments in Hausa and Pidgin in Nigeria | Nigeria | DSRS | 1. Depression | Adolescents | 12-17 years | 330 | 1. Haussa 2. Pidgin | 1. DSRS: 0.77 | 8 | NR | 21 | 0.86 | 0.45 | 0.1 | 0.98 | 0.71 |
|  |  |  |  | CPSS | 2. PTSD |  |  |  |  | 2. CPSS: 0.73 | 13 | NR | 41 | 0.88 | 0.36 | 0.16 | 0.95 | 0.67 |
|  |  |  |  | DBDRS-ODD | 3. Oppositional-Deficient disorder (ODD) |  |  |  |  | 3. DBDRS-ODD: 0.69 | 11 | NR | 58 | 0.85 | 0.39 | 0.23 | 0.92 | 0.7 |
|  |  |  |  | DBDRS-CD | 4. Conduct disorder (CD) |  |  |  |  | 4. DBDRS-CD:0.82 | 2 | NR | 91 | 0.83 | 0.51 | 0.4 | 0.88 | 0.76 |
| 119 | Lovero, 2022(39) | Validation of brief screening instruments for internalizing and externalizing disorders in Mozambican adolescents | Mozambique | PHQ-A | Depression | Adolescents | 12-19 years | 485 | Portuguese | 1. PHQ-A: 0.80 | 8 | 8.5% | 41 | 0.78 | 0.8 | NR | NR | 0.85 (0.76-0.90) |
|  |  |  |  | GAD-7 | Anxiety |  |  |  |  | 2. GAD-7: 0.84 | 5 | 17.5% | 85 | 0.81 | 0.72 |  |  | 0.84 (0.79-0.89) |
|  |  |  |  | SDQ - Internalizing disorders | Internalizing disorders Depression / anxiety) |  |  |  |  | SDQ internalizing subscale: 0.73 | 10 | 19.6% | 95 | 0.76 | 0.74 |  |  | 0.80 (0.75-0.85) |
|  |  |  |  | SDQ -  Externalizing disorders | Externalized disorders (Conduct disorder, oppositional defiant disorder, and/or ADHD) |  |  |  |  | SDQ externalizing subscale: 0.65 | 9 | 7.4% | 36 | 0.78 | 0.59 |  |  | 0.72 (0.65-0.80) |
|  |  |  |  | SDQ | ADHD |  |  |  |  | NR | 9 | 3.3% | 16 | 0.75 | 0.57 |  |  | 0.70 (0.60–0.81) |
|  |  |  |  | SDQ | Disruptive Behavior Disorders |  |  |  |  | NR | 10 | 6.6% | 32 | 0.72 | 0.7 |  |  | 0.74 (0.67–0.82) |
| 120 | Mamah, 2022(175)* | Longitudinal and cross-sectional validation of the WERCAP screen for assessing psychosis risk and conversion | Kenya | pWERCAP Screen (3MO) | Psychosis risk | Adolescents and young adults (high psychosis scorers (≥30) on the WERCAP Screen and comparable number of participants: 0-29) | 14-25 years | 825 | NR | 1. p-WERCAP (3MO): 0.90 (n = 825) | >12 | NR | NR | 0.78 | 0.77 | 0.41 | 0.94 | 0.83 |
|  |  |  |  | pWERCAP Screen (12MO) | Psychosis risk |  | 14-25 years | 540 |  | 2. p-WERCAP (12MO): 0.91 (n = 540) | >32 | NR | NR | 0.71 | 0.74 | 0.24 | 0.96 | 0.79 |
| 121 | Moya, 2022(51) | Reliability and validity of depression anxiety stress scale (DASS)-21 in screening for common mental disorders among postpartum women in Malawi | Malawi | The adapted DASS-D subscale | Depression | Postpartum women | 24 ± 6.8 | 115 | Chichewa | 1.1.DASS-21: 0.74 1.2.DASS-D subscale: 0.66 | 1 | 11.3% | 13 | 0.69 | 0.75 | NR | NR | 0.76 (0.61-0.91) |
|  |  |  |  | The adapted DASS-A subscale | Anxiety |  |  |  |  | 1.3.DASS-A subscale: 0.29 | 1 | 14.8% | 17 | 0.53 | 0.76 |  |  | 0.6505 (0.52-0.79) |
|  |  |  |  | EPDS | Depression |  |  |  |  | 2. EDPS: 0.74 | 1 | 11.3% | 13 | 0.77 | 0.67 |  |  | 0.75 (0.60-0.89) |
| 122 | Abas, 2023(176) | Prevalence of post-traumatic stress disorder and validity of the Impact of Events Scale - Revised in primary care in Zimbabwe, a non-war-affected African country | Zimbabwe | IES-R | PTSD | Primary care center patients | 18 years and above | 264 | Shona | 0.95 | 47 | 23.9% | NA | 0.84(0.73-0.92) | 0.81(0.75-0.86) | NR | NR | 0.90 |
| 123 | Basaraba, 2023(177)* | Does It Matter What Screener We Use? A Comparison of Ultra-brief PHQ-4 and E-mwTool-3 Screeners for Anxiety and Depression Among People With and Without HIV | Mozambique | PHQ-2 (PLWIH) Depression | 1. Depression and 2. Anxiety | 1. People with HIV (PWH) | 18 years and over | 286 | Portuguese | NR | 1 | NR | NR | 0.74 (0.63-0.83) | 0.54 (0.47-0.61) | NR | NR | 0.66(0.60-0.73) |
|  |  |  |  | GAD-2 (PLWIH) Anxiety |  |  |  |  |  |  | 2 | NR | NR | 0.79 (0.49-0.95) | 0.43 (0.37-0.49) |  |  | 0.71 (0.55-0.87) |
|  |  |  |  | PHQ-4 (PLWIH) Anxiety |  |  |  |  |  |  | 3 | NR | NR | 0.79 (0.49-0.95) | 0.29 (0.24-0.35) |  |  | 0.72 (0.55-0.89) |
|  |  |  |  | PHQ-4 (PLWIH) Depression |  |  |  |  |  |  | 2 | NR | NR | 0.84(0.75-0.91) | 0.34 (0.28-0.41) |  |  | 0.69(0.62-0.76) |
|  |  |  |  | PHQ-2 (HIV- People) Depression |  | 2. People without HIV |  | 625 |  |  | 1 | NR | NR | 0.82 (0.77-0.87) | 0.54(0.49-0.59) |  |  | 0.74 (0.70-0.78) |
|  |  |  |  | PHQ-2 (HIV- People) Anxiety |  |  |  |  |  |  | 2 | NR | NR | 0.92 (0.81-0.98) | 0.42 (0.38-0.46) |  |  | 0.76(0.70-0.83) |
|  |  |  |  | PHQ-4 (HIV- People) Anxiety |  |  |  |  |  |  | 3 | NR | NR | 0.92 (0.81-0.98) | 0.29 (0.25-0.33) |  |  | 0.78 (0.71-0.85) |
|  |  |  |  | PHQ-4 (HIV- People) Depression |  |  |  |  |  |  | 2 | NR | NR | 0.92 (0.87-0.95) | 0.37 (0.32-0.42) |  |  | 0.77(0.74-0.81) |
| 124 | Bothe, 2023(178)* | Compulsive sexual behavior disorder in 42 countries: Insights from the International Sex Survey and introduction of standardized assessment tools | South Africa | CSBD-19 scale | Compulsive Sexual Behavior Disorder | Adult | 18-99 years | 1849 | NR | 1. CSBD-19 version: NR | 18 | NR | NR | 0.87 | 0.98 | 0.67 | overall= 0.97 | NR |
| 125 | Marlow, 2023(36) | Detecting Depression and Anxiety Among Adolescents in South Africa: Validity of the isiXhosa Patient Health Questionnaire-9 and Generalized Anxiety Disorder-7 | South Africa | PHQ-9 | 1.Depression | Adolescents in need of mental health support | 10 - 19 years | 302 | isiXhosa | NR | 10 | 7.6% | 23 | 0.91 | 0.76 | 0.24 | 0.99 | 0.88 ( 0.81-0.95) |
|  |  |  |  | GAD-7 | 2.Anxiety |  |  |  |  |  | 6 | 14.9% | 45 | 0.67 | 0.75 | 0.32 | 0.93 | 0.78 (0.71-0.85) |
| 126 | Mutiso, 2023(53) | Edinburgh Postnatal Depression Scale (EPDS) for screening for depression in the first year post delivery in a low-resourced rural setting in Kenya | Kenya | EPDS | Postnatal depression | Women in the first year post delivery | 18 - 44 years | 544 | Kamba | 0.85 | 11 | 14.5% | 79 | 0.81 (0.71-0.89) | 0.83 (0.79-0.86) | 0.44 | 0.96 | 0.87(0.84-0.89) |
| 127 | Tele, 2023(37) | Validation of the English and Swahili Adaptation of the Patient Health Questionnaire-9 for Use Among Adolescents in Kenya | Kenya | PHQ-9 - English Version | Major depressive episodes (MDE) | Adolescents | 10 - 19 years | 250 | English | 1. PHQ-9 (English version): 0.862 | 9 | 7.6% | 19 | 0.95 | 0.73 | 0.23 | 0.99 | 0.89 (0.84- 0.92) |
|  |  |  |  | PHQ-9 -Swahili version |  |  |  |  | 2. Swahili | 2. PHQ-9 (Swahili version: 0.834 | 9 | 7.6% | 19 | 0.89 | 0.7 | 0.2 | 0.9 | 0.87 (0.82-0.90) |
| 128 | Yusuf, 2023(179) | Psychometric properties of Hamilton Depression Rating Scale among people with epilepsy in Jimma University Medical Center, Neurology Clinic, Southwest Ethiopia, 2020: cross-sectional study | Ethiopia | HAMD-17 | Depression | Epilepsy patients | 18-63 years | 133 | Afaan Oromo | 0.74 | 9 | 27.8% | 37 | 0.92 (0.78-0.96) | 0.91 (0.81-0.96) | 0.81 | 0.96 | 0.97 (0.94-0.99) |

**Abbreviations / Acronyms:** ASSIST: The alcohol, smoking and substance involvement screening test, TSI: Total substance involvement , SSI: Specific Substance Involvement , AUDIT-C = consumption items (Items 1-3) from the AUDIT , AUDIT-3= one AUDIT item (Item 3), assessing binge drinking only , PSQ: Psychosis Screening Questionnaire, EPDS: Edinburgh postnatal depression scale ,PHQ-9: Patient Health Questionnaire-9, SCQ: Social Communication Questionnaire, 23Q: 23 Questions screener, SRS-2: Social responsiveness scale, Second Edition , Proxy mwTool-3: The Proxy Mental Wellness Tool-a three time screener, ECAS: Ethiopian Cognitive Assessment Battery for Schizophrenia , WLLT: World List Learning Test, DST: Digit Sequencing Task - verbal working memory, CBTT: Visual working memory , ANT: Animal Naming Test-Verbal learning, DSST: Digit Symbol Substitution Test, TMT Part A & SP: Trail Making Test for Attention part and speed of processing , TMT B (EF): Trail Making Test for Executive Function, CDQ: Client Diagnostic Questionnaire , MDQ: Mood Disorder Questionnaire , R-MDQ: Rwandese version of the Mood Disorder Questionnaire , CRAFFT: Car, Relax, Alone, Forget, Family/Friends, Trouble tool, mwTool-13: Mental Wellness Tool - thirteen items, CDM: Common mental disorder, MDE: Major Depressive Episode, GAD: General Anxiety Disorder, PTSD: Post Traumatic Stress Disorder, AUD: Alcohol Use Disorder, SUD: Substance Use Disorder, SMD: Severe Mental Disorder, PD: Psychotic Disorder, M/HE: Manic or Hypomanic Episode, SR: Suicidality Risk, GAD-7: a seven-time Generalized Anxiety Disorder scale, PC-PTSD-5: Primary Care Post Traumatic Stress Disorder Screen for DSM-5, GAD-2: a two-item Generalized Anxiety Disorder scale, PC-PTSD-5: Primary Care Post Traumatic Stress Disorder Screen for DSM-5, C-SSRS: Columbia Suicide Severity Rating Scale, PHQ-2: a two-item Patient Health Questionnaire , PHQ-2/9: Patient Health Questionnaire with two steps, starting with a 2-item PHQ and, if depression suspected, continue to the remaining 7 items of the PHQ , PHQ-4: a four-item Patient Health Questionnaire, PHQ-Q9: a one-item (question 9 only) Patient Health Questionnaire , SRi: Suicide Risk, GAD-2/7: Generalized Anxiety Disorder screening tool starting with two questions and, if anxiety suspected, continue with remaining 5 items of the GAD screening tool, C-SSRS: Columbia Suicide Severity Rating Scale , HSCL-25: Hopkins Symptom Checklist-25 , Minor DD: Minor Depression Disorder, BDI: Beck Depression Inventory , K10: Kessler Psychological Distress Scale-10 , K6: Kessler Psychological Distress Scale-6, SRQ: Self-reporting Questionnaire , CPDS: Child Psychosocial Distress Screener , CES-D: Center for Epidemiological Studies Depression Scale , HTQ: Harvard Trauma Questionnaire , ADD: Any depressive disorders, cMajor DD: Current Major Depressive Disorder, cMinor and cMajor DD: Current Minor and Major Depressive Disorder , PDS: Posttraumatic Diagnostic Scale, DHSCL: Depression section of the Hopkins Symptom Checklist , PSC: Pediatric Symptom Checklist, PSC-Y: Pediatric Symptom Checklist – youth self-report version , PSC-17-Y: a 17-item Pediatric Symptom Checklist – youth self-report version , PTSD-RI: Adapted trauma-focused mental health assessment tool - the UCLA Post-traumatic Stress Disorder - Reaction Index , PTSD-RI SS: PTSD-RI Symptom scale , PTSD-RI LSSS: PTSD-RI - Locally-specific symptom scale: , PTSD-RI – TSS: PTSD-RI - Total symptom scale, AVIDI: Akena visual depression inventory, CES-DC: Center for Epidemiological Studies Depression Scale for Children , CBQ: Child Behavior Questionnaire , RQC: Reporting Questionnaire for Children K-2: Shorter version of the Kessler-10 , CIDI: Structured Composite International Diagnostic Interview , PHST – Depression and Anxiety: Primary Healthcare Screening Tool to Identify Depression and Anxiety Disorders, EPDS-10: 10-item Edinburgh Postnatal Depression Scale , EPDS-7: 7-item traditional depression subscale of Edinburgh Postnatal Depression Scale , EPDS-5R: 5-item Edinburgh Postnatal Depression Scale, EPDS-3R: 3-item Edinburgh Postnatal Depression Scale, RFA Item 11: an 11-item risk factor assessment (RFA), PRI-3items (EPDS8, RFA1&11): Psychiatric referral items (3 items - EPDS8, RFA1&11- combined), SSQ: Shona Symptom Questionnaire , BDI-II: Beck Depression Inventory-II , CDI-II-S: Children Depression Inventory-II-Short, YCPS-R: Youth Conduct Problems Scale-Rwanda , YCPS-R SF: Youth Conduct Problems Scale-Rwanda Short Form, EPDS-2: a 2-item EPDS with two items analogue of the Patient Health Questionnaire, EPDS-3: a 3-item anxiety subscale of the EPDS, EPDS-5: a 5-item version of the depressive symptoms subscale of the EPDS, EPDS-7: a 7-item depressive symptoms subscale of the EPDS, DSRS: Depression Self-Rating Scale, CPSS: Child PSTD Symptom Scale, SCARED-41: a 41-item Screen for Child Anxiety Related Emotional Disorders, SAST: Sexual Addiction Screening Test , W-SAST: Women Sexual Addiction Screening Test, SRQ-SIB: Self-Report Questionnaire, Suicide Ideation and Behavior , MINI: Mini International Neuropsychiatric Interview questionnaire, CDI: Children Depression Inventory , PGQ: Prolonged Grief Questionnaire , DTI: Developmental Trauma Inventory , DTI-PTSD: Developmental Trauma Inventory for PTSD (DTI-PTSD), DTI-Complex PTSD: Developmental Trauma Inventory for Complex PTSD , SBQ-R-4: a 4-item Suicidal Behaviors Questionnaire-Revised (SBQ-R) CES-D-10: a 10-item Centre for Epidemiological Studies Depression Scale , Whooley – no help Q: Whooley excluding help question , Whooley – with help Q: Whooley including help question , CDQ: Client Diagnostic Questionnaire , AUDIT-QF = quantity and frequency items (Items 1 and 2) from the AUDIT , AUDIT-PC-5: AUDIT Primary Care using five AUDIT items (Items 1, 2, 4, 5, and 10) , AUDIT-4 = four AUDIT items (Items 1, 2, 3, and 10) , m-FAST- AUDIT-4 = modified Fast Alcohol Screening Test - four AUDIT items (Items 3, 5, 8, and 10) , PANSI: Positive and Negative Suicide Ideation Inventory, PANSI - NSI - PANSI Negative Suicide Ideation subscale , BHS: Beck Hopelessness Scale , HSCL: Hopkins Symptoms Checklist-15, PDEPS: Perinatal Depression Screening, LSEQ: Leeds Sleep Evaluation Questionnaire, LSEQ-M: The Adapted Leeds Sleep Evaluation Questionnaire Mizan , DBD-TRS: Disruptive behaviour disorders teacher rating scale , DBD - ADHD: Disruptive Behaviour Disorders - attention-deficit/hyperactivity disorder (DBD-ADHD), DBD - CD: Disruptive Behaviour Disorders – Conduct disorders , DBD-ODD: Disruptive behaviour disorders -Oppositional defiant disorder, BDI-I: Beck Depression Inventory-I, SHI: Sleep Hygiene Index , PCL-5: PTSD Checklist for DSM-5 , PLWHIV: People Living with HIV, WERCAP Screen: The Washington Early Recognition Center Affectivity and Psychosis Screen, aWERCAP screen: Affectivity (aWERCAP) Screen, pWERCAP screen: Psychosis (pWERCAP) Screen, IHDS: International HIV Dementia Scale, y-IHDS - major ND: Youth-International HIV Dementia Scale for major neurocognitive disorder, y-IHDS - minor ND: Youth-International HIV Dementia Scale for major neurocognitive disorder , y-IHDS – CI: Youth-International HIV Dementia Scale for major cognitive impairment , Whooley Q: Whooley questions, BOSHAS: Body Shape Scale , CBS: Current Body Shape, DBS: Desired Body Shape, IBS: Ideal Body Shape, PHQ-9-MZ: Patient Health Questionnaire-9 Mozambique , PHQ-2-MZ: Patient Health Questionnaire-2 Mozambique , HDRS: Hamilton Depression Rating Scale , SADS: South African Depression Scale, AUDIT-10-MZ: Alcohol Use Disorders Identification Test 10-Item –Mozambique, AUDIT-C-MZ: Alcohol Use Disorders Identification Test-Concise – Mozambique, CDST: Child Depression Screening Tool , PRS-SA: Patient-Reported Symptoms-South Africa Instrument, MiSQuaSHI: Mizan Sleep Quality and Sleep Hygiene Index , SQQ: Sleep Quality Questionnaire , CAAD-PC-MZ: A Combined Assessment for Anxiety and Depression in Primary Care in Mozambique , DBDRS-ODD: Disruptive Behavior Disorders Rating Scale -Oppositional Defiant Disorder Subscale , DBDRS-CD: Disruptive Behavior Disorders Rating Scale -Conduct Disorder Subscale, SDQ: Strengths and Difficulties Questionnaire , PHQ-A: Patient Health Questionnaire – Adolescents, pWERCAP Screen (3MO): Washington Early Recognition Center Affectivity and Psychosis (WERCAP) Screen within 3 months (3MO), pWERCAP Screen (12MO): Washington Early Recognition Center Affectivity and Psychosis (WERCAP) Screen within 12 months (12MO), DASS- D: Depression Anxiety Stress Scale - Depression Subscale (DASS- D), DASS- A: Depression Anxiety Stress Scale - Anxiety Subscale (DASS- A), IES-R: Impact of Events Scale-Revised , PHQ-2 (PLWIH) Depression: 2-item Patient Health Questionnaire for depression – People Living with HIV , GAD-2 (PLWIH) Anxiety: 2-item Patient Health Questionnaire for anxiety – People Living with HIV , PHQ-4 (PLWIH) Anxiety: 4-item Patient Health Questionnaire for anxiety – People Living with HIV, PHQ-4 (PLWIH) Depression: 4-item Patient Health Questionnaire for Depression – People Living with HIV, PHQ-2 (HIV- People) Depression: 2-item Patient Health Questionnaire for depression – HIV negative People, PHQ-2 (HIV- People) Anxiety: 2-item Patient Health Questionnaire for Anxiety – HIV negative People, PHQ-4 (HIV- People) Anxiety: 4-item Patient Health Questionnaire for anxiety – HIV negative People, PHQ-4 (HIV- People) Depression: 4-item Patient Health Questionnaire for depression – HIV negative People, CSBD-19 scale: Compulsive sexual behavior disorder-19 scale HAMD-17: Hamilton Depression Rating Scale 17-item

**Note:** *Studies that are not eligible for meta-analysis they neither reported actual positives nor prevalence per gold/reference standard
