# Supplementary material for: A systematic review and meta-analysis of diagnostic test accuracy of mental health screening tools applicable to adolescents in sub-Saharan Africa
Source: Front Psychiatry. 2026 Jun 16;17:1728252. doi: 10.3389/fpsyt.2026.1728252 (PMC13317013; doi:10.3389/fpsyt.2026.1728252)
Supplement: Supplementary file 3 [file Table3.docx]

**Supplementary appendix 3:** Number of tools validated per country

| **Country (number of validated tools)** | **Number of validated tools** | **Name of tool (condition: target population)-author, year (Reference #)** |
| --- | --- | --- |
| Botswana | 5 | 1. PSC (Psychosocial health: HIV positive children aged 8-16 years) – Lowenthal,2011(77), 2.PSC-17 (Psychosocial health: HIV positive children aged 8-16 years) – Lowenthal,2011(77), 3. PSC-Y (Psychosocial health: HIV positive children aged 8-16 years) – Lowenthal,2011(77), 4. PSC-17-Y (Psychosocial health: HIV positive children aged 8-16 years) – Lowenthal,2011(77), 5.PHQ-9 (Depression: Primary care attendants aged 18-79 years)- Molebatsi,2020(50) |
| Burkina Faso | 2 | 1. K10 (PND: Postpartum women aged 17-46 years) – Baggaley, 2007(100), 2. K6 (PND: Postpartum women aged 17-46 years ) – Baggaley, 2007(100) |
| Burundi | 4 | 1. CPDS (Psychosocial distress: Children aged 7-17 years) – Jordans, 2008(142), 2. (DSRS: Primary school children aged 10-15 years)- Ventevogel,2014(111), 3. CPSS (PTSD: Primary school children aged 10-15 years)- Ventevogel,2014(111), 4. SCARED-41 (Anxiety disorders: Primary school children aged 10-15 years)- Ventevogel,2014(111) |
| Cameroon | 11 | 1. EPDS (Perinatal depression: Adolescent mothers aged 12-20 years) – Djatche Miafo,2024(136), 2. PHQ-9 (MDD: HIV-positive patients aged 18-55 years) – Pence,2021, (135), 3. BOSHAS - Body weight Self-satisfaction ^iv^ (Body image disorders: NR age range of 18-75 years) – Cohen,2020(134), 4. BOSHAS - Normal body- CBS ^v^ (Body image disorders: NR age range of 18-75 years) – Cohen,2020(134), 5. BOSHAS - Normal body- self-satisfaction vi (Body image disorders: NR age range of 18-75 years) – Cohen,2020 (134), 6. BOSHAS - Importance of appearance-Modern DBS ^vii^ (Body image disorders: NR age range of 18-75 years) – Cohen,2020 (134), 7. BOSHAS - Importance of appear-Modern IBS ^viii^ (Body image disorders: NR age range of 18-75 years) – Cohen,2020(134), 8. BOSHAS -Importance of appearance-Modern Self-self-satisfaction ^ix^ (Body image disorders: NR age range of 18-75 years) – Cohen,2020(134), 9. BOSHAS - Modern Aesthetic criteria-Modern DBS ^x^ (Body image disorders: NR age range of 18-75 years) – Cohen,2020(134), 10. BOSHAS - Modern Aesthetic criteria-Modern IBS ^xi^ (Body image disorders: NR age range of 18-75 years) – Cohen,2020(134), 11. BOSHAS - Modern Aesthetic criteria-Modern self-satisfaction ^xii^ (Body image disorders: NR age range of 18-75 years) – Cohen,2020(134) |
| Eritrea | 3 | 1.SRQ-20 (CMD: Outpatients aged 18-65 years at health facilities) – Netsereab,2018(153), 2.SRQ-20 (CMD: Male outpatients aged 18-65 years at health facilities) – Netsereab,2018(153), 3.SRQ-20 (CMD: Female outpatients aged 18-65 years at health facilities) – Netsereab,2018(153) |
| Ethiopia | 30 | 1. ECAS-WLLT (CI: Schizophrenia outpatients aged 18-65 years) – Gebreegziabhere,2024(138), 2. ECAS-DST (CI –DST-verbal working memory: Schizophrenia outpatients aged 18-65 years) – Gebreegziabhere,2024(138), 3. ECAS-CBTT (CI- visual working memory: Schizophrenia outpatients aged 18-65 years) – Gebreegziabhere,2024(138), 4. ECAS-ANT (CI- Animal Naming Test-Verbal learning: Schizophrenia outpatients aged 18-65 years) – Gebreegziabhere,2024(138, 5. ECAS-DSST (CI- Digit Symbol Substitution Test: Schizophrenia outpatients aged 18-65 years) – Gebreegziabhere,2024(138), 6. ECAS-TMT A & SP (CI- [attention] and SP [Speed of Processing] (Trail Making Test)): Schizophrenia outpatients aged 18-65 years) – Gebreegziabhere,2024(138), 7. ECAS-TMT B EF (CI-Executive execution: Schizophrenia outpatients aged 18-65 years) – Gebreegziabhere,2024(138), 8. ECAS (CI-Overall: Schizophrenia outpatients aged 18-65 years) – Gebreegziabhere,2024(138), 9.EPDS (CMD: postnatal women aged ≥15 years) – Hanlon,2008 (32), 10. SRQ-20 - Study 1 (CMD: perinatal women aged ≥15 years) – Hanlon,2008(32), 11. SRQ-20 - Study 2 (CMD: perinatal women aged ≥15 years) – Hanlon,2008(32), 12. EPDS (Postnatal CMD: Postnatal women aged 18-38 years)-Tesfaye,2010(33), 13. K6(Postnatal CMD: Postnatal women aged 18-38 years)-Tesfaye,2010(33), 14.K10(Postnatal CMD: Postnatal women aged 18-38 years)-Tesfaye,2010(33), 15. PHQ-9 (MDD: Adult patients aged 18-69 years)- Gelaye,2013(43), 16. CIDI (MDD: Adult patients aged 18-69 years)- Gelaye,2013(43), 17. PHQ-9 (MDD: Patients aged ≥18 years attending the PHC facilities)- Hanlon, 2015(44), 18. PHQ-9 (MDD: Patients aged ≥18 years attending the PHC facilities)- Hanlon, 2015(44), 19. SRQ20 (MDD: Patients aged ≥18 years attending the PHC facilities)- Hanlon, 2015(44), 20. K10 (MDD: Patients aged ≥18 years attending the PHC facilities)- Hanlon, 2015(44), 21. K6 (MDD: Patients aged ≥18 years attending the PHC facilities)- Hanlon, 2015(44), 22. PHQ-2 (MDD: Adult outpatients aged 18-69 years) – Gelaye,2016(120), 23. LSEQ-M (Insomnia: University students aged 21.87 ± 4.13 years)-Manzar,2018(115), 24. PHQ-9 (Depression: Pregnant women aged 18 - 40 years) – Woldetensay,2018(45), 25. PHQ9 (MDE: Cancer patients aged ≥18 years) – Degefa,2020(131), 26. MiSQuaSHI (Sleep quality and sleep hygiene practices: University students aged 21.9±4.2 years)- Manzar,2021(163), 27.AUDIT (Alcohol use disorder: Female medical students aged 18-63 years)-Habtamu,2022(165), 28. AUDIT (Alcohol use disorder: Medical students aged 18-63 years)-Habtamu,2022(165), 29. AUDIT (Alcohol use disorder: male medical students aged 18-63 years)-Habtamu,2022(165), 30. HAMD-17 (Depression: Epilepsy patients aged 18-63 years)- Yusuf,2023(119) |
| Ghana | 3 | 1. SRQ-20 (Psychological distress: Women aged 15-46 years within postnatal period 5 and 11 weeks) – Weobong,2008(123), 2. EPDS (Depression, anxiety and panic and thoughts of self-harm: Women aged 15-46 years within postnatal period 5 and 11 weeks) – Weobong,2008(123), 3. PHQ-9 (Depression: Women aged 15-46 years within postnatal period 5 and 11 weeks)- Weobong,2008(123) |
| Kenya | 14 | 1. CDQ^ii^ (MDD, Alcohol use, Panic disorder, anxiety, drug abuse, PTSD, and psychosis) – Kwobah,2024(96), 2. PHQ-2 (MDD: PLWHIV aged 18-61 years) – Monahan,2009(74), 3. PHQ-2 (ADD: PLWHIV aged 18-61 years) – Monahan,2009(74), 4. EPDS –two weeks (MDE: Pregnant women and new mothers aged ≥18 years) – Green,2018(38), 5.EPDS – one week (MDE: Pregnant women and new mothers aged ≥18 years) – Green,2018(38), 6. PHQ-9 (MDD: Pregnant women and new mothers aged ≥18 years) – Green,2018(38), 7. PDEPS (Pregnant women and new mothers aged ≥18 years) – Green,2018(38), 8. aWERCAP Screen (Affectivity: Adults aged 18-90 years with positive screens on the WHO mhGAP-IG)- Ndetei,2019(156), 9. pWERCAP Screen (Psychosis: Adults aged 18-90 years with positive screens on the WHO mhGAP-IG)- Ndetei,2019(156), 10. pWERCAP Screen 3MO (Psychosis risk: Adolescents and young adults age 14-25 years)- Mamah,2022(168), 11. pWERCAP Screen 12MO (Psychosis risk: Adolescents and young adults age 14-25 years)- Mamah,2022(168), 12. EPDS (Postnatal depression: Women aged 18 - 44 years in the first year post-delivery)- Mutiso,2023(172), 13. PHQ-9 English (MDE: Adolescents aged 10-19 years)- Tele,2023(47), 14. PHQ-9 Swahili (MDE: Adolescents aged 10-19 years)- Tele,2023(47) |
| Malawi | 11 | 1. SRQ (cMajor DD: Mothers/infants aged 16-18 years attending a child health clinic) – Stewart,2009(122), 2. SRQ (cMinor and cMajor DD: Mothers/infants aged 16-18 years attending a child health clinic)- Stewart,2009(122), 3. BDI-II (Depression: HIV-positive adolescents aged 12-18 years) – Kim,2014(66), 4. CDI-II-S (Depression: HIV-positive adolescents aged 12-18 years)- Kim,2014(66), 5. EPDS (Depression: Pregnant women aged ≥18 years)- Chorwe-Sungani,2018(36), 6. HSCL-15 (Depression: Pregnant women aged ≥18 years)- Chorwe-Sungani,2018(36), 7-SRQ (Depression: Pregnant women aged ≥18 years)- Chorwe-Sungani,2018(36), 8. 3-item screener (Depression: Pregnant women aged ≥18 years)- Chorwe-Sungani,2018(36), 9. Adapted DASS-D subscale (Depression: Postpartum women aged 24 ±6.8 years)- Moya,2022(37), 10. Adapted DASS-A subscale (Anxiety: Postpartum women aged 24 ±6.8 years)- Moya,2022(37), 11. EPDS (Depression: Postpartum women aged 24 ±6.8 years)- Moya,2022(37) |
| Mali | 1 | SCQ (Autism spectrum disorders: Patients aged 4-20 years old diagnosed with ASD) – Sangare,2019(157) |
| Mozambique | 30 | 1. Proxy mwTool-3^i^ (SMD and CMD: Patients and accompaniers aged 18-78 years)- Stockton,2023(133), 2. PSQ (Psychosis: Adults aged ≥18 years in general healthcare setting)- Lovero,2024(104), 3. PSQ (Mania: Adults aged ≥18 years in general healthcare setting)- Lovero,2024(104), 4. PHQ-9-MZ (MDD: Primary care patients aged ≥18 years)- Cumbe,2020(49), 5. PHQ-2-MZ (MDD: Primary care patients aged ≥18 years)- Cumbe,2020(49), 6. AUDIT-10-MZ (alcohol use, alcohol dependence, drug dependence and drug use: Antenatal, postpartum and outpatient clinics aged 18- 29 years)- Atkins,2021(160), 7. AUDIT-C-MZ (alcohol use, alcohol dependence, drug dependence and drug use: Antenatal, postpartum and outpatient clinics aged 18- 29 years)- Atkins,2021(160), 8. mwTool (Any disorder: patients and accompaniers aged ≥18 years)- Lovero,2021(162), 9. mwTool (SMD: patients and accompaniers aged ≥18 years)- Lovero,2021(162), 10. mwTool (CMD: patients and accompaniers aged ≥18 years)- Lovero,2021(162), 11.mwTool (SUD: patients and accompaniers aged ≥18 years)- Lovero,2021(162), 12. mwTool (SRi: patients and accompaniers aged ≥18 years)- Lovero,2021(162), 13. CAAD-PC-MZ (MDD: Patients ≥18 years attending for antenatal, postpartum, or general outpatient consultations)- Belus,2022(117), 14. CAAD-PC-MZ (GAD: Patients ≥18 years attending for antenatal, postpartum, or general outpatient consultations)-Belus,2022 (117), 15. CAAD-PC-MZ (MDD or GAD: Patients ≥18 years attending for antenatal, postpartum, or general outpatient consultations)-Belus,2022(117), 16. PHQ (Depression: Adolescents aged 12-19 years)- Lovero (2022), 17. GAD-7 (Anxiety: Adolescents aged 12-19 years)- Lovero,2022(167), 18. SDQ (Internalized disorders [Depression or anxiety]: Adolescents aged 12-19 years)- Lovero,2022(167), 19. SDQ (Externalized disorders [Conduct disorder, oppositional defiant disorder, and/or ADHD]: Adolescents aged 12-19 years)- Lovero,2022(167), 20. SDQ (ADHD: Adolescents aged 12-19 years) - Lovero,2022(167), 21. SDQ (Disruptive Behavior Disorders: Adolescents aged 12-19 years) - Lovero,2022(167), 22. PHQ-2 (Depression: PLWHIV aged ≥18 years)- Basaraba,2023(169), 23. GAD-2 (Anxiety: PLWHIV aged ≥18 years)- Basaraba,2023(169), 24. PHQ-4 (Anxiety: PLWHIV aged ≥18 years)- Basaraba,2023(169), 25. PHQ-4 (Depression: PLWHIV aged ≥18 years)- Basaraba,2023(169), 26. PHQ-2 (Depression: HIV negative people aged ≥18 years)- Basaraba,2023(169), 27. PHQ-2 (Anxiety: HIV negative people aged ≥18 years)- Basaraba,2023(169), 28.PHQ-4 (Anxiety: HIV negative people aged ≥18 years)- Basaraba,2023(169), 29. PHQ-4 (Depression: HIV negative people aged ≥18 years)- Basaraba,2023(169), 30. mwTool-3 (SMD, CMD or AUD: patients and accompaniers)- Stockton,2023(133) |
| Namibia | 6 | 1. AUDIT-C (Harmful drinking: General population aged 18-80 years)- Seth,2015(99), 2. AUDIT-C(Harmful drinking: Men aged 18-80 years)- Seth,2015(99), 3. AUDIT-C(Harmful drinking: Women aged 18-80 years)- Seth,2015(99), 4. AUDIT-3 (Harmful drinking: General population aged 18-80 years)- Seth,2015(99), 5. AUDIT-3 (Harmful drinking: Men aged 18-80 years)- Seth,2015(99),6. AUDIT-3 (Harmful drinking: Women aged 18-80 years)- Seth,2015(99) |
| Nigeria | 31 | 1. SCQ (ASD: Adolescents aged 11-26 years suspected of having ASD)- Nwokolo,2024(141), 2. EPDS (Depression: Postpartum women aged 18-39 years)- Uwakwe,2003(98), 3. AUDIT (Alcohol hazardous use: University students aged 22.5 ± 5.2 years) – Adewuya,2005(53), 4.AUDIT (Alcohol harmful use: University students aged 22.5 ± 5.2 years) – Adewuya,2005(53), 5. AUDIT (Alcohol dependence: University students aged 22.5 ± 5.2 years) – Adewuya,2005(53), 6. PHQ-9 (Minor DD: University students aged 15-40 years)- Adewuya,2006(53), 7. PHQ-9 (MDD: University students aged 15-40 years)- Adewuya,2006(53), 8. EDDS (Major and Minor DD: Women aged 24.89 (SD= 6.41) in late pregnancy; 32 weeks and above)- Adewuya,2006(53), 9. EDDS (Major depression only: Women aged 24.89 (SD= 6.41) in late pregnancy; 32 weeks and above) – Adewuya,2006(53), 10. BDI (MDD: Secondary school students aged 13-18 years)- Adewuya, 2007(73), 11. CBQ (Psychiatric disorders: Children aged 7-14 years and their accompanying mothers attending hospital)- Tunde-Ayinmode, 2012(78), 12. RQC (Psychiatric disorders: Children aged 7-14 years and their accompanying mothers attending hospital)- Tunde-Ayinmode, 2012(78), 13. K6 (Depression: Hospital outpatients aged ≥18 years)- Makanjuola,2014(81), 14. K6 (Anxiety: Hospital outpatients aged ≥18 years)- Makanjuola,2014(81), 15. GHQ12 (Depression: Hospital outpatients aged ≥18 years)- Makanjuola,2014(81), 16. GHQ12 (Anxiety: Hospital outpatients aged ≥18 years)- Makanjuola,2014(81), 17. SAST (Sexual addiction: Stable psychiatriac inpatients and outpatients - aged ≥18 years)- Abdullahi,2015(112), 18. W-SAST (Sexual addiction: Stable female psychiatriac inpatients and outpatients - aged ≥18 years)-Abdullahi,2015(112), 19. SBQ-R-4 (Suicide risk: undergraduate students aged 18-31 years)- Aloba, 2017(84), 20. CRAFFT (SRD: Adolescents aged 13-17 years in correctional centers) – Ola,2017(130), 21. PANSI-NSI (Under-graduate university students aged 16-35 years) – Aloba,2018(151), 22. BHS (High suicide risk: Undergraduate students aged 18 to 29 years)- Aloba,2018(152), 23. DBD (ADHD: Children/Adolescents aged 4-16 years) – Olagundoye,2018(103), 24.DBD (CD: Children/Adolescents aged 4-16 years) – Olagundoye,2018(103), 25. DBD (ODD: Children/Adolescents aged 4-16 years) – Olagundoye,2018(103), 26. SHI (Poor sleep quality: Undergraduate Students aged 21.60 ± 2.87 years) - Seun-Fadipe,2018(155), 27. SQQ (insomnia: Junior and senior secondary school students aged 10-21 years) – Akanni,2022(164), 28. DSRS (Depression: Adolescents aged 12-17 years) – Kaiser,2022(93), 29. CPSS (PTSD: Adolescents aged 12-17 years) – Kaiser,2022(93), 30. DBDRS (ODD: Adolescents aged 12-17 years) – Kaiser,2022(93), 31. DBDRS (CD: Adolescents aged 12-17 years) – Kaiser,2022(93). |
| Rwanda | 19 | 1. R-MDQ (Bipolar disorder: Outpatients aged ≥18 years diagnosed with either bipolar disorder or unipolar major depression) - Musoni-Rwililiza,2024(139), 2. SRQ-20 (MD: Community members aged 16-74 years)- Scholte (2011), 3. SRQ-20 (MD: Male community members aged 16-74 years)- Scholte,2011(108), 4. SRQ-20 (MD: Female community members aged 16-74 years)- Scholte,2011(108), 5. CES-DC (Depression: Children and adolescents aged 10-17 years) – Betancourt,2012(109), 6. YCPS-R (CD: Youth aged 10-17 years) – Ng,2014(110), 7. YCPS-R (CD: Female care givers) – Ng, 2014(110), 8. YCPS-R (CD: (Male caregivers) – Ng,2014(110), 9. YCPS-R SF (CD: Youth aged 10-17 years) – Ng,2014(110), 10. YCPS-R SF (CD: Female caregivers) – Ng,2014(110), 11. YCPS-R SF (CD: Male caregivers) – Ng,2014(110), 12. SRQ-SIB (CMD: Congolese refugee women aged 15-49 years)- Bell, 2015(101), 13. CDI (Depression: Children aged 7-14 years living with HIV) – Binagwaho, 2016(146), 14. PGQ (Prolonged Grief: Parentally bereaved adolescents aged 14-18 years) – Unterhitzenberger, 2016(147), 15. HDRS (Depression: Patients with depression and healthy volunteers aged ≥18 years) – Dedeken,2020(158), 16. PHQ-9 (Mild depression: PwE aged ≥15 years)- Sebera,2020(51), 17. PHQ-9 (Moderate depression: PwE aged ≥15 years) – Sebera,2020(51), 18. PHQ-9 (Severe depression: PwE aged ≥15 years) – Sebera,2020(51), 19. CDST (MDD: Children 7-14 years living with HIV) – Binagwaho,2021(161) |
| Senegal | 9 | 1. BOSHAS - Body weight Self-satisfaction ^iv^ (Body image disorders: NR age range of 18-75 years) – Cohen,2020(134), 2. BOSHAS - Normal body- CBS ^v^ (Body image disorders: NR age range of 18-75 years) – Cohen,2020(134), 3. BOSHAS - Normal body- self-satisfaction ^vi^ (Body image disorders: NR age range of 18-75 years) - Cohen2020(134), 4. BOSHAS - Importance of appearance-Modern DBS ^vii^ (Body image disorders: NR age range of 18-75 years) – Cohen,2020(134), 5. BOSHAS - Importance of appear-Modern IBS ^viii^ (Body image disorders: NR age range of 18-75 years) – Cohen,2020(134), 6. BOSHAS -Importance of appearance-Modern Self-self-satisfaction ^ix^ (Body image disorders: NR age range of 18-75 years) – Cohen,2020(134), 7. BOSHAS - Modern Aesthetic criteria-Modern DBS ^x^ (Body image disorders: NR age range of 18-75 years) – Cohen,2020(134), 8. BOSHAS - Modern Aesthetic criteria-Modern IBS ^xi^ (Body image disorders: NR age range of 18-75 years) – Cohen,2020(134), 9. BOSHAS - Modern Aesthetic criteria-Modern self-satisfaction ^xii^ (Body image disorders: NR age range of 18-75 years) – Cohen,2020(134). |
| South Africa | 178 | 1. PSQ (current SMD: Patients and accompaniers aged 18-88 years at PTF) – Stockton,2023(95), 2. PSQ (current PD: Patients and accompaniers aged 18-88 years at PTF) – Stockton,2023(95), 3. PSQ (current M/HE: Patients and accompaniers aged 18-88 years at PTF) – Stockton,2023(95), 4. PSQ (Life SMD: Patients and accompaniers aged 18-88 years at PTF) – Stockton,2023(95), 5. PSQ (Life PD: Patients and accompaniers aged 18-88 years at PTF) – Stockton,2023(95), 6. PSQ (Life M/HE: Patients and accompaniers aged 18-88 years at PTF) – Stockton,2023(95), 7. Proxy mwTool-3^i^ Portuguese, isiXhosa or English (SMD, CMD, AUD, SUD and SRi: patients and accompaniers at HF aged 18-78) – Stockton,2023(133), 8. Proxy mwTool-3 isiXhosa version (SMD, CMD, AUD, SUD and SRi: patients and accompaniers aged 18-78) – Stockton,2023(133), 9. Proxy mwTool-3 isiXhosa or English (SMD, CMD, AUD, SUD and SRi: patients and accompaniers at HF aged 18-78) – Stockton,2023(133), 10. mwTool-13 for any disorder subscale (CDM, AUD, SUD, SMD or SRi: patients and accompaniers at HF aged 18-88) Stockton,2024(97), 11. mwTool-13 for CDM subscale (CDM: patients and accompaniers at HF aged 18-88) Stockton,2024(97), 12. mwTool-13 with question of unspecified disorder (CDM: patients and accompaniers at HF aged 18-88) – Stockton,2024(97), 13. mwTool-13 without question of unspecified disorder (CDM: patients and accompaniers at HF aged 18-88) – Stockton,2024(97), 14. mwTool-13 for MDE sub-scale (MDE: patients and accompaniers at HF aged 18-88) – Stockton,2024(97), 15. mwTool-13 for GAD sub-scale (GAD: patients and accompaniers at HF aged 18-88) – Stockton,2024(97), 16. mwTool-13 for PTSD sub-scale: (PTSD: patients and accompaniers at HF aged 18-88) – Stockton,2024(97), 17. mwTool-13 for AUD sub-scale (AUD: patients and accompaniers at HF aged 18-88)-Stockton,2024(97), 18. mwTool-13 for SUD sub-scale (SUD: patients and accompaniers at HF aged 18-88)-Stockton,2024(97), 19. mwTool-13 for SMD sub-scale ( SMD: patients and accompaniers at HF aged 18-88)-Stockton,2024(97), 20. mwTool-13 for PD sub-scale (PD: patients and accompaniers at HF aged 18-88)-Stockton,2024(97), 21. mwTool-13 for M/HE sub-scale (M/HE: patients and accompaniers at HF aged 18-88)-Stockton,2024(97), 22. mwTool-13 for SRi sub-scale (SRi: patients and accompaniers at HF aged 18-88)-Stockton,2024(97), 23.PHQ-2 (Depression: patients and accompaniers at HF aged 18-88)-Stockton.2024(40), 24. PHQ-9 with cut off of 5 (Depression: patients and accompaniers at HF aged 18-88)-Stockton,2024(40), 25. PHQ-9 with cut off of 10 (Depression: patients and accompaniers at HF aged 18-88)-Stockton,2024(40), 26. PHQ-2/9 with cut off of 5 (Depression: patients and accompaniers at HF aged 18-88)-Stockton,2024(40), 27. PHQ-2/9 with cut off of 10 (Depression: patients and accompaniers at HF aged 18-88)-Stockton,2024(40), 28. PHQ-4 for depression (Depression: patients and accompaniers at HF aged 18-88)-Stockton,2024(40), 29. PHQ-4 for anxiety (Anxiety: patients and accompaniers at HF aged 18-88)-Stockton,2024(40), 30. PHQ-4 for anxiety or panic (Anxiety or panic: patients and accompaniers at HF aged 18-88)-Stockton,2024(40), 31. PHQ-4 for anxiety or depression (Anxiety or depression: patients and accompaniers at HF aged 18-88)-Stockton,2024(40), 32. PHQ-4 for PTSD (PTSD: patients and accompaniers at HF aged 18-88)-Stockton,2024(40), 33. PHQ-4 for any CMD (CMD: patients and accompaniers at HF aged 18-88)-Stockton,2024(40), 34. PHQ-Q9 Low risk of SRi (Low risk of SRi: patients and accompaniers at HF aged 18-88)-Stockton,2024(40), 35. PHQ-Q9 High risk of SRi (High risk of SRi: patients and accompaniers at HF aged 18-88)-Stockton,2024(40), 36. GAD-2 for Anxiety (Anxiety: patients and accompaniers at HF aged 18-88)-Stockton,2024(40), 37. GAD-7 for Anxiety for cut off of 5 (Anxiety: patients and accompaniers at HF aged 18-88)-Stockton,2024(40), 38. GAD-7 for Anxiety for cut off of 10 (Anxiety: patients and accompaniers at HF aged 18-88)-Stockton,2024(40), 39. GAD-2/7 for Anxiety with cut off of 5 (Anxiety: patients and accompaniers at HF aged 18-88)-Stockton,2024(40), 40. GAD-2/7 for Anxiety with cut off of 10 (Anxiety: patients and accompaniers at HF aged 18-88)-Stockton,2024(40), 41. GAD-2 for Anxiety or panic (Anxiety or panic: patients and accompaniers at HF aged 18-88)-Stockton,2024 (40), 42. GAD-7 for Anxiety or panic with cut off of 5 (Anxiety or panic: patients and accompaniers at HF aged 18-88)-Stockton,2024(40), 43. GAD-7 for Anxiety or panic with cut off of 10 (Anxiety or panic: patients and accompaniers at HF aged 18-88)-Stockton,2024(40), 44. GAD-2/7 for Anxiety or panic with cut off of 5 (for Anxiety or panic: patients and accompaniers at HF aged 18-88)-Stockton,2024(40), 45. GAD-2/7 for Anxiety or panic with cut off of 10 (for Anxiety or panic: patients and accompaniers at HF aged 18-88)-Stockton,2024(40), 46. PC-PTSD-5 (PTSD: patients and accompaniers at HF aged 18-88)-Stockton,2024(40), 47. C-SSRS for Low Risk SRi (Low Risk SRi: patients and accompaniers at HF aged 18-88)-Stockton,2024(40), 48. C-SSRS for Low Risk SRi (High Risk SRi: patients and accompaniers at HF aged 18-88)-Stockton,2024(40), 49. CES-D - Afrikaans, Xhosa and English (Depression: HIV-infected individuals aged 18 - 65 years) - Myer,2008(121), 50. HTQ - Afrikaans, Xhosa and English (PTSD: HIV-infected individuals aged 18 - 65 years) - Myer2008(121), 51. AUDIT - Afrikaans, Xhosa and English (Alcohol dependence/abuse: HIV-infected individuals aged 18 - 65 years) – Myer,2008 (121), 52. CES-D – Afrikaans (Depression: HIV-infected individuals aged 18 - 65 years) - Myer2008(121), 53. HTQ – Afrikaans (PTSD: HIV-infected individuals aged 18 - 65 years) – Myer,2008(121), 54. AUDIT – Afrikaans (Alcohol dependence/abuse: HIV-infected individuals aged 18 - 65 years) – Myer,2008(121), 55. CES-D – Xhosa (Depression: HIV-infected individuals aged 18 - 65 years) – Myer,2008(121), 56. HTQ – Xhosa (PTSD: HIV-infected individuals aged 18 - 65 years) - Myer (2008), 57. AUDIT – Xhosa (Alcohol dependence/abuse: HIV-infected individuals aged 18 - 65 years) – Myer,2008(121), 58. K-10 - Current MDE (Current MDE: HIV-infected adults aged 16-65 years) – Spies,2010(75), 59. K-10 -Past MDE (Past MDE: HIV-infected adults aged 16-65 years) – Spies,2010(75), 60. K-10 Mood disorders (Mood disorders: HIV-infected adults aged 16-65 years) – Spies,2010(75), 61. K-10 –Agoraphobia (Agoraphobia: HIV-infected adults aged 16-65 years) – Spies,2010(75), 62. K-10 for GAD (GAD: HIV-infected adults aged 16-65 years) - Spies,2010(75), 63. K-10 for Social Phobia (Social Phobia: HIV-infected adults aged 16-65 years) - Spies,2010(75), 64. K-10 for Panic disorder (Panic disorder: HIV-infected adults aged 16-65 years) - Spies,2010(75) , 65. K-10 (PTSD: HIV-infected adults aged 16-65 years) - Spies,2010(75), 66. K10 (Depression: General population aged 18-92 years) – Andersen,2011(91), 67. K6 (Anxiety: General population aged 18-92 years) – Andersen,2011(91), 68. EPDS-10 (Antenatal depression: Pregnant women aged 16-40 years in their second trimester) – Rochat,2013(144), 69. EPDS-7 (Antenatal depression: Pregnant women aged 16-40 years in their second trimester) – Rochat,2013(144), 70. EPDS-5R (Antenatal depression: Pregnant women aged 16-40 years in their second trimester) – Rochat,2013(144), 71. EPDS-3R (Antenatal depression: Pregnant women aged 16-40 years in their second trimester) – Rochat,2013(144), 72. EPDS Item 8 (psychological distress-Depression or anxiety disorders: Pregnant women ≥ 18 years during their first or second antenatal visit) – Vythilingum,2013(80), 73. RFA Item (psychological distress-Depression or anxiety disorders: Pregnant women ≥ 18 years during their first or second antenatal visit) – Vythilingum,2013(80), 74. RFA Item 11 (psychological distress-Depression or anxiety disorders: Pregnant women ≥ 18 years during their first or second antenatal visit) – Vythilingum,2013(80), 75. PRI-3items- EPDS8, RFA1&11 (psychological distress-Depression or anxiety disorders: Pregnant women ≥ 18 years during their first or second antenatal visit)- Vythilingum,2013(80), 76. EPDS-study1 (antenatal depression: Pregnant women aged 18-42 years) –Tsai,2014(127), 77. EPDS-3 study 1 (antenatal depression: Pregnant women aged 18-42 years) –Tsai,2014(127), 78. EPDS-5 study 1 (antenatal depression: Pregnant women aged 18-42 years) –Tsai,2014(127), 79. EPDS-7 study 1 (antenatal depression: Pregnant women aged 18-42 years) –Tsai,2014(127), 80. EPDS-study2 (antenatal depression: Pregnant women aged 18-43 years) –Tsai,2014(127), 81. EPDS-3 study 2 (antenatal depression: Pregnant women aged 18-43 years) –Tsai,2014(127), 82. EPDS-5 study 2 (antenatal depression: Pregnant women aged 18-43 years) –Tsai,2014(127), 83. EPDS-7 study 2 (antenatal depression: Pregnant women aged 18-43 years) –Tsai,2014(127), 84. PHQ-9 (MDD: Chronic care –HIV, HT, diabetes -patients aged 18-88 years) – Bhana,2015(41), 85. DTI-PTSD (PTSD: Adolescents aged 12-20 years) – Valjee, 2016(148), 86. DTI-Complex PTSD (Complex PTSD: Adolescents aged 12-20 years) – Valjee, 2016(148), 87. ASSIST-TSI (Total substance use / abuse: Patients aged ≥ 18 years visiting emergency centers for injuries related to alcohol or drug use) – Van der Westhuizen,2016(149), 88. ASSIST-TSI (Total substance abuse / dependence: Patients aged ≥ 18 years visiting emergency centers for injuries related to alcohol or drug use) – Van der Westhuizen,2016(149), 89. The ASSIST - SSI- Alcohol use / abuse (Alcohol use disorder use / abuse: Patients aged ≥ 18 years visiting emergency centers for injuries related to alcohol or drug use) – Van der Westhuizen,2016(149), 90. ASSIST -SSI- Alcohol - abuse /dependence (Alcohol use disorder use / abuse: Patients aged ≥ 18 years visiting emergency centers for injuries related to alcohol or drug use) – Van der Westhuizen,2016(149), 91. ASSIST - SSI- Illicit drugs - use / abuse (Illicit drugs - use / abuse: Patients aged ≥ 18 years visiting emergency centers for injuries related to alcohol or drug use) – Van der Westhuizen,2016(149), 92. ASSIST - Illicit drugs - abuse / dependence (Illicit drug abuse / dependence: Patients aged ≥ 18 years visiting emergency centers for injuries related to alcohol or drug use) – Van der Westhuizen,2016(149), 93. SRQ-20 (Non-specific psychological distress, including suicidality: EC patients aged ≥ 18 years) - Van der Westhuizen,2016(149), 94. SRQ-20 (Non-specific psychological distress, including suicidality: EC male patients aged ≥ 18 years) - Van der Westhuizen,2016(149), 95. SRQ-20 (Non-specific psychological distress, including suicidality: EC female patients aged ≥ 18 years) - Van der Westhuizen,2016(149), 96. CES-D-10 - Zulu (MDD: Community dwellers aged 15-86 years) – Baron,2017(113), 97. CES-D-10 – Xhosa (MDD: Community dwellers aged 15-77 years) – Baron,2017(113), 98. CES-D-10 – Afrikaans (MDD: Community dwellers aged 15-84 years) – Baron,2017(113), 99. Whooley – no help Q (Depression, trauma / anxiety: 22 and 28 weeks pregnant women aged 18-42 years) – Marsay,2017(150), 100. Whooley – no help Q (Depression: 22 and 28 weeks pregnant women aged 18-42 years) – Marsay,2017(150), 101. Whooley- no Help Q (Trauma /Anxiety: 22 and 28 weeks pregnant women aged 18-42 years) – Marsay,2017(150), 102. Whooley – with help Q (Depression, trauma / anxiety: 22 and 28 weeks pregnant women aged 18-42 years) – Marsay,2017(150), 103. Whooley – with help Q (Depression: 22 and 28 weeks pregnant women aged 18-42 years) – Marsay,2017(150), 104. Whooley- with Help Q (Trauma /Anxiety: 22 and 28 weeks pregnant women aged 18-42 years) – Marsay,2017(150), 105. EPDS (Depression, trauma / anxiety: 22 and 28 weeks pregnant women aged 18-42 years) – Marsay,2017(150), 106. EPDS (Depression: 22 and 28 weeks pregnant women aged 18-42 years) – Marsay,2017(150), 107. EPDS (Trauma /Anxiety: 22 and 28 weeks pregnant women aged 18-42 years) – Marsay,2017(150), 108. EPDS - Anxiety sub-scale (Trauma / Anxiety: 22 and 28 weeks pregnant women aged 18-42 years) – Marsay,2017(150), 109. EPDS - Anxiety sub-scale (Trauma: 22 and 28 weeks pregnant women aged 18-42 years) – Marsay,2017(150), 110. EPDS - Anxiety sub-scale (Anxiety: 22 and 28 weeks pregnant women aged 18-42 years) – Marsay,2017(150), 111. CDQ (Substance use: Female caregivers aged 18-74 years) – Mellins,2017(129), 112. CDQ (PD: Female caregivers aged 18-74 years) – Mellins,2017(129), 113. AUDIT-C (Excessive alcohol use: Male patients aged ≥ 18 years receiving ART) – Morojele,2017(85), 114. AUDIT-C (Excessive alcohol use: Female patients aged ≥ 18 years receiving ART) – Morojele,2017(85), 115. AUDIT-3 (Excessive alcohol use: Male patients aged ≥ 18 years receiving ART) – Morojele,2017(85), 116. AUDIT-3 (Excessive alcohol use: Female patients aged ≥ 18 years receiving ART) – Morojele,2017(85), 117. QF (Excessive alcohol use: Male patients aged ≥ 18 years receiving ART) – Morojele,2017 (85), 118. QF(Excessive alcohol use: Female patients aged ≥ 18 years receiving ART) – Morojele,2017(85), 119. AUDIT-PC-5 (Excessive alcohol use: Male patients aged ≥ 18 years receiving ART) – Morojele,2017(85), 120. AUDIT-PC-5 (Excessive alcohol use: Female patients aged ≥ 18 years receiving ART) – Morojele,2017(85), 121. AUDIT-4 (Excessive alcohol use: Male patients aged ≥ 18 years receiving ART) – Morojele,2017(85), 122. AUDIT-4 (Excessive alcohol use: Female patients aged ≥ 18 years receiving ART) – Morojele,2017(85), 123. m-FAST- AUDIT-4 (Excessive alcohol use: Male patients aged ≥ 18 years receiving ART) – Morojele,2017(85), 124. m-FAST- AUDIT-4 (Excessive alcohol use: Female patients aged ≥ 18 years receiving ART) – Morojele,2017(85), 125. AVIDI-18^iii^ (MDD: Patients with low literacy) – Akena,2018(114), 126. BDI-I (MDD: HIV testing seekers aged) – Saal,2018(154), 127. EPDS (MDE: women aged 18-48 years) – van Heyningen,2018(86), 128. 3-item EPDS (MDE: women aged 18-48 years) – van Heyningen,2018(86), 129. K10 (MDE: women aged 18-48 years) – van Heyningen,2018(86), 130. K6 (MDE: women aged 18-48 years) – van Heyningen,2018(86), 131. PHQ-9 (MDE: women aged 18-48 years) – van Heyningen,2018(86), 132. PHQ-2 (MDE: women aged 18-48 years) – van Heyningen,2018(86), 133. Whooley (MDE: women aged 18-48 years) – van Heyningen,2018(86), 134. Whooley + help Q (MDE: women aged 18-48 years) – van Heyningen,2018(86), 135. EPDS - 3A (Anxiety disorders: women aged 18-48 years) – van Heyningen,2018(86), 136. K10 (Anxiety disorders: women aged 18-48 years) – van Heyningen,2018(86), 137. K6 (Anxiety disorders: women aged 18-48 years) – van Heyningen,2018(86), 138. GAD-2 (Anxiety disorders: women aged 18-48 years) – van Heyningen,2018(86), 139. EPDS (MDE & / Anxiety: women aged 18-48 years) – van Heyningen,2018(86), 140. 3-item EPDS (MDE & / Anxiety: women aged 18-48 years) – van Heyningen,2018(86), 141. K10 (MDE & / Anxiety: women aged 18-48 years) – van Heyningen,2018(86), 142. K6 (MDE & / Anxiety: women aged 18-48 years) – van Heyningen,2018(86), 143. PHQ-9 (MDE & / Anxiety: women aged 18-48 years) – van Heyningen,2018(86), 144. PHQ-2 (MDE & / Anxiety: women aged 18-48 years) – van Heyningen,2018(86), 145. Whooley (MDE & / Anxiety: women aged 18-48 years) – van Heyningen,2018(86), 146. Whooley + help Q (MDE & / Anxiety: women aged 18-48 years) – van Heyningen,2018(86), 147. The 3-item screening tool based on a 4-week recall (CMD: Pregnant women aged 15-38 years) – Abrahams,2019(87), 148. The 3-item screening tool based on a 2-week recall (CMD: Pregnant women aged 15-38 years) – Abrahams,2019(87), 149. AUD-C (Alcohol use disorder: Primary care patients aged ≥ 18 years) – Bhana,2019(88), 150. PHQ-2 (Depression: Primary care patients aged ≥ 18 years) – Bhana,2019(88), 151. GAD-2 (Anxiety: Primary care patients aged ≥ 18 years) – Bhana,2019(88), 152. y-IHDS (HIV-associated major neurocognitive disorders: Perinatally HIV-infected adolescents and HIV-negative adolescents aged 9-12 years) – Phillips,2019(89), 153. y-IHDS (HIV-associated minor neurocognitive disorders: Perinatally HIV-infected adolescents and HIV-negative adolescents aged 9-12 years) – Phillips,2019(89), 154. y-IHDS (CI: Perinatally HIV-infected adolescents and HIV-negative adolescents aged 9-12 years) – Phillips,2019(89), 155. Whooley01-GAD02-binary (Antenatal depression, Anxiety disorders or Maternal suicidal ideation: Pregnant women aged ≥ 18 years) – van Heyningen,2019(39), 156. Whooley01-Whooley02 - GAD02 – binary (Antenatal depression, Anxiety disorders or Maternal suicidal ideation: Pregnant women aged ≥ 18 years) – van Heyningen,2019(39), 157. Whooley01-Whooley02- EPDS10 –binary- (Antenatal depression, Anxiety disorders or Maternal suicidal ideation: Pregnant women aged ≥ 18 years) – van Heyningen,2019(39), 158. Whooley01- GAD02 -binary -EPDS10 – binary (Antenatal depression, Anxiety disorders or Maternal suicidal ideation: Pregnant women aged ≥ 18 years) – van Heyningen,2019(39), 159. Whooley01 - Whooley02 - GAD02 -binary, EPDS10-binary (Antenatal depression, Anxiety disorders or Maternal suicidal ideation: Pregnant women aged ≥ 18 years) – van Heyningen,2019(39), 160. EPDS items 1-10 (Antenatal depression, Anxiety disorders or Maternal suicidal ideation: Pregnant women aged ≥ 18 years) – van Heyningen,2019(39), 161. HSCL-25 (CMD: HIV testing seekers aged 18-71 years) – Bantjes, 2020(90), 162. AUDIT (AUD: Male HIV test-seekers aged 18 - 71 years) – Saal,2020(159), 163. AUDIT (AUD: Female HIV test-seekers aged 18 - 71 years) – Saal,2020(159), 164. SADS (MDD: HIV patients aged 18 to 74 years) – Andersen,2021(132), 165. PRS-SA Instrument (Distress: Patients ≥ 18 years with solid tumors) – Blanchard,2021(92), 166. PCL-5 (PTSD: Patients aged ≥ 18 years receiving ART) – Kagee, 2022(166), 167. CSBD-19 scale (CSBD: Adults aged 18-99 years) – Bothe,2023(170), 168. PHQ-9 (Depression: Adolescents aged 10-19 years in need of mental health support) – Marlow,2023(171), 169. GAD-7 (Anxiety: Adolescents aged 10-19 years in need of mental health support) – Marlow,2023(171), 170. K-10 (Current MDE: Pregnant women aged 15.5-43 years) – Spies,2009(143), 171. K-10 (Past MDE: Pregnant women aged 15.5-43 years) – Spies,2009(143), 172. K-10 (past bipolar disorder: Pregnant women aged 15.5-43 years) – Spies,2009(143), 173. K-10 (Past dysthymic disorder: Pregnant women aged 15.5-43 years) – Spies,2009(143), 174. K-10 (Current panic disorder: Pregnant women aged 15.5-43 years) – Spies,2009(143), 175. K-10 (Past panic disorder: Pregnant women aged 15.5-43 years) – Spies,2009(143), 176. K-10 (Social anxiety disorder: Pregnant women aged 15.5-43 years) – Spies,2009(143), 177. K-10 (Current PTSD: Pregnant women aged 15.5-43 years) – Spies,2009(143), 178. K-10 (Past PTSD: Pregnant women aged 15.5-43 years) – Spies,2009(143) |
| Sudan | 1 | EPDS (PND: Women aged 15-25 years at 3 months postpartum) – Khalifa,2015(35) |
| Tanzania | 11 | 1.AUDIT (AUD: Adults aged ≥ 18 years) – Vissoci,2023(137), 2. AUDIT-3 (AUD: Adults aged ≥ 18 years) – Vissoci,2023(137), 3.AUDIT-4 (AUD: Adults aged ≥ 18 years) – Vissoci,2023(137), 4. AUDIT-5 (AUD: Adults aged ≥ 18 years) – Vissoci,2023(137), 5. AUDIT-C (AUD: Adults aged ≥ 18 years) – Vissoci,2023(137), 6.AUDIT-PC (AUD: Adults aged ≥ 18 years) – Vissoci,2023(137), 7. HSCL-25 (Anxiety / depression: HIV-positive pregnant women aged ≥ 15 years attending antenatal clinics) – Kaaya,2002(54), 8. HSCL-15 (Anxiety / depression: HIV-positive pregnant women aged ≥ 15 years attending antenatal clinics) – Kaaya,2002(54), 9. HSCL-8 (Anxiety / depression: HIV-positive pregnant women aged ≥ 15 years attending antenatal clinics) – Kaaya,2002(54), 10. MINI (AUD: Alcohol users aged 18-24 years) – Francis,2015(145), 11. PHQ-9 (current MDE: Public clinics patients aged 18-63 years) – Smith-Fawzi, 2019(48) |
| Uganda | 23 | 1. SCQ^ii^ (ASD: Children aged 4-18 years) – Awadu,2021(102), 2. SRS-2 (ASD: Children aged 4-18 years) – Awadu,2021(102), 3. 23Q (ASD: Children aged 4-18 years) – Awadu,2021(102), 4. EPDS (PPD: Postpartum women aged 18-49 years) – Atuhaire,2023(31), 5.CDQ (MDD, Alcohol use, panic disorder, anxiety, drug abuse, PTSD, psychosis: PLWHIV in age range of ≥ 18 years) – Kwobah,2024(96), 6. CRAFFT (SUD: Primary school aged children aged 6-13 years) – Nalugya,2024(140), 7. CRAFFT (SUD: Boys aged 6-13 years) – Nalugya,2024(140), 8. CRAFFT (SUD: Girls aged 6-13 years) – Nalugya,2024 (140), 9. PDS (PTSD: war-affected adolescents and young adults aged 12-25 years) - Ertl,2011(124), 10. DHSCL (Depression: war-affected adolescents and young adults aged 12-25 years) – Ertl,2011(124) , 11. AVIDI (Depression: PLWHIV in age range of 18-71 years ) – Akena,2012(125), 12. AVIDI-4 (Depression: PLWHIV in age range of 18-71 years ) – Akena,2012(125), 13. SRQ-20 (Current depression: HIV+ individuals aged ≥ 18 years) - Nakimuli-Mpungu,2012(56), 14. SRQ-20 (Any depression: HIV+ individuals aged ≥ 18 years) - Nakimuli-Mpungu,2012(56), 15. K-10 (Depression/ anxiety: HIV patients aged ≥ 18 years) – Akena,2013(79), 16. K-2 (Depression/ anxiety: HIV patients aged ≥ 18 years) – Akena,2013(79), 17. PHQ-9 (Depression: HIV patients aged ≥ 18 years) – Akena,2013(79), 16. PHQ-2 (Depression: HIV patients aged ≥ 18 years) – Akena,2013(79), 18. CES-D (Depression: HIV patients aged ≥ 18 years) – Akena,2013(79), 19. PHQ-9 (Depression: Primary care and hospital patients aged 18-82 years) – Nakku,2016(128), 20. PHQ-2 (Depression: Primary care and hospital patients aged 18-82 years) – Nakku,2016(128), 21. AVIDI-18^iii^ (MDD: Patients aged ≥ 18 years with low literacy) – Akena,2018(114), 22. AVIDI-18 (MDD: Patients aged ≥ 18 years with low literacy) – Akena,2018(114), 23. 20-item depression scale (Depression: Adolescents aged 13-17 years living with HIV) – Ashaba,2019(107). |
| Zambia | 30 | 1. SRQ-20 (Depression / anxiety disorders: Primary health care clinic attendees aged 16 - 67 years) – Chipimo,2010(55), 2. SRQ-10 (Mental distress: Primary health care clinic attendees aged 16 - 67 years) – Chipimo,2010(55), 3. GHQ-12 (Mental distress: Primary health care clinic attendees aged 16 - 67 years) – Chipimo,2010(55), 4. CES-D (MDD: TB and ART patients aged ≥ 16 years) – Chishinga,2011(76), 5. AUDIT (AUD: TB and ART female patients aged ≥ 16 years) – Chishinga,2011(76), 6. AUDIT (AUD: TB and ART male patients aged ≥ 16 years) – Chishinga,2011(76), 7. PTSD-RI SS (Traumatic stress: Youth aged 6-15 years experienced sexual abuse) – Murray,2011(105), 8. PTSD-RI LSSS (Traumatic stress: Youth aged 6-15 years experienced sexual abuse) – Murray,2011(105), 9. PTSD-RI – TSS (Traumatic stress: Youth aged 6-15 years experienced sexual abuse) – Murray,2011(105), 10. PHST (Depression / anxiety: PWE in age range of 18-50 years) – Mbewe,2013(106), 11. ASSIST (Low vs moderate use of tobacco: OVC aged 13-17 years) – Kane,2016(82), 12. ASSIST (Moderate vs high use of tobacco: OVC aged 13-17 years) – Kane,2016(82), 13.ASSIST (Low vs moderate use of alcohol: OVC aged 13-17 years) – Kane,2016(82), 14. ASSIST (Moderate vs high use of alcohol: OVC aged 13-17 years) – Kane,2016(82), 15. ASSIST (Low vs moderate use of inhalants: OVC aged 13-17 years) – Kane,2016(82), 16. ASSIST (Moderate vs high use of inhalants: OVC aged 13-17 years) – Kane,2016(82), 17. ASSIST (Low vs moderate use of cannabis: OVC aged 13-17 years) – Kane,2016(82), 18. ASSIST (Moderate vs high use of cannabis: OVC aged 13-17 years) – Kane,2016(82), 19. ASSIST (Low vs moderate use of cocaine: OVC aged 13-17 years) – Kane,2016(82), 20. ASSIST (Moderate vs high use of cocaine: OVC aged 13-17 years) – Kane,2016(82), 21. ASSIST (Low vs moderate use of Amphetamines: OVC aged 13-17 years) – Kane,2016(82), 22. ASSIST (Moderate vs high use of Amphetamines: OVC aged 13-17 years) – Kane,2016(82), 23. ASSIST (Low vs moderate use of Sedatives: OVC aged 13-17 years) – Kane,(2016(82), 24. ASSIST (Moderate vs high use of Sedatives: OVC aged 13-17 years) – Kane,2016(82), 25. ASSIST (Low vs moderate use of Hallucinogens: OVC aged 13-17 years) – Kane,2016(82), 26. ASSIST (Moderate vs high use of Hallucinogens: OVC aged 13-17 years) – Kane,2016(82), 27. ASSIST (Low vs moderate use of other substances: OVC aged 13-17 years) – Kane,2016(82), 28. ASSIST (Moderate vs high use of other substances: OVC aged 13-17 years) – Kane,2016(82), 29. ASSIST (Low vs moderate use of TSI: OVC aged 13-17 years) – Kane,2016(82), 30. ASSIST (Moderate vs high use of TSI: OVC aged 13-17 years) – Kane,2016(82). |
| Zimbabwe | 28 | 1.ASSIST (TSI use vs abuse : in-patients and out-patients aged 18 - 45 years from PHC centers and drug treatment centers) – Humeniuk,2008(94), 2. ASSIST (TSI abuse vs dependence: in-patients and out-patients aged 18 - 45 years from PHC centers and drug treatment centers) – Humeniuk,2008(94), 3. ASSIST (alcohol use vs abuse: in-patients and out-patients aged 18 - 45 years from PHC centers and drug treatment centers) – Humeniuk,2008(94), 4. ASSIST (alcohol abuse vs dependence for alcohol: in-patients and out-patients aged 18 - 45 years from PHC centers and drug treatment centers) – Humeniuk,2008(94), 5.ASSIST (cannabis use vs abuse: in-patients and out-patients aged 18 - 45 years from PHC centers and drug treatment centers) – Humeniuk,2008(94), 6. ASSIST (cannabis abuse vs dependence: in-patients and out-patients aged 18 - 45 years from PHC centers and drug treatment centers) – Humeniuk,2008(94), 7.ASSIST (cocaine use vs abuse: in-patients and out-patients aged 18 - 45 years from PHC centers and drug treatment centers) – Humeniuk,2008(94), 8. ASSIST (cocaine abuse vs dependence: in-patients and out-patients aged 18 - 45 years from PHC centers and drug treatment centers) – Humeniuk,2008(94), 9. ASSIST (ATS use vs abuse: in-patients and out-patients aged 18 - 45 years from PHC centers and drug treatment centers) – Humeniuk,2008(94), 10. ASSIST (ATS abuse vs dependence: in-patients and out-patients aged 18 - 45 years from PHC centers and drug treatment centers) – Humeniuk,2008(94), 11.ASSIST (sedatives use vs abuse: in-patients and out-patients aged 18 - 45 years from PHC centers and drug treatment centers) – Humeniuk,2008(94), 12. ASSIST (sedatives abuse vs dependence: in-patients and out-patients aged 18 - 45 years from PHC centers and drug treatment centers) – Humeniuk,2008(94), 13. ASSIST (opioids use vs abuse: in-patients and out-patients aged 18 - 45 years from PHC centers and drug treatment centers) – Humeniuk,2008(94), 14. ASSIST (opioids abuse vs dependence: in-patients and out-patients aged 18 - 45 years from PHC centers and drug treatment centers) – Humeniuk,2008(94), 15. ASSIST-SSI (self-reported use of cocaine vs its presence in hair: in-patients and out-patients aged 18 - 45 years from PHC centers and drug treatment centers) – Humeniuk,2008(94), 16. ASSIST-SSI (self-reported use of ATS vs its presence in hair: in-patients and out-patients aged 18 - 45 years from PHC centers and drug treatment centers) – Humeniuk,2008(94), 17. ASSIST-SSI (self-reported use of Benzodiazepines vs its presence in hair: in-patients and out-patients aged 18 - 45 years from PHC centers and drug treatment centers) – Humeniuk,2008(94), 18. ASSIST-SSI (self-reported use of opioids vs its presence in hair: in-patients and out-patients aged 18 - 45 years from PHC centers and drug treatment centers) – Humeniuk,2008(94), 19. EPDS (MDD: Postpartum HIV-infected and uninfected women aged ≥ 18 years) – Chibanda,2010(34), 20. SSQ (Depression: Adolescents aged 15-19 years) – Haney,2014(126), 21. SSQ-14 (CMD: Adults aged ≥ 18 years attending the clinic) – Chibanda,2016(46), 22. SSQ-14 (Depression: Adults aged ≥ 18 years attending the clinic) – Chibanda,2016(46), 23. PHQ-9 (Depression: Adults aged ≥ 18 years attending the clinic) – Chibanda,2016(46), 24. PHQ-2 (Depression: Adults aged ≥ 18 years attending the clinic) – Chibanda,2016(46), 25. GAD-7 (Anxiety: Adults aged ≥ 18 years attending the clinic) – Chibanda,2016(46), 26.PCL-5 (PTSD: Clinical attendants aged ≥ 18 years) – Verhey,2018(116), 27. PCL-5 (PTSD: PLWHIV aged ≥ 18 years) – Verhey,2018(116), 28. IES-R (PTSD: Primary care center patients aged ≥ 18 years) – Abas,2023(118) |
| Total | 450 |  |

**Abbreviations:** ASSIST: The alcohol, smoking and substance involvement screening test, TSI: Total substance involvement , SSI: Specific Substance Involvement , AUDIT-C = consumption items (Items 1-3) from the AUDIT , AUDIT-3= one AUDIT item (Item 3), assessing binge drinking only , PSQ: Psychosis Screening Questionnaire, EPDS: Edinburgh postnatal depression scale ,PHQ-9: Patient Health Questionnaire-9, SCQ: Social Communication Questionnaire, 23Q: 23 Questions screener, SRS-2: Social responsiveness scale, Second Edition , Proxy mwTool-3: The Proxy Mental Wellness Tool-a three time screener, ECAS: Ethiopian Cognitive Assessment Battery for Schizophrenia , WLLT: World List Learning Test, DST: Digit Sequencing Task - verbal working memory, CBTT: Visual working memory , ANT: Animal Naming Test-Verbal learning, DSST: Digit Symbol Substitution Test, TMT Part A & SP: Trail Making Test for Attention part and speed of processing , TMT B (EF): Trail Making Test for Executive Function, CDQ: Client Diagnostic Questionnaire , MDQ: Mood Disorder Questionnaire , R-MDQ: Rwandese version of the Mood Disorder Questionnaire , CRAFFT: Car, Relax, Alone, Forget, Family/Friends, Trouble tool, mwTool-13: Mental Wellness Tool - thirteen items, CDM: Common mental disorder, MDE: Major Depressive Episode, GAD: General Anxiety Disorder, PTSD: Post Traumatic Stress Disorder, AUD: Alcohol Use Disorder, SUD: Substance Use Disorder, SMD: Severe Mental Disorder, PD: Psychotic Disorder, M/HE: Manic or Hypomanic Episode, SR: Suicidality Risk, GAD-7: Generalized Anxiety Disorder-7, PC-PTSD-5: Primary Care Post Traumatic Stress Disorder Screen for DSM-5, GAD-7: Generalized Anxiety Disorder-7 , PC-PTSD-5: Primary Care Post Traumatic Stress Disorder Screen for DSM-5, C-SSRS: Columbia Suicide Severity Rating Scale, PHQ-2: a two-item Patient Health Questionnaire , PHQ-2/9: Patient Health Questionnaire with two steps, starting with a 2-item PHQ and, if depression suspected, continue to the remaining 7 items of the PHQ , PHQ-4: a four-item Patient Health Questionnaire, PHQ-Q9: a one-item (question 9 only) Patient Health Questionnaire , SRi: Suicide Risk, GAD-2/7: Generalized Anxiety Disorder screening tool starting with two questions and, if anxiety suspected, continue with remaining 5 items of the GAD screening tool, C-SSRS: Columbia Suicide Severity Rating Scale , HSCL-25: Hopkins Symptom Checklist-25 , Minor DD: Minor Depression Disorder, BDI: Beck Depression Inventory , K10: Kessler Psychological Distress Scale-10 , K6: Kessler Psychological Distress Scale-6, SRQ: Self-reporting Questionnaire , CPDS: Child Psychosocial Distress Screener , CES-D: Center for Epidemiological Studies Depression Scale , HTQ: Harvard Trauma Questionnaire , ADD: Any depressive disorders, cMajor DD: Current Major Depressive Disorder, cMinor and cMajor DD: Current Minor and Major Depressive Disorder , PDS: Posttraumatic Diagnostic Scale, DHSCL: Depression section of the Hopkins Symptom Checklist , PSC: Pediatric Symptom Checklist, PSC-Y: Pediatric Symptom Checklist – youth self-report version , PSC-17-Y: a 17-item Pediatric Symptom Checklist – youth self-report version , PTSD-RI: Adapted trauma-focused mental health assessment tool - the UCLA Post-traumatic Stress Disorder - Reaction Index , PTSD-RI SS: PTSD-RI Symptom scale , PTSD-RI LSSS: PTSD-RI - Locally-specific symptom scale: , PTSD-RI – TSS: PTSD-RI - Total symptom scale, AVIDI: Akena visual depression inventory, CES-DC: Center for Epidemiological Studies Depression Scale for Children , CBQ: Child Behavior Questionnaire , RQC: Reporting Questionnaire for Children K-2: Shorter version of the Kessler-10 , CIDI: Structured Composite International Diagnostic Interview , PHST – Depression and Anxiety: Primary Healthcare Screening Tool to Identify Depression and Anxiety Disorders, EPDS-10: 10-item Edinburgh Postnatal Depression Scale , EPDS-7: 7-item traditional depression subscale of Edinburgh Postnatal Depression Scale , EPDS-5R: 5-item Edinburgh Postnatal Depression Scale, EPDS-3R: 3-item Edinburgh Postnatal Depression Scale, RFA Item 11: an 11-item risk factor assessment (RFA), PRI-3items (EPDS8, RFA1&11): Psychiatric referral items (3 items - EPDS8, RFA1&11- combined), SSQ: Shona Symptom Questionnaire , BDI-II: Beck Depression Inventory-II , CDI-II-S: Children Depression Inventory-II-Short, YCPS-R: Youth Conduct Problems Scale-Rwanda , YCPS-R SF: Youth Conduct Problems Scale-Rwanda Short Form, EPDS-2: a 2-item EPDS with two items analogue of the Patient Health Questionnaire, EPDS-3: a 3-item anxiety subscale of the EPDS, EPDS-5: a 5-item version of the depressive symptoms subscale of the EPDS, EPDS-7: a 7-item depressive symptoms subscale of the EPDS, DSRS: Depression Self-Rating Scale, CPSS: Child PSTD Symptom Scale, SCARED-41: a 41-item Screen for Child Anxiety Related Emotional Disorders, SAST: Sexual Addiction Screening Test , W-SAST: Women Sexual Addiction Screening Test, SRQ-SIB: Self-Report Questionnaire, Suicide Ideation and Behavior , MINI: Mini International Neuropsychiatric Interview questionnaire, CDI: Children Depression Inventory , PGQ: Prolonged Grief Questionnaire , DTI: Developmental Trauma Inventory , DTI-PTSD: Developmental Trauma Inventory for PTSD (DTI-PTSD), DTI-Complex PTSD: Developmental Trauma Inventory for Complex PTSD , SBQ-R-4: a 4-item Suicidal Behaviors Questionnaire-Revised (SBQ-R) CES-D-10: a 10-item Centre for Epidemiological Studies Depression Scale , Whooley – no help Q: Whooley excluding help question , Whooley – with help Q: Whooley including help question , CDQ: Client Diagnostic Questionnaire , AUDIT-QF = quantity and frequency items (Items 1 and 2) from the AUDIT , AUDIT-PC-5: AUDIT Primary Care using five AUDIT items (Items 1, 2, 4, 5, and 10) , AUDIT-4 = four AUDIT items (Items 1, 2, 3, and 10) , m-FAST- AUDIT-4 = modified Fast Alcohol Screening Test - four AUDIT items (Items 3, 5, 8, and 10) , PANSI: Positive and Negative Suicide Ideation Inventory, PANSI - NSI - PANSI Negative Suicide Ideation subscale , BHS: Beck Hopelessness Scale , HSCL: Hopkins Symptoms Checklist-15, PDEPS: Perinatal Depression Screening, LSEQ: Leeds Sleep Evaluation Questionnaire, LSEQ-M: The Adapted Leeds Sleep Evaluation Questionnaire Mizan, DBD-TRS: Disruptive behaviour disorders teacher rating scale , DBD - ADHD: Disruptive Behaviour Disorders - attention-deficit/hyperactivity disorder (DBD-ADHD), DBD - CD: Disruptive Behaviour Disorders – Conduct disorders , DBD-ODD: Disruptive behaviour disorders -Oppositional defiant disorder, BDI-I: Beck Depression Inventory-I, SHI: Sleep Hygiene Index , PCL-5: PTSD Checklist for DSM-5 , PLWHIV: People Living with HIV, WERCAP Screen: The Washington Early Recognition Center Affectivity and Psychosis Screen, aWERCAP screen: Affectivity (aWERCAP) Screen, pWERCAP screen: Psychosis (pWERCAP) Screen, IHDS: International HIV Dementia Scale, y-IHDS - major ND: Youth-International HIV Dementia Scale for major neurocognitive disorder, y-IHDS - minor ND: Youth-International HIV Dementia Scale for major neurocognitive disorder , y-IHDS – CI: Youth-International HIV Dementia Scale for major cognitive impairment , Whooley Q: Whooley questions, BOSHAS: Body Shape Scale , CBS: Current Body Shape, DBS: Desired Body Shape, IBS: Ideal Body Shape, PHQ-9-MZ: Patient Health Questionnaire-9 Mozambique , PHQ-2-MZ: Patient Health Questionnaire-2 Mozambique , HDRS: Hamilton Depression Rating Scale , SADS: South African Depression Scale, AUDIT-10-MZ: Alcohol Use Disorders Identification Test 10-Item –Mozambique, AUDIT-C-MZ: Alcohol Use Disorders Identification Test-Concise – Mozambique, CDST: Child Depression Screening Tool , PRS-SA: Patient-Reported Symptoms-South Africa Instrument, MiSQuaSHI: Mizan Sleep Quality and Sleep Hygiene Index , SQQ: Sleep Quality Questionnaire , CAAD-PC-MZ: A Combined Assessment for Anxiety and Depression in Primary Care in Mozambique , DBDRS-ODD: Disruptive Behavior Disorders Rating Scale -Oppositional Defiant Disorder Subscale , DBDRS-CD: Disruptive Behavior Disorders Rating Scale -Conduct Disorder Subscale, SDQ: Strengths and Difficulties Questionnaire , PHQ-A: Patient Health Questionnaire – Adolescents, pWERCAP Screen (3MO): Washington Early Recognition Center Affectivity and Psychosis (WERCAP) Screen within 3 months (3MO), pWERCAP Screen (12MO): Washington Early Recognition Center Affectivity and Psychosis (WERCAP) Screen within 12 months (12MO), DASS- D: Depression Anxiety Stress Scale - Depression Subscale (DASS- D), DASS- A: Depression Anxiety Stress Scale - Anxiety Subscale (DASS- A), IES-R: Impact of Events Scale-Revised , PHQ-2 (PLWIH) Depression: 2-item Patient Health Questionnaire for depression – People Living with HIV , GAD-2 (PLWIH) Anxiety: 2-item Patient Health Questionnaire for anxiety – People Living with HIV , PHQ-4 (PLWIH) Anxiety: 4-item Patient Health Questionnaire for anxiety – People Living with HIV, PHQ-4 (PLWIH) Depression: 4-item Patient Health Questionnaire for Depression – People Living with HIV, PHQ-2 (HIV- People) Depression: 2-item Patient Health Questionnaire for depression – HIV negative People, PHQ-2 (HIV- People) Anxiety: 2-item Patient Health Questionnaire for Anxiety – HIV negative People, PHQ-4 (HIV- People) Anxiety: 4-item Patient Health Questionnaire for anxiety – HIV negative People, PHQ-4 (HIV- People) Depression: 4-item Patient Health Questionnaire for depression – HIV negative People, CSBD-19 scale: Compulsive sexual behavior disorder-19 scale HAMD-17: Hamilton Depression Rating Scale 17-item, ART: Anti-retroviral therapy, PHC: Primary health care , Kenyan adults with positive screens on the WHO mental health treatment GAP- Intervention Guidelines mhGAP-IG: Mental health treatment GAP- Intervention Guidelines, PLWHIV: People living with HIV, WHO: World Health Organization, AUD: Autism spectrum disorders, SD: Standard Deviation, SRD: Substance-related disorders, BHS: Beck Hopelessness Scale , MD: Mental Disorder, PwE: Patients with epilepsy, PTF: Primary and tertiary facilities, PD: Psychotic disorder, M/HE: Manic or Hypomanic Episode, HT: Hypertension, EC: Emergency center, CSBD: Compulsive Sexual Behavior Disorder, PND: Postnatal depression , PPD: Postpartum depression , ATS : Amphetamine -type stimulants , VWM: Verbal Working Memory.

**Key notes:** ^i^ Validated in a two-country study at Mozambique and South Africa, ^ii^ Validated in a two-country study at Kenya and Uganda, ^iii^ Validated in a two-country study at South Africa and Uganda, ^iv^ Validated in a two-country study at Cameroon and Senegal , ^v^ Validated in a two-country study at Cameroon and Senegal , ^vi^ Validated in a two-country study at Cameroon and Senegal, ^vii^ Validated in a two-country study at Cameroon and Senegal, ^viii^ Validated in a two-country study at Cameroon and Senegal, ^ix^ Validated in a two-country study at Cameroon and Senegal, ^x^ Validated in a two-country study at Cameroon and Senegal, ^xi^ Validated in a two-country study at Cameroon and Senegal, ^xii^ Validated in a two-country study at Cameroon and Senegal.
