# Supplementary material for: A systematic review and meta-analysis of diagnostic test accuracy of mental health screening tools applicable to adolescents in sub-Saharan Africa
Source: Front Psychiatry. 2026 Jun 16;17:1728252. doi: 10.3389/fpsyt.2026.1728252 (PMC13317013; doi:10.3389/fpsyt.2026.1728252)
Supplement: Supplementary file 4 [file Table4.pdf]

frontiers

Supplementary appendix 4: Studies and validated screening tools eligible for meta-analyses

Reference # in the paper	Lead author	Country	Name of the tool(s) (abbreviation(s))	Condition(s)	Target population	Final sample size	Gender	Final age range	Gold standard	Language of the tool	Items number in validated tool	Internal consistency	Cut-off point	Sensitivity(se)	Specificity (sp)	AU
1	Miafo 2024 (40)	Cameroon	EPDS	Perinatal depression	Adolescent mothers	1633	Females only	12-20 years	Diagnostic and Statistical Manual of Mental Disorders, Fifth Edition (DSM-V) diagnosis of depression	French	10	NR	11	0.93 (0.91-0.94)	0.53 (0.51-0.56)	0.85
2	Atuhaire 2023 (47)	Uganda	EPDS	Postpartum depression (PPD)	Postpartum women	278	Females only	18-49 years	The Mini-International Neuropsychiatric Interview (MINI 7.0.2)	Runyankore-Rukiga	10	NR	10	0.87 (0.77-0.94)	0.92(0.88-0.95)	NR
3	Stockton 2024 (59)	South Africa	PHQ-2	Depression	Patients or accompanies at health facilities	1885	Both females	18-88 years	The MINI diagnostic questionnaire	isiXhosa or English	2	NR	>0	0.94 (0.93-0.96)	0.43 (0.41 - 0.46)	0.80 (0.78-0.82)
			PHQ-9 (cut off of 5)								9	0.89	5	0.84 (0.80-0.88)	0.67 (0.65-0.70)	NR
			PHQ-9 (cut off of 10)								9	NR	10	0.49 (0.44-0.54)	0.90 (0.89-0.92)	0.83 (0.81-0.86)
			PHQ-2/9 (cut off of 5)								9	NR	5	0.84 (0.80-0.87)	0.70 (0.67-0.72)	NR
			PHQ-2/9 (cut off of 10)								9	NR	5	0.49 (0.44-0.54)	0.91 (0.89-0.92)	0.84 (0.82-0.86)
4	Uwakwe 2003 (57)	Nigeria	EPDS	Depression	Postpartum women	225	Females only	18-39 years	The modified ICD-10 Symptom Check list (SCL)	1. English 2. Igbo (local language)	10	0.83	9	0.75	0.97	NR

Reference # in the paper	Lead author	Country	Name of the tool(s) (abbreviation(s))	Condition(s)	Target population	Final sample size	Gender	Final age range	Gold standard	Language of the tool	Items number in validated tool	Internal consistency	Cut-off point	Sensitivity(se)	Specificity (sp)	AU
5	Adewuya 2006i(65)	Nigeria	PHQ-9	Minor depressive disorder	University students	512	Both females and males	15 to 40 years	The MINI diagnostic questionnaire	English	9	0.85	5	0.90	0.99	0.99 (0.98-1.00)
	Adewuya 2006i(65)		PHQ-9	Major depressive disorder							9	0.85	10	0.85	0.99	0.995 (0.991 -1.00)
6	Adewuya 2006ii(56)	Nigeria	EPDS - Major and minor depression	Major and minor depression	Women in late pregnancy (32 weeks and above)	86	Females only	24.89 (SD= 6.41)	The MINI diagnostic questionnaire	1. English 2. Yoruba language	10	0.85	10	0.87	0.92	0.97 (0.93-1.00)
	Adewuya 2006ii(56)		EPDS - Major depression only	Major depression only				24.89 (SD= 6.41)			10	0.85	12	1	0.96	0.995 (0.984 - 1.00)
7	Baggaley 2007(46)	Burkina Faso	K10	Postnatal depression	Postpartum women	61	Females only	17-46 years	Diagnostic interview with a local psychiatrist	1. West African French 2. Moore 3. Dioula	10	0.87	14	0.59	0.91	0.77
8	Spies 2009(77)	South Africa	K-10 - Current MDE	Current major depressive disorder	pregnant women	129	Females only	15.5 - 43.0 years	The Structured Clinical Interview DSM-IV (SCID)	English or Afrikaans	10	NR	21.5	0.73	0.54	0.66
			K-10 - Past MDE	Past major depressive disorder							10	NR	23.5	0.53	0.63	0.58
			K-10 -Past dysthymic disorder	Past dysthymic disorder							10	NR	33.5	1	0.93	0.93
9	Weobong 2008(58)	Ghana	EPDS	Depression	Women within postnatal period 5 and 11 weeks	160	Females only	15-46 years	The semi-structured Comprehensive Psychopathological Rating Scale (CPRS)	Twi	10	0.79	10/11	0.78	0.73	0.84 (0.76-0.92)
			PHQ-9	Depression							9	0.79	4/5	0.94	0.75	0.90 (0.81-0.98)

Reference # in the paper	Lead author	Country	Name of the tool(s) (abreviation(s))	Condition(s)	Target population	Final sample size	Gender	Final age range	Gold standard	Language of the tool	Items number in validated tool	Internal consistency	Cut-off point	Sensitivity(se)	Specificity (sp)	AU
10	Chibanda 2009(48)	Zimbabwe	EPDS	Major depression	Postpartum HIV-infected and uninfected women	210	Females only	18 years and older	A structured clinical interview based on DSM IV (4th edition) criteria for major depression	Shona	10	0.87	12	0.88	0.87	0.82
11	Spies 2010(78)	South Africa	K-10 (Current MDE)	Current MDE	HIV-infected adults	429	Both females and males	16-65 years	The MINI diagnostic questionnaire	1.Xhosa 2.Africaans 3. English	10	0.87	≤ 28	0.67	0.77	0.77
			K-10	Past MDE							10	0.87	≤28	0.77	0.75	0.75
12	Akena 2013(66)	Uganda	K-10	Depression	HIV patients	368	Both females and males	18 years and older	The MINI diagnostic questionnaire	NR	10	NR	23	0.83	0.72	0.82 (0.72-0.93)
			PHQ-9							NR	9	NR	10	0.92	0.81	0.96 (0.92-0.99)
13	Gelaye 2013(61)	Ethiopia	PHQ-9	Major Depressive Disorder (MDD)	Adult outpatients	926	Both females and males	18-69 years	Schedules for Clinical Assessment in Neuropsychiatry (SCAN)	Amharic	9	0.81	10	0.86 (0.78-0.92)	0.67 (0.61-0.73)	0.77 (0.68-0.85)
14	Rochat 2013(55)	South Africa	EPDS	Antenatal depression	Pregnant women in their second trimester who live in the catchment area	109	Females only	16-40 years	The Structured Clinical Interview for Depression (SCID)	Zulu	10	0.613	≥ 13	0.69	0.78	0.82(0 .73-0.89)
15	Bhana 2015(60)	South Africa	PHQ-2	Major depressive disorder	Chronic care patients (e.g., HIV, hypertension , diabetes)	676	Both females and males	18-88 years	The Structured Clinical Interview for DSM-IV (SCID) by clinical psychologists	seTswana or English	2	NA	2	0.6	0.84	0.85 (0.82-0.88)
			PHQ-9								9	0.76	9	0.49	0.94	0.76 (0.73-0.79)
16	Hanlon 2015 (62)	Ethiopia	K10	Major Depressive	Patients attending	306	Both females	18 years	The MINI diagnostic questionnaire	Amharic	10	0.88	18	0.79	0.77	0.83 (0.74-0.92)

Reference # in the paper	Lead author	Country	Name of the tool(s) (abreviation(s))	Condition(s)	Target population	Final sample size	Gender	Final age range	Gold standard	Language of the tool	Items number in validated tool	Internal consistency	Cut-off point	Sensitivity(se)	Specificity (sp)	AU
			PHQ-9	Disorder (MDD) Major	the PHC facilities			and above			9	0.84	5	0.83	0.75	0.85 (0.77-0.95)
			PHQ-2								2	NR	1	0.83	0.61	0.78 (0.66-0.90)
17	Khalifa 2015(146)	Sudan	EPDS	Postnatal depression (PND)	Women at 3 months postpartum	238	Females only	15-25 years	The MINI diagnostic questionnaire	Arabic	10	0.83	12	0.89	0.82	0.89 (0.779 - 0.999)
18	Chibanda 2016(68)		PHQ-9	Depression	Adults attending the clinic	264	Both females and males	18 years or older	Psychiatrists using the Structured Clinical Interview of the DSM-IV (SCID)	Shona	9	0.86	11	0.85 (0.78-0.90)	0.69 (0.59-0.77)	0.84 (0.79-0.88)
			PHQ-2								2	NR	2	0.91 (0.86-0.95)	0.40 (0.31-0.5)	NR
19	Gelaye 2016(76)	Ethiopia	PHQ-2	Major Depressive Disorder (MDD)	Adult outpatients	363	Both females and males	18-69 years	Clinical Assessment in Neuropsychiatry (SCAN)	Amharic	2	NR	3	0.74(0.59-0.86)	0.60 (0.54-0.65)	0.72 (0.64-0.79)
20	Nakku 2016(67)	Uganda	PHQ-9	Depression	Primary care and hospital patients	153	Both females and males	18-82 years	The MINI diagnostic questionnaire	Luganda	9	0.68	5	0.67	0.78	0.74 (0.60-0.89)
			PHQ-2								2	NR	1	0.66	0.59	0.68 (0.54-0.82)
21	Chorwe-Sungani 2018(50)	Malawi	EPDS	Depression	Pregnant women	97	Females only	18 years and above	The Mini International Neuropsychiatric Interview (MINI)	Chichewa	10	0.8	10	0.68 (0.47–0.85)	0.88 (0.78-0.94)	0.85 (0.763 - 0.915)
22	Green 2018(52)	Kenya	EPDS - Original (two weeks)	Major Depressive Episode (MDE)	Pregnant women and new mothers	193	Females only	18 years old and above	The Structured Clinical Interview for DSM-5, Research Version to diagnose cases of depression (SCID-5-RV)	Kiswahili	10	0.78	16	0.7	0.72	0.8
			EPDS: Revised (one week)							Kiswahili	10	0.83	13	0.6	0.73	0.8
			PHQ-9							Kiswahili	9	0.81	15	0.7	0.74	O.79

Reference # in the paper	Lead author	Country	Name of the tool(s) (abreviation(s))	Condition(s)	Target population	Final sample size	Gender	Final age range	Gold standard	Language of the tool	Items number in validated tool	Internal consistency	Cut-off point	Sensitivity(se)	Specificity (sp)	AU
23	Woldetensay 2018 (63)	Ethiopia	PHQ-9	Depression	Pregnant women	216	Females only	18 to 40 years	The MINI-Plus scale	Afaan Oromo	9	0.84	8	0.81	0.79	0.88 (0.81-0.95)
24	Bhana 2019(75)	South Africa	PHQ-2	Depression	Primary care patients	1214	Both females and males	18 years and above	Adult Primary Care (APC) guidelines	isiZulu or English	2	0.71	3	0.58	0.77	0.72 (0.65-0.78)
25	Smith Fawzi 2019(69)	Tanzania	PHQ-9	Current MDE	Public clinics patients	174	Both females and males	18-63 years	The MINI diagnostic questionnaire	Swahili	9	0.83	9	0.78 (0.52-0.94)	0.87 (0.80-0.92)	0.87 (0.77-0.96)
26	Heyningen 2019(54)	South Africa	EPDS	MDE	Pregnant women	376	Females only	18 years or older	The MINI diagnostic questionnaire	1. Afrikaans 2. isiXhosa	10	NR	13	0.75	0.78	0.83 (0.78-0.88)
27	Cumbe 2020(70)	Mozambique	PHQ-2-MZ	Major depressive disorder (MDD)	Primary care patients	502	Both females and males	18 years old or over	The MINI 5.0-Mozambique (MINI 5.0-MZ)	Mozambican Portuguese	2	0.61	2	0.74	0.72	0.78 (0.70-0.85)
			PHQ-9-MZ								9	0.84	9	0.47	0.94	0.81 (0.73-0.89)
28	Degefa 2020(64)	Ethiopia	PHQ-9	MDE	Cancer outpatients	163	Both females and males	18 years and above	The MINI diagnostic questionnaire	Amharic	9	0.78	4	0.88	0.78	0.93 (0.88-0.97)
29	Molebatsi 2020(71)	Botswana	PHQ-9	Depression	Adult primary care attendants	257	Both females and males	18 - 79 years	The MINI depression module	Setswana or English	9	0.799	9	0.72(0.63-0.81)	0.76 (0.69–0.83)	0.81 (0.76-0.85)
30	Sebera 2020(72)	Rwanda	PHQ-9	Mild depression	Patients with epilepsy (PwE)	434	Both females and males	15 years and older 15	The Hamilton Depression Rating Scale (HDRS)	Kinyarwanda	9	0.87	5	0.72	0.7	0.80 (0.74-0.86)
			PHQ-9	Moderate depression							9	0.87	5	0.89	0.59	0.84 (0.80-0.90)

Reference # in the paper	Lead author	Country	Name of the tool(s) (abbreviation(s))	Condition(s)	Target population	Final sample size	Gender	Final age range	Gold standard	Language of the tool	Items number in validated tool	Internal consistency	Cut-off point	Sensitivity(se)	Specificity (sp)	AU
			PHQ-9	Severe depression							9	0.87	7	0.94	0.65	0.87 (0.83-0.93)
31	Pence 2012(73)	Cameroon	PHQ-9	MDD	HIV-positive patients on anti-retroviral therapy (ART)	400	Both females and males	18-55 Years	Composite International Diagnostic Instrument (CIDI)	English	9	NR	10	0.27 (0.06-0.61)	0.94 (0.91-0.96)	NR
32	Lovero 2022(39)	Mozambique	PHQ-A	Depression	Adolescents	485	Both females and males	12-19 years	The MINI for Children and Adolescents (MINI-KID)	Portuguese	9	0.80	8	0.78	0.80	0.85 (0.76-0.90)
33	Moya 2022(51)	Malawi	EPDS	Depression	Postpartum women	115	Females only	24 ± 6.8 years	Structured Clinical Interview for DSM-IV (SCID)	Chichewa	10	0.74	10	0.77	0.67	0.75 (0.60-0.89)
34	Marlow 2022(36)	South Africa	PHQ-9	Depression	Adolescents in need of mental health support	302	Both females and males	10 - 19 years	Kiddie Schedule for Affective Disorders and Schizophrenia (K-SADS)	isiXhosa	9	NR	10	0.91	0.76	0.75 (0.60-0.89)
35	Mutiso 2022(53)	Kenya	EPDS	Postnatal depression	Women in the first year post delivery	544	Females only	18 - 44 years	The DSM-IV criteria Mini-International Neuropsychiatric Interview for adults (MINI-Plus)	Kamba	NR	0.85	11	0.81 (0.71-0.89)	0.83(0.79-0.86)	0.87 (0.84-0.89)
36	Tele 2023(37)		PHQ-9	MDE	Adolescents	250	Both females and males	10 - 19 years	Clinical interview using Kiddie Schedule of Affective Disorders and Schizophrenia	English	9	0.86	9	0.95	0.73	0.89 (0.84-0.92)
			PHQ-9							2. Swahili	9	0.83	9	0.89	0.7	0.87 (0.82-0.90)

Abbreviations / Acronyms: EPDS: Edinburgh postnatal depression scale, PHQ-2: a two-item Patient Health Questionnaire, PHQ-9: a nine-item Patient Health Questionnaire, PHQ-2/9: a two-step nine-item Patient Health Questionnaire, PHQ-A: PHQ-9 for adolescents, PHQ-9-MZ: Patient Health Questionnaire-9 Mozambique, PHQ-2-MZ: Patient Health Questionnaire-2 Mozambique, NR: Not Reported and MINI: Mini-International Neuropsychiatric Interview (MINI).
