# Supplementary material for: A systematic review and meta-analysis of diagnostic test accuracy of mental health screening tools applicable to adolescents in sub-Saharan Africa
Source: Front Psychiatry. 2026 Jun 16;17:1728252. doi: 10.3389/fpsyt.2026.1728252 (PMC13317013; doi:10.3389/fpsyt.2026.1728252)
Supplement: Supplementary file 5 [file Table5.pdf]

Supplementary appendix 5: Outputs of HSROC sub-group meta-analysis models

1. Tables for sub-group analysis for EPDS

Table 1.1: Outputs of the HSROC meta-analysis model for EPDS considering prenatal depression studies only

Study author, country	Sample size (N)	Cut off	Se (95% CI)	Sp (95% CI)	TP	FN	FP	TN	Key outputs of HSROC model[1]
Miafo 2024, Cameroon	1633	11	0.93 (0.91-0.94)	0.53 (0.51-0.56)	920	74	299	340	1. Pooled se (95%CI): 0.74 (0.59-0.86)<br>2. Pooled FPR (95%CI): 0.18 (0.10-0.31)<br>3. Pooled sp(95%CI)*: 0.82 (0.69- 0.90)<br>4. AUC: 0.85<br>5.Partial AUC (restricted to FPR): 0.77<br>6. Between study std.dev for se: 0.82<br>7. Between study std.dev for FPR: 0.80<br>8. rho between se and sp: 0.1<br>9. I^2 estimate by Holling sample size unadjusted approaches: 27.2- 76.6 %
Adewuya 2006a, Nigeria	86	10	0.87	0.92	13	2	6	65	
Adewuya 2006b, Nigeria	86	12	1	0.96	9	0	3	74	
Rochat 2013, South Africa	109	≥ 13	0.69	0.78	35	16	13	45	
Chorwe-Sungani 2018, Malawi	97	10	0.68 (0.47–0.85)	0.88 (0.78–0.94)	17	8	9	63	
vanHeyningen 2019, South Africa	376	13	0.75	0.78	113	38	50	176	

[1]Primary studies are very few, *Deduced from FPR: 1-FPR

Table 1.2: Outputs from the HSROC Meta-analysis model for EPDS considering postpartum depression studies only

Study author, country	Sample size (N)	Cut off	Se (95%CI)	Sp (95% CI)	TP	FN	FP	TN	Key outputs of HSROC model
Atuhaire 2023, Uganda	278	10	0.87(0.77–0.94)	0.92(0.88–0.95)	66	10	16	186	1.Pooled se (95%CI): 0.83 (0.78- 0.87)<br>2.Pooled FPR (95%CI): 0.15 (0.08- 0.25)<br>3.Pooled sp(95%CI)*: 0.85 (0.75-0.92)<br>4.AUC: 0.83<br>5.Partial AUC (restricted to FPR):0.82<br>6.Between study std.dev for se: 0.03<br>7.Between study std.dev for FPR: 0.84<br>8.rho between se and sp: -1.00<br>9. I^2 estimates by Holling sample size unadjusted appr oaches: 28.3 - 36.9 %
Uwakwe 2003, Nigeria	225	9	0.75	0.97	18	6	6	195	
Weobong 2008, Ghana	160	10/11	0.78	0.73	14	4	38	104	
Chibanda 2010, Zimbabwe	210	12	0.88	0.87	56	8	19	127	
Khalifa 2015, Sudan	238	12	0.89	0.82	19	2	39	177	
Moya 2022, Malawi	115	10	0.77	0.67	10	3	34	68	
Mutiso 2022, Kenya	544	11	0.81 (0.71-0.89)	0.83(0.79-0.86)	64	15	81	384	

*Deduced from FPR: 1-FPR

2. **Tables for sub-group analysis for PHQ-9 / PHQ-2/9**

Table 2.1: Outputs from the HSROC Meta-analysis model for PHQ-9 administered in one-step for all population categories (patients and their respective accompaniers, prenatal / postnatal women, adolescents and students)

Study author, country	Sample size (N)	Cut-off	Se (95%CI)	Sp (95% CI)	TP	FN	FP	TN	Key outputs of HSROC model
Gelaye 2013, Ethiopia	926	10	0.86 (0.78-0.92)	0.67(0.61-0.73)	40	6	290	590	1.Pooled se (95%CI): 0.79(0.72 - 0.84)<br>2.Pooled FPR (95%CI): 0.18(0.13- 0.24)<br>3.Pooled sp(95%CI)*: 0.82 (0.76– 0.87)<br>4.AUC: 0.87<br>5.Partial AUC (restricted to FPR): 0.75<br>6.Between study std.dev for the logit-transformed se: 0.81<br>7.Between study std.dev for the logit-transformed FPR: 0.99<br>8.rho between the logit-transformed se and the logit-transformed sp: 0.58<br>9.I^2 estimates by Holling sample size unadjusted approaches: 43.1– 77.6 %
Stockton 2024a, South Africa	1885	5	0.84 (0.80- 0.88)	0.67 (0.65-0.70)	354	66	480	985	
Stockton 2024b, South Africa	1885	10	0.49 (0.44–0.54)	0.90 (0.89–0.92)	198	207	144	1336	
Adewuya 2006a, Nigeria	512	5	0.90	0.99	23	3	5	481	
Adewuya 2006b, Nigeria	512	10	0.85	0.99	11	2	3	496	
Weobong 2008, Ghana	160	4/5	0.94	0.75	17	1	36	107	
Akena 2013, Uganda	368	10	0.92	0.81	59	5	58	246	
Bhana 2015, South Africa	676	9	0.49	0.94	38	39	38	561	
Hanlon 2015, Ethiopia	306	5	0.83	0.75	15	3	73	215	
Chibanda 2016, Zimbabwe	264	11	0.85(0.78-0.90)	0.69 (0.59–0.77)	44	8	66	146	
Nakku 2016, Uganda	153	5	0.674	0.781	57	27	15	54	
Green 2018, Kenya	193	15	0.70	0.74	7	3	48	135	
Woldetensay 2018, Ethiopia	216	8	0.81	0.79	23	5	39	149	

Study author, country	Sample size (N)	Cut-off	Se (95%CI)	Sp (95% CI)	TP	FN	FP	TN	Key outputs of HSROC model
Smith-Fawzi 2019, Tanzania	174	9	0.78 (0.52-0.94)	0.87 (0.80-0.92)	14	4	20	136	
Cumbe 2020, Mozambique	502	9	0.47	0.94	20	23	30	429	
Degefa 2020, 2020	163	4	0.88	0.78	22	3	30	108	
Molebatsi 2020, Botswana	257	9	0.72(0.63-0.81)	0.76 (0.69–0.83)	76	29	36	116	
Sebera 2020a, Rwanda	434	5	0.72	0.70	89	34	95	216	
Sebera 2020b, Rwanda	434	5	0.89	0.59	63	8	148	215	
Sebera 2020c, Rwanda	434	7	0.94	0.65	59	4	129	242	
Pence 2012, Cameroon	400	10	0.27 (0.06-0.61)	0.94 (0.91-0.96)	3	8	23	366	
Lovero 2022, Mozambique	485	8	0.78	0.80	32	9	89	355	
Marlow 2022, South Africa	302	10	0.91	0.76	21	2	67	212	
Tele 2023a, Kenya	250	9	0.95	0.73	18	1	62	169	
Tele 2023b, Kenya	250	9	0.89	0.70	17	2	69	162	

- *Deduced from FPR: 1-FPR

Table 2.2: Outputs from the HSROC meta-analysis model for PHQ-9 in general for clinical populations (and their accompaniers)

Study author, country	Sample size (N)	Cut-off	Se (95%CI)	Sp (95% CI)	TP	FN	FP	TN	Key outputs of HSROC model
Gelaye 2013, Ethiopia	926	10	0.86 (0.78-0.92)	0.67(0.61-0.73)	40	6	290	590	1.Pooled se (95%CI): 0.72(0.64 - 0.79)<br>2.Pooled FPR (95%CI): 0.20(0.15- 0.26)<br>3.Pooled sp(95%CI)*: 0.80 (0.74– 0.85)<br>4.AUC: 0.83<br>5.Partial AUC (restricted to FPR): 0.72<br>6.Between study std.dev for the logit-transformed se: 0.81<br>7.Between study std.dev for the logit-transformed FPR: 0.74<br>8.rho between the logit-transformed se and the logit-transformed sp: 1<br>9.I^2 estimates by Holling sample size unadjusted approaches: 47.1– 80.1%
Stockton 2024a, South Africa	1885	5	0.84 (0.80- 0.88)	0.67 (0.65-0.70)	354	66	480	985	
Stockton 2024b, South Africa	1885	10	0.49 (0.44–0.54)	0.90 (0.89–0.92)	198	207	144	1336	
Akena 2013, Uganda	368	10	0.92	0.81	59	5	58	246	
Bhana 2015, South Africa	676	9	0.49	0.94	38	39	38	561	
Hanlon 2015, Ethiopia	306	5	0.83	0.75	15	3	73	215	
Chibanda 2016, Zimbabwe	264	11	0.85(0.78-0.90)	0.69 (0.59–0.77)	44	8	66	146	
Nakku 2016, Uganda	153	5	0.674	0.781	57	27	15	54	
Green 2018, Kenya	193	15	0.70	0.74	7	3	48	135	
Smith-Fawzi 2019, Tanzania	174	9	0.78 (0.52-0.94)	0.87 (0.80-0.92)	14	4	20	136	
Cumbe 2020, Mozambique	502	9	0.47	0.94	20	23	30	429	
Degefa 2020, 2020	163	4	0.88	0.78	22	3	30	108	
Molebatsi 2020, Botswana	257	9	0.72(0.63-0.81)	0.76 (0.69–0.83)	76	29	36	116	

**Study author, country**	**Sample size (N)**	**Cut-off**	**Se (95%CI)**	**Sp (95% CI)**	**TP**	**FN**	**FP**	**TN**	**Key outputs of HSROC model**
Sebera 2020a, Rwanda	434	5	0.72	0.70	89	34	95	216	
Sebera 2020b, Rwanda	434	5	0.89	0.59	63	8	148	215	
Sebera 2020c, Rwanda	434	7	0.94	0.65	59	4	129	242	
Stockton 2024c, South Africa	1885	5	0.84 (0.80-0.87)	0.70 (0.67-0.72)	351	69	443	1022	
Stockton 2024d, South Africa	1885	10	0.49 (0.44-0.54)	0.91 (0.89-0.92)	351	69	134	1331	
Pence 2012, Cameroon	400	10	0.27 (0.06-0.61)	0.94 (0.91-0.96)	3	8	23	366	

*Deduced from FPR: 1-FPR

Table 2.3: Outputs from the HSROC Meta-analysis model for one-step PHQ-9 administered in clinical population

Study author, country	Sample size (N)	Cut-off	Se (95%CI)	Sp (95% CI)	TP	FN	FP	TN	Key outputs of HSROC model
Gelaye 2013, Ethiopia	926	10	0.86 (0.78-0.92)	0.67(0.61-0.73)	40	6	290	590	1.Pooled se (95%CI): 0.72(0.64 - 0.79)<br>2.Pooled FPR (95%CI): 0.20(0.15- 0.26)<br>3.Pooled sp(95%CI)*: 0.80 (0.74– 0.85)<br>4.AUC: 0.83<br>5.Partial AUC (restricted to FPR): 0.72<br>6.Between study std.dev for the logit-transformed se: 0.81<br>7.Between study std.dev for the logit-transformed FPR: 0.74<br>8.rho between the logit-transformed se and the logit-transformed sp: 1<br>9.I^2 estimates by Holling sample size unadjusted approaches: 47.1– 80.1%
Stockton 2024a, South Africa	1885	5	0.84 (0.80- 0.88)	0.67 (0.65-0.70)	354	66	480	985	
Stockton 2024b, South Africa	1885	10	0.49 (0.44–0.54)	0.90 (0.89–0.92)	198	207	144	1336	
Akena 2013, Uganda	368	10	0.92	0.81	59	5	58	246	
Bhana 2015, South Africa	676	9	0.49	0.94	38	39	38	561	
Hanlon 2015, Ethiopia	306	5	0.83	0.75	15	3	73	215	
Chibanda 2016, Zimbabwe	264	11	0.85(0.78-0.90)	0.69 (0.59–0.77)	44	8	66	146	
Nakku 2016, Uganda	153	5	0.674	0.781	57	27	15	54	
Green 2018, Kenya	193	15	0.70	0.74	7	3	48	135	
Smith-Fawzi 2019, Tanzania	174	9	0.78 (0.52-0.94)	0.87 (0.80-0.92)	14	4	20	136	
Cumbe 2020, Mozambique	502	9	0.47	0.94	20	23	30	429	
Degefa 2020, 2020	163	4	0.88	0.78	22	3	30	108	
Molebatsi 2020, Botswana	257	9	0.72(0.63-0.81)	0.76 (0.69–0.83)	76	29	36	116	
Sebera 2020a, Rwanda	434	5	0.72	0.70	89	34	95	216	

Study author, country	Sample size (N)	Cut-off	Se (95%CI)	Sp (95% CI)	TP	FN	FP	TN	Key outputs of HSROC model
Sebera 2020b, Rwanda	434	5	0.89	0.59	63	8	148	215	
Sebera 2020c, Rwanda	434	7	0.94	0.65	59	4	129	242	
Pence 2012, Cameroon	400	10	0.27 (0.06-0.61)	0.94 (0.91-0.96)	3	8	23	366	

*Deduced from FPR: 1-FPR

Table 2.4: Outputs from the HSROC meta-analysis model for one-step PHQ-9 for non-clinical populations

**Study author, country**	**Sample size (N)**	**Cut-off**	**Se (95%CI)**	**Sp (95%CI)**	**TP**	**FN**	**FP**	**TN**	**Key outputs of HSROC model**[1]
Adewuya 2006, Nigeria	512	5	0.90	0.99	23	3	5	481	1.Pooled se (95%CI): 0.85 (0.79-0.90)<br>2.Pooled FPR (95%CI): 0.12 (0.04-0.30)<br>3.Pooled sp(95%CI)*: 0.88 (0.70 – 0.96)<br>4.AUC: 0.85<br>5.Partial AUC (restricted to FPR): 0.84<br>6.Between study std.dev for the logit-transformed se: 0.03<br>7.Between study std.dev for the logit-transformed FPR: 1.62<br>8.rho between se and sp: -1<br>9. I^2 estimates by Holling sample size unadjusted approaches: 26.5 - 36.6 %
Adewuya 2006, Nigeria	512	10	0.85	0.99	11	2	3	496	
Weobong 2008, Ghana	160	4/5	0.94	0.75	17	1	36	107	
Woldetensay 2018, Ethiopia	216	8	0.81	0.79	23	5	39	149	
Lovero 2022, Mozambique	485	8	0.78	0.80	32	9	89	355	
Marlow 2022, South Africa	302	10	0.91	0.76	21	2	67	212	
Tele 2023, Kenya	250	9	0.95	0.73	18	1	62	169	
Tele 2023, Kenya	250	9	0.89	0.70	17	2	69	162	

*Deduced from FPR: 1-FPR

Table 3.1: Outputs from the HSROC meta-analysis model for PHQ-2 for depression validated with optimal reference standards only

	**Sample size (N)**	**Cut-off**	**Se (95%CI)**	**Sp (95%CI)**	**TP**	**FN**	**FP**	**TN**	**Key outputs of HSROC model1**
Stockton 2024, South Africa	1885	>0	0.94 (0.92-0.96)	0.43(0.41 -0.46)	396	24	830	635	1.Pooled se (95%CI): 0.81 (0.67-0.89)<br>2. Pooled FPR (95%CI): 0.38 (0.27-0.51)<br>3.Pooled sp(95%CI)*: 0.62 (0.49 – 0.73)<br>4.AUC: 0.77<br>5.Partial AUC (restricted to FPR): 0.76<br>6.Between study std.dev for the logit-transformed se: 0.86<br>7.Between study std.dev for the logit-transformed FPR: 0.69<br>8.rho between the logit-transformed se and the logit-transformed sp: 0.88<br>9. I^2 estimate: 65.7-76.8 %
Bhana 2015, South Africa	676	2	0.60	0.84	47	31	96	503	
Hanlon 2015, Ethiopia	306	1	0.83	0.61	15	3	113	175	
Chibanda 2016, Zimbabwe	264	2	0.91 (0.86-0.95)	0.40 (0.31-0.50)	47	5	127	85	
Gelaye 2016, Ethiopia	363	3	0.74 (0.59-0.86)	0.60 (0.54-0.65)	34	12	128	189	
Nakku 2016, Uganda	153	1	0.66	0.59	56	28	28	41	
Cumbe 2020, Mozambique	502	2	0.74	0.72	32	11	130	329	

*Deduced from FPR: 1-FPR
